# Supplementary material for: Copper-catalyzed enantioselective Sonogashira-type oxidative cross-coupling of unactivated C(sp3)−H bonds with alkynes
Source: Nat Commun. 2019 Dec 12;10:5689. doi: 10.1038/s41467-019-13705-1 (PMC6908613; doi:10.1038/s41467-019-13705-1)
Supplement: Supplementary file 2 — Supplementary Information [file 41467_2019_13705_MOESM2_ESM.pdf]

Supplementary Information

Copper-Catalyzed Enantioselective Sonogashira-Type Oxidative Cross-Coupling of Unactivated C(sp^3)–H Bonds with Alkynes

Zhang et al.

Supplementary Table 1 HPLC data and optical rotation of **5a** in literatures.[1-4]

entry	absolute configuration of **5**	chiral resolution conditions	t_R (minor)	t_R (major)	[α]	references
1	***R*** (89% ee)	OD-H (*n* hexane/*i*-PrOH = 99.5/0.5; flow: 0.8 mL/min)	13.60	19.32	$[\alpha]_D^{27} = -12.8$ (*c* 0.71, $CHCl_3$).	this work
2	***R*** (82% ee)	OD-H (*n* hexane; flow: 0.5 mL/min)	20.67	26.00	--	*Chem. Eur. J.* **14**, 741-746 (2008)
3	***S*** (84% ee)	OD-H (*n* hexane; flow: 0.5 mL/min)	27.54	20.79	$[\alpha]_D^{20} = +12.1$ (*c* 1.85, $CHCl_3$)	*Chem. Eur. J.* **14**, 741-746 (2008)
4	***S*** (90% ee)	IA (*n* hexane; flow: 0.5 mL/min)	25.3	18.1	$[\alpha]_D^{20} = +12.7$ (*c* 0.70, CH_2Cl_2)	*Chem. Eur. J.* **18**, 9775-9779 (2012)
5	***S*** (74% ee)	OD-H (*n* hexane; flow: 0.5 mL/min)	55.0	44.0	$[\alpha]_D^{25} = +9.4$ (*c* 0.89, $CHCl_3$)	*Synlett* **29**, 2251-2256 (2018)
6	***R*** (98% ee)	ID [CO_2], 1.0 mL/min	24.5	22.9	$[\alpha]_D^{20} = -16.3$ (*c* 1.0, $CHCl_3$).	*J. Am. Chem. Soc.* **139**, 8448-8451 (2017)

Supplementary Figures

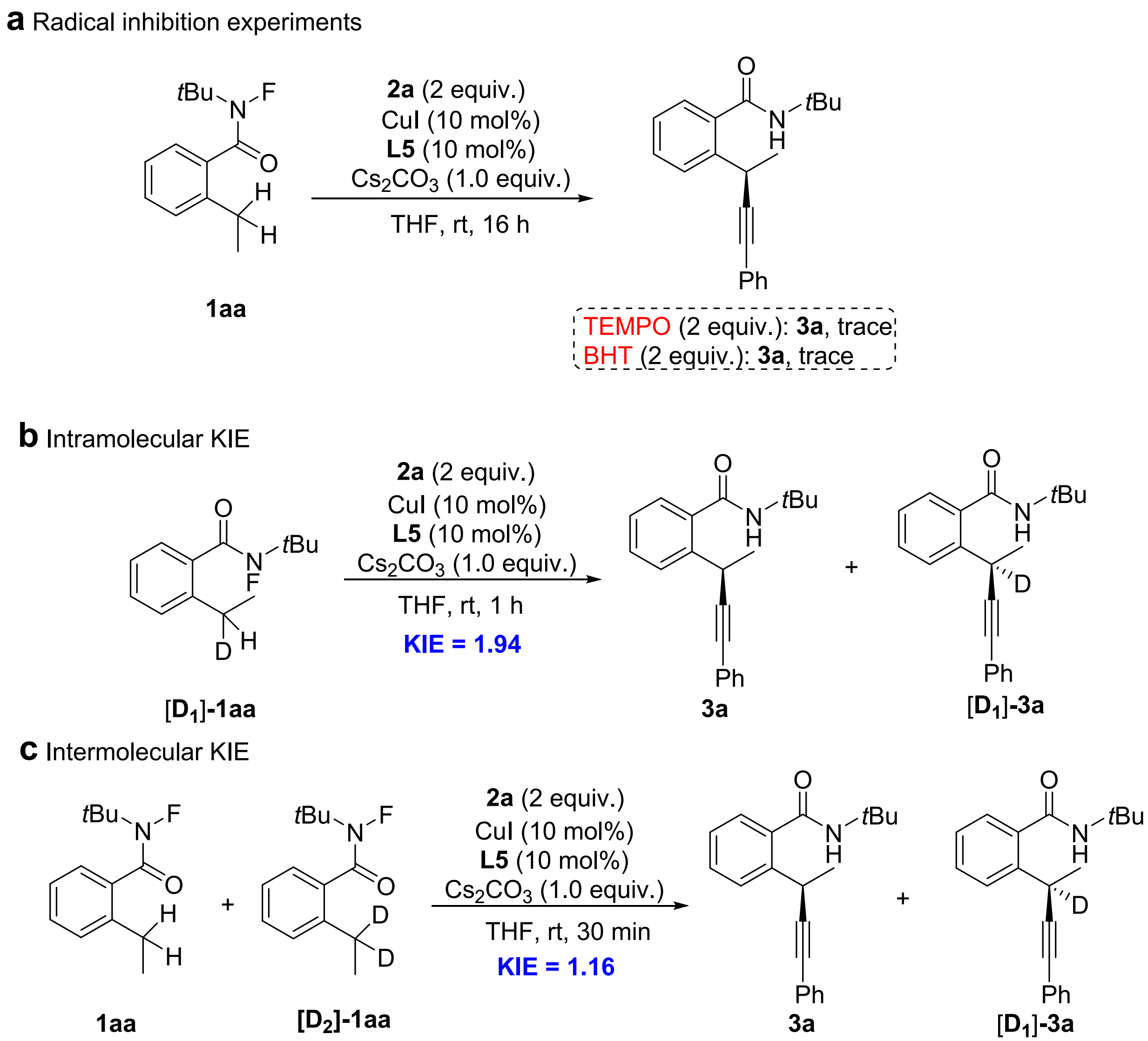

Supplementary Figure 1 Mechanistic studies of Sonogashira-type enantioselective oxidative cross-coupling reaction. **a** The reaction was significantly inhibited by addition of common radical inhibitors TEMPO and BHT. **b** and **c** An intramolecular KIE of 1.94 and an intermolecular KIE of 1.16 were observed, respectively, on substrate **1aa** and its corresponding deuterated derivatives, thus indicating the HAA step might not be involved in the rate-determing step(s).

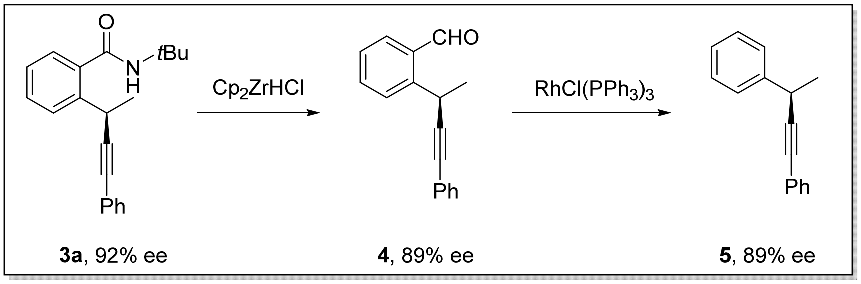

The absolute configuration of **3a** was determined by comparing the HPLC spectrum and specific rotation of prepared **5** with those reported in literature, as shown below.[1-4] The product **3a** was determined to be of an "*S*" absolute configuration according to the "*R*" absolute configuration of **5**.

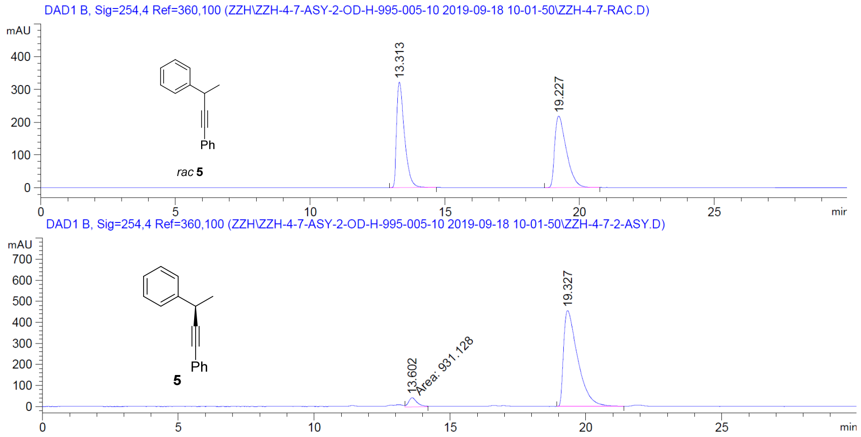

Supplementary Figure 2 Determination of absolute stereochemistry

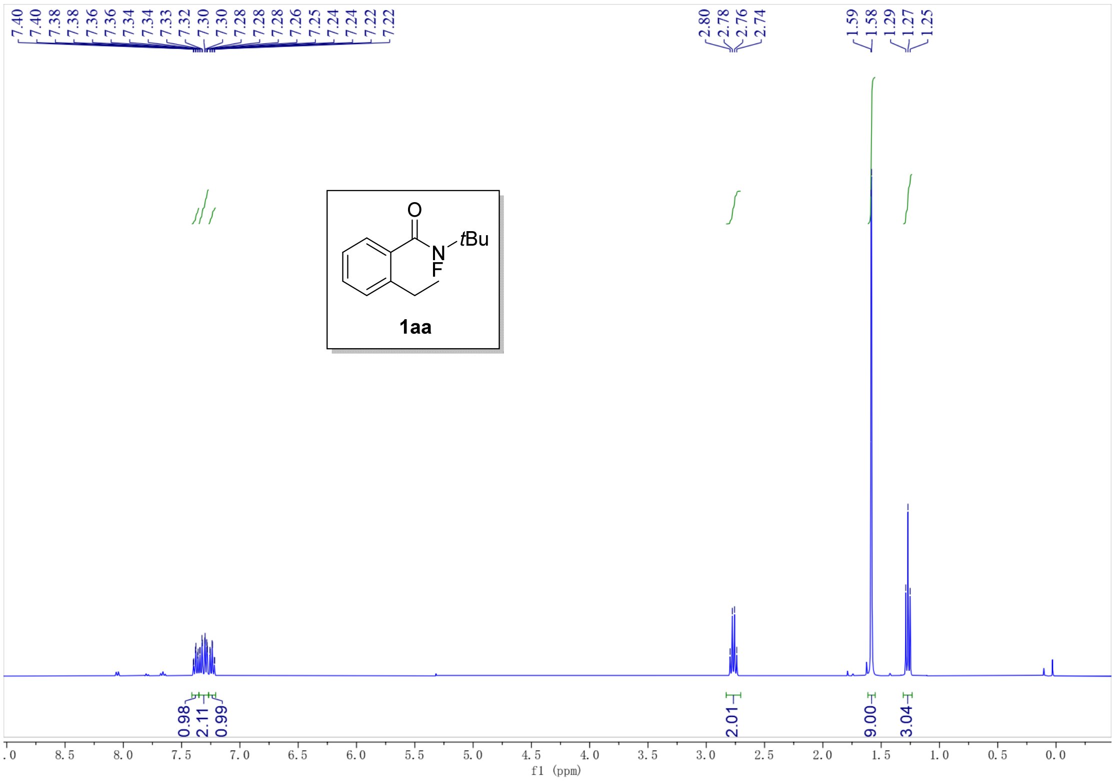

Supplementary Figure 3 ^{1}H NMR of **1aa**

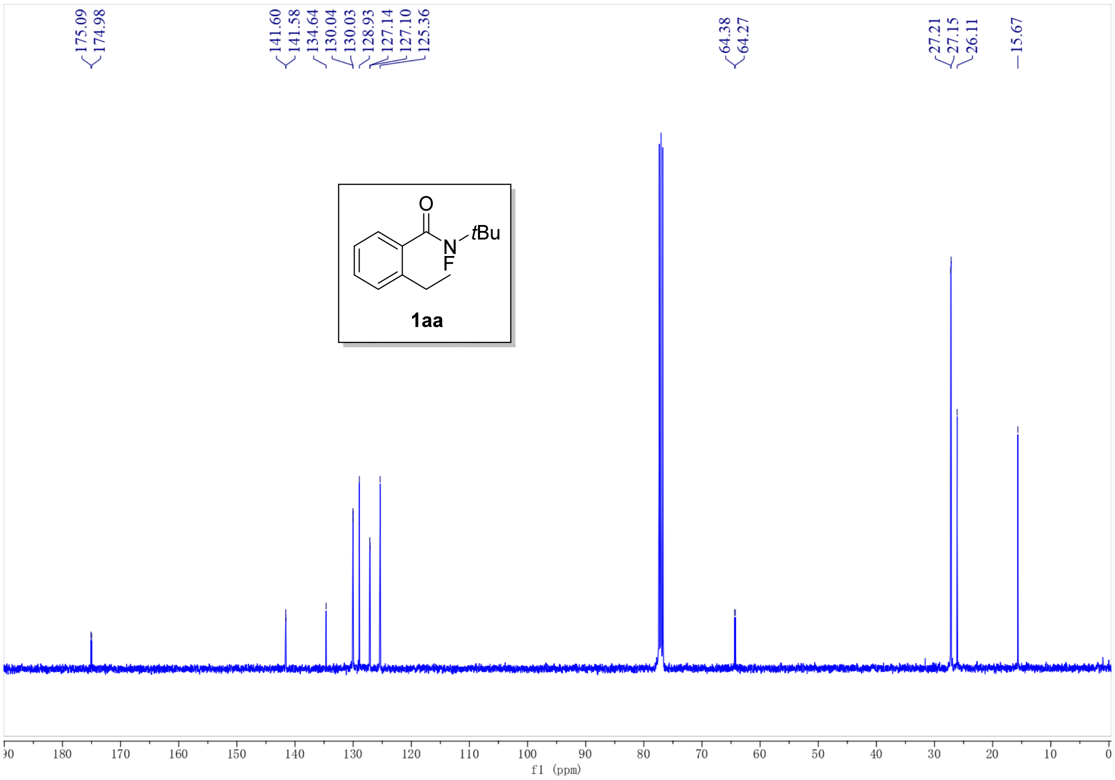

Supplementary Figure 4 ^{13}C NMR of **1aa**

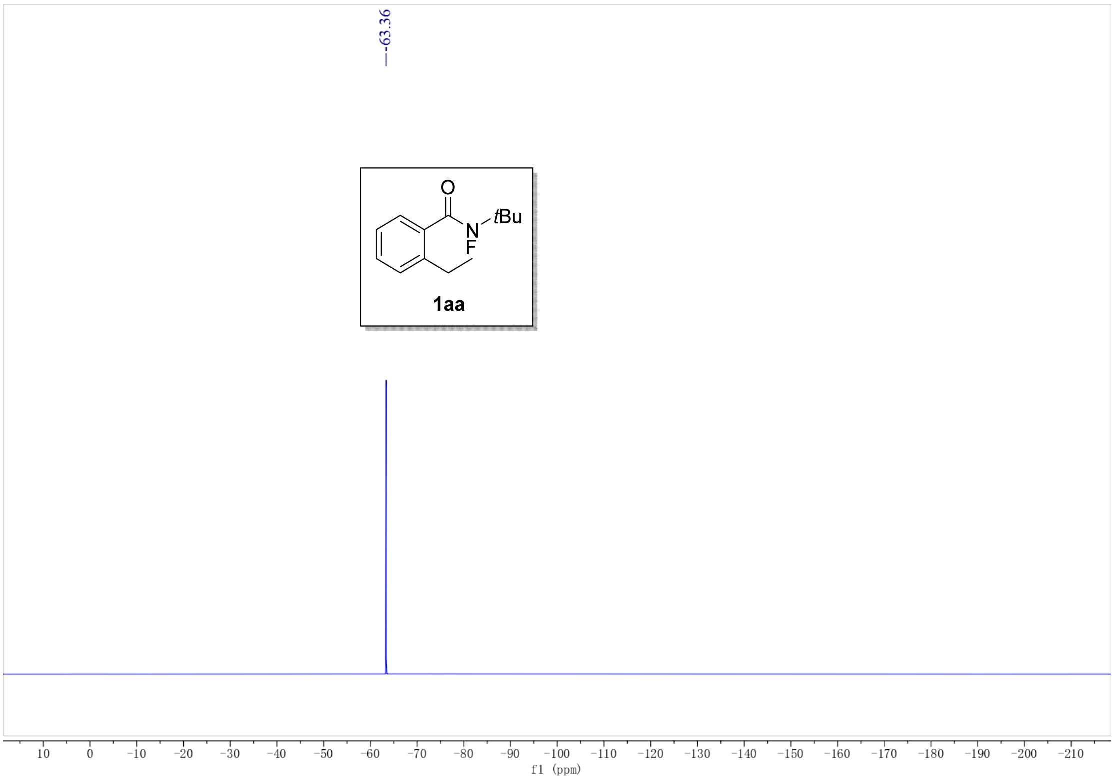

Supplementary Figure 5 ^{19}F NMR of **1aa**

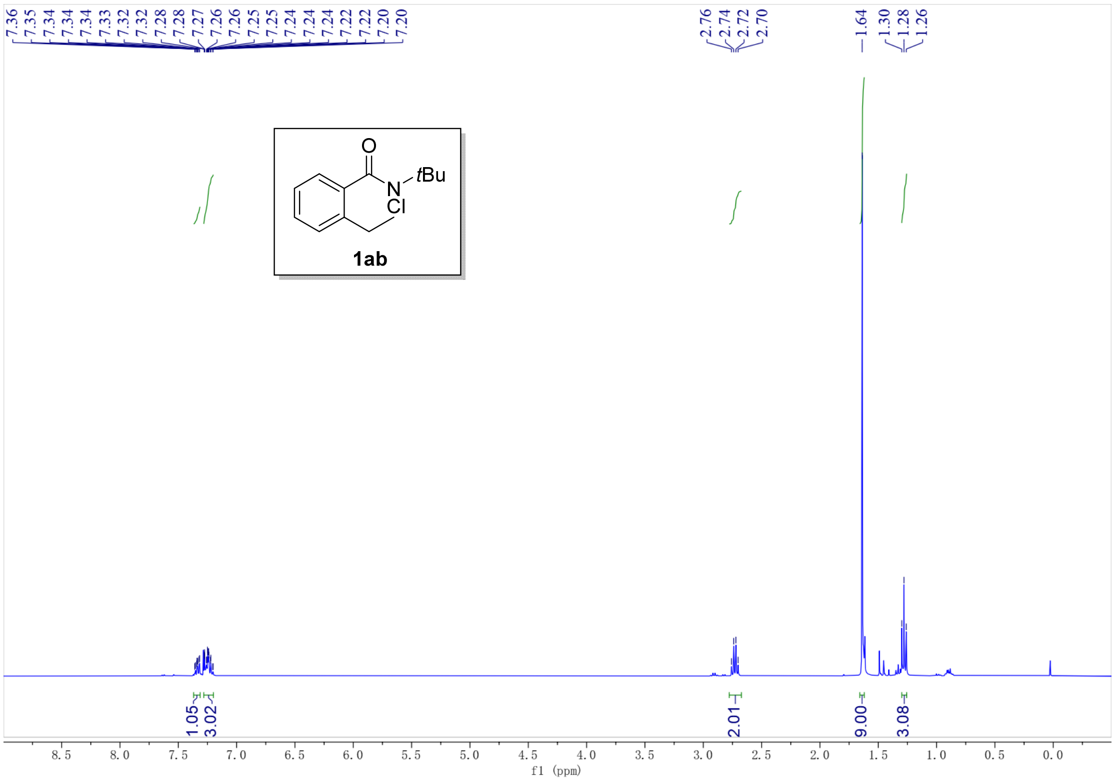

Supplementary Figure 6 ^{1}H NMR of **1ab**

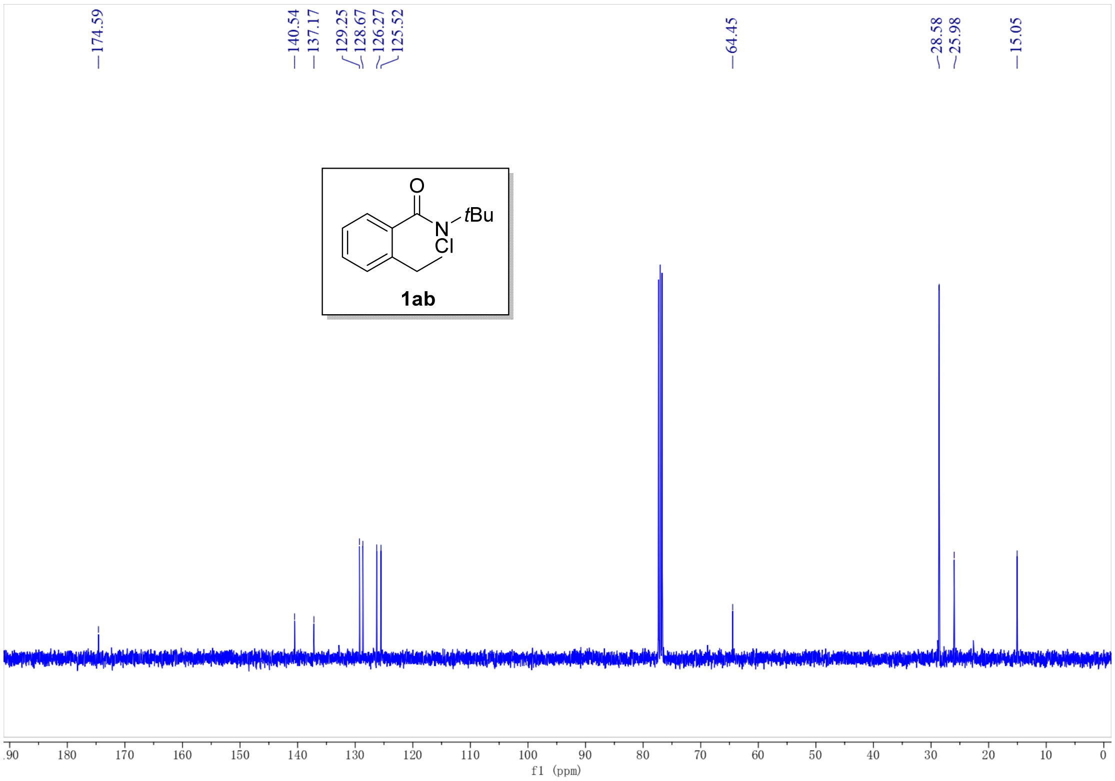

Supplementary Figure 7 ^{1}H NMR of **1ab**

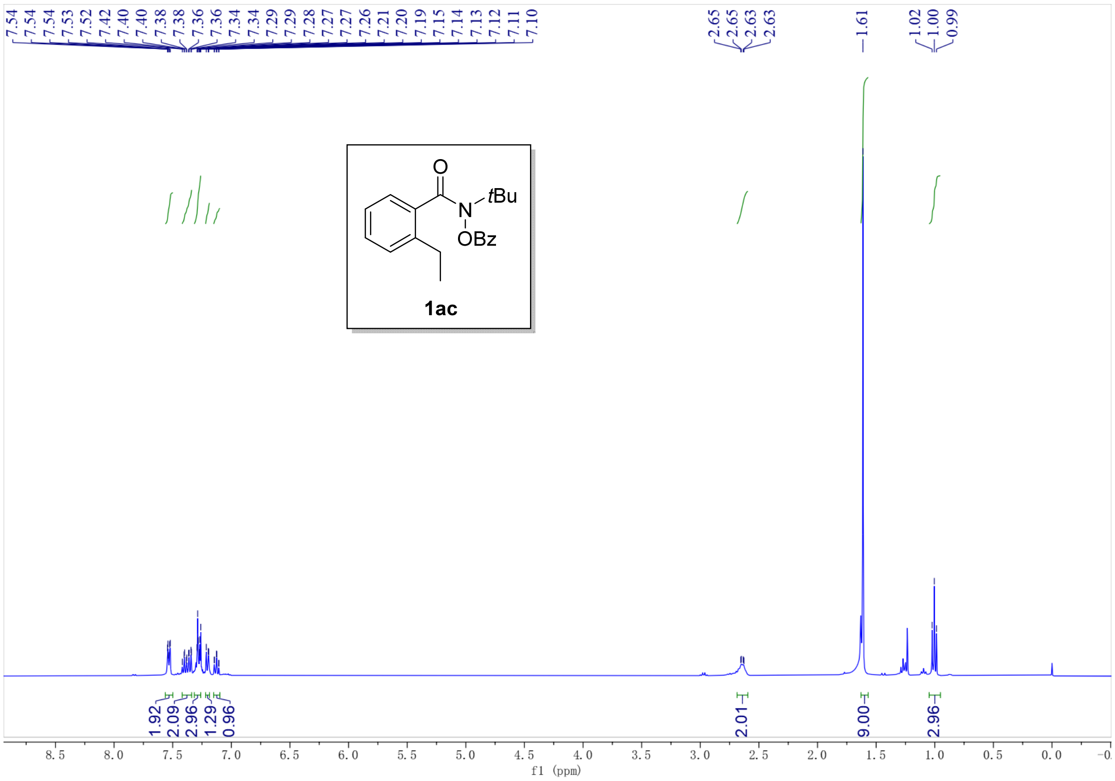

Supplementary Figure 8 ^{1}H NMR of **1ac**

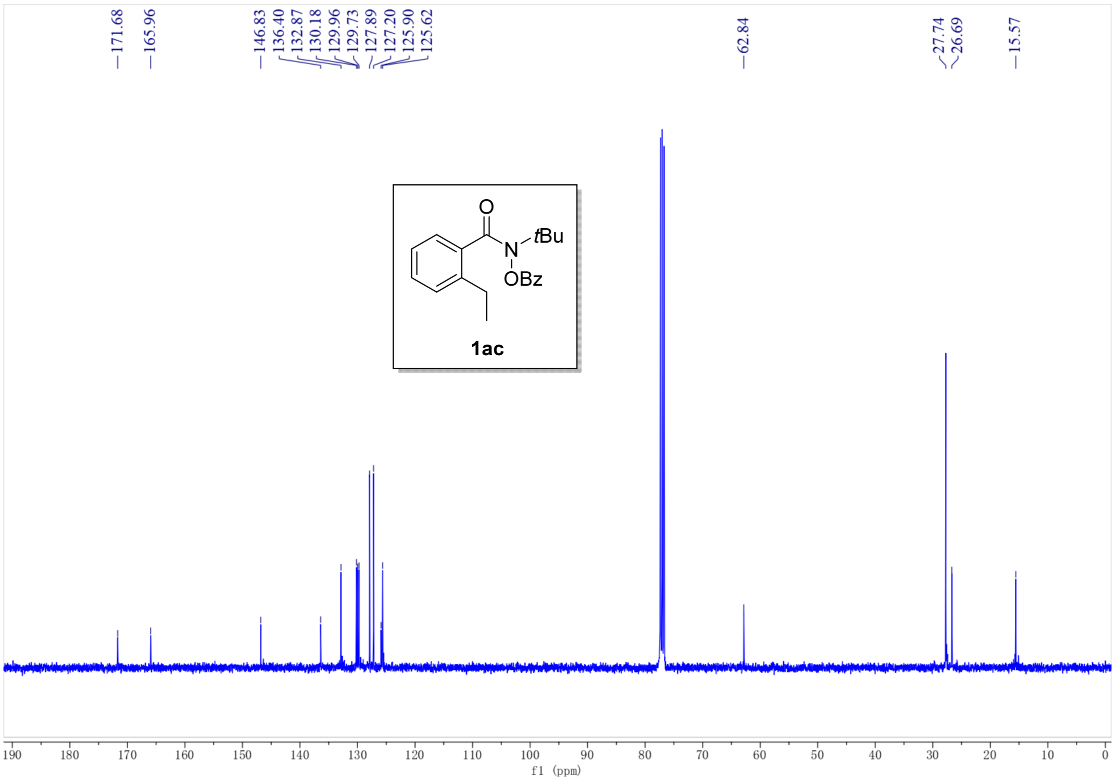

Supplementary Figure 9 ^{13}C NMR of **1ac**

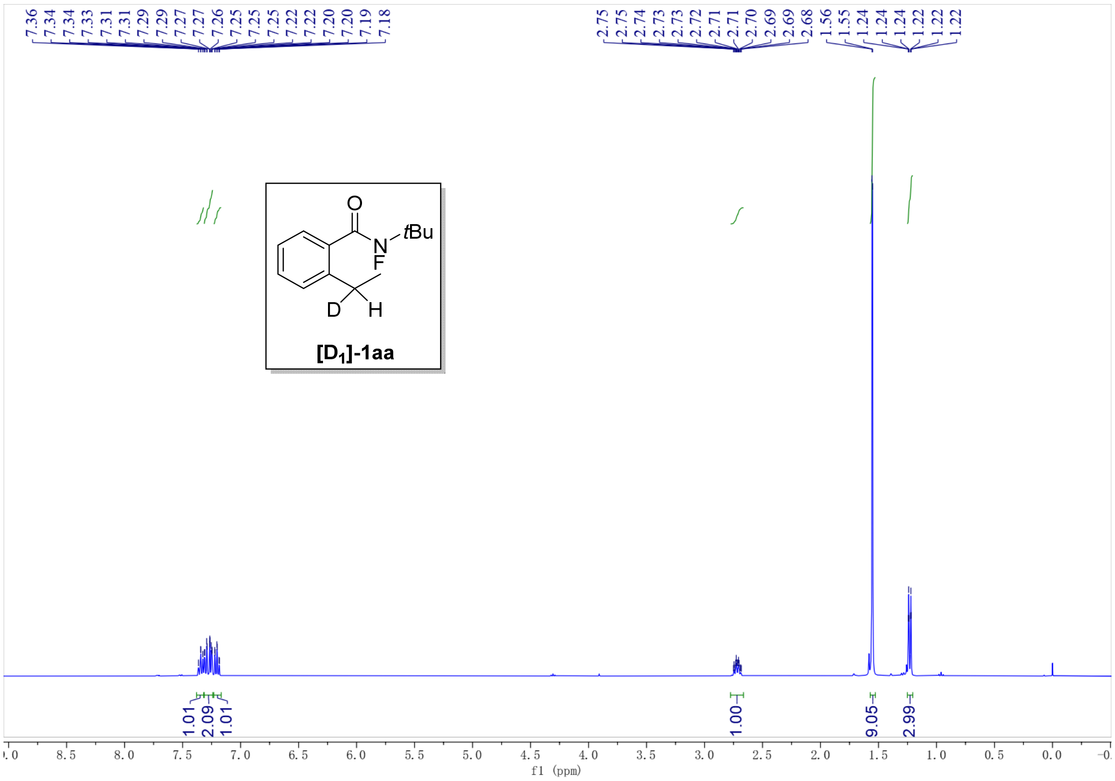

Supplementary Figure 10 ^{1}H NMR of **[D$_1$]-1aa**

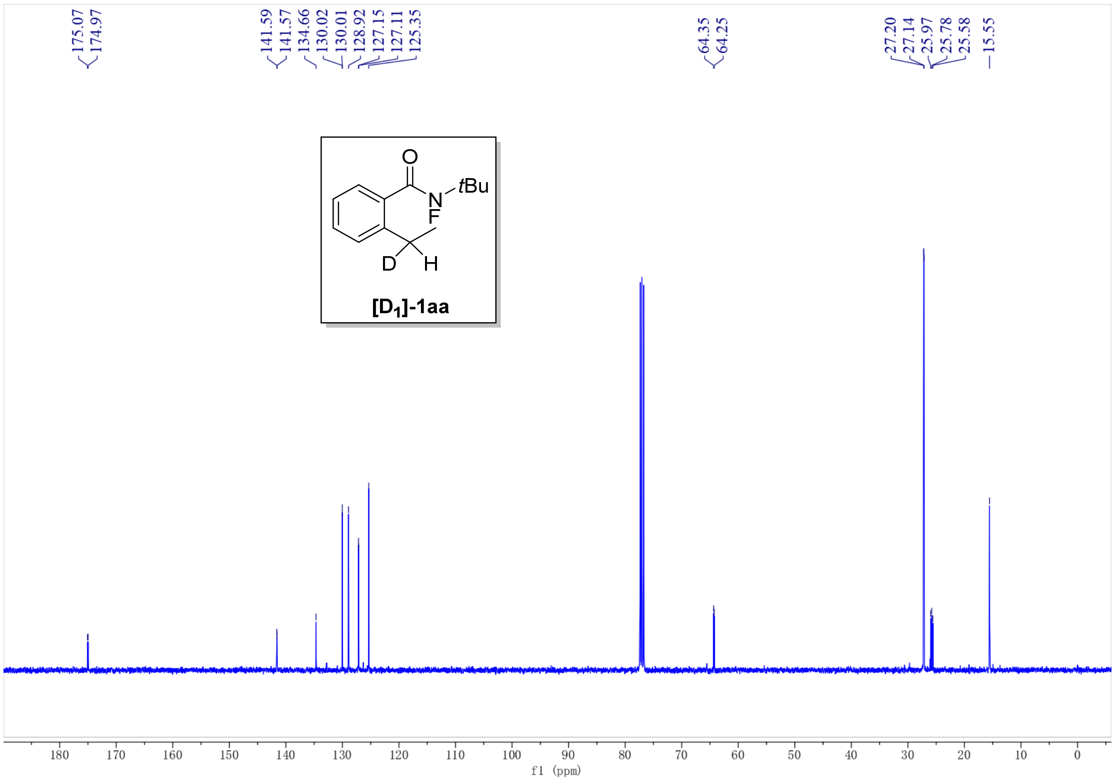

Supplementary Figure 11 ^{13}C NMR of **[D$_1$]-1aa**

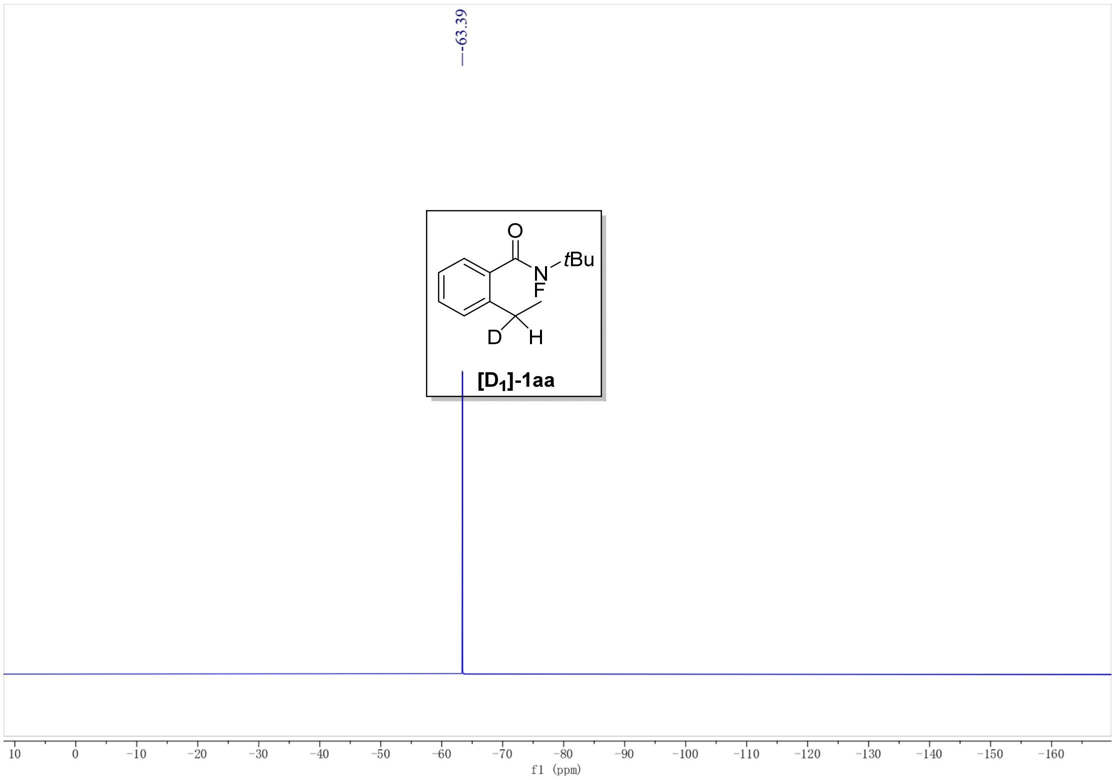

Supplementary Figure 12 ^{19}F NMR of **[D$_1$]-1aa**

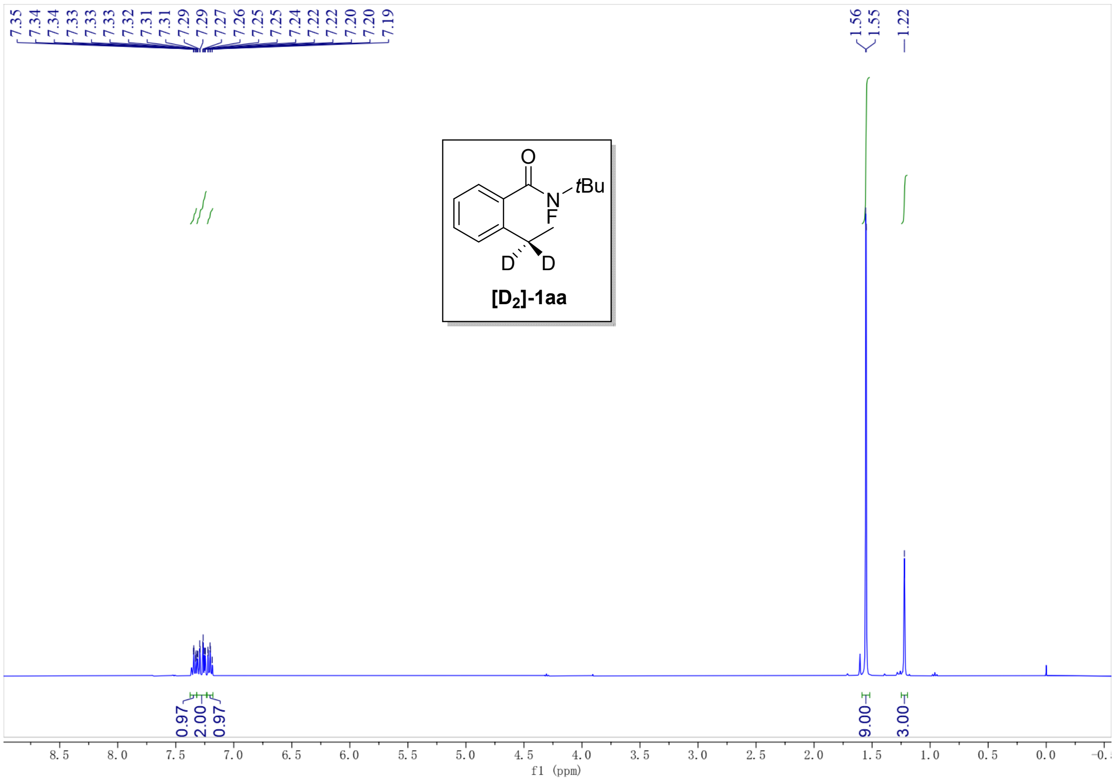

Supplementary Figure 13 ^{1}H NMR of **[D$_2$]-1aa**

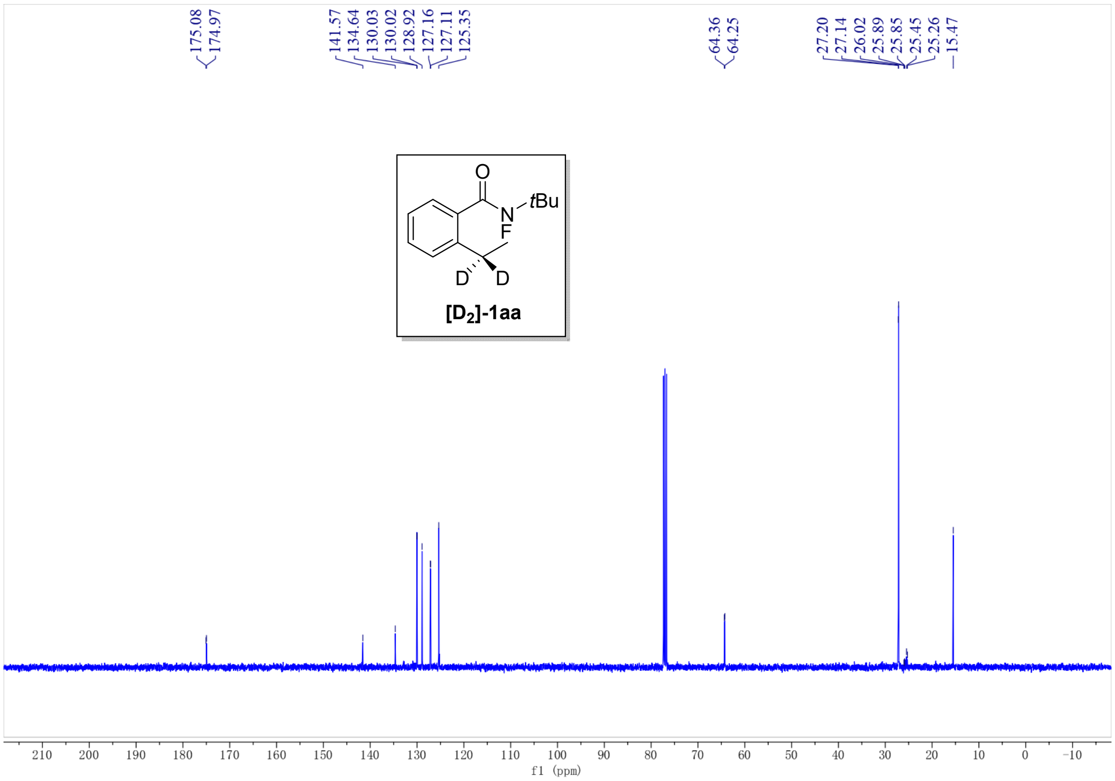

Supplementary Figure 14 ^{13}C NMR of **[D$_2$]-1aa**

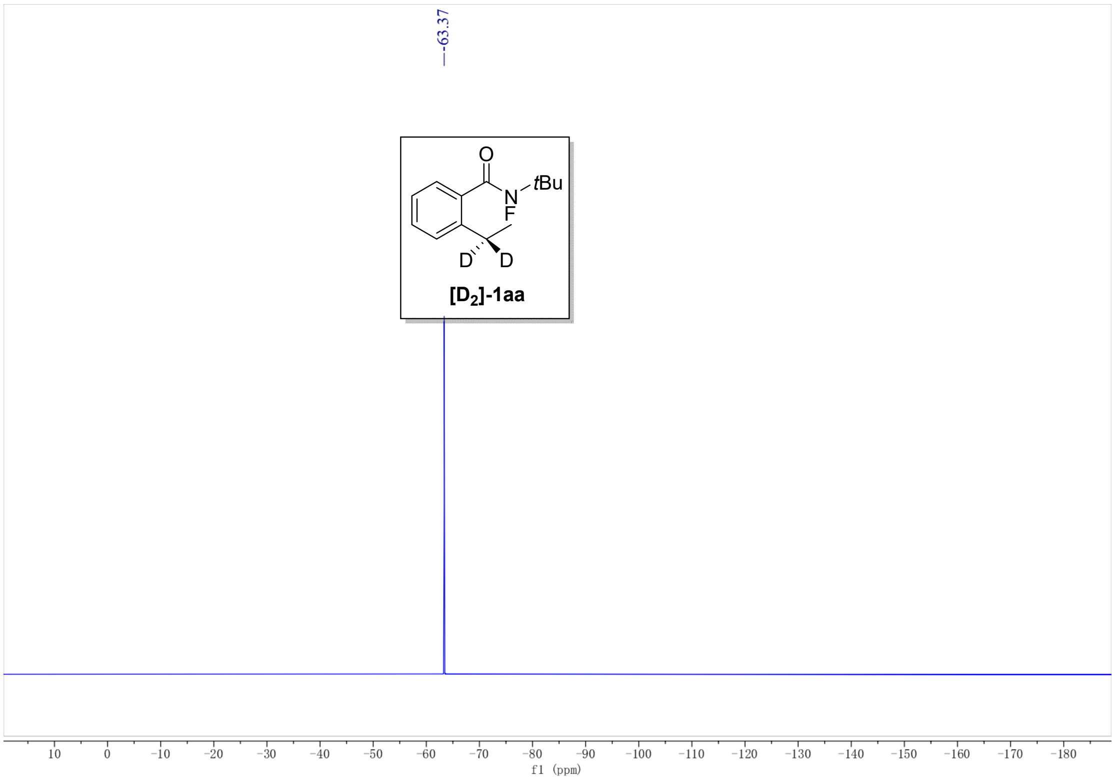

Supplementary Figure 15 ^{19}F NMR of **[D$_2$]-1aa**

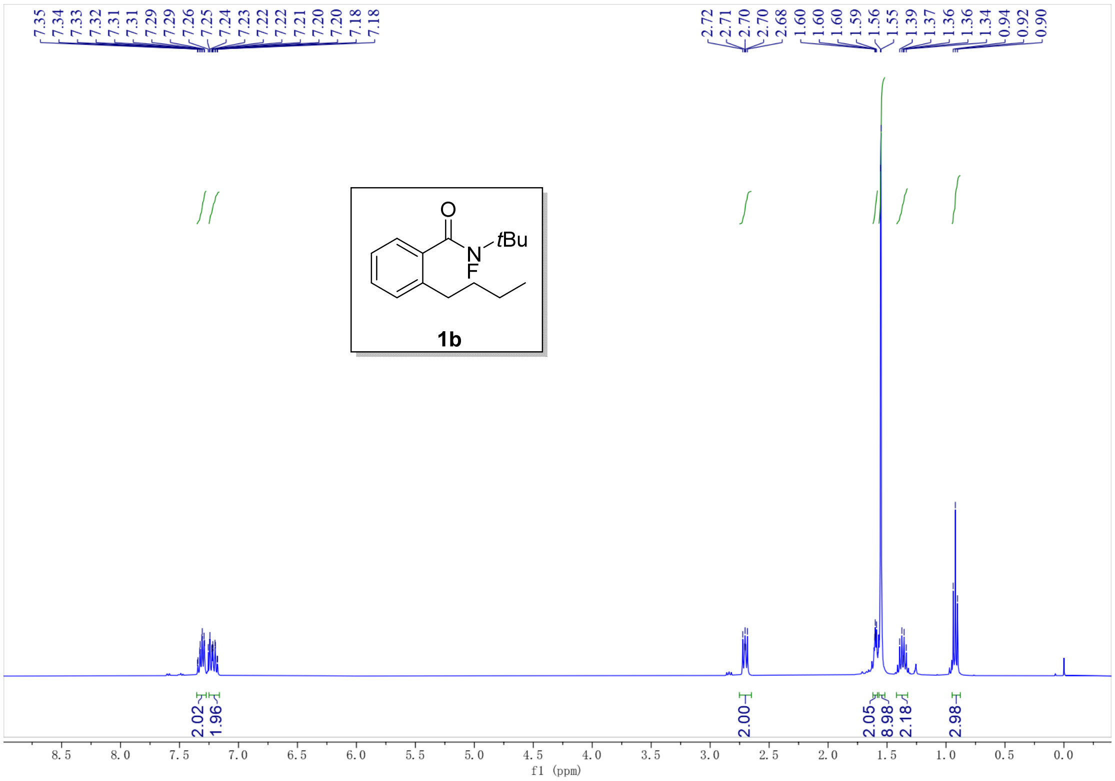

Supplementary Figure 16 ^{1}H NMR of **1b**

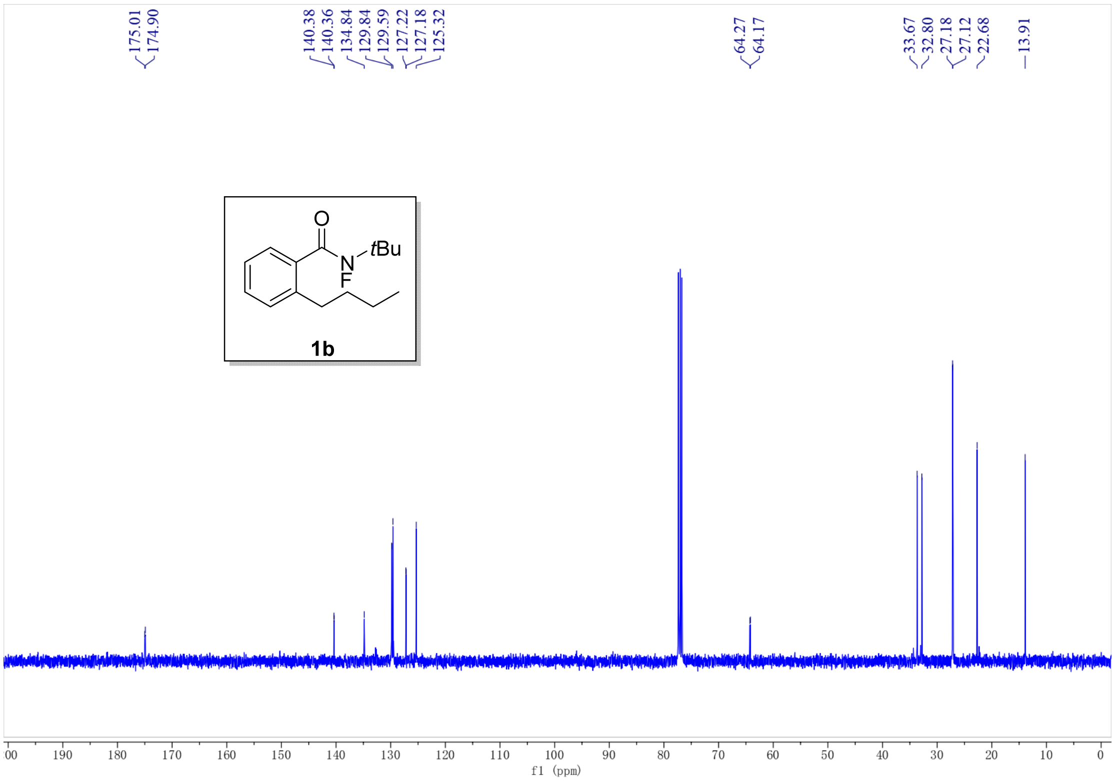

Supplementary Figure 17 ^{13}C NMR of **1b**

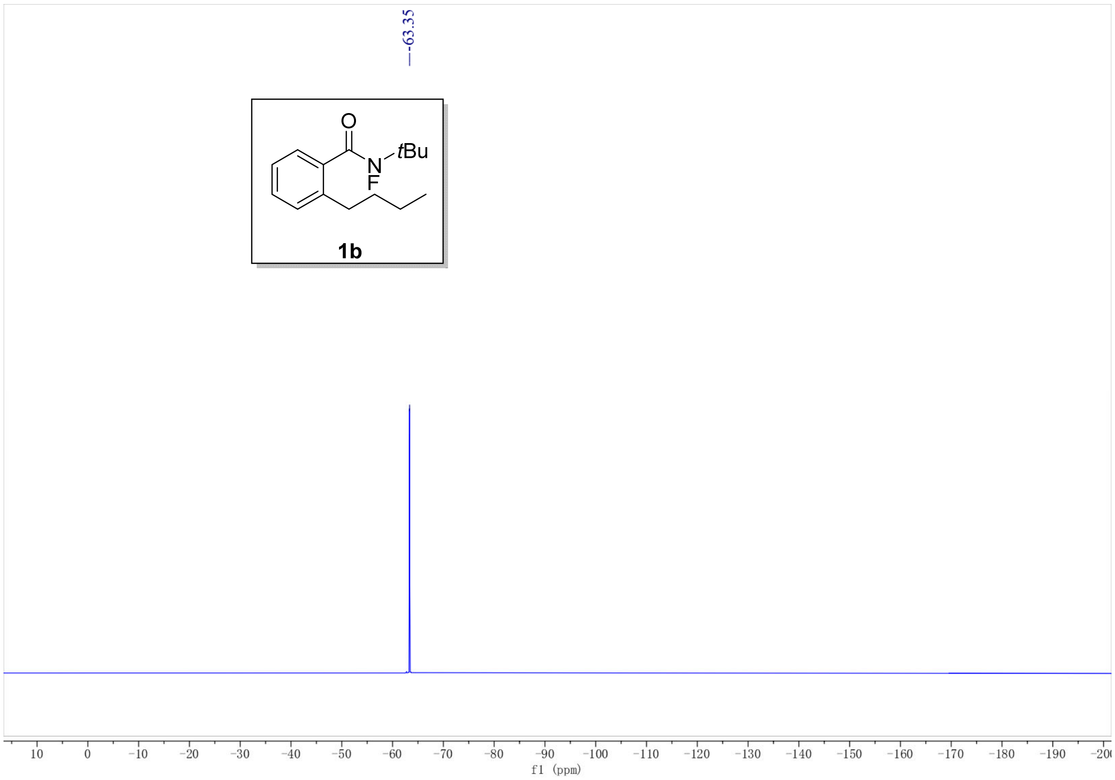

Supplementary Figure 18 ^{19}F NMR of **1b**

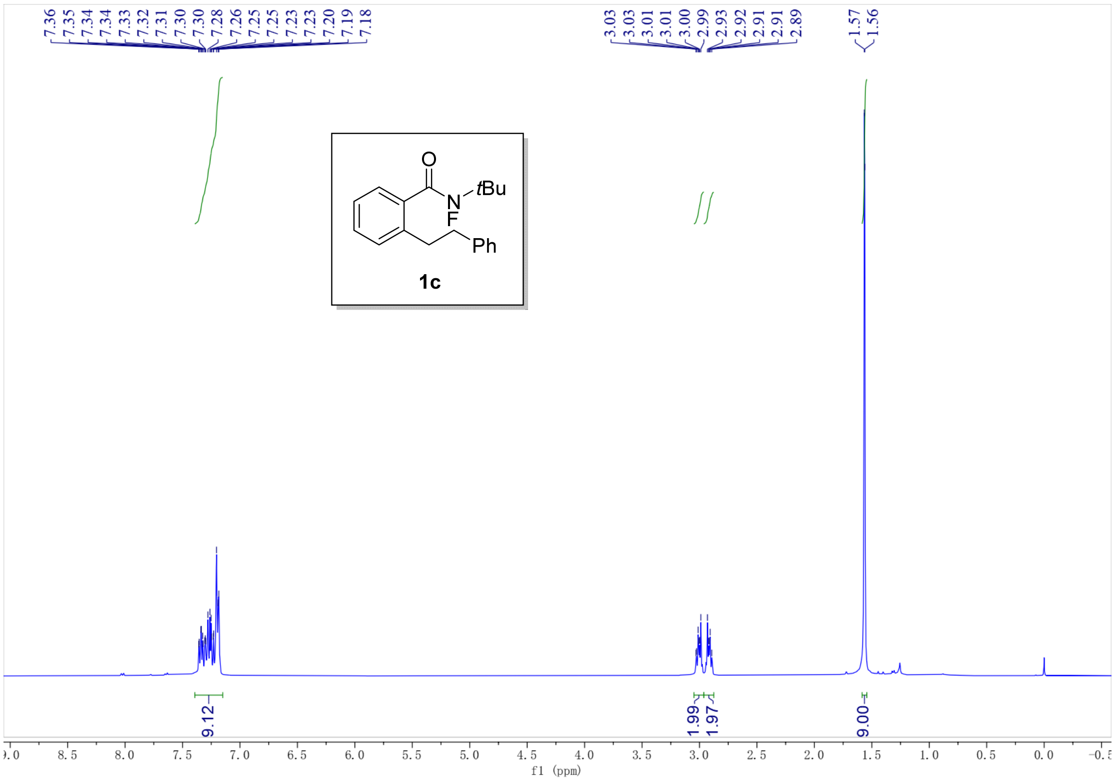

Supplementary Figure 19 ^{1}H NMR of **1c**

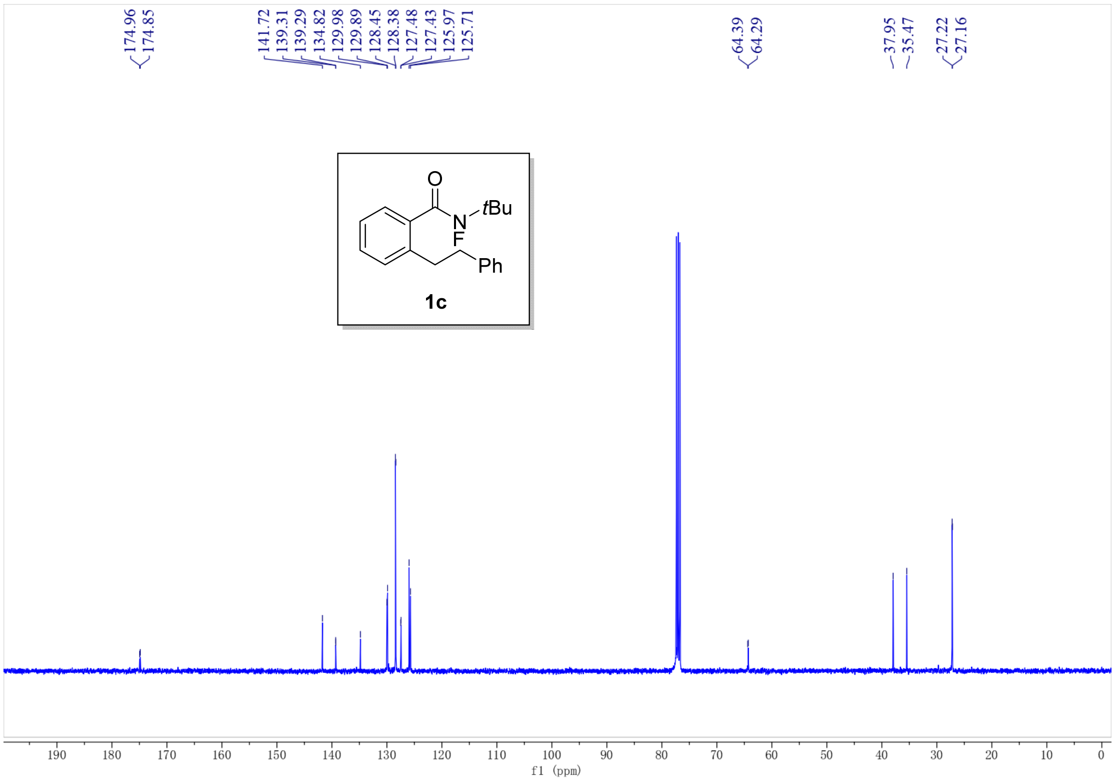

Supplementary Figure 20 ^{13}C NMR of **1c**

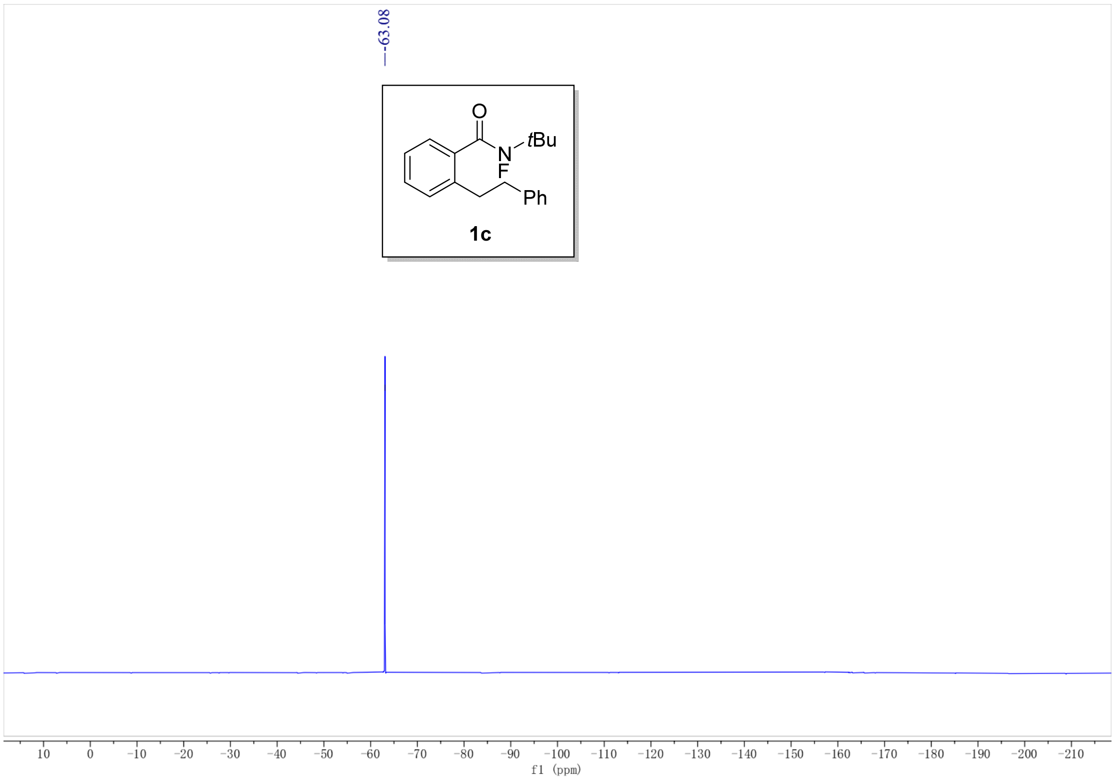

Supplementary Figure 21 ^{19}F NMR of **1c**

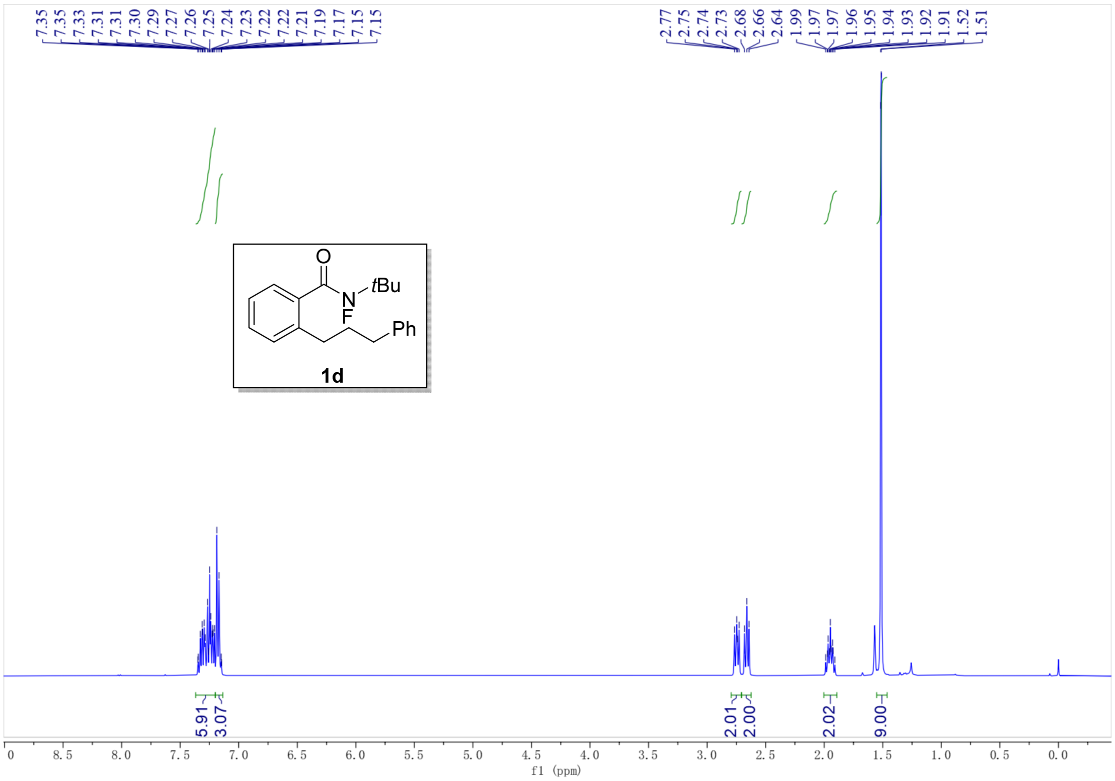

Supplementary Figure 22 ^{1}H NMR of **1d**

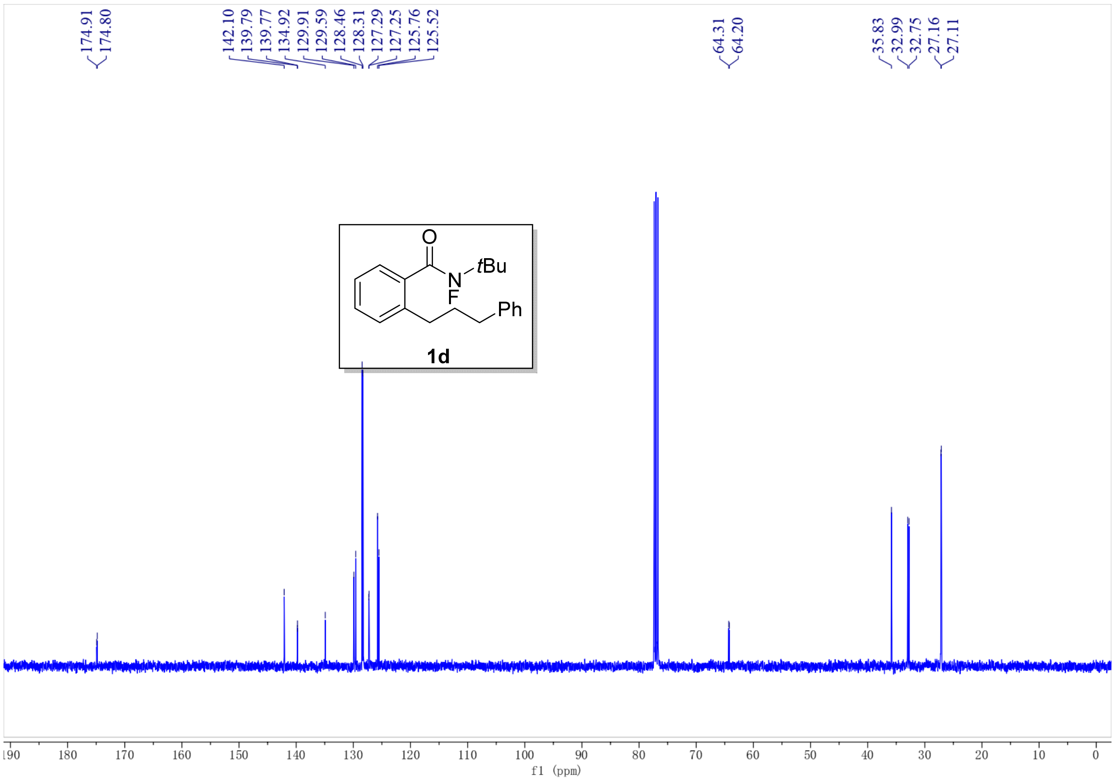

Supplementary Figure 23 ^{13}C NMR of **1d**

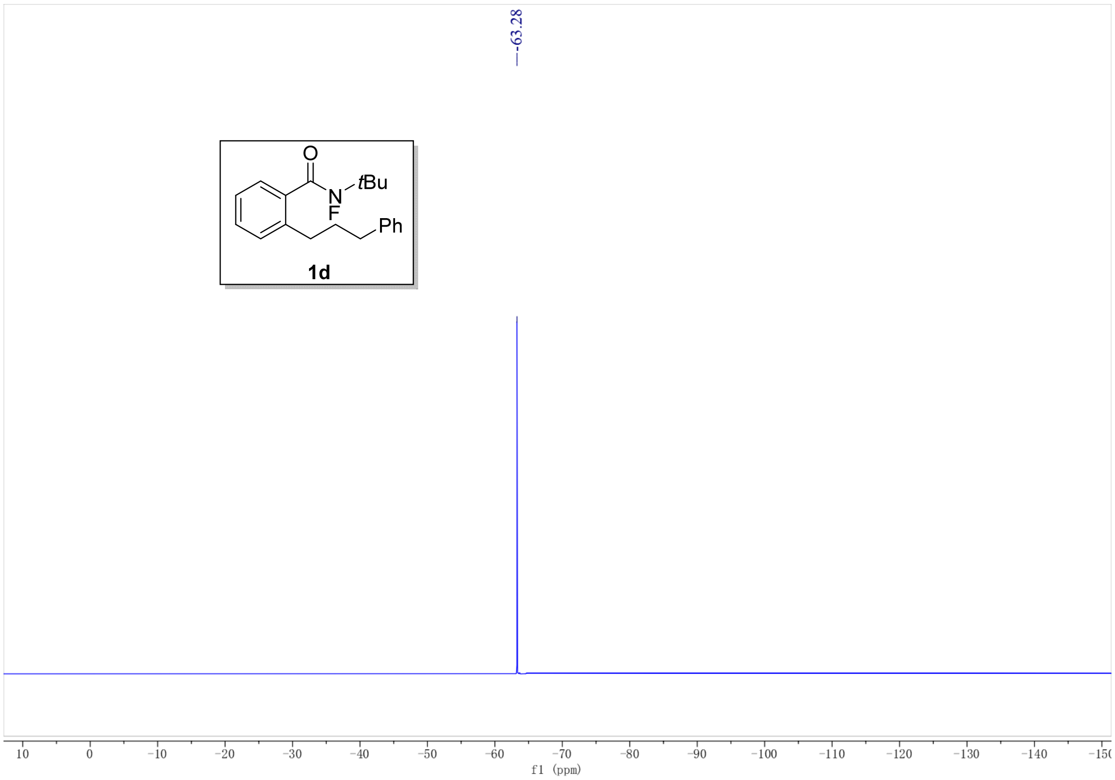

Supplementary Figure 24 ^{19}F NMR of **1d**

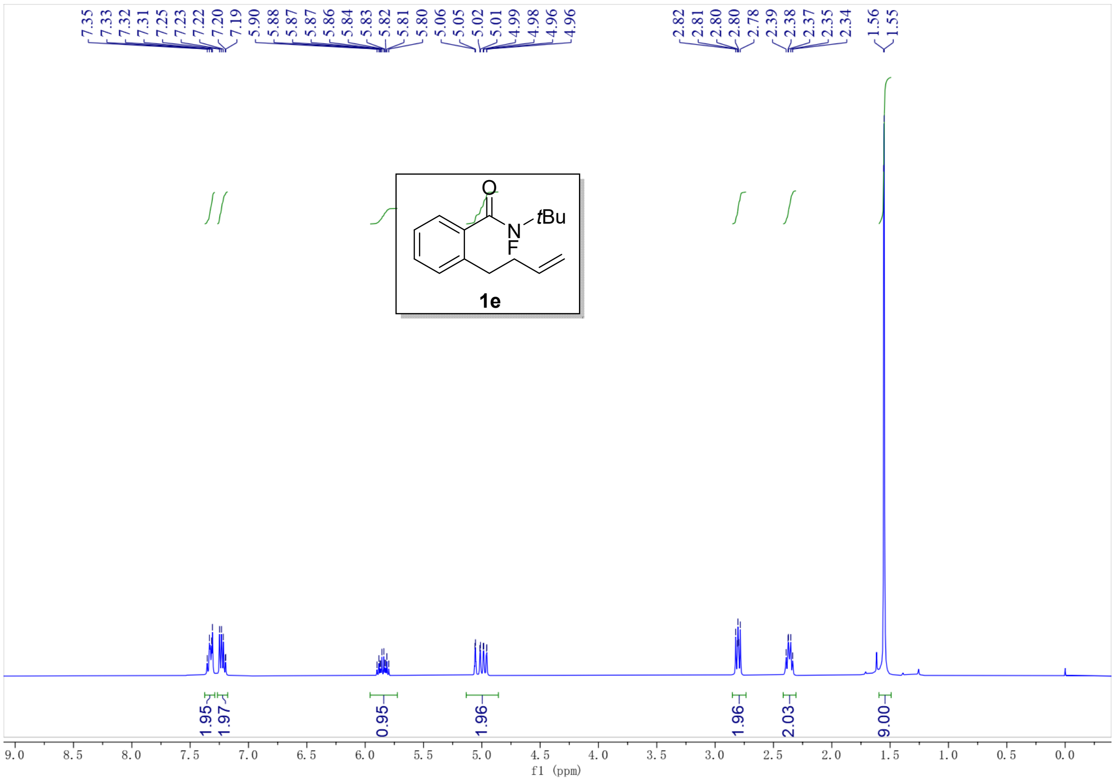

Supplementary Figure 25 ^{1}H NMR of **1e**

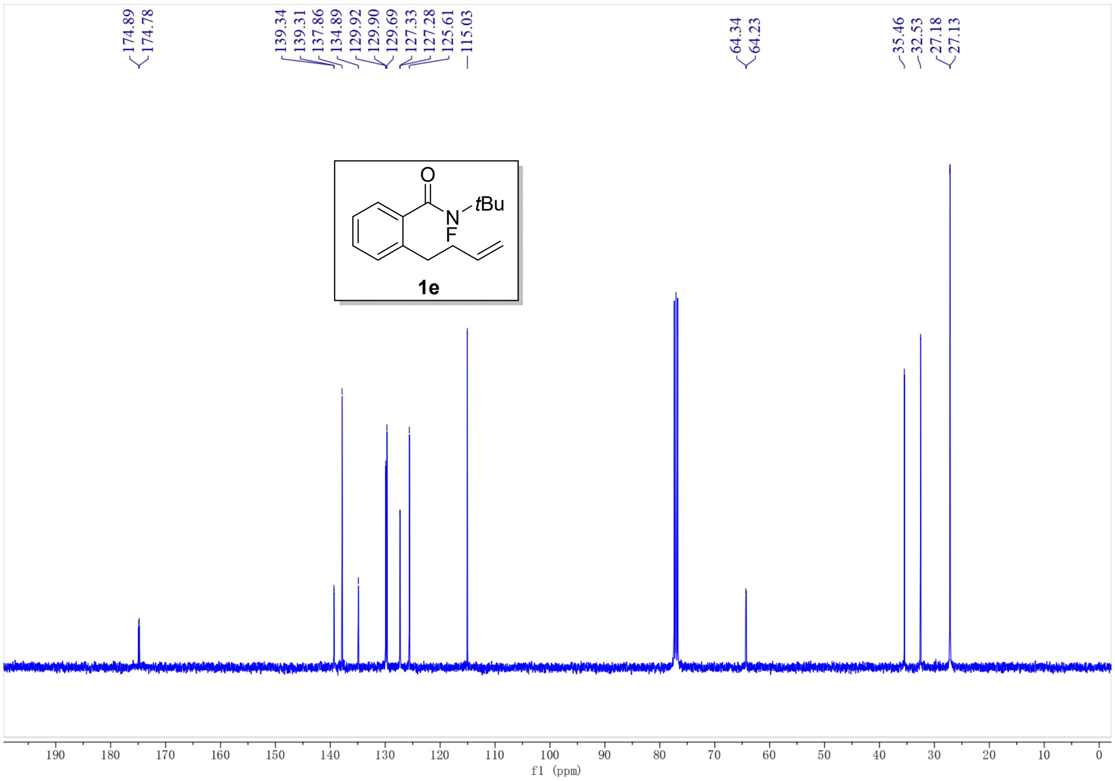

Supplementary Figure 26 ^{13}C NMR of **1e**

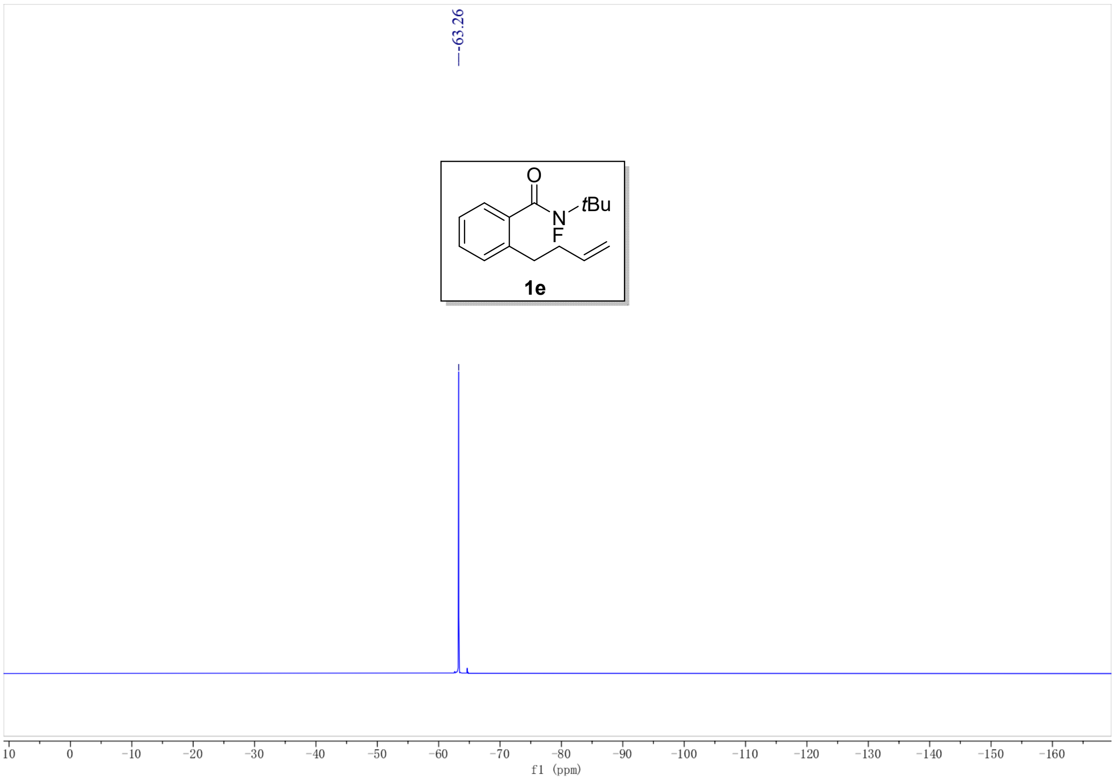

Supplementary Figure 27 ^{19}F NMR of **1e**

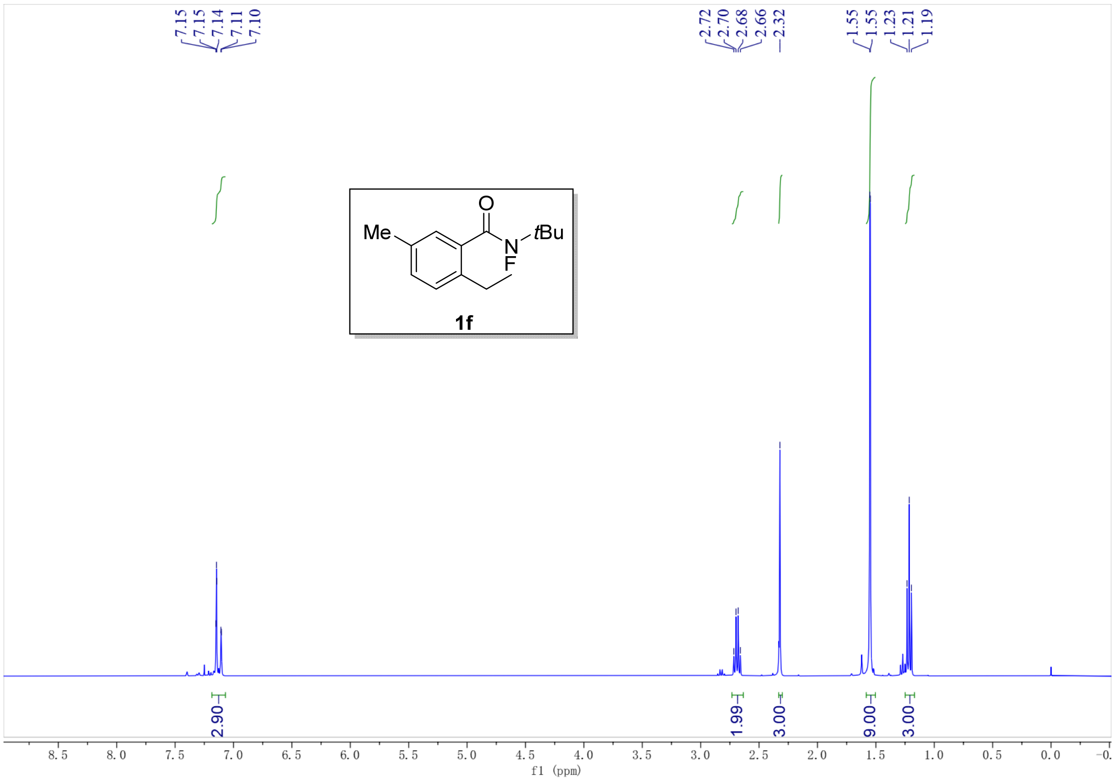

Supplementary Figure 28 ^{1}H NMR of **1f**

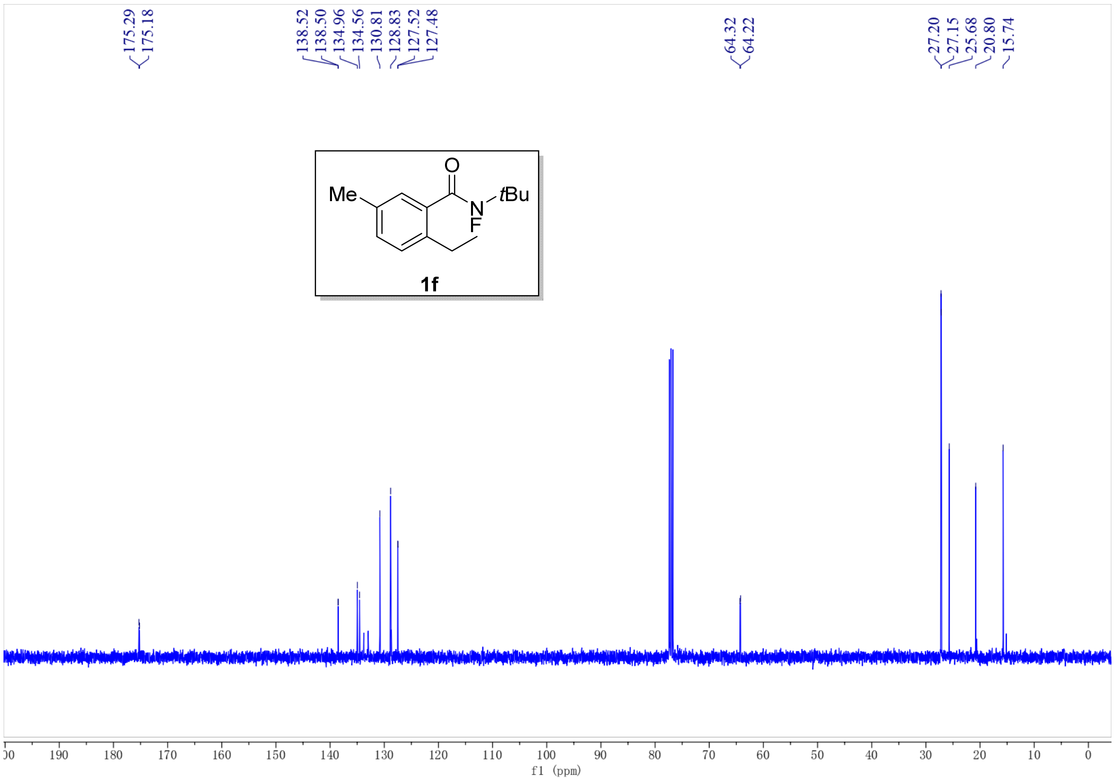

Supplementary Figure 29 ^{13}C NMR of **1f**

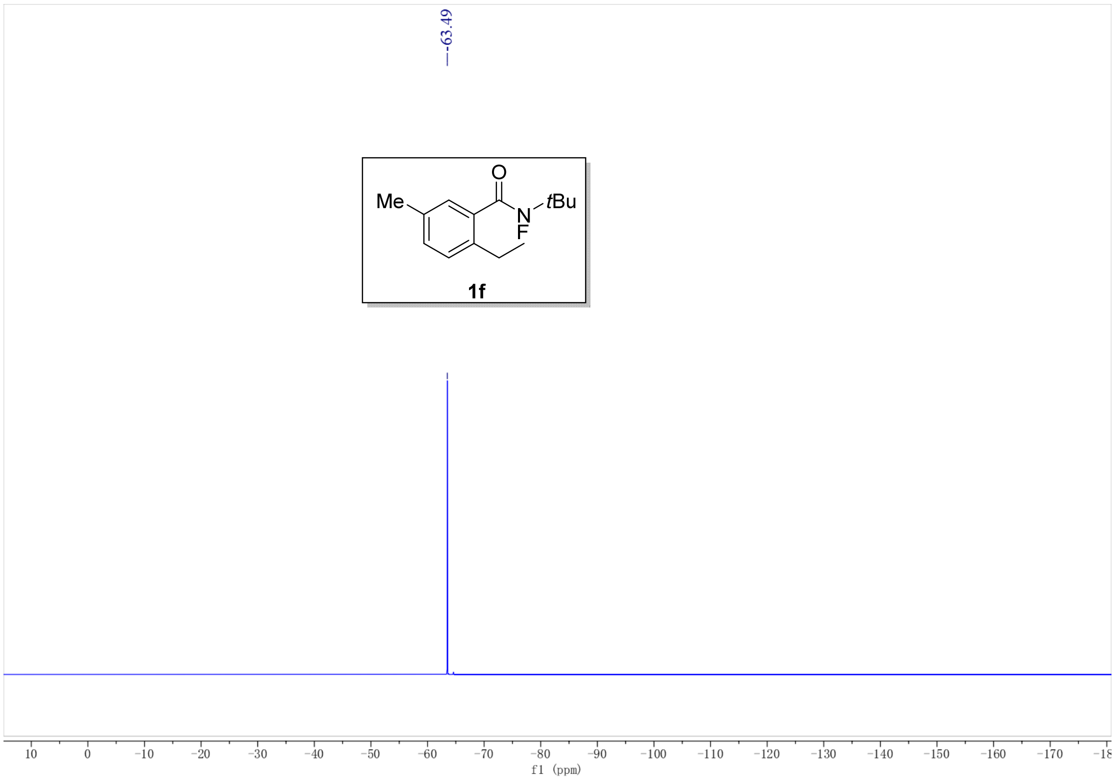

Supplementary Figure 30 ^{19}F NMR of **1f**

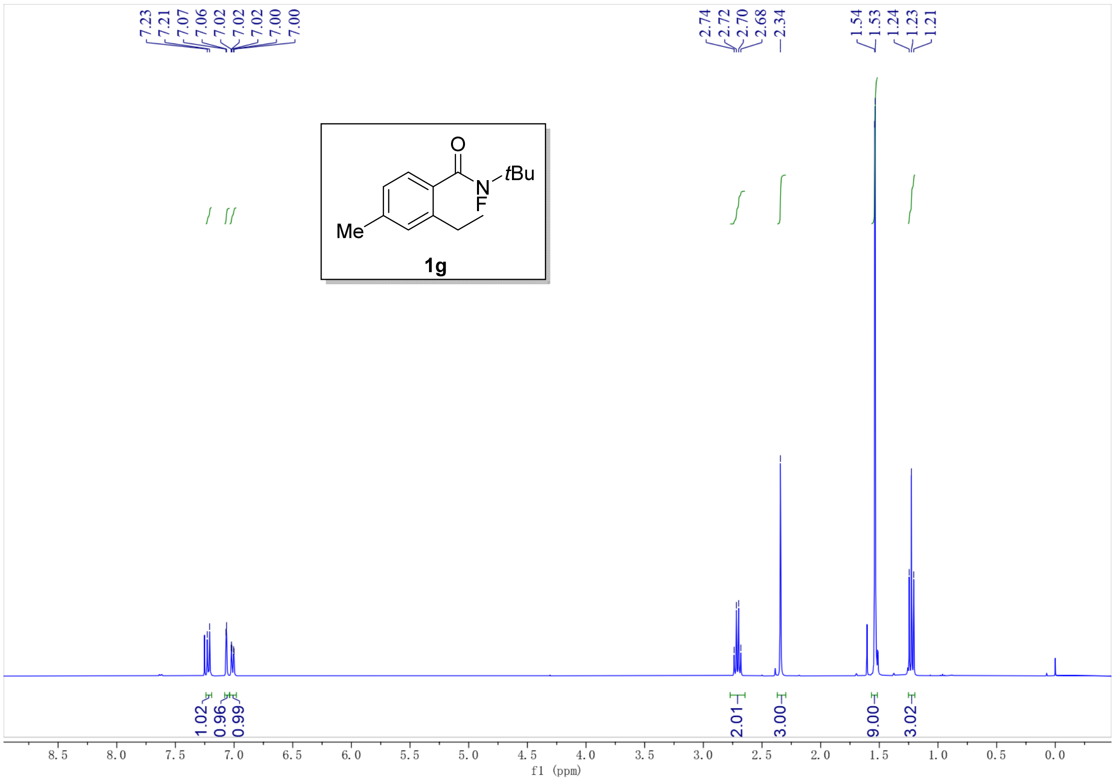

Supplementary Figure 31 ^{1}H NMR of **1g**

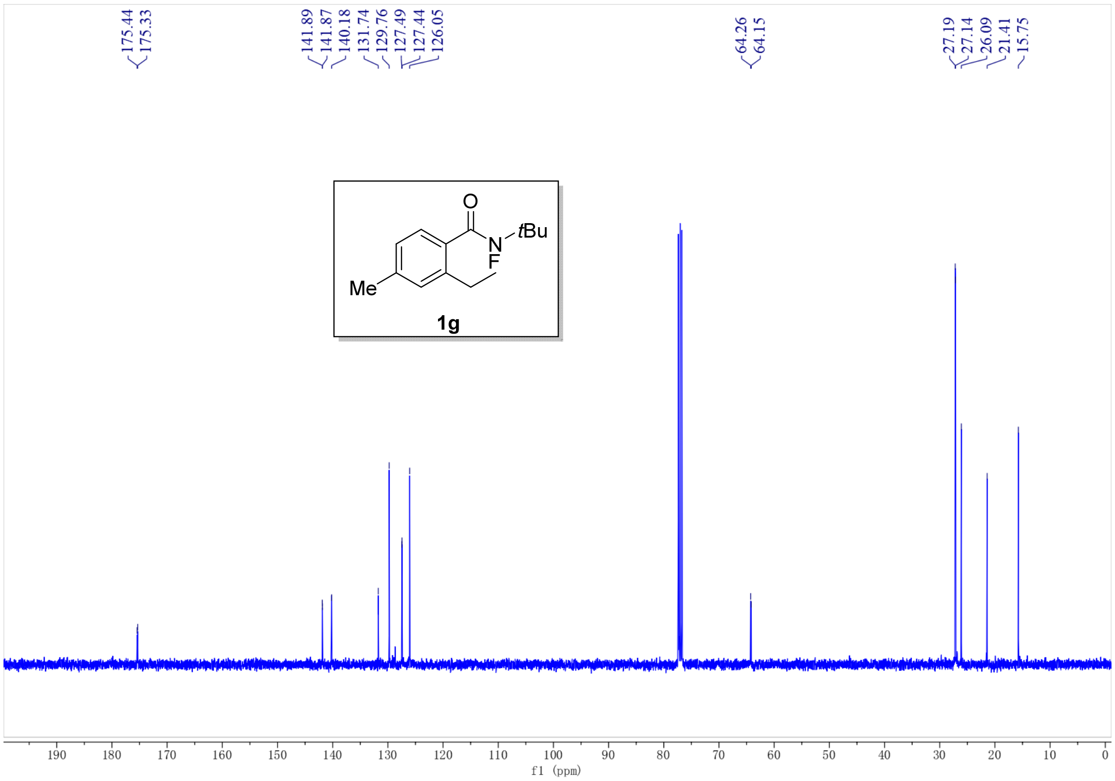

Supplementary Figure 32 ^{13}C NMR of **1g**

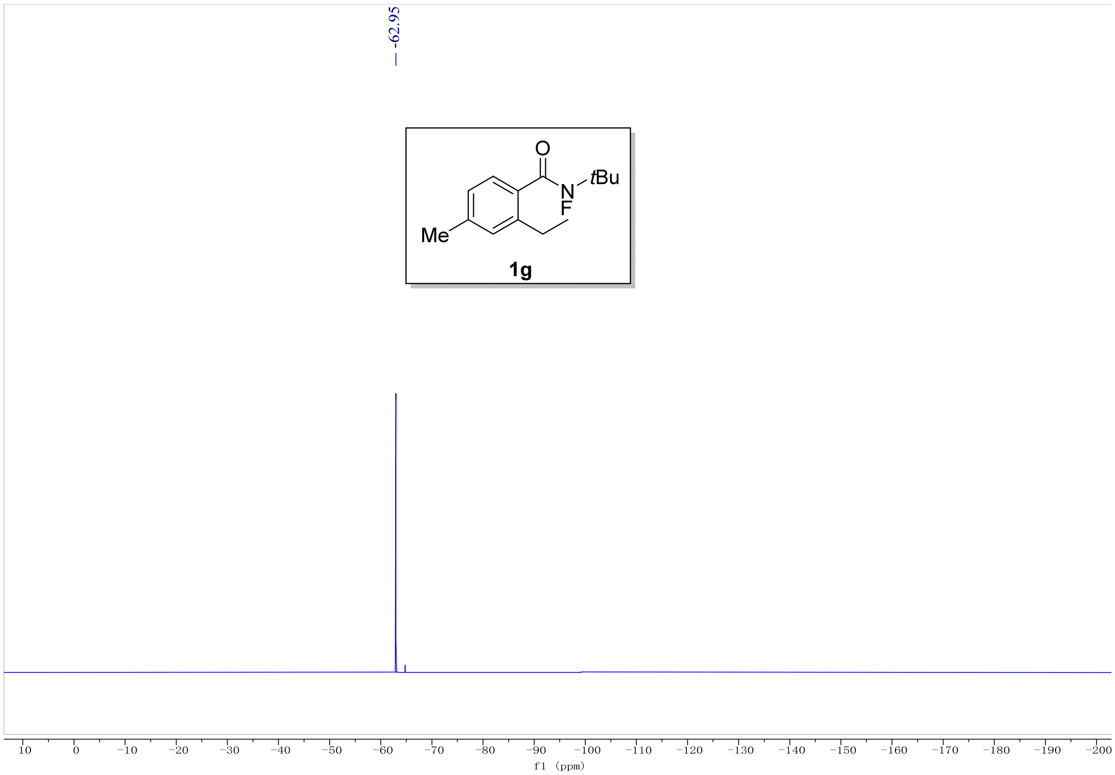

Supplementary Figure 33 ^{19}F NMR of **1g**

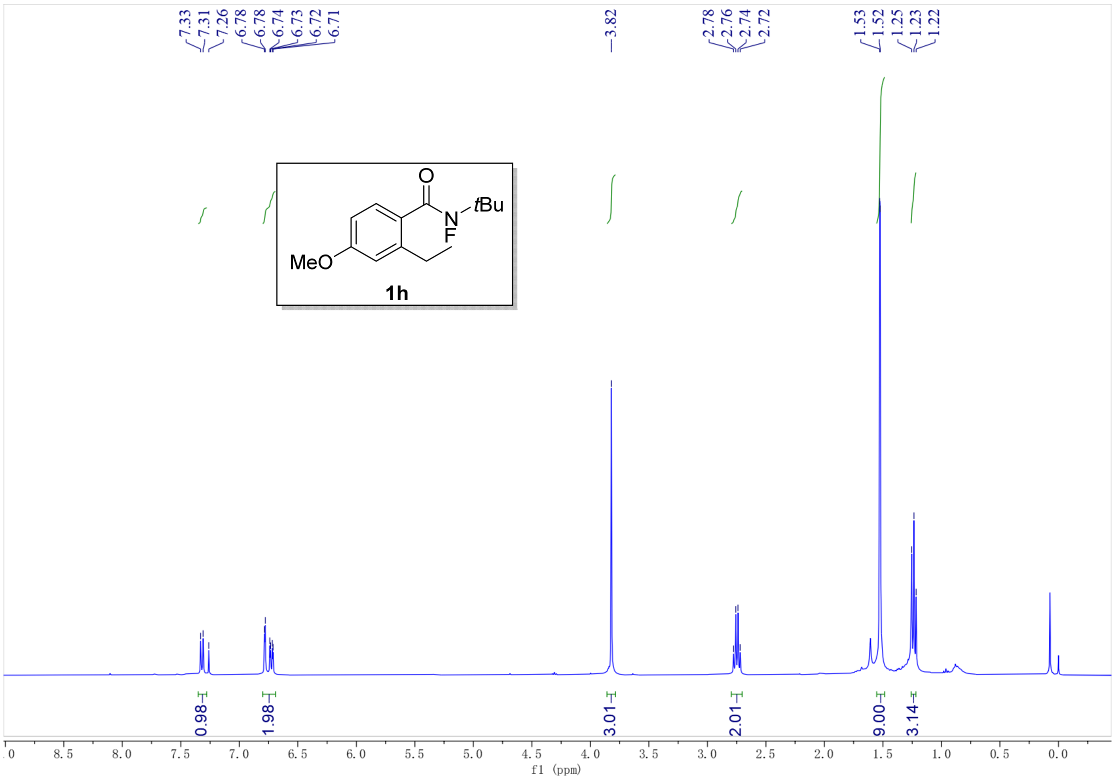

Supplementary Figure 34 ^{1}H NMR of **1h**

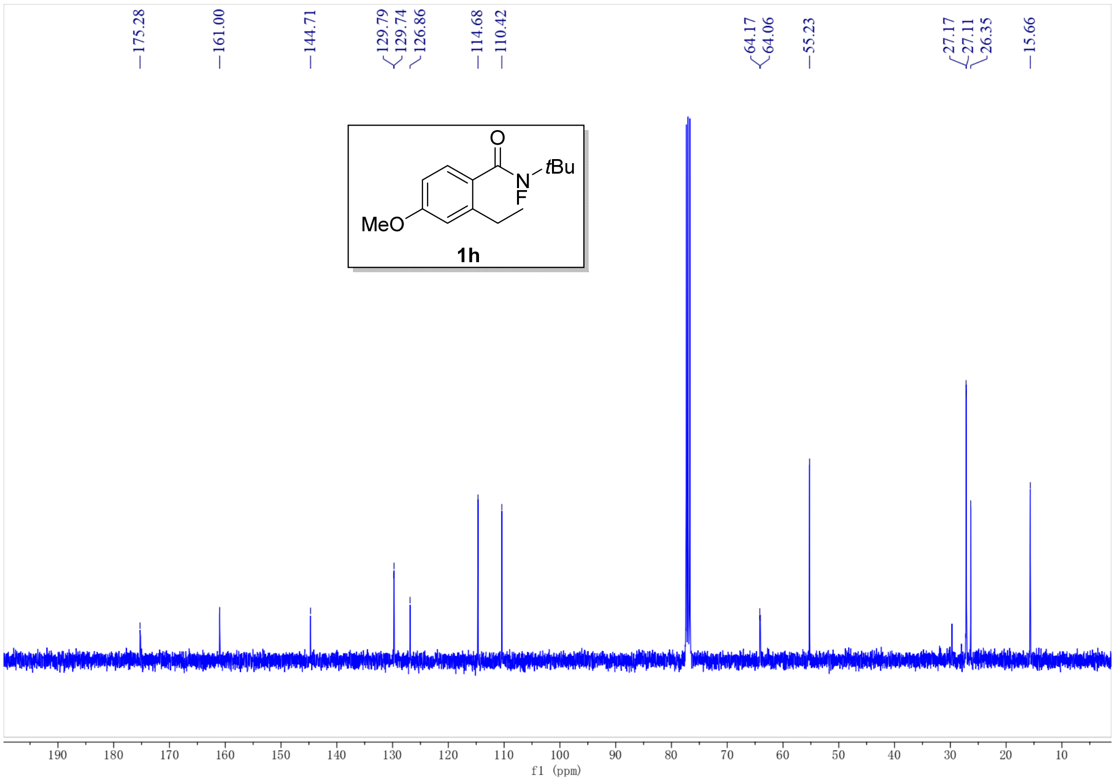

Supplementary Figure 35 ^{13}C NMR of **1h**

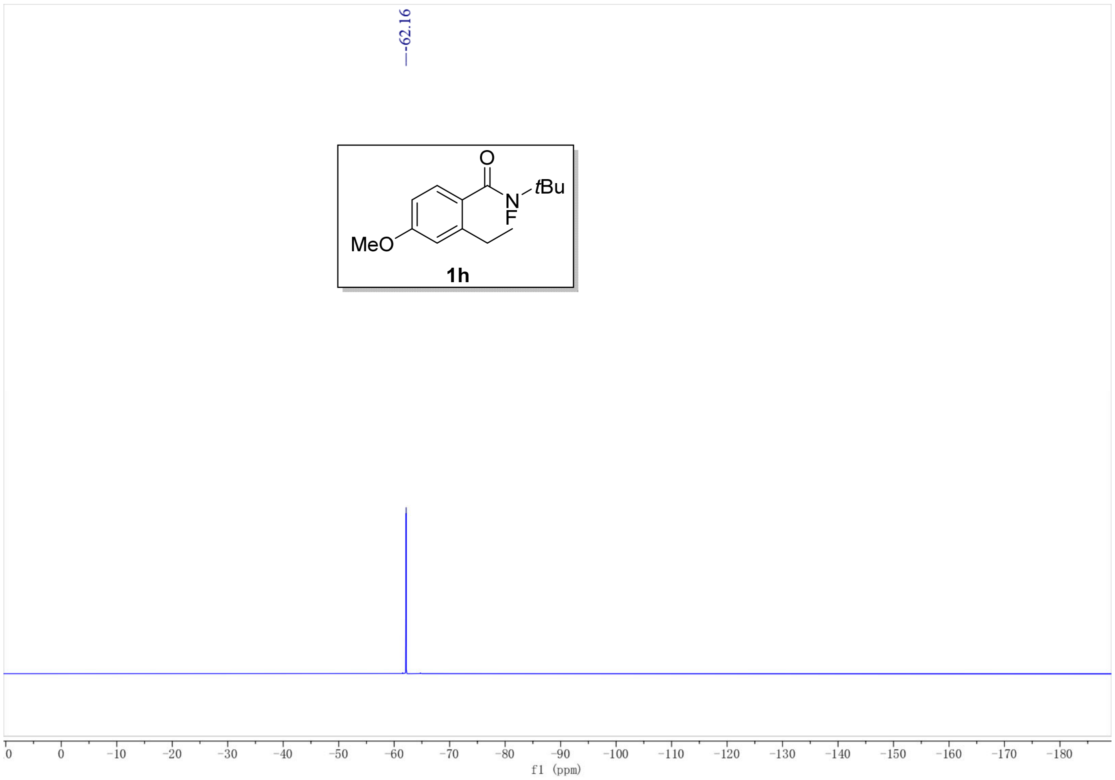

Supplementary Figure 36 ^{19}F NMR of **1h**

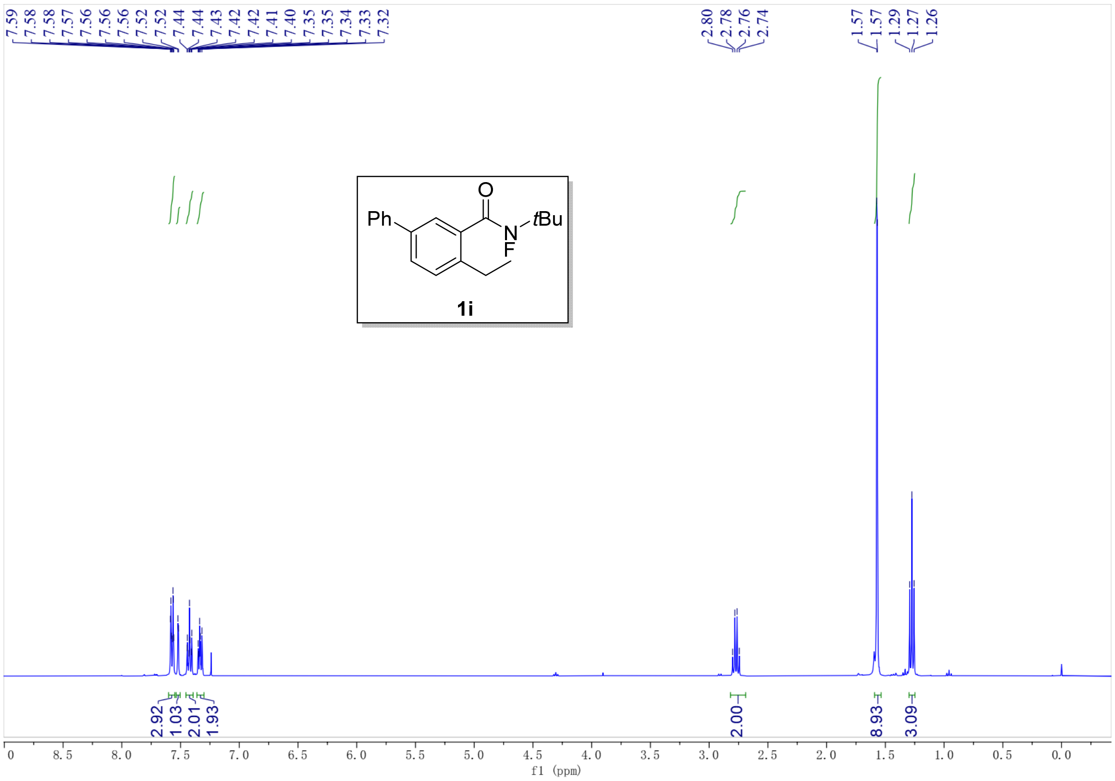

Supplementary Figure 37 ^{1}H NMR of **1i**

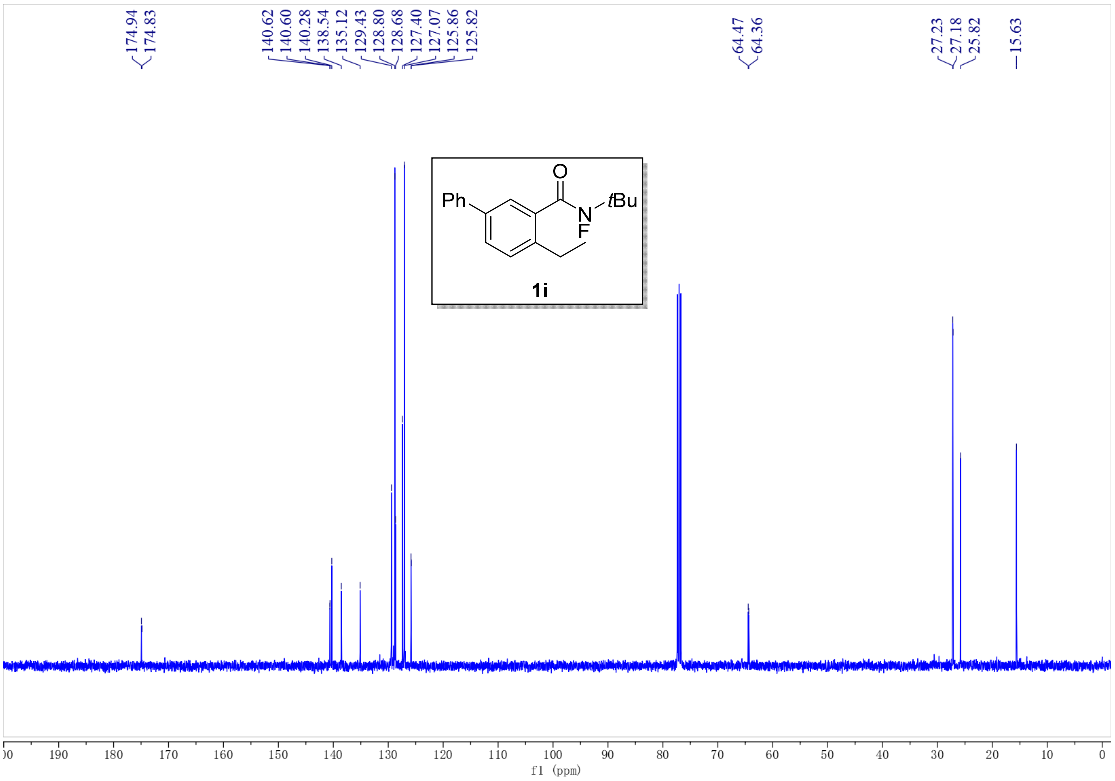

Supplementary Figure 38 ^{13}C NMR of **1i**

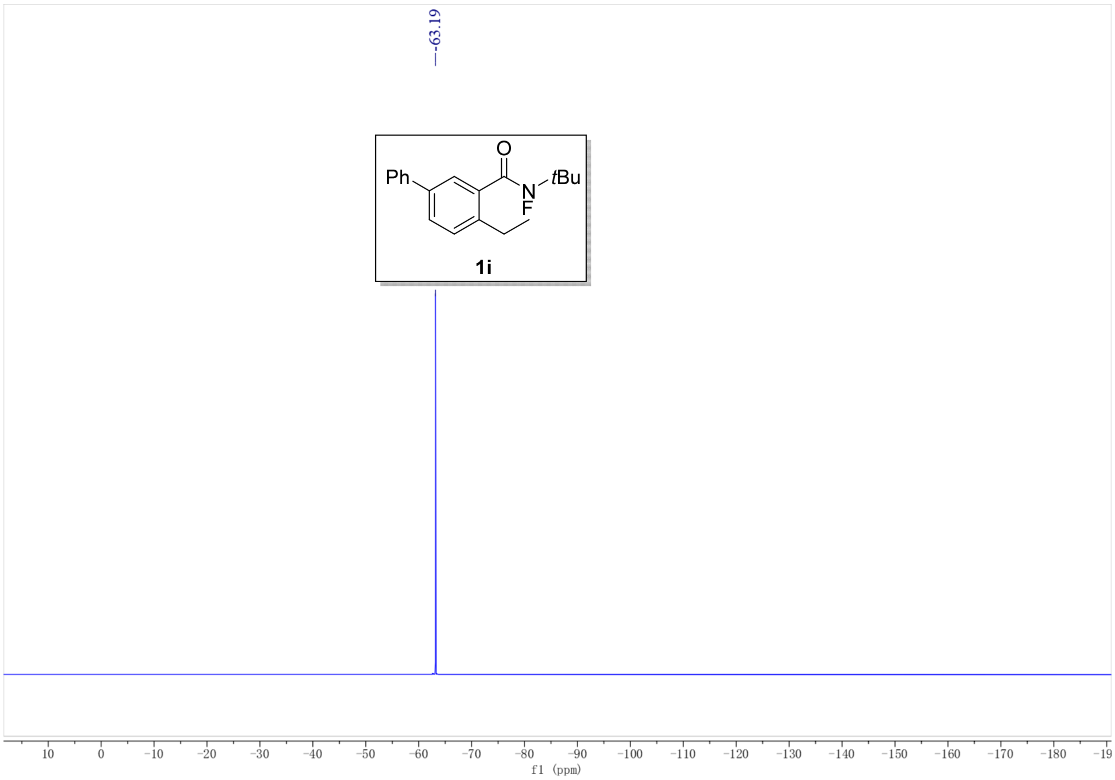

Supplementary Figure 39 ^{19}F NMR of **1i**

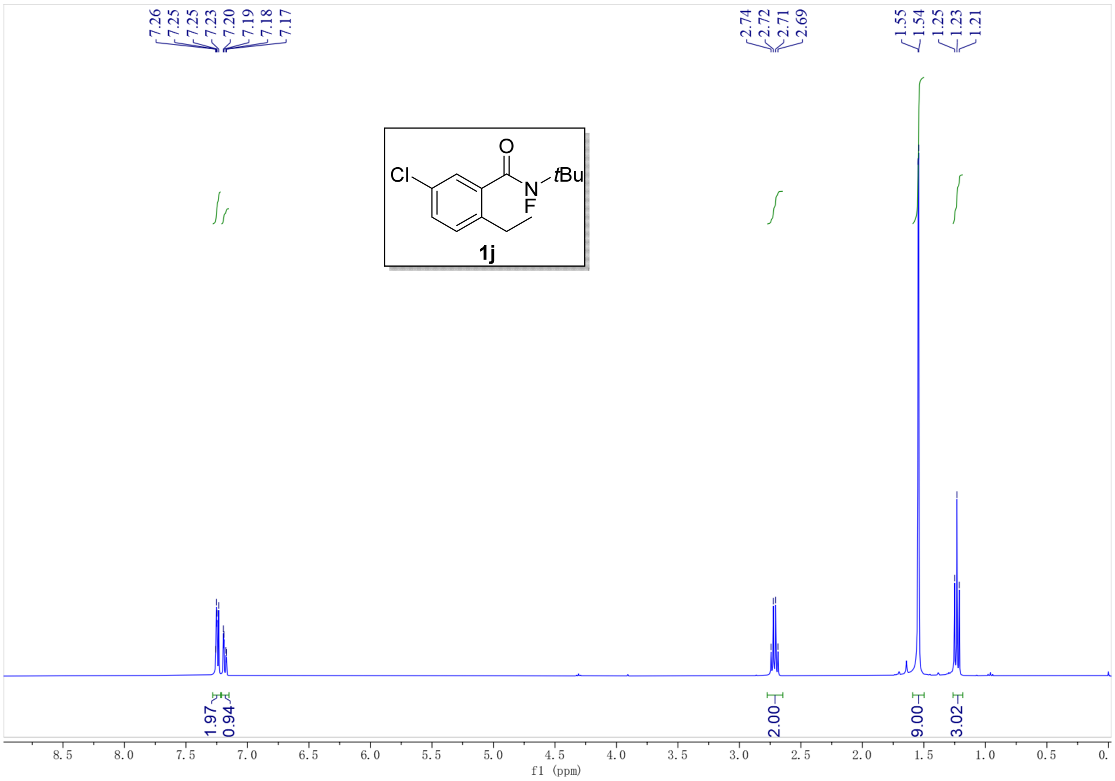

Supplementary Figure 40 ^{1}H NMR of **1j**

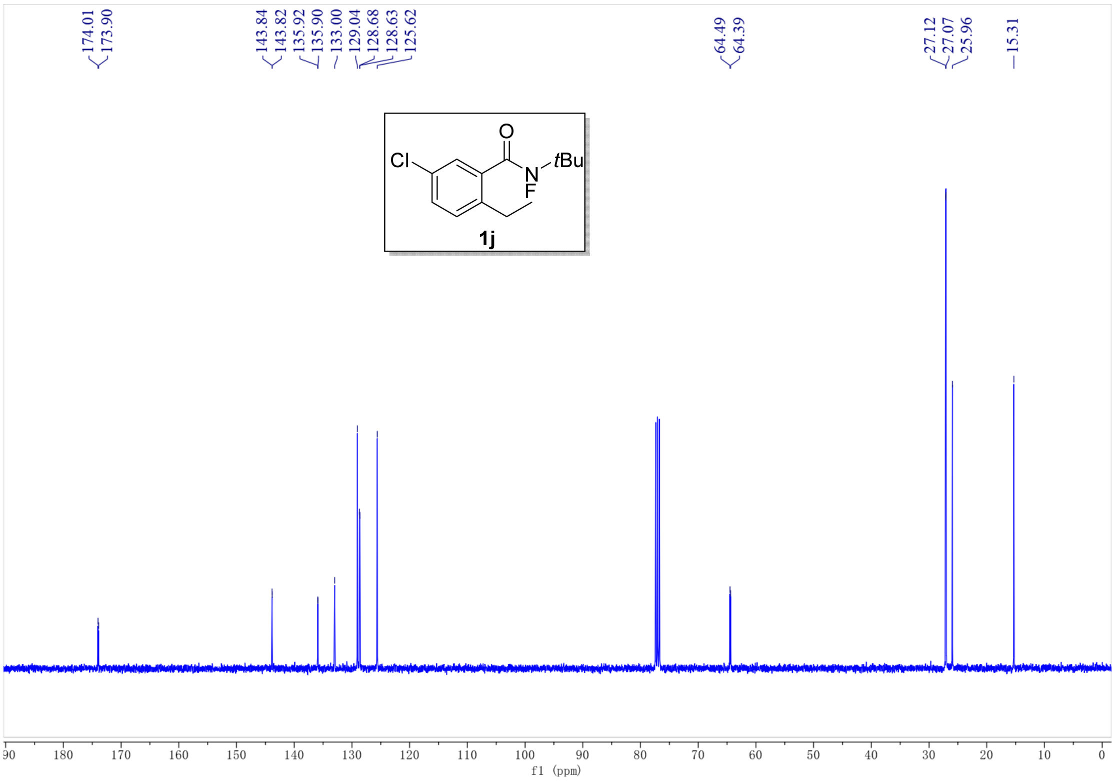

Supplementary Figure 41 ^{13}C NMR of **1j**

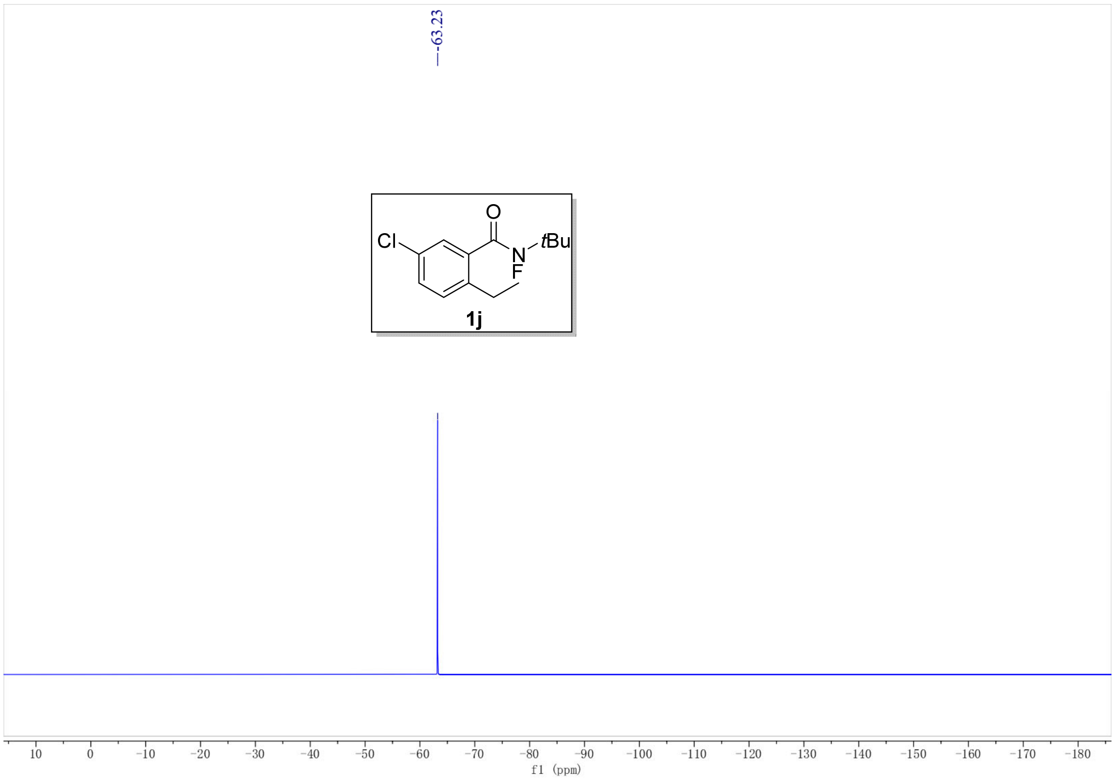

Supplementary Figure 42 ^{19}F NMR of **1j**

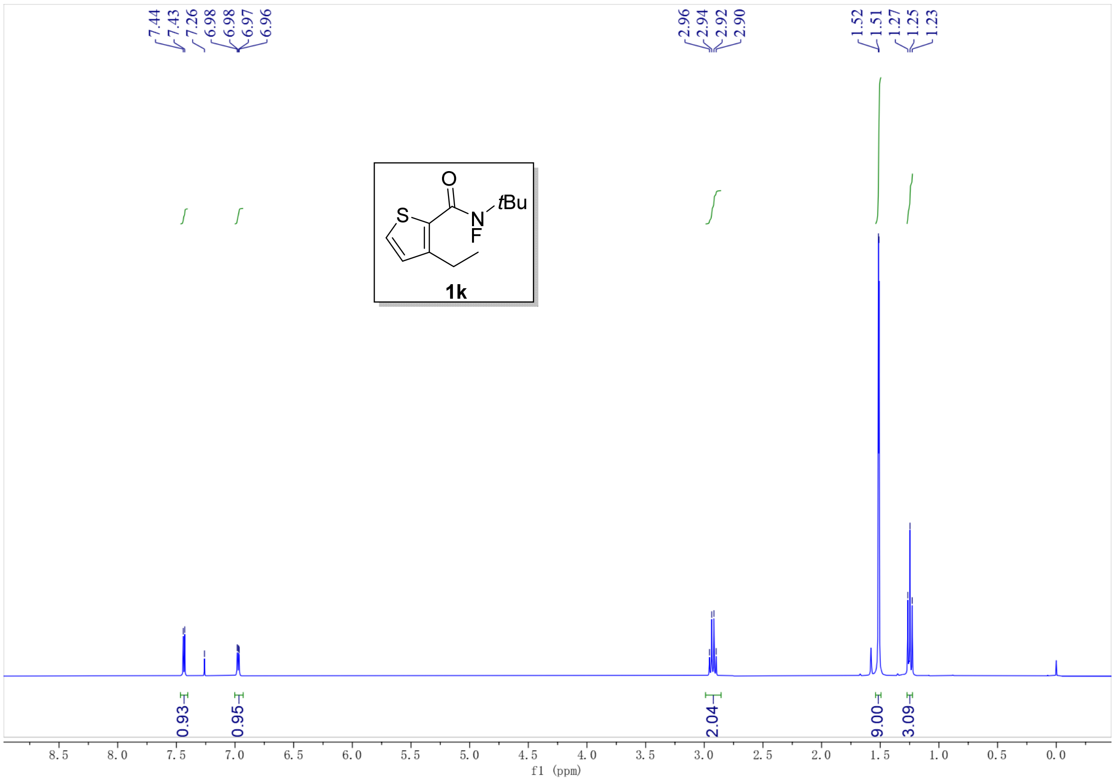

Supplementary Figure 43 ^{1}H NMR of **1k**

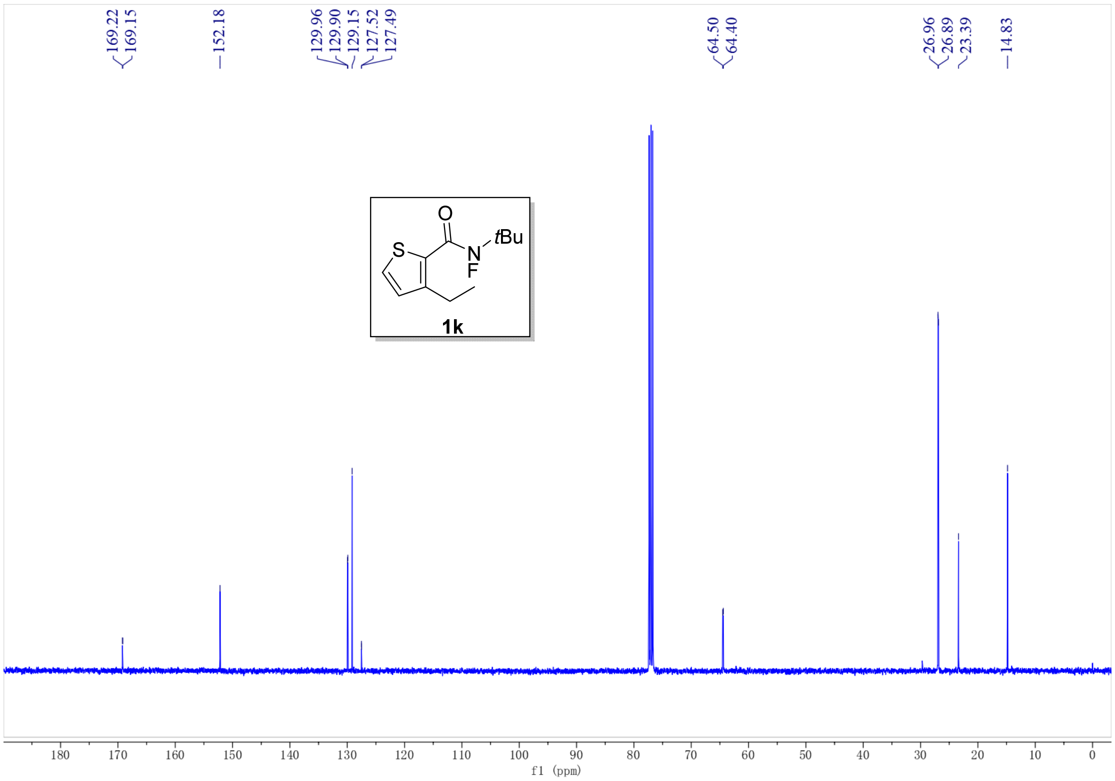

Supplementary Figure 44 ^{13}C NMR of **1k**

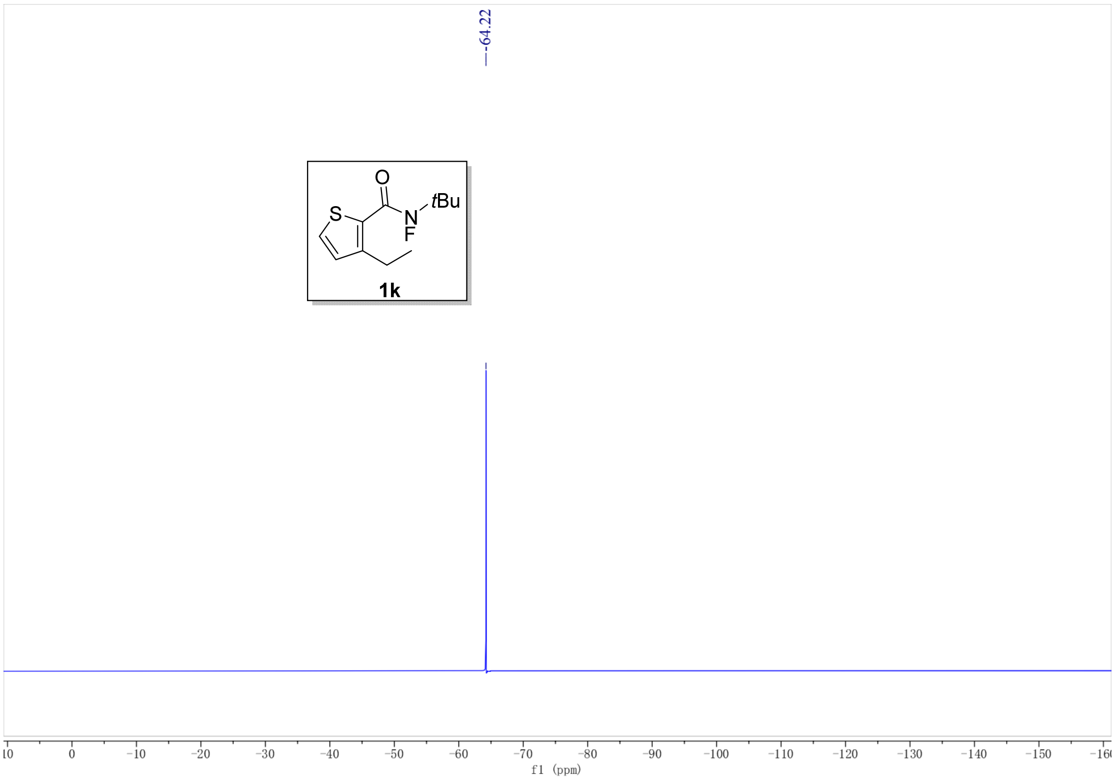

Supplementary Figure 45 ^{19}F NMR of **1k**

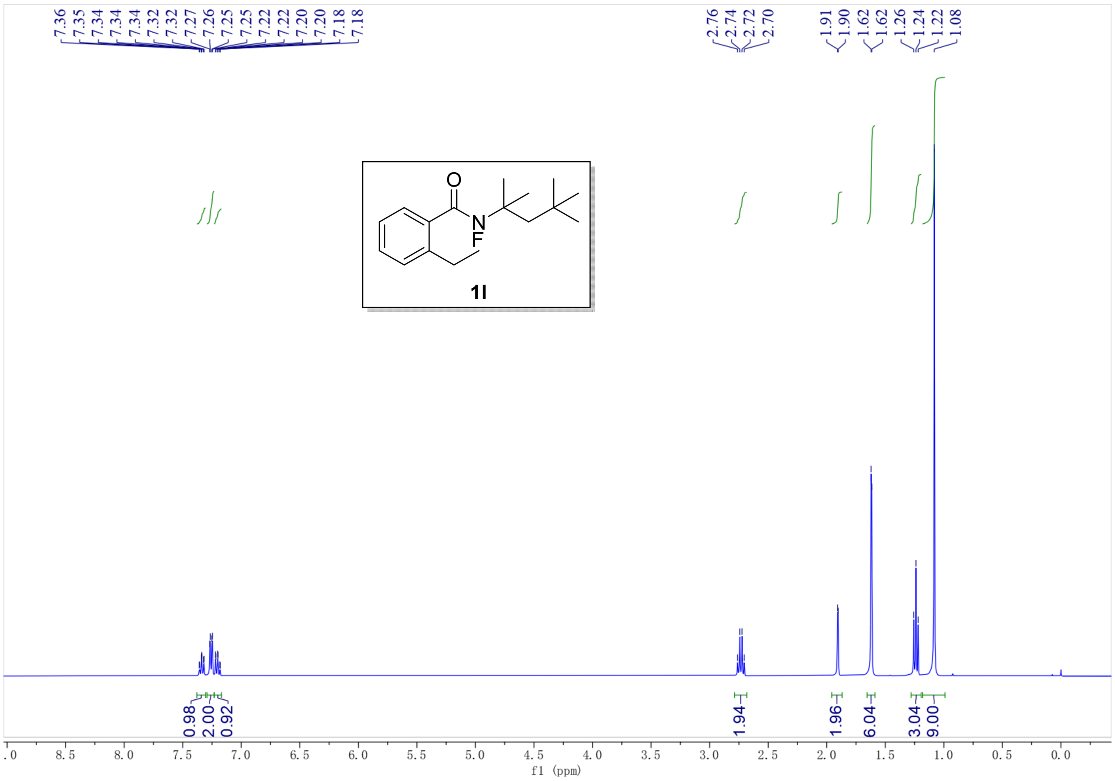

Supplementary Figure 46 ^{1}H NMR of **1l**

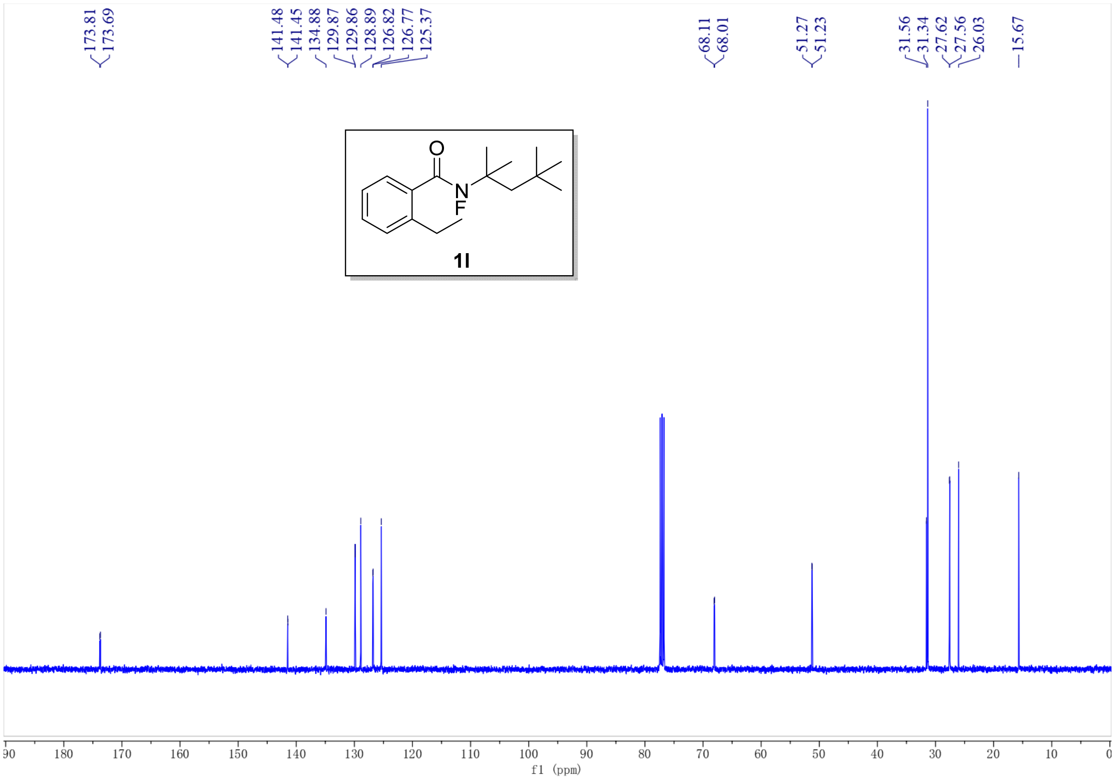

Supplementary Figure 47 ^{13}C NMR of **1l**

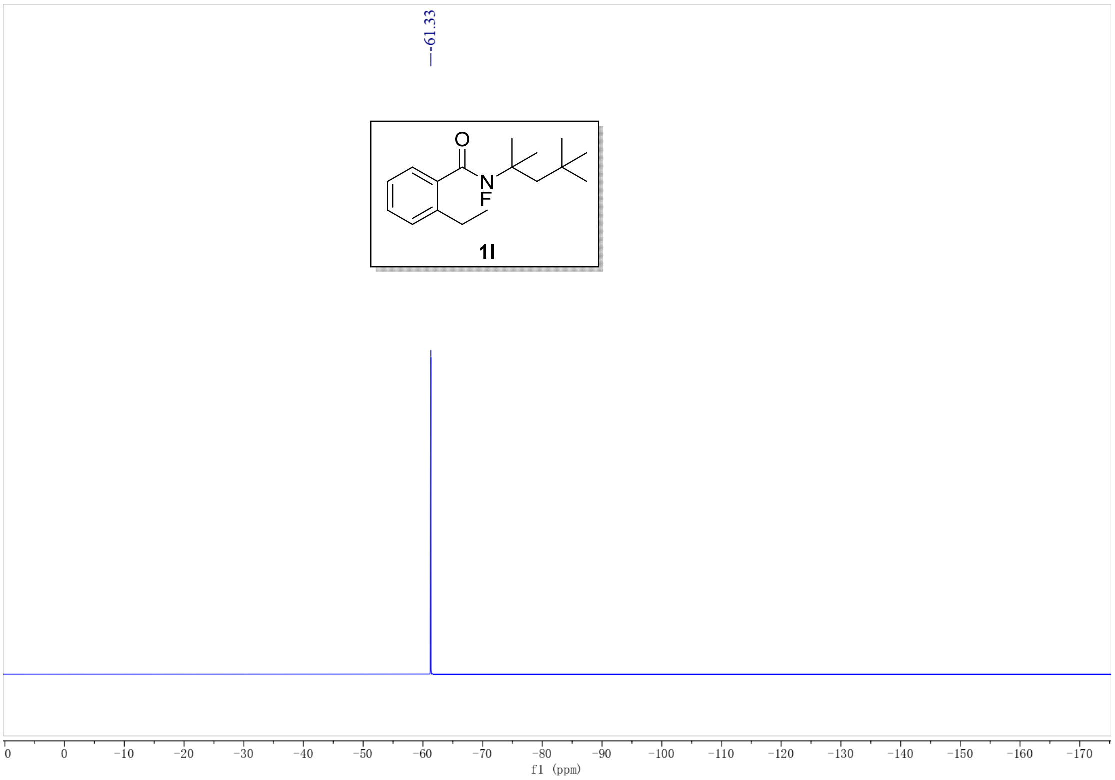

Supplementary Figure 48 ^{19}F NMR of **1l**

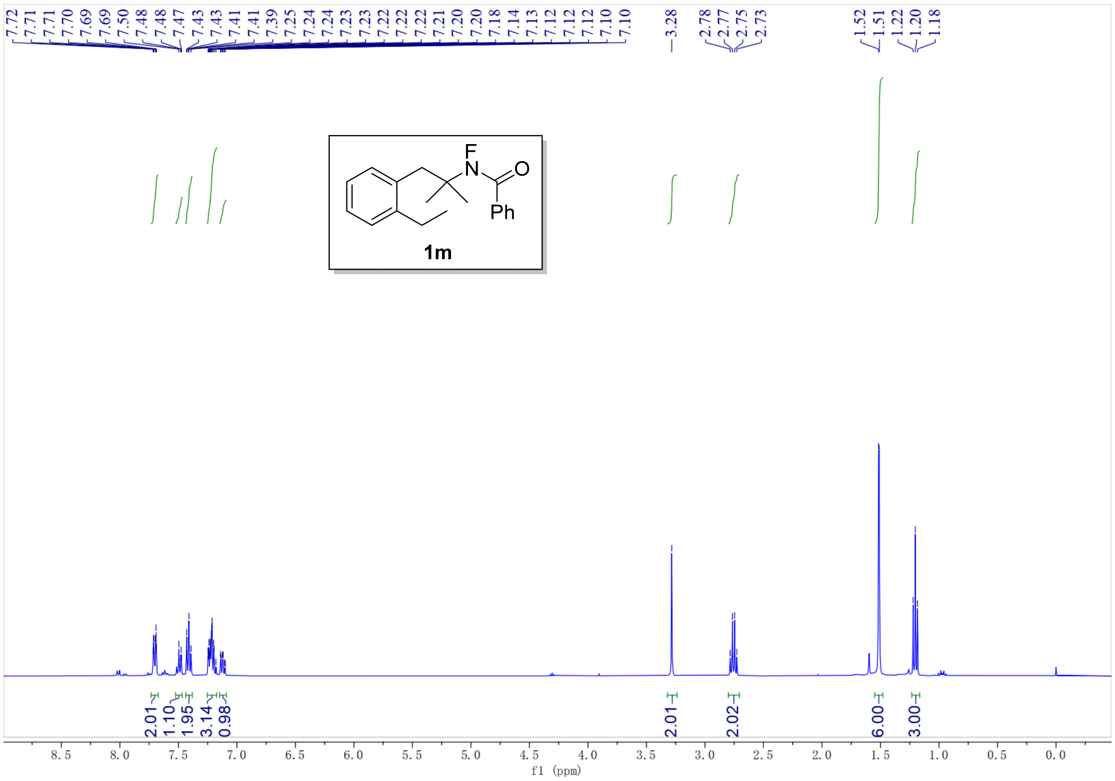

Supplementary Figure 49 ^{1}H NMR of **1m**

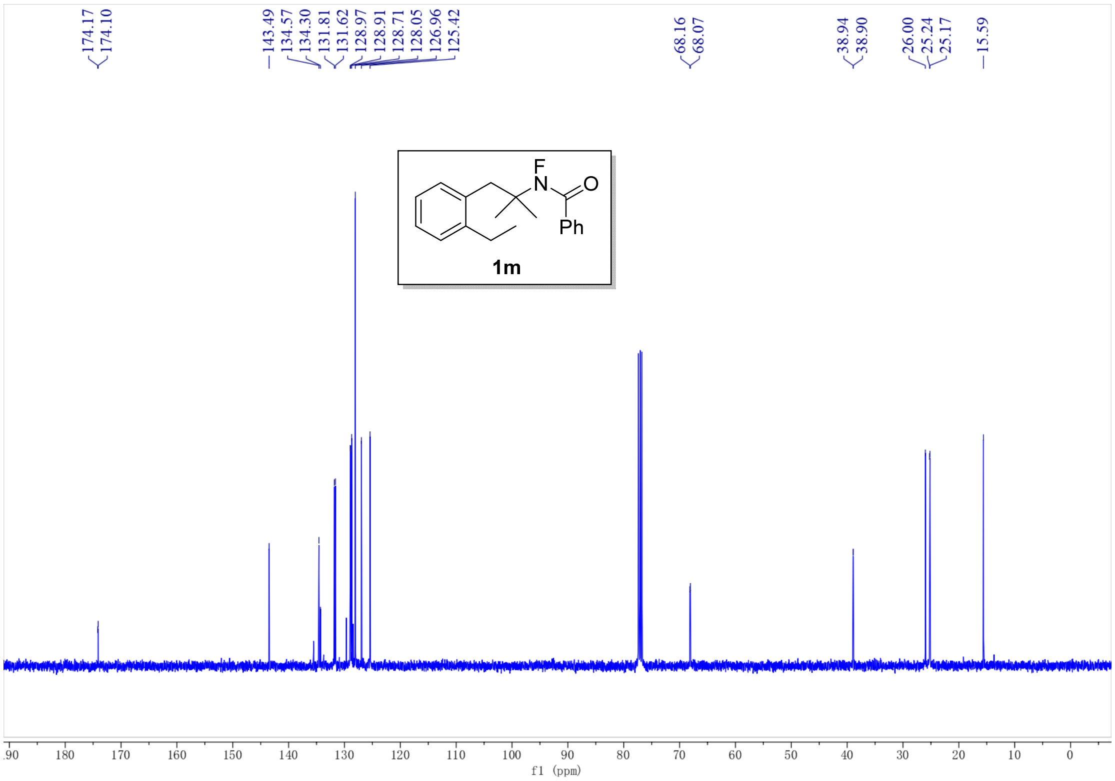

Supplementary Figure 50 ^{13}C NMR of **1m**

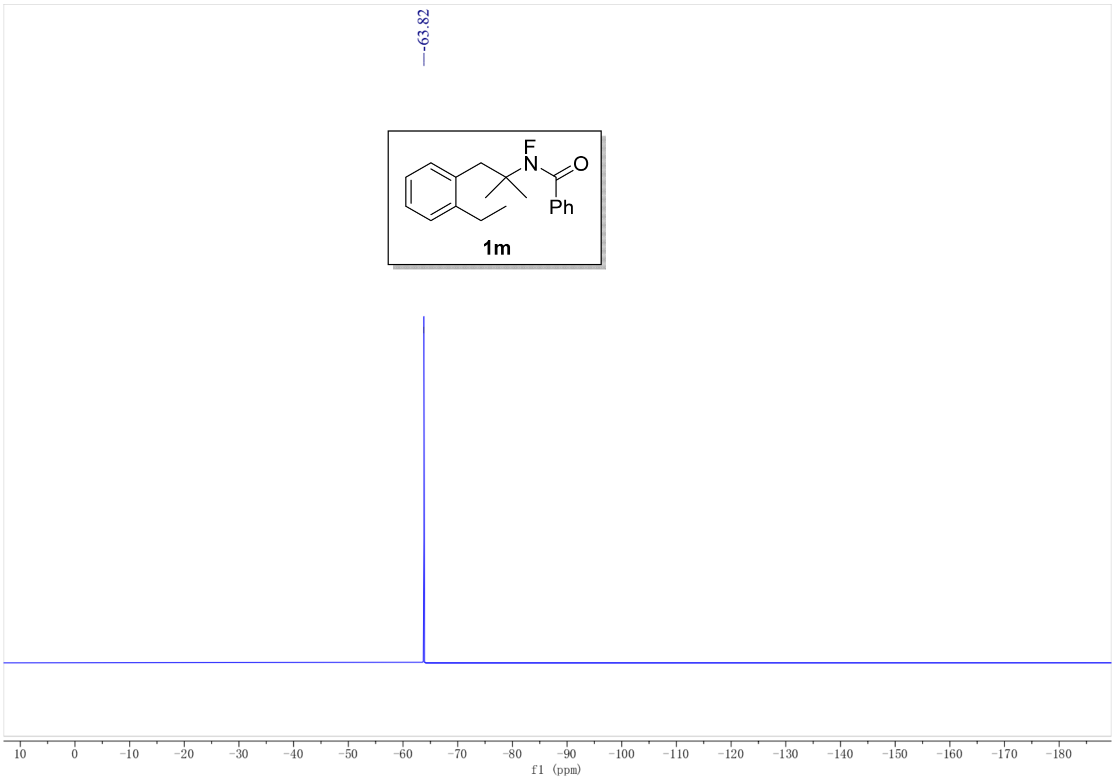

Supplementary Figure 51 ^{19}F NMR of **1m**

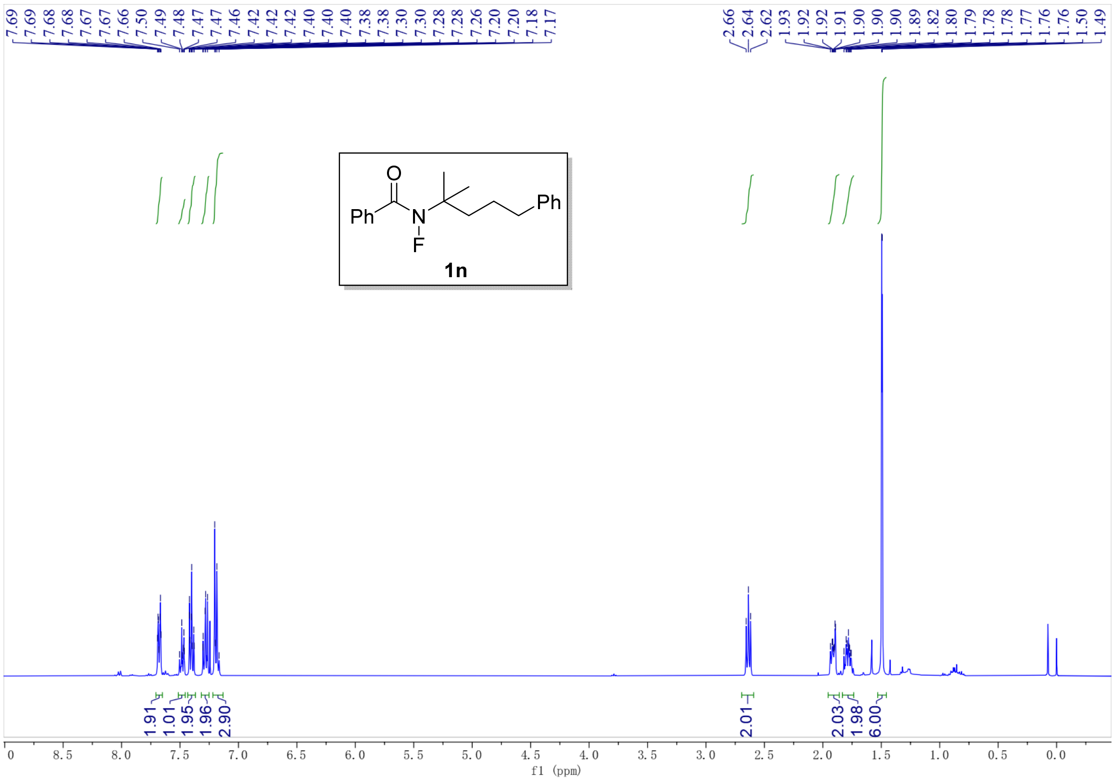

Supplementary Figure 52 ^{1}H NMR of **1n**

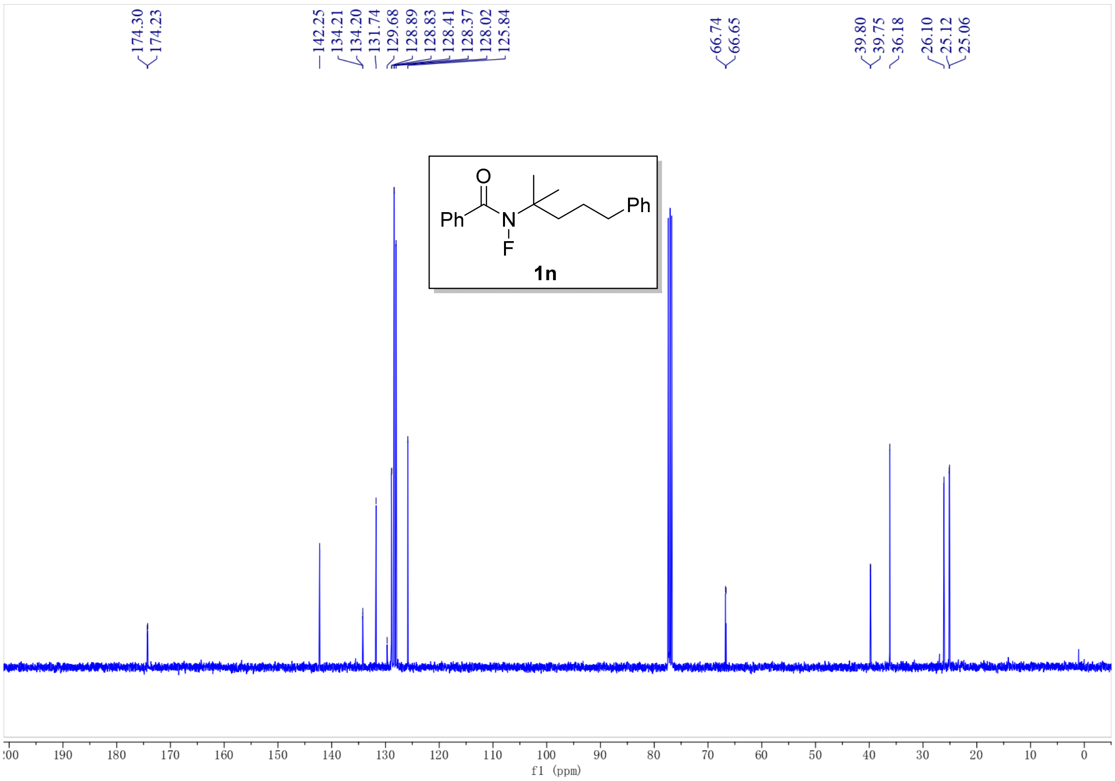

Supplementary Figure 53 ^{13}C NMR of **1n**

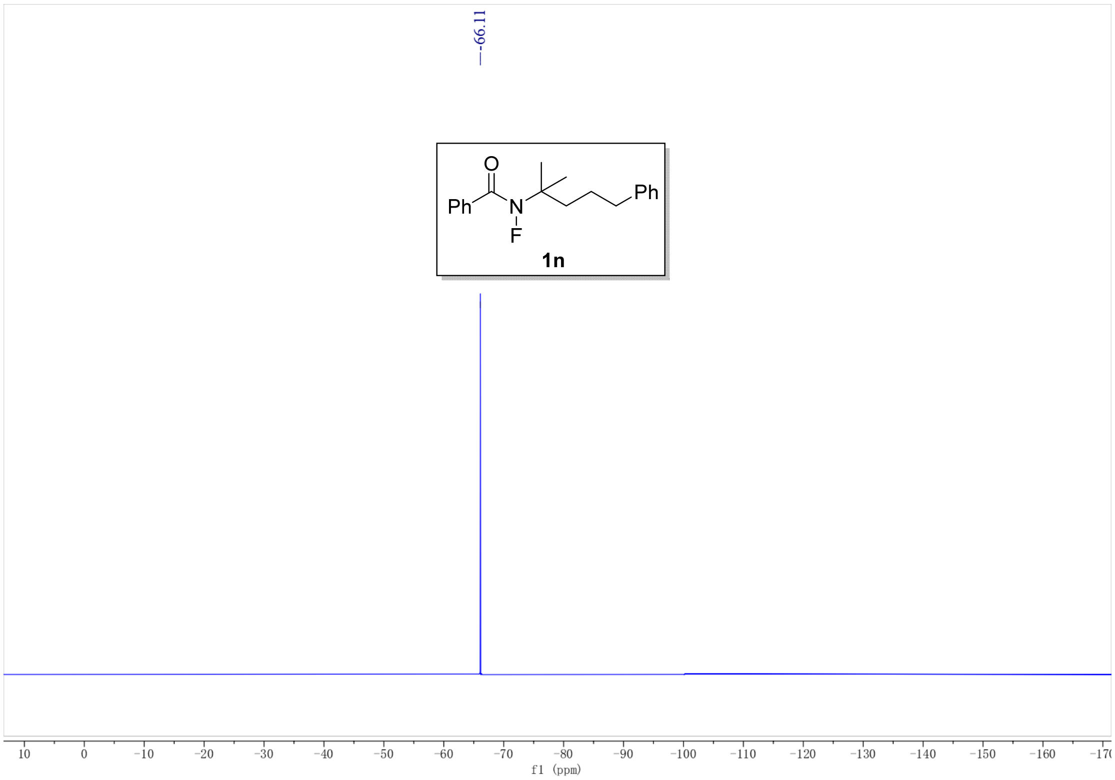

Supplementary Figure 54 ^{19}F NMR of **1n**

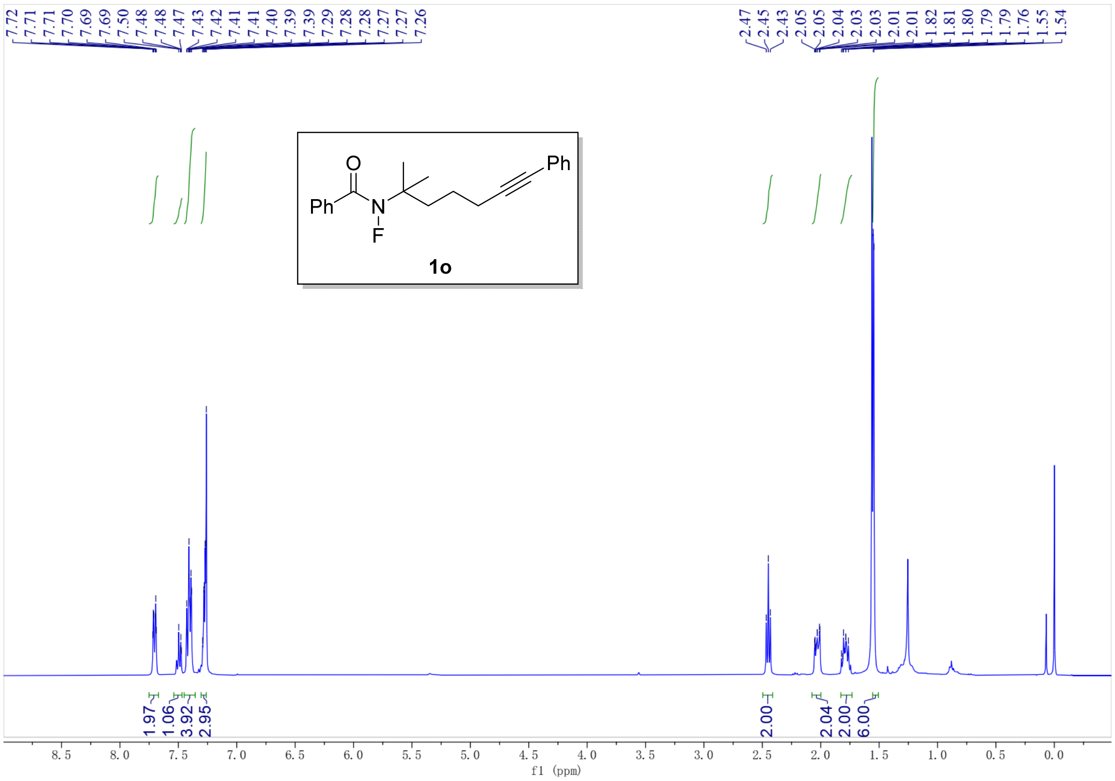

Supplementary Figure 55 ^{1}H NMR of **1o**

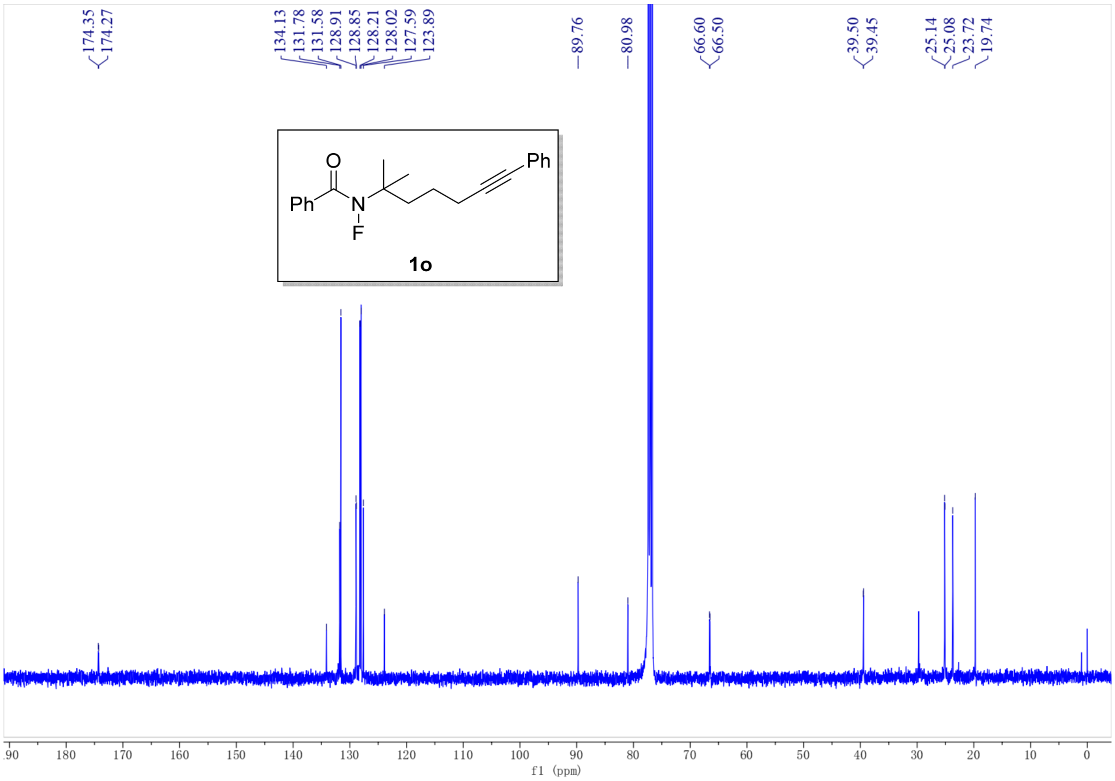

Supplementary Figure 56 ^{13}C NMR of **1o**

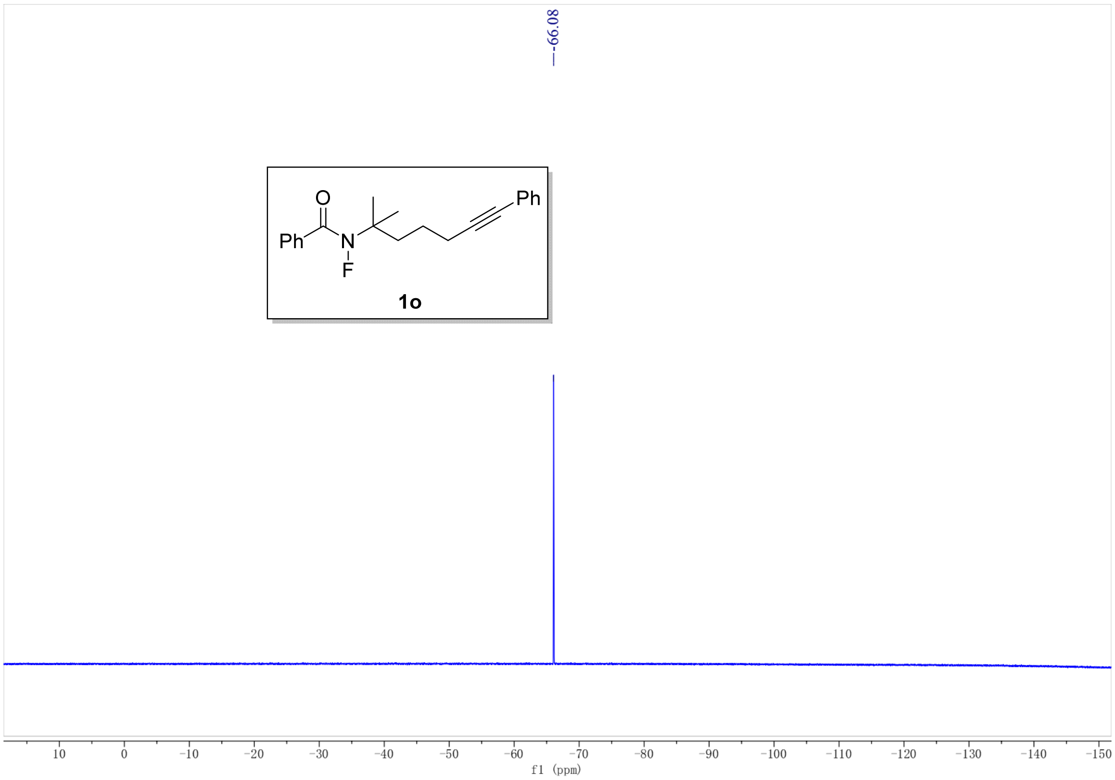

Supplementary Figure 57 ^{19}F NMR of **1o**

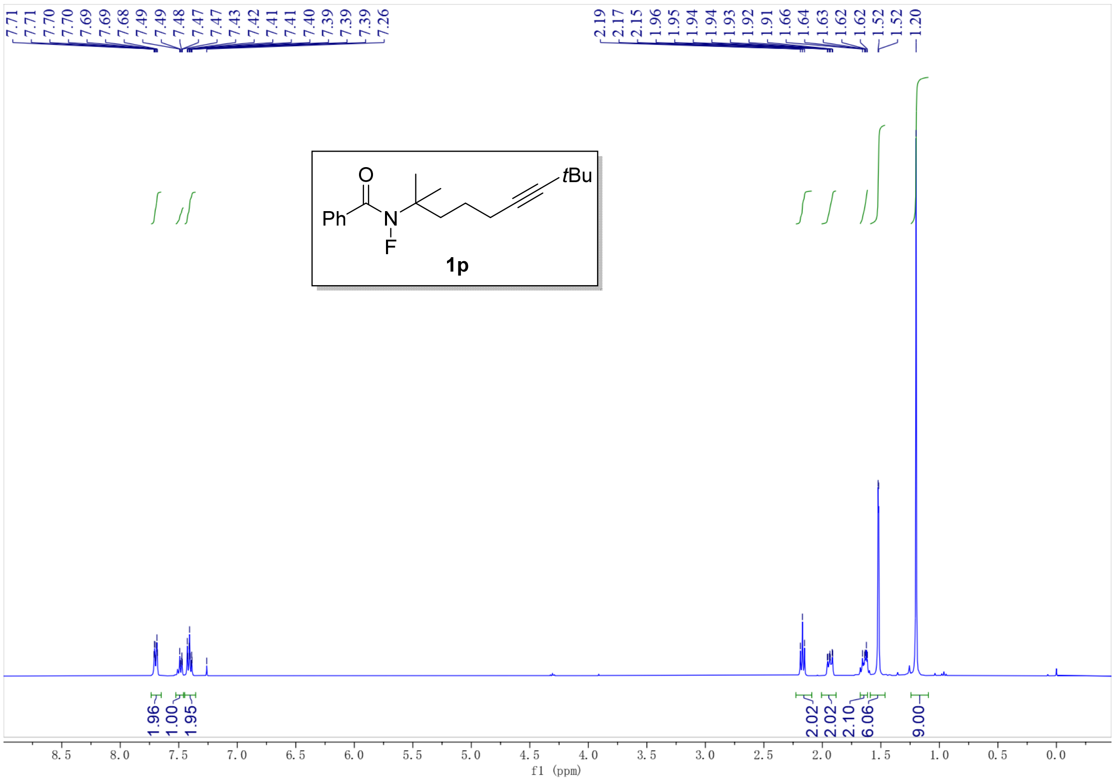

Supplementary Figure 58 ^{1}H NMR of **1p**

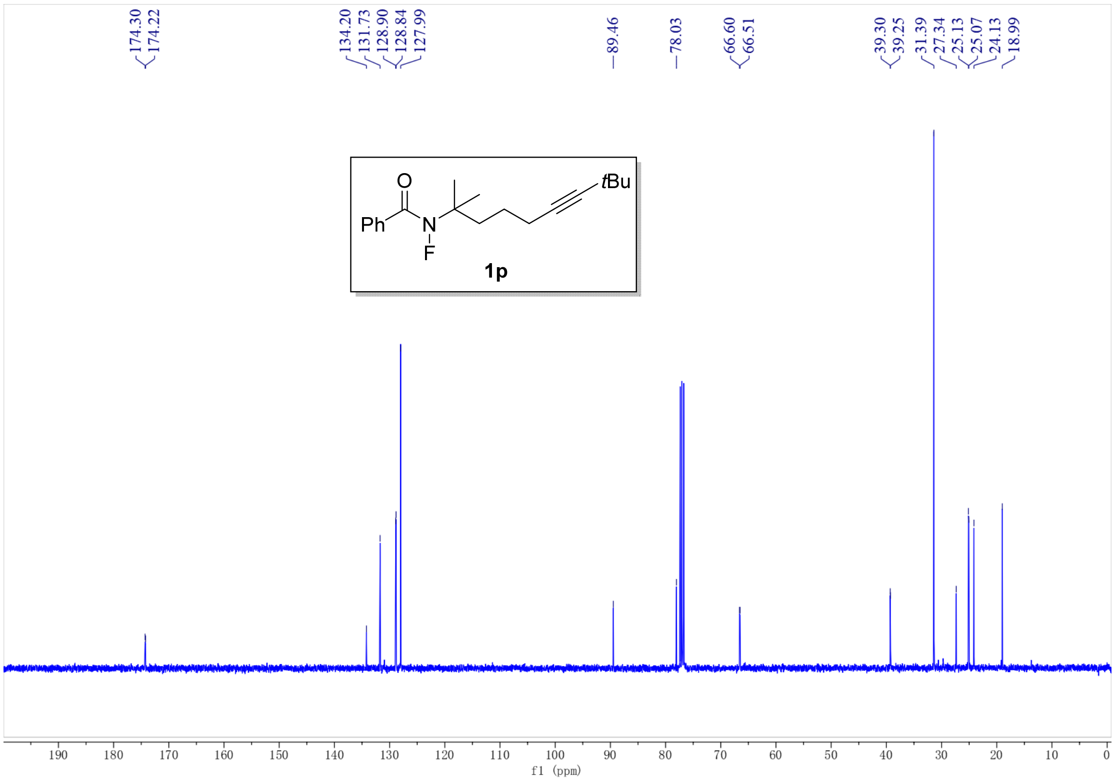

Supplementary Figure 59 ^{13}C NMR of **1p**

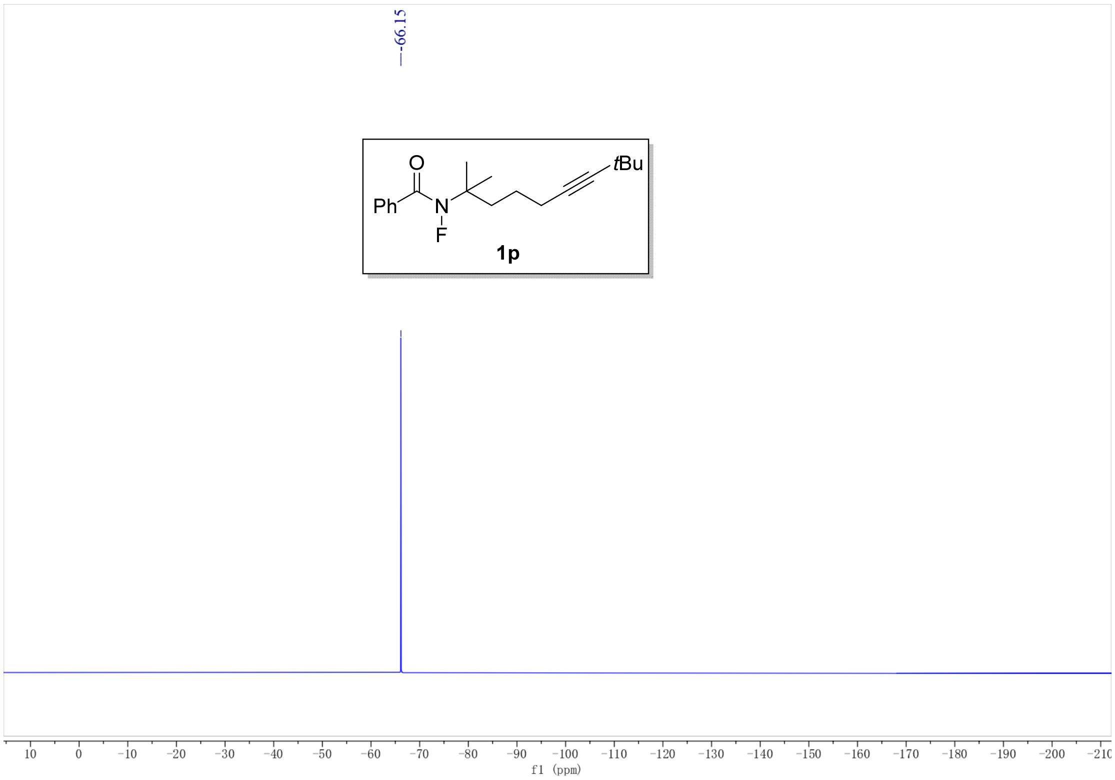

Supplementary Figure 60 ^{19}F NMR of **1p**

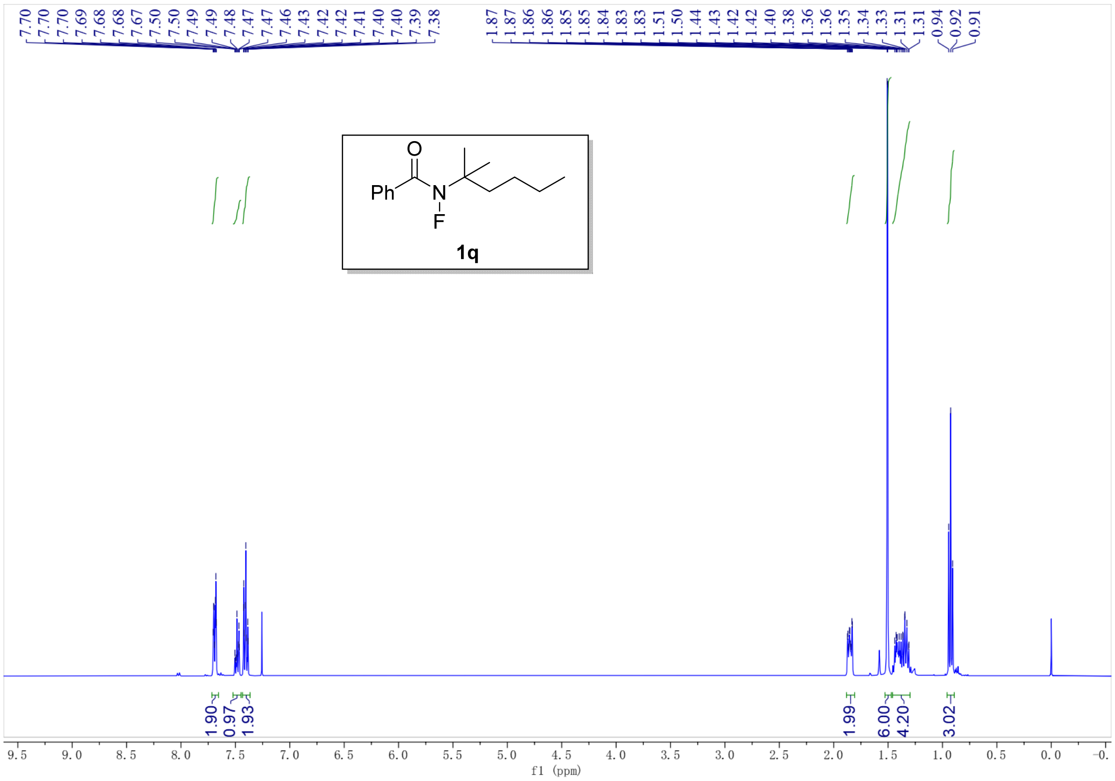

Supplementary Figure 61 ^{1}H NMR of **1q**

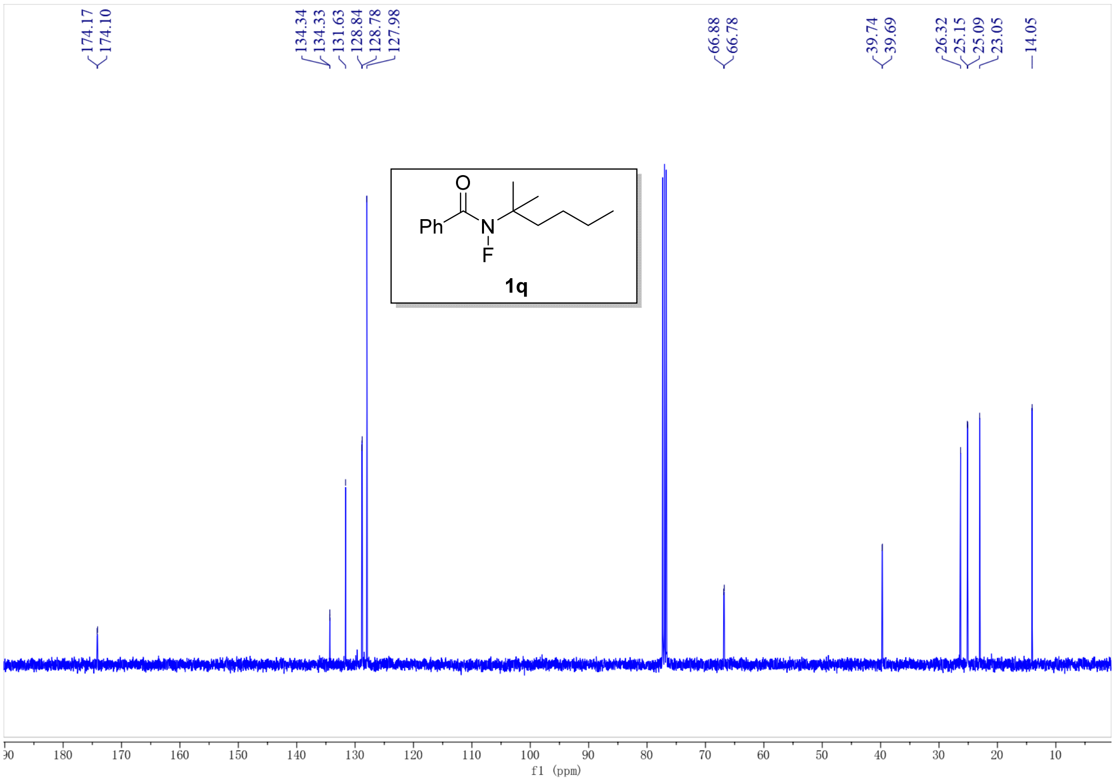

Supplementary Figure 62 ^{13}C NMR of **1q**

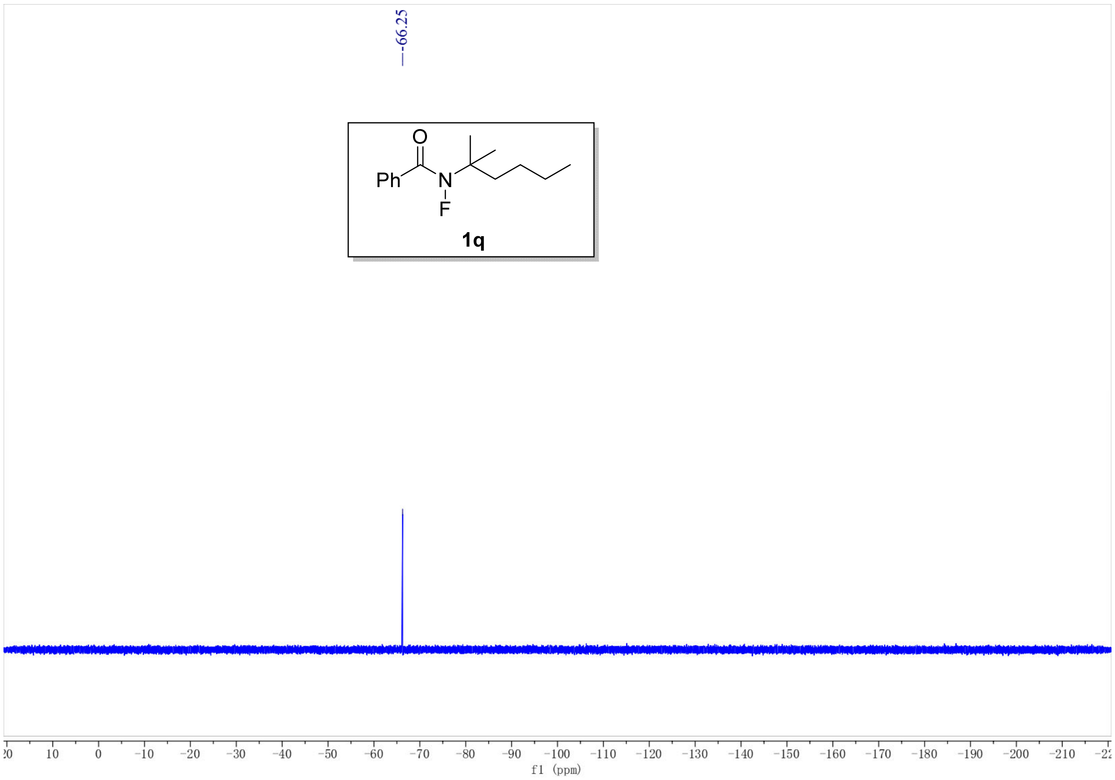

Supplementary Figure 63 ^{19}F NMR of **1q**

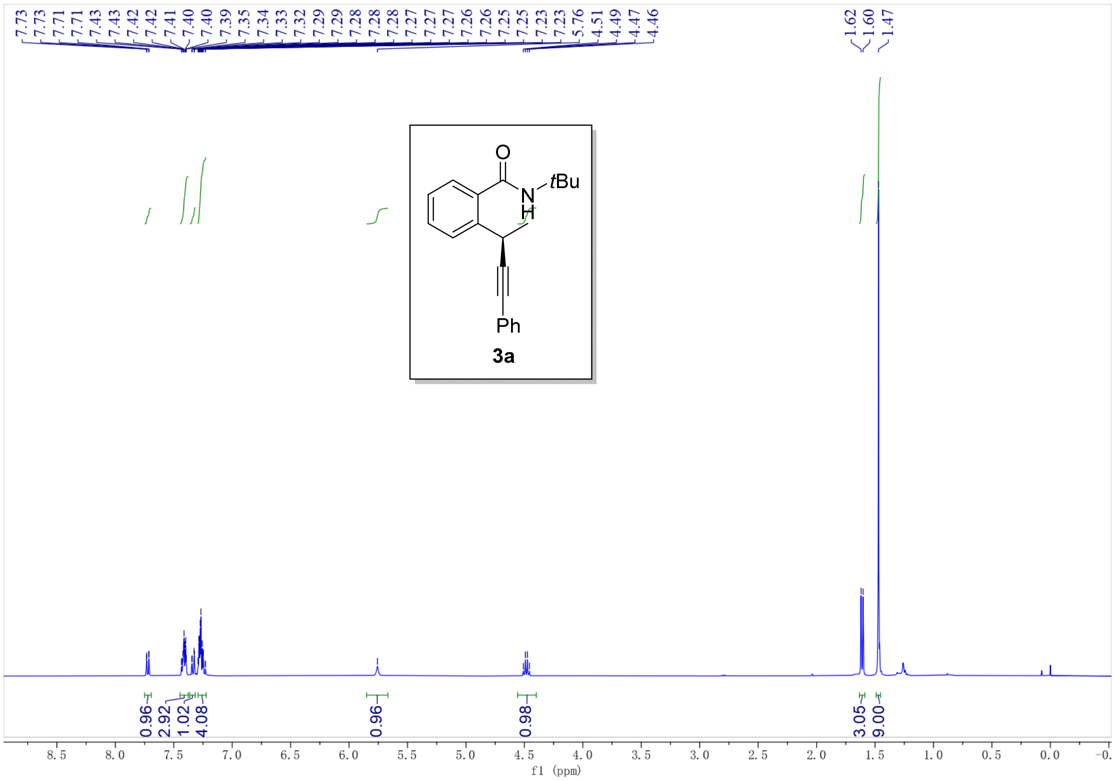

Supplementary Figure 64 ^{1}H NMR of **3a**

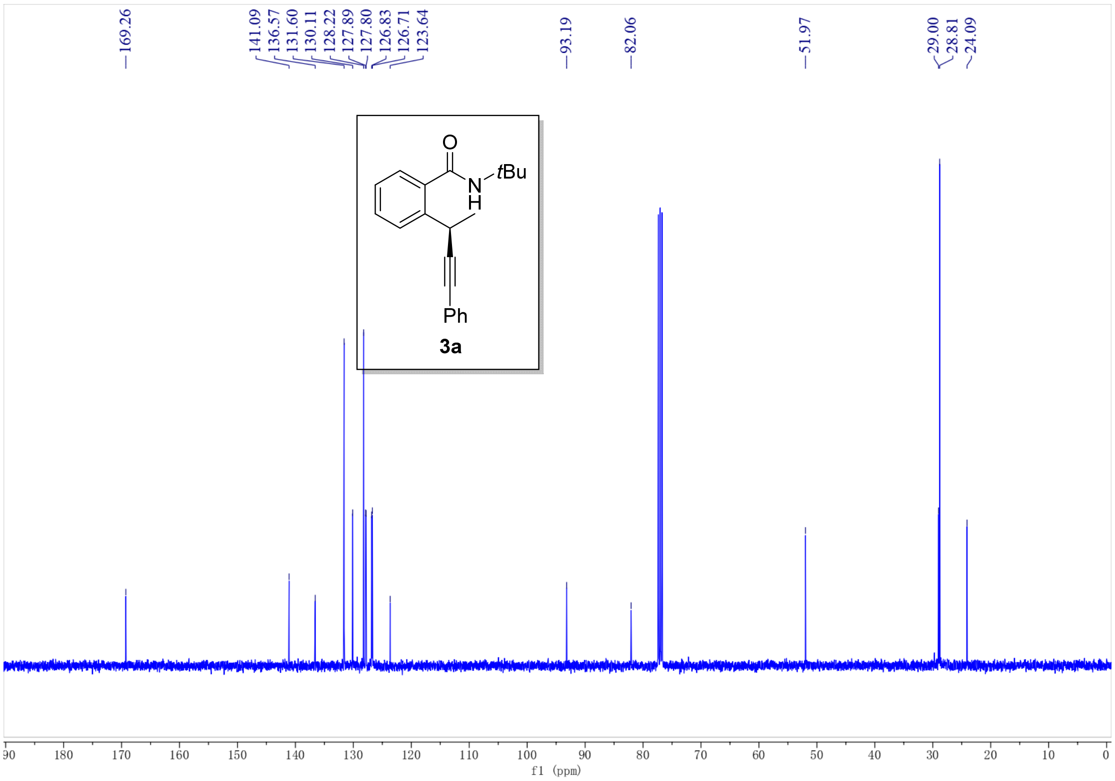

Supplementary Figure 65 ^{13}C NMR of **3a**

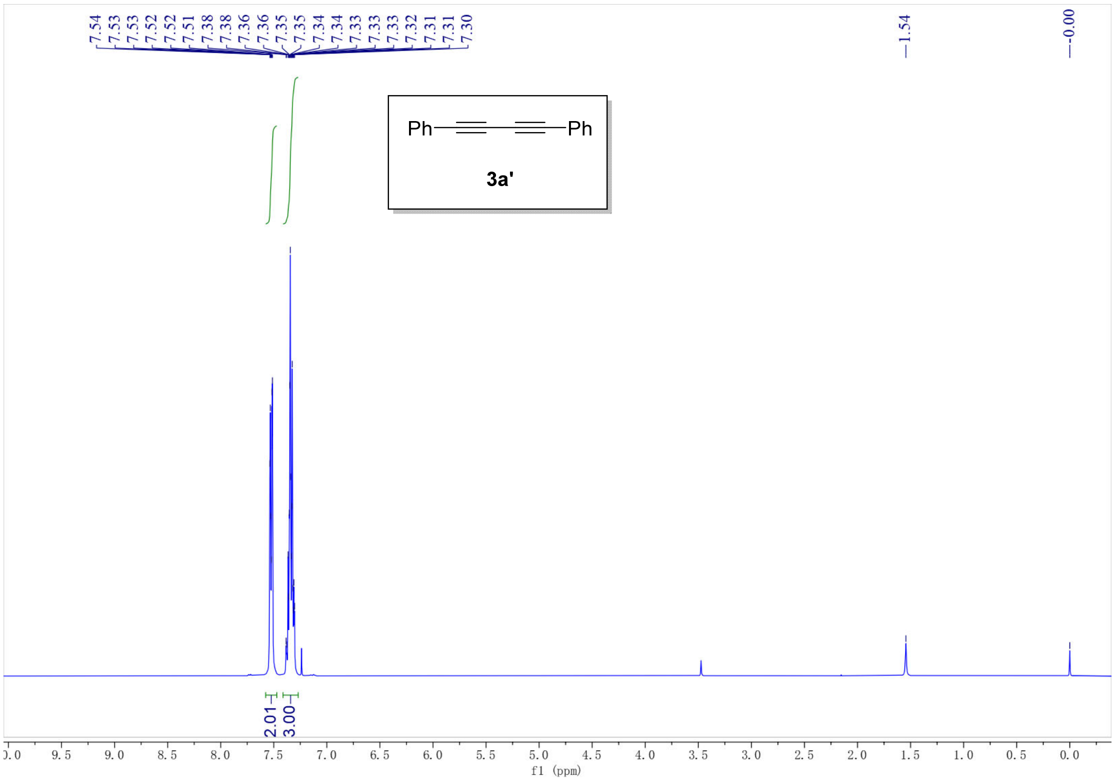

Supplementary Figure 66 ^{1}H NMR of **3a’**

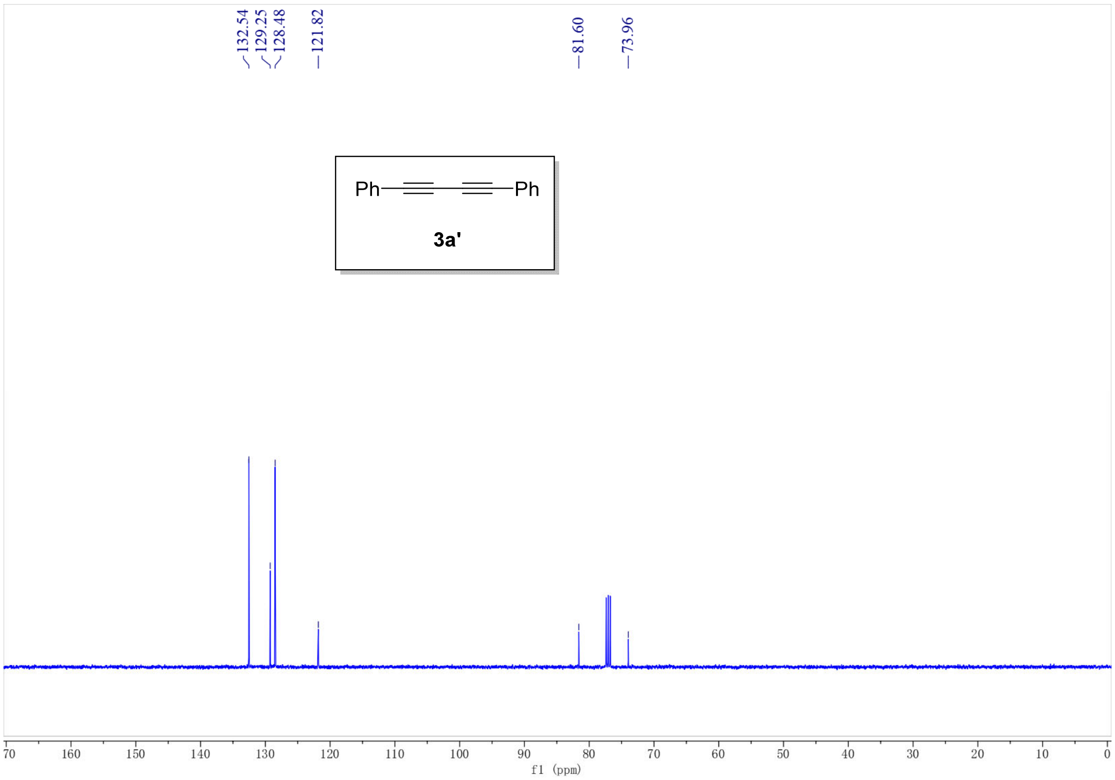

Supplementary Figure 67 ^{13}C NMR of **3a'**

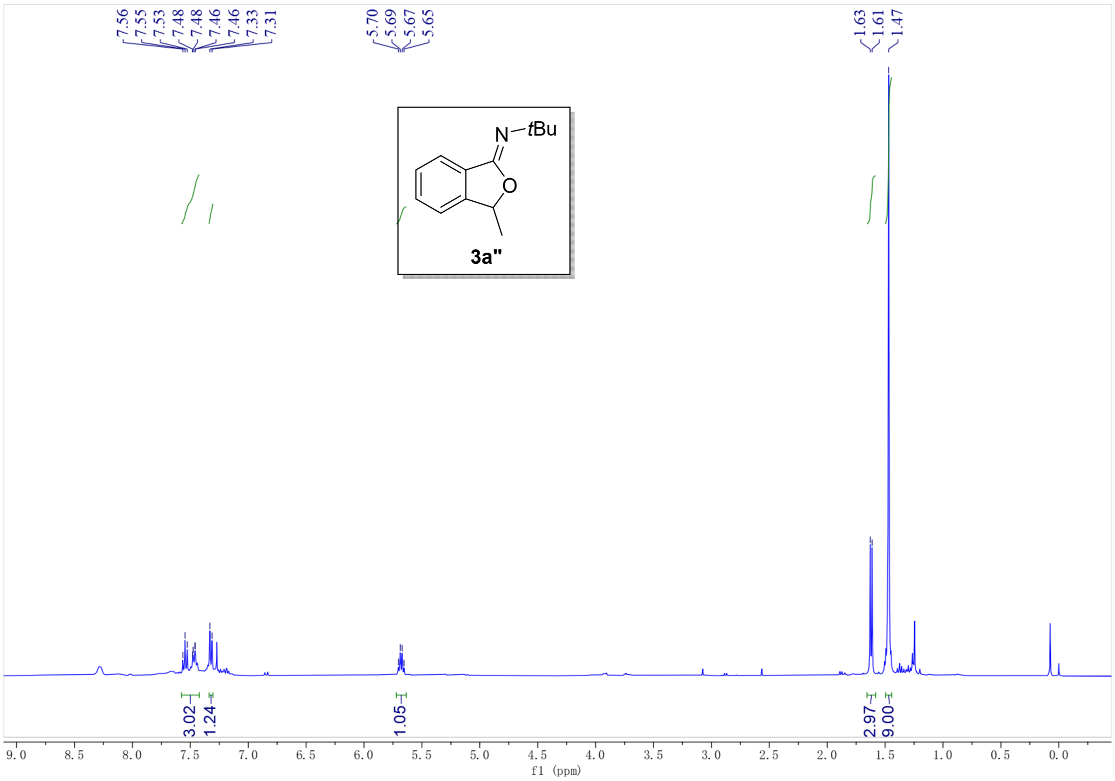

Supplementary Figure 68 ^{1}H NMR of **3a"**

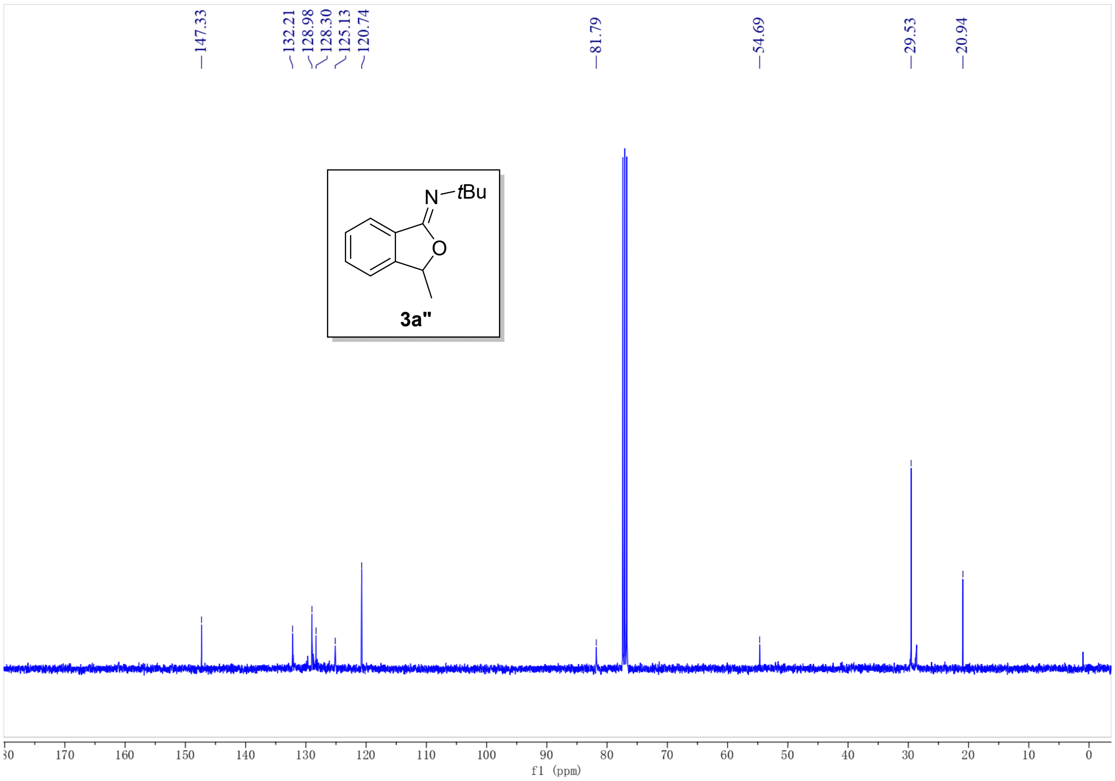

Supplementary Figure 69 ^{13}C NMR of **3a”**

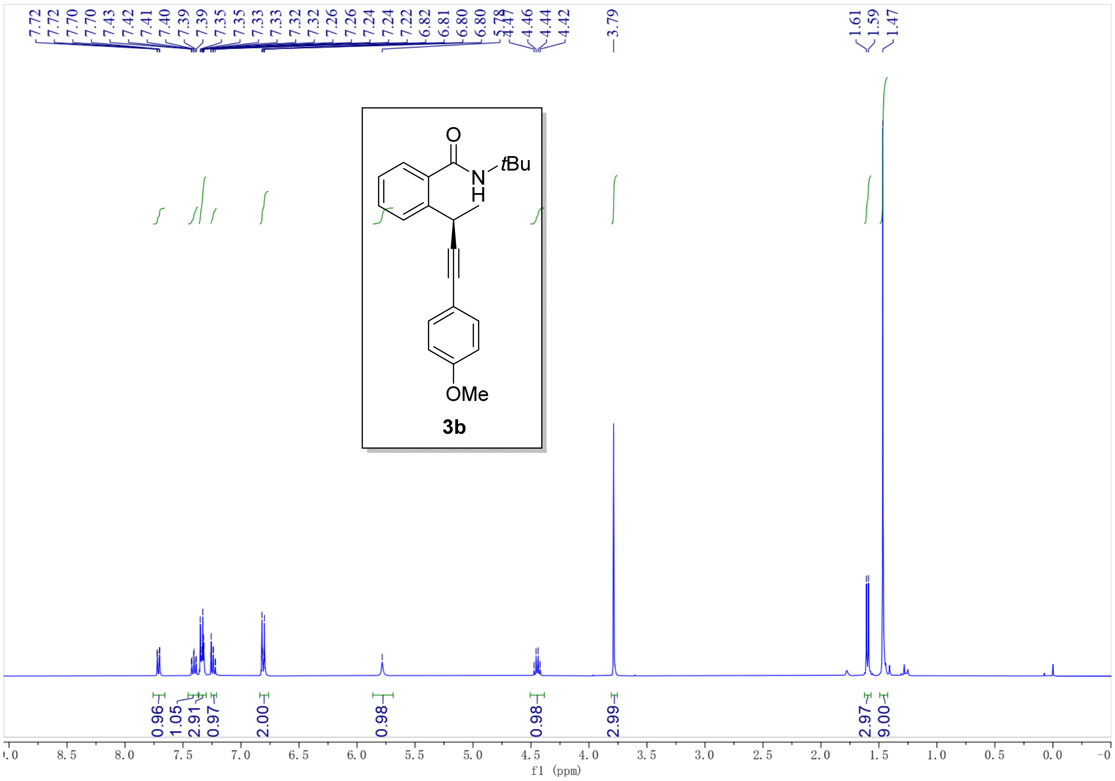

Supplementary Figure 70 ^{1}H NMR of **3b**

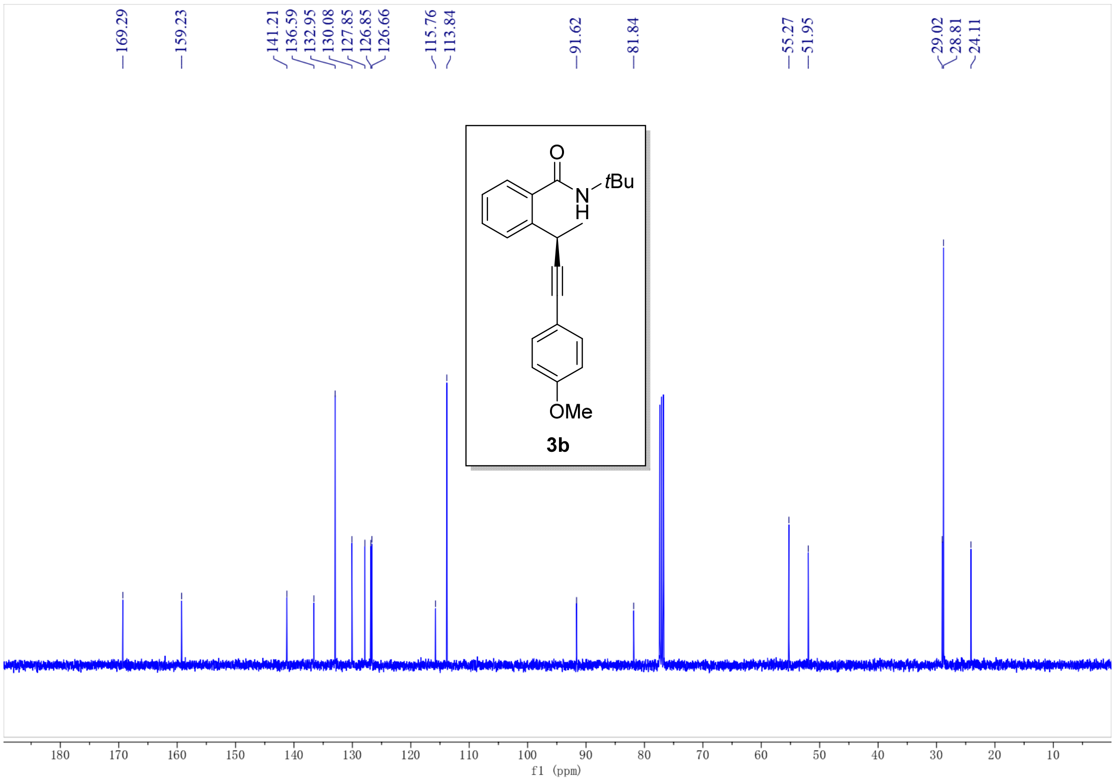

Supplementary Figure 71 ^{13}C NMR of **3b**

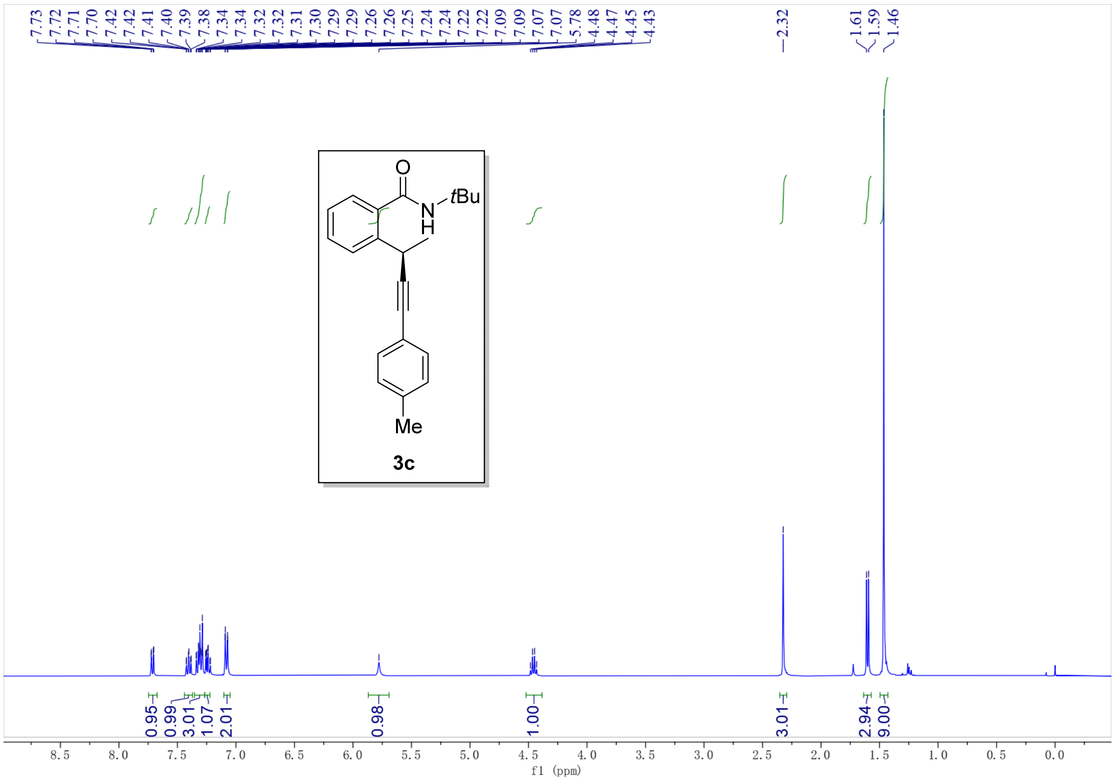

Supplementary Figure 72 ^{1}H NMR of **3c**

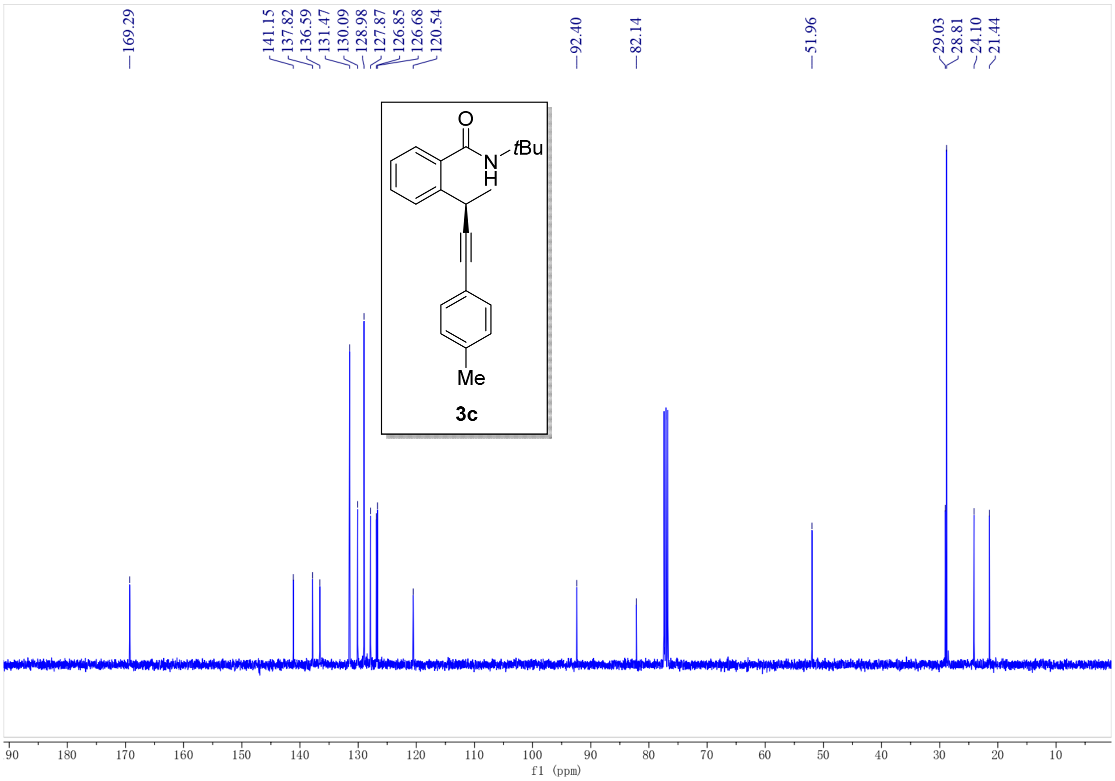

Supplementary Figure 73 ^{13}C NMR of **3c**

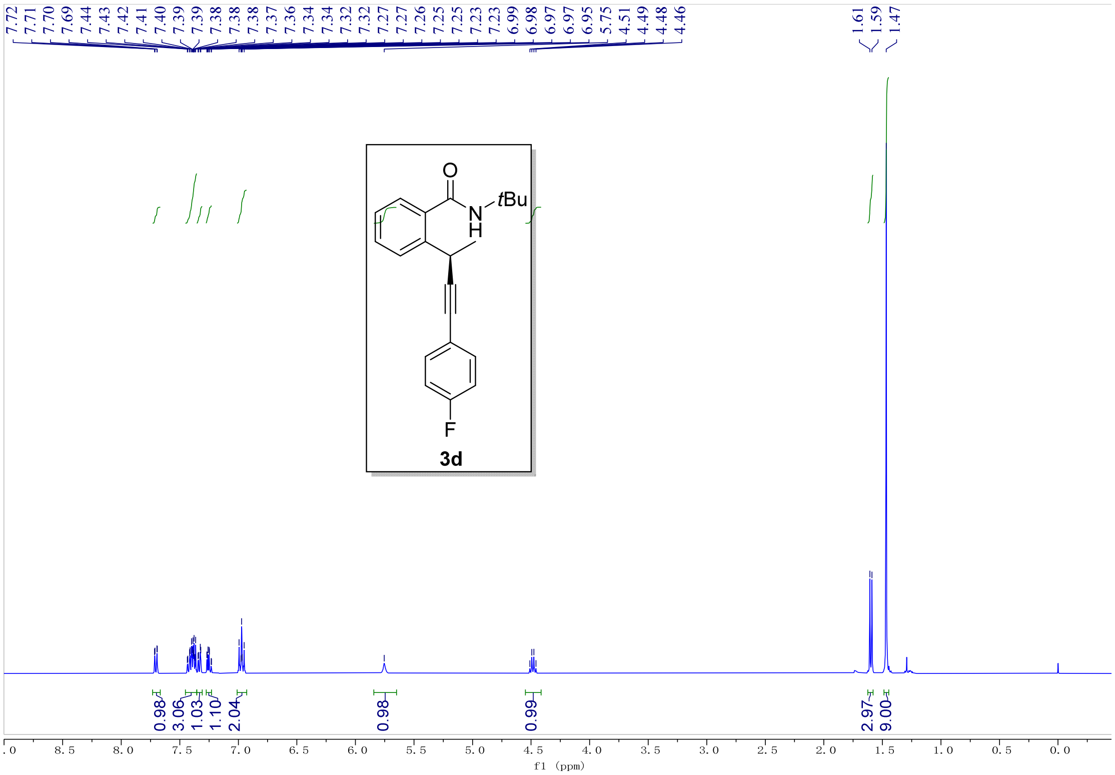

Supplementary Figure 74 ^{1}H NMR of **3d**

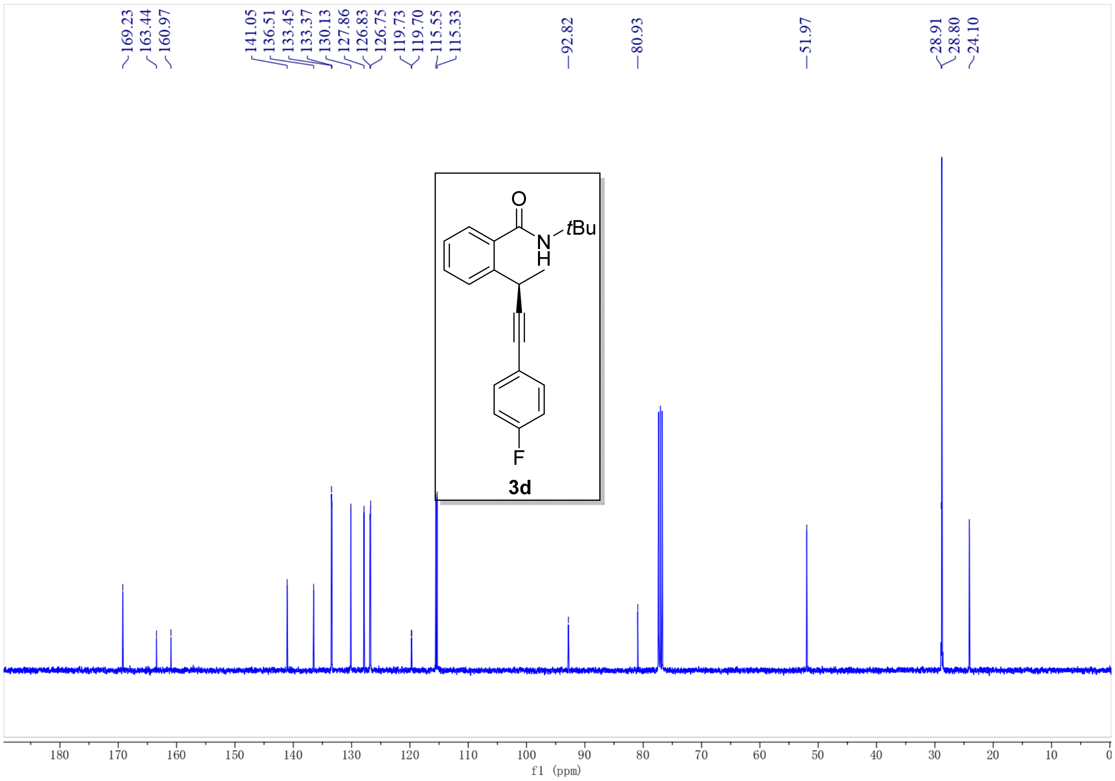

Supplementary Figure 75 ^{13}C NMR of **3d**

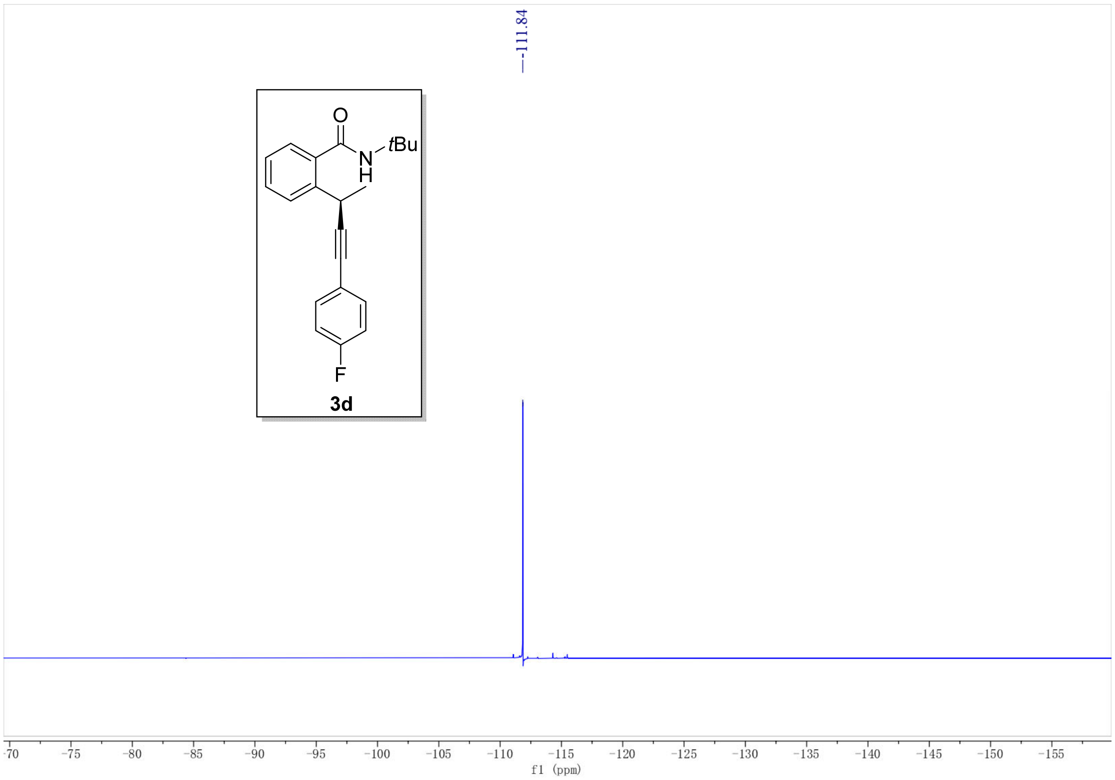

Supplementary Figure 76 ^{19}F NMR of **3d**

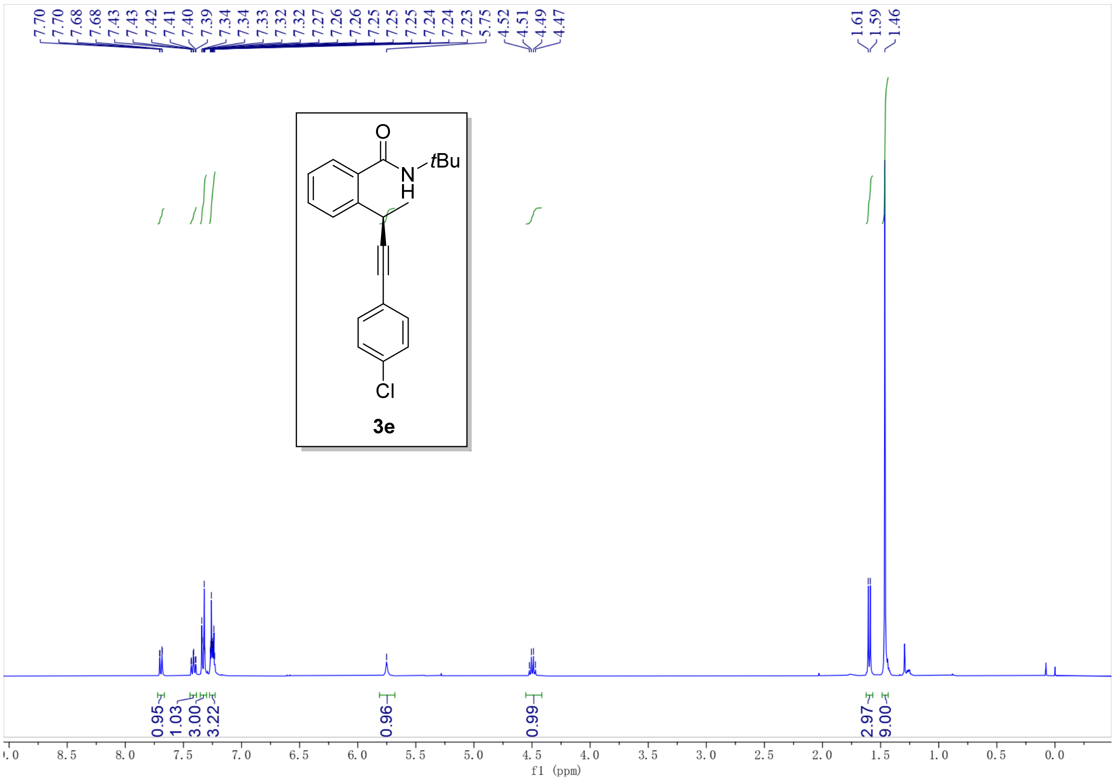

Supplementary Figure 77 ^{1}H NMR of **3e**

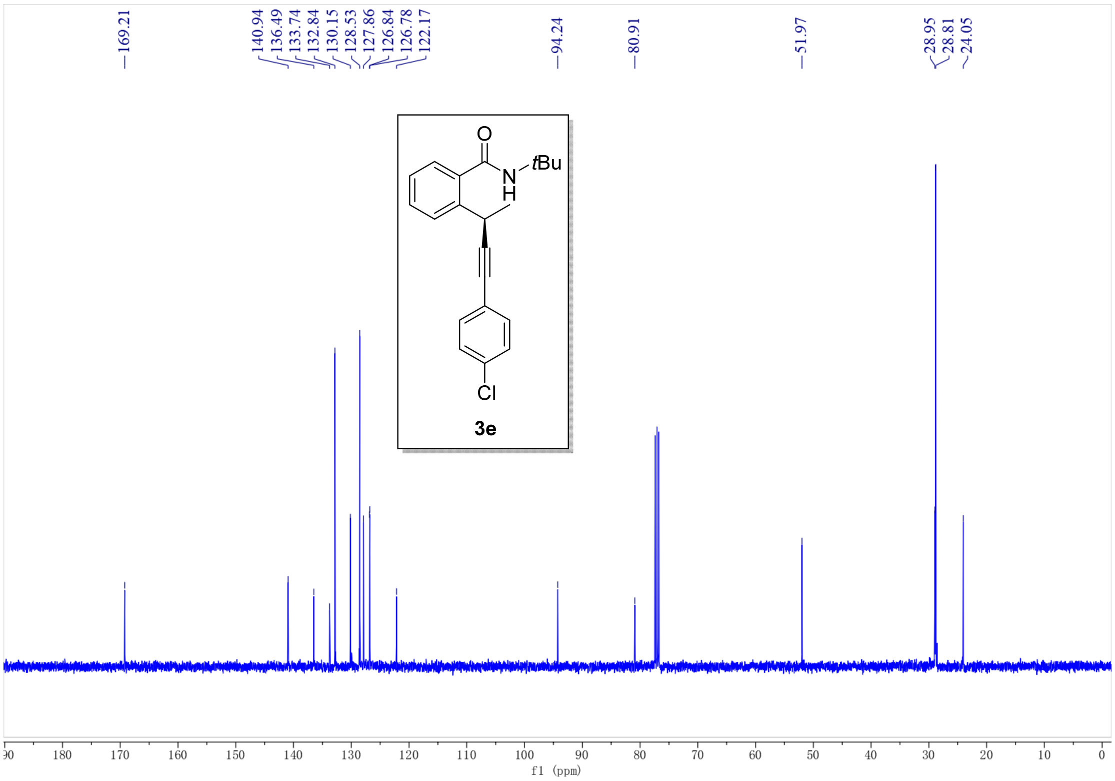

Supplementary Figure 78 ^{13}C NMR of **3e**

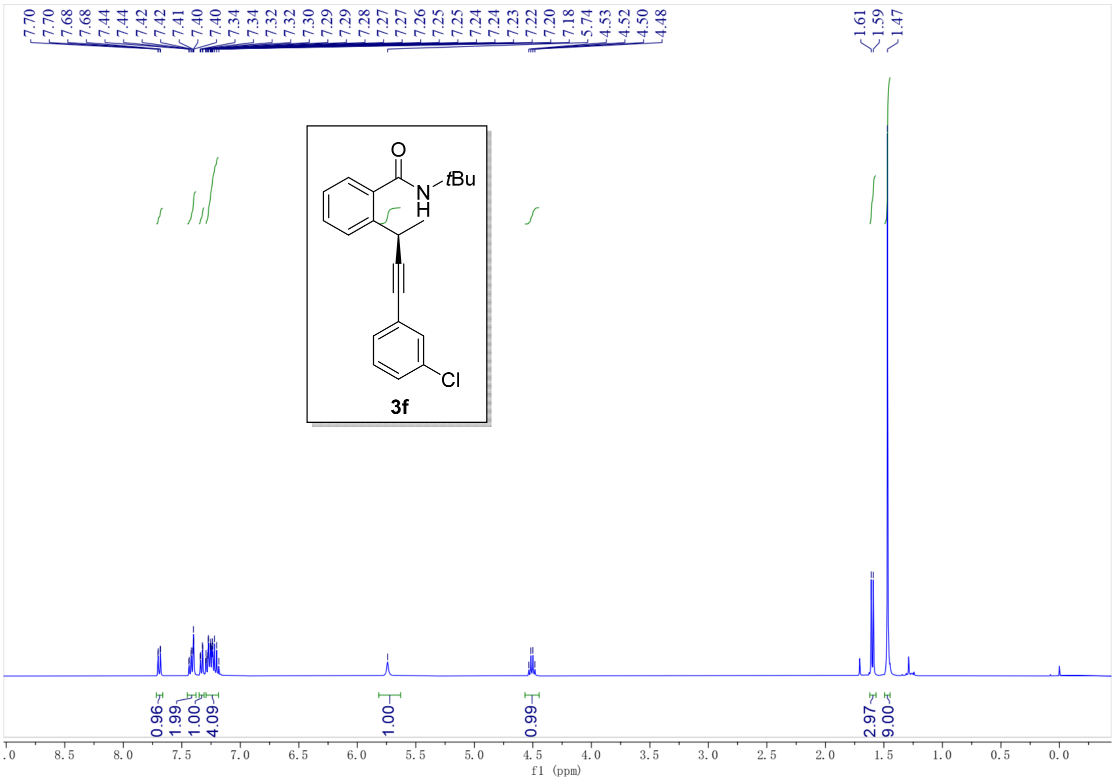

Supplementary Figure 79 ^{1}H NMR of **3f**

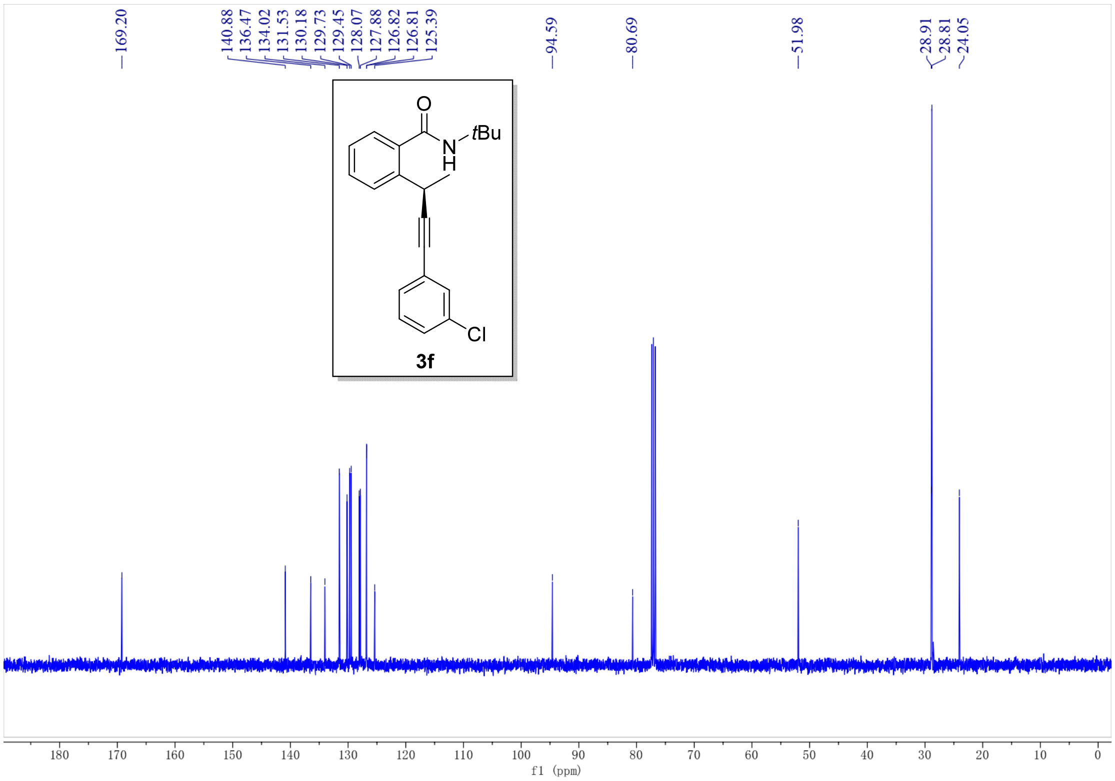

Supplementary Figure 80 ^{13}C NMR of **3f**

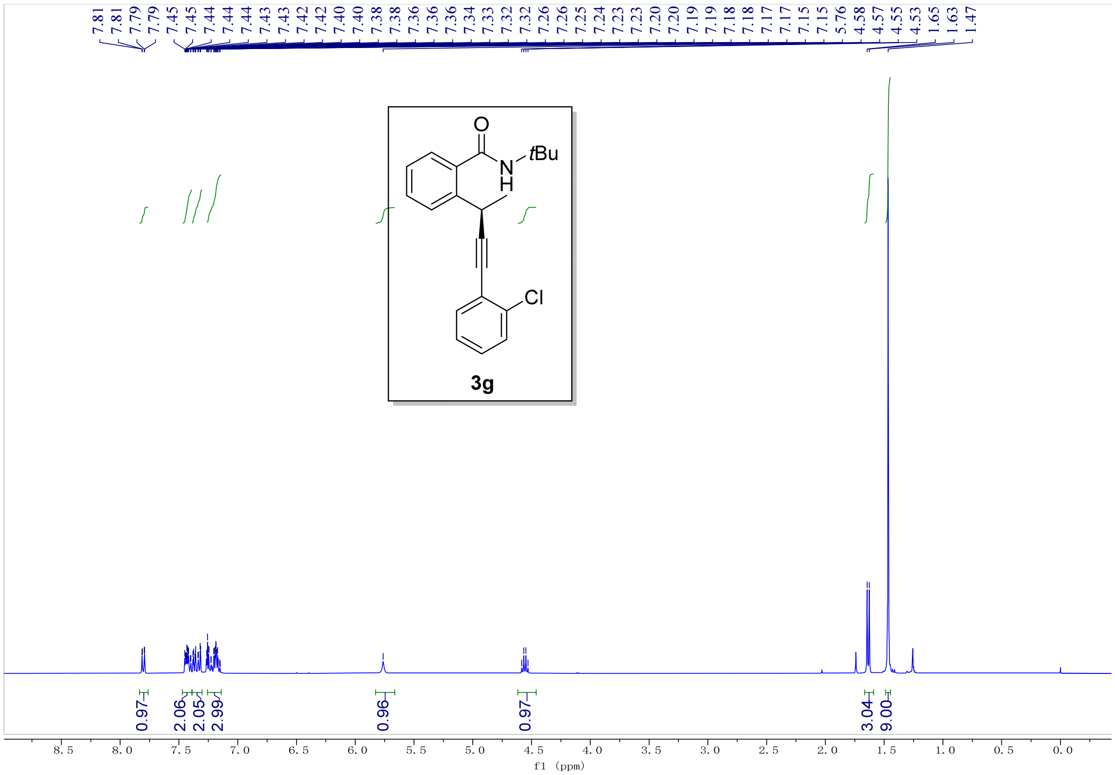

Supplementary Figure 81 ^{1}H NMR of **3g**

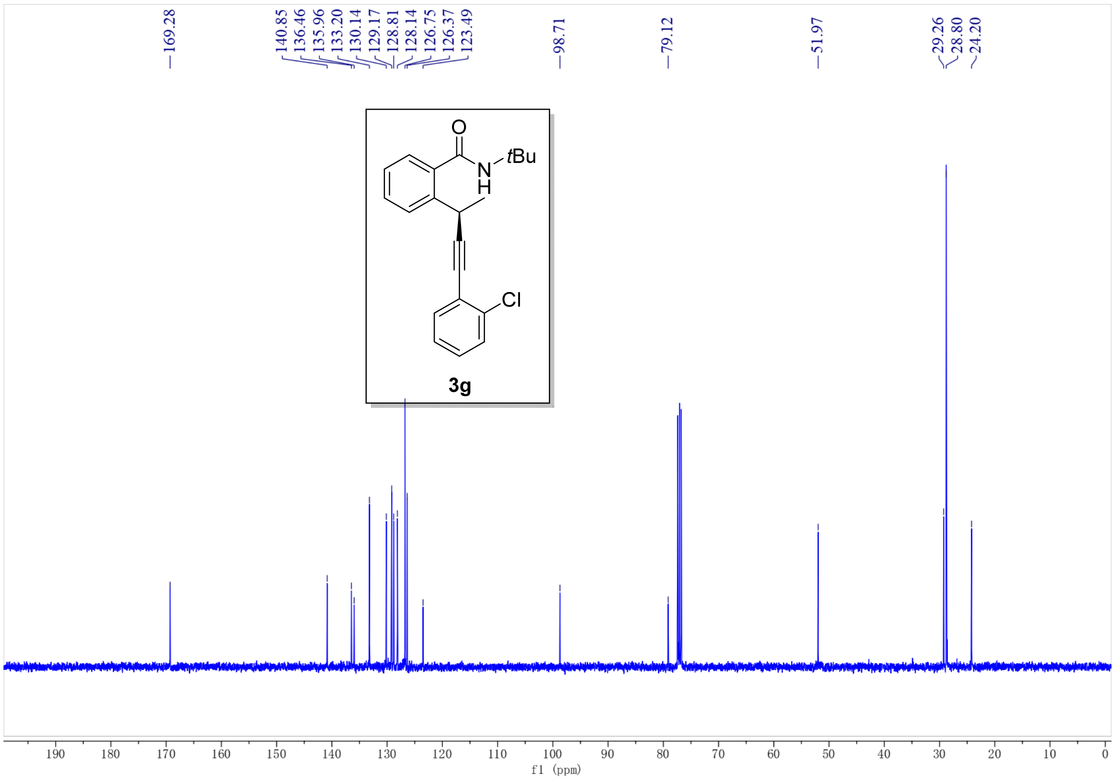

Supplementary Figure 82 ^{13}C NMR of **3g**

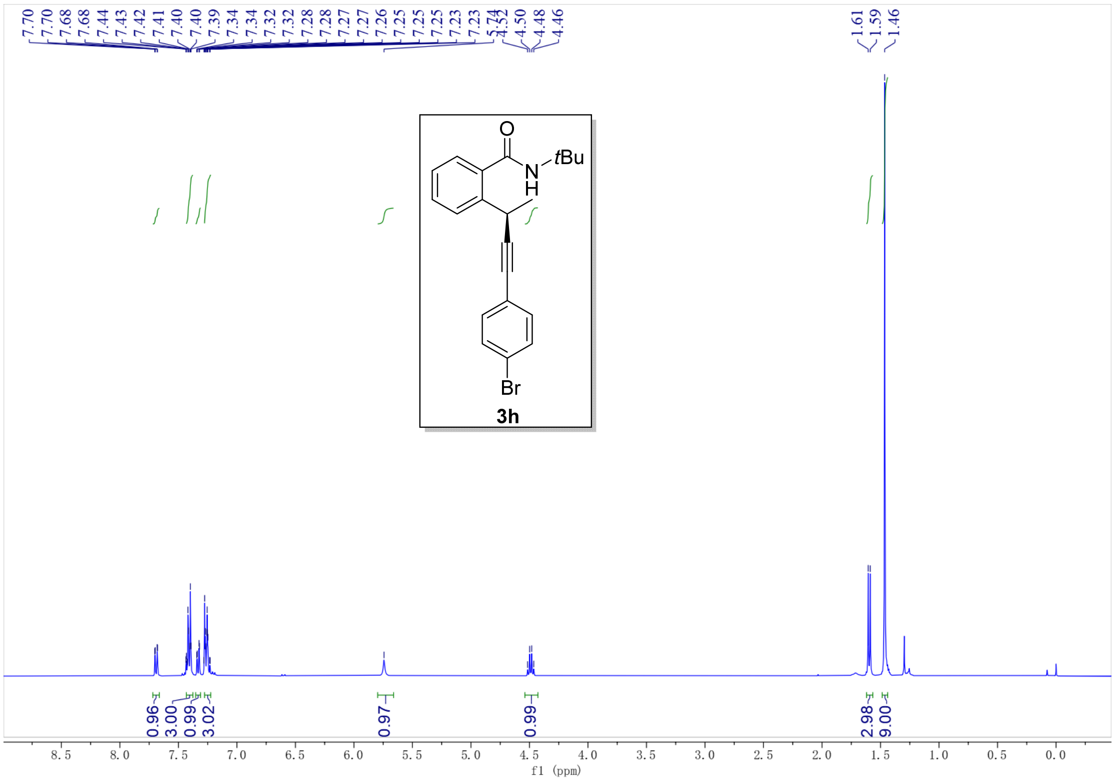

Supplementary Figure 83 ^{1}H NMR of **3h**

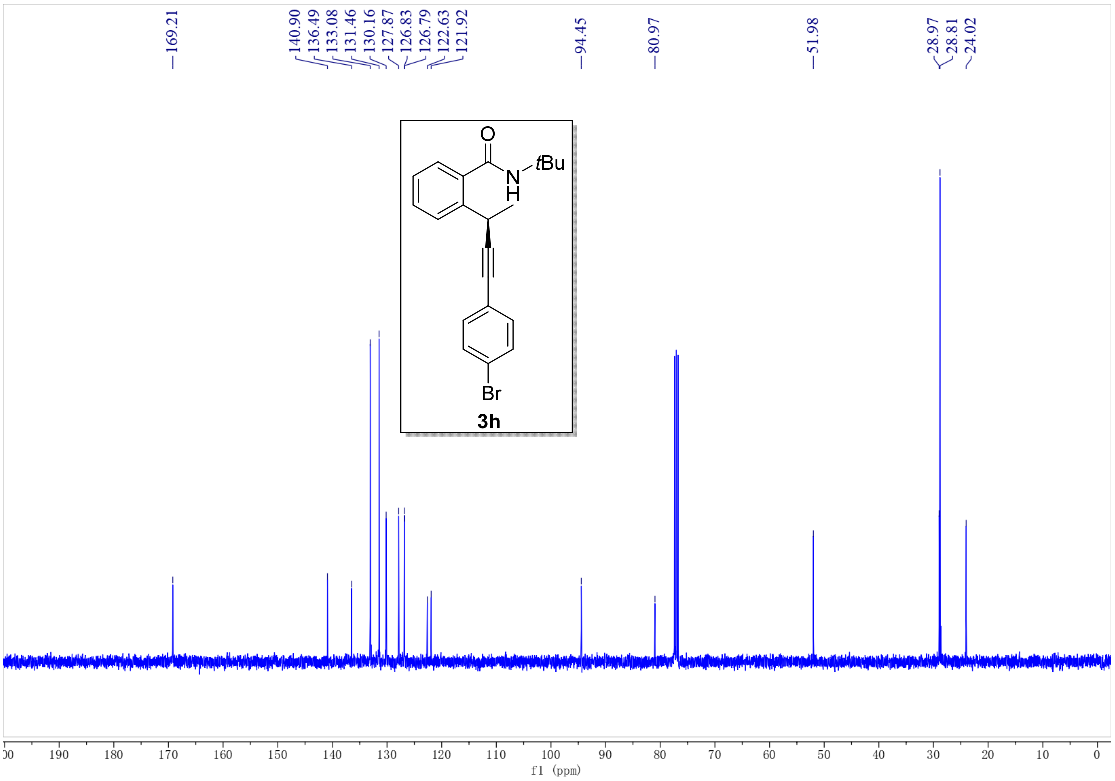

Supplementary Figure 84 ^{13}C NMR of **3h**

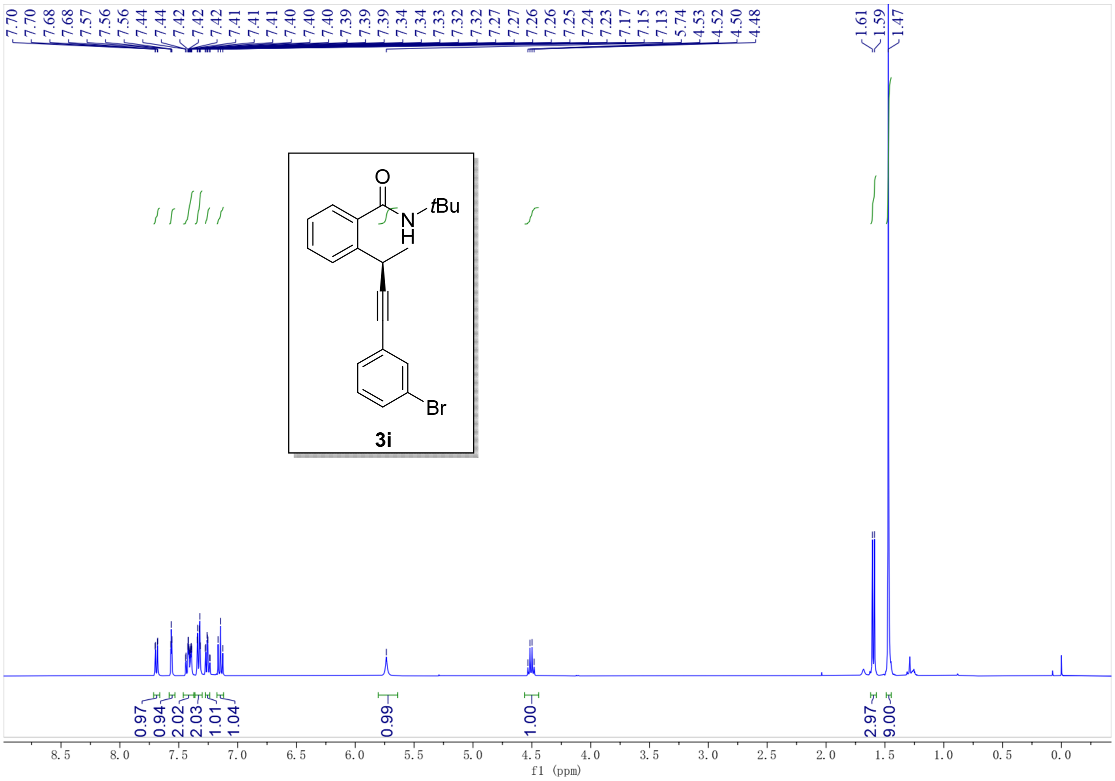

Supplementary Figure 85 ^{1}H NMR of **3i**

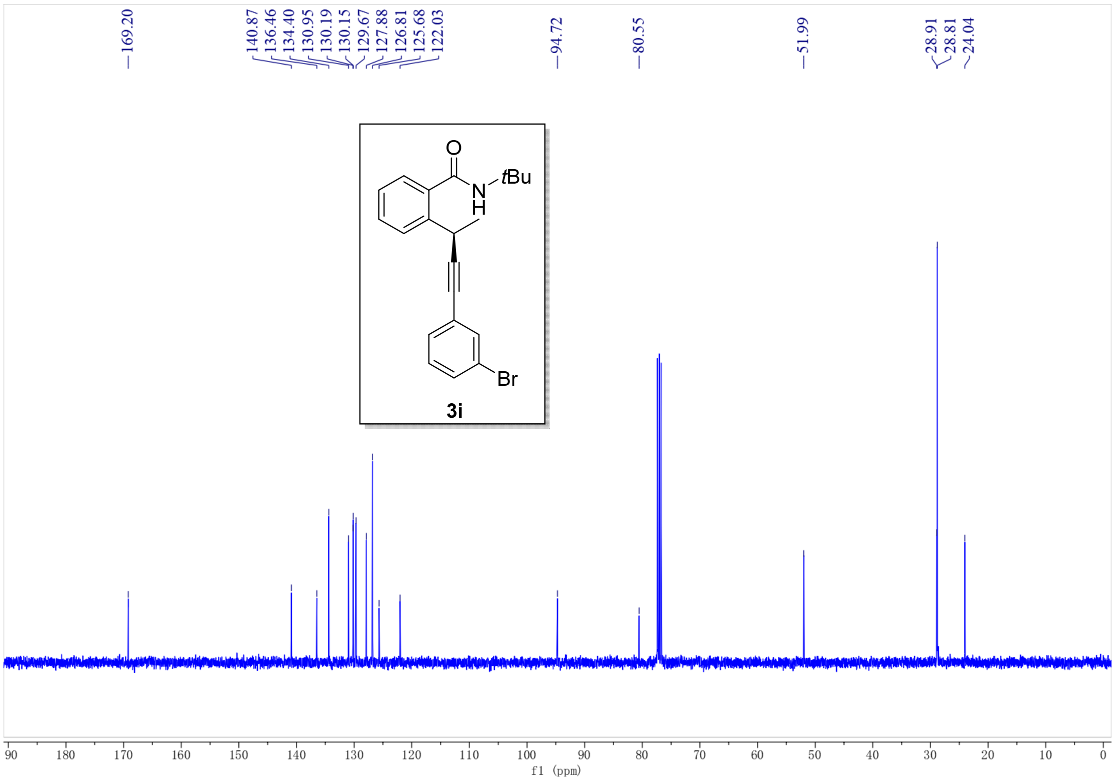

Supplementary Figure 86 ^{13}C NMR of **3i**

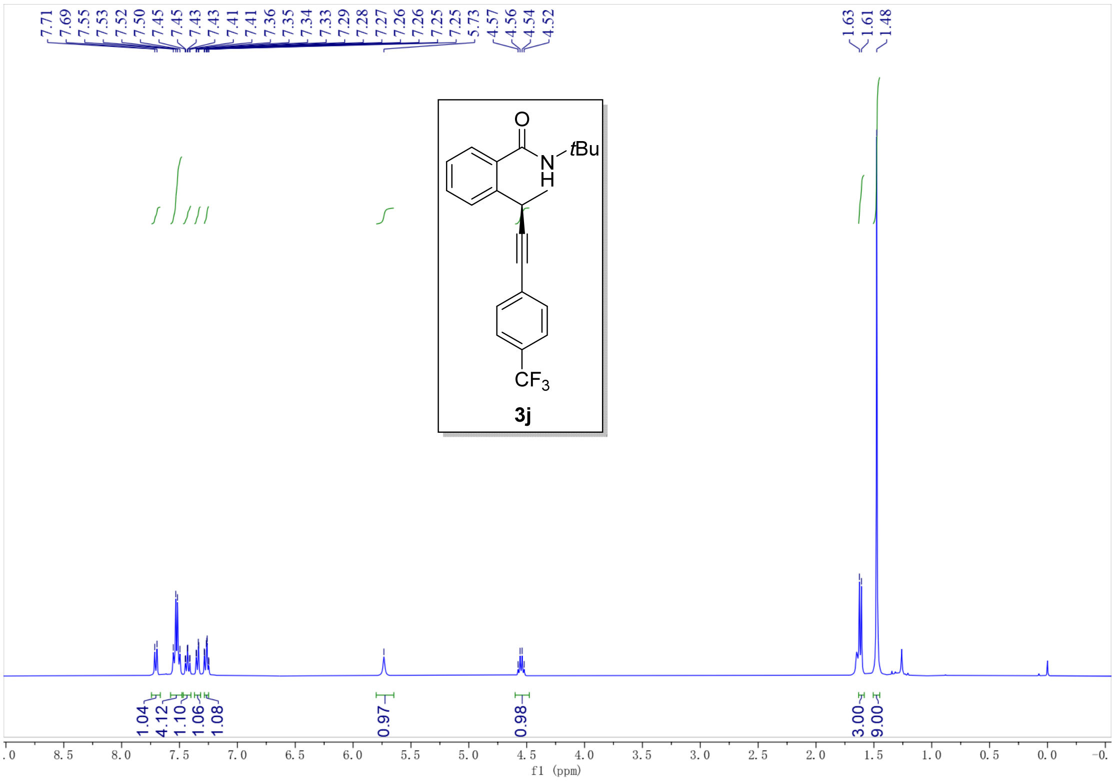

Supplementary Figure 87 ^{1}H NMR of **3j**

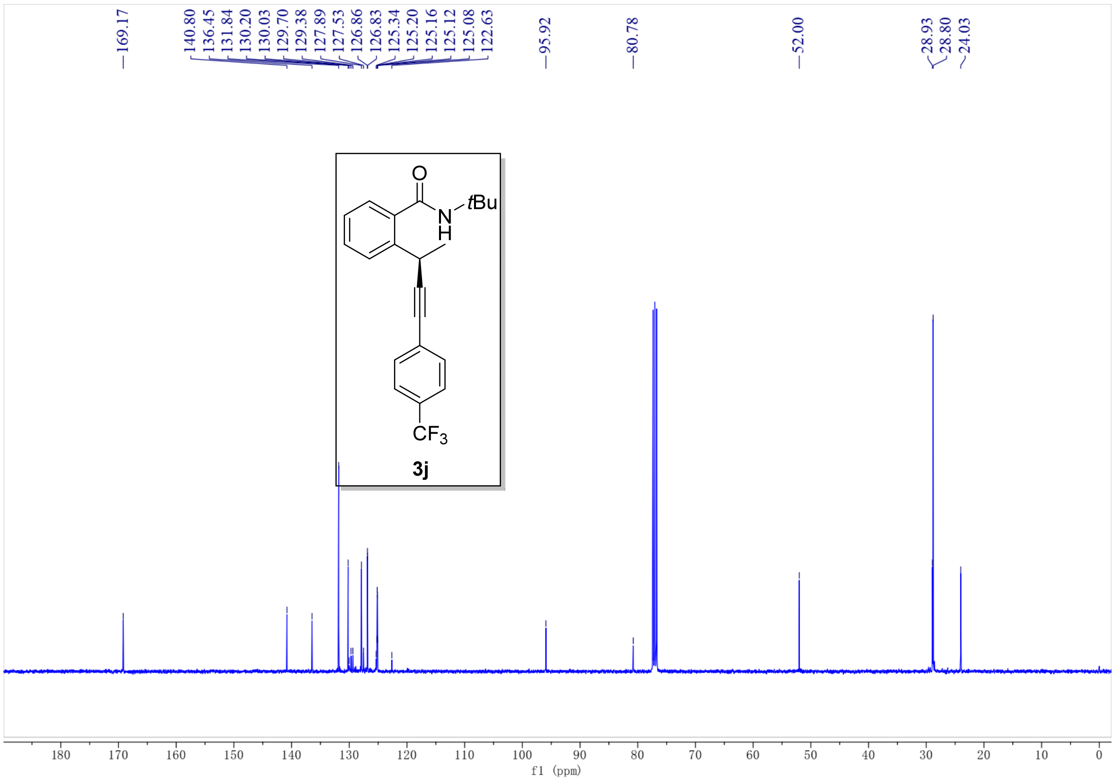

Supplementary Figure 88 ^{13}C NMR of **3j**

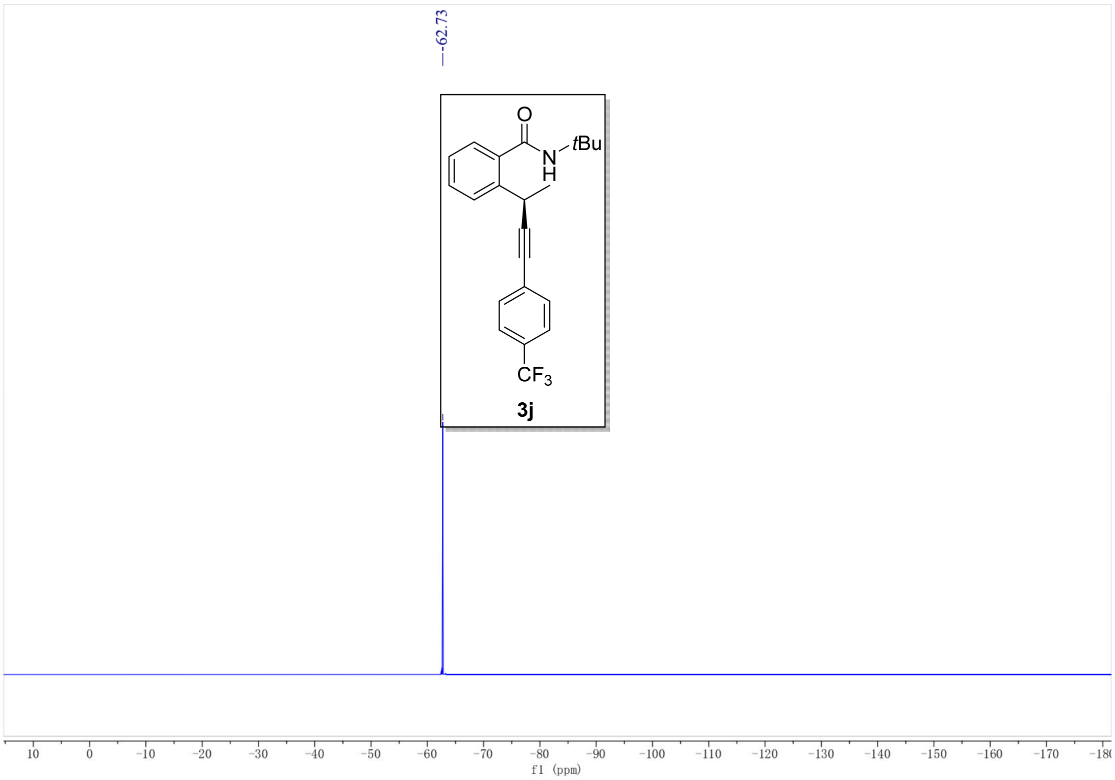

Supplementary Figure 89 ^{19}F NMR of **3j**

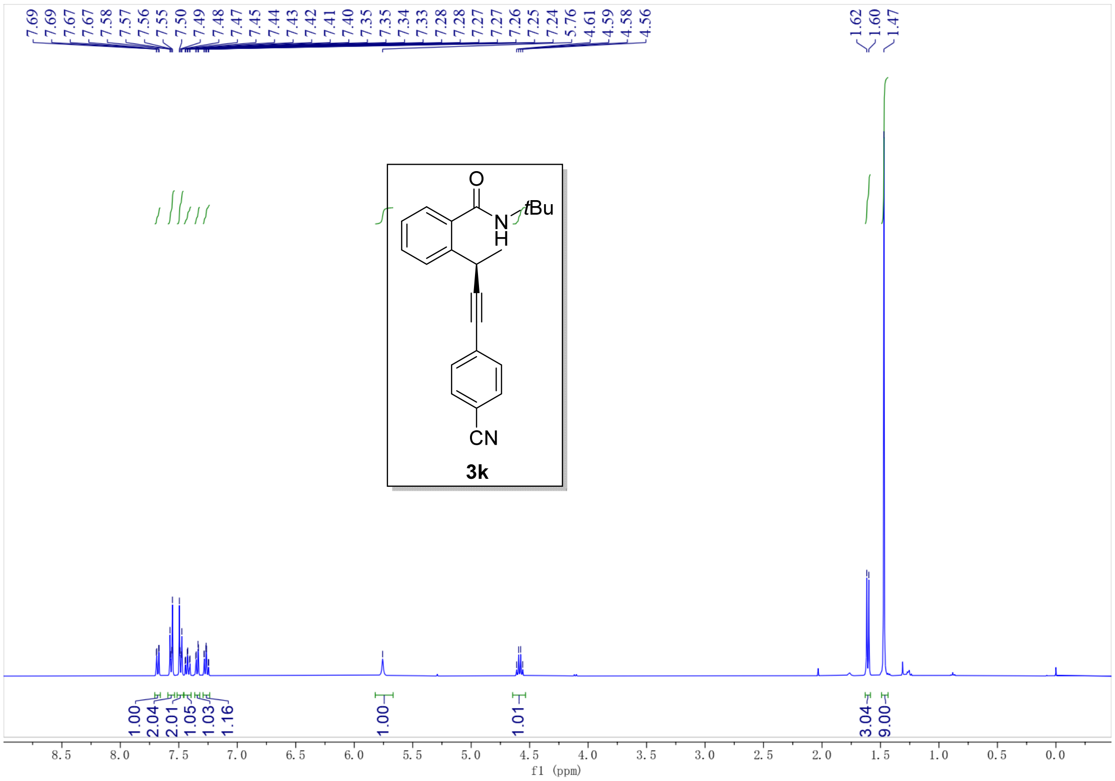

Supplementary Figure 90 ^{1}H NMR of **3k**

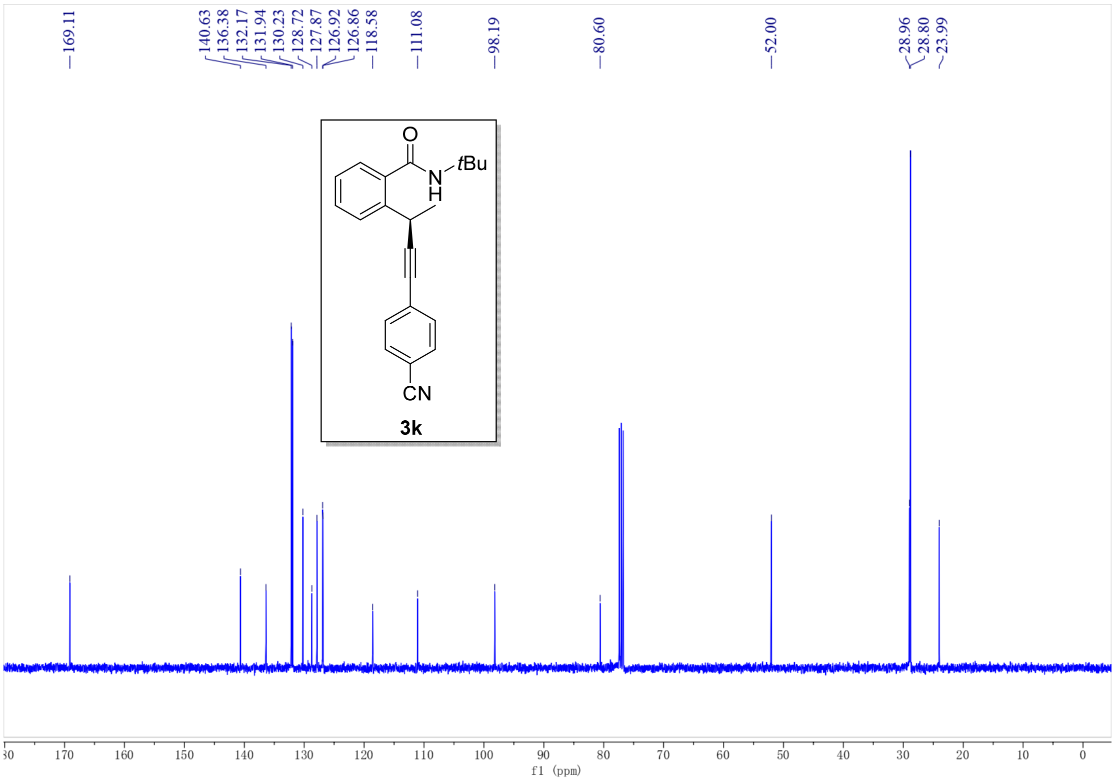

Supplementary Figure 91 ^{13}C NMR of **3k**

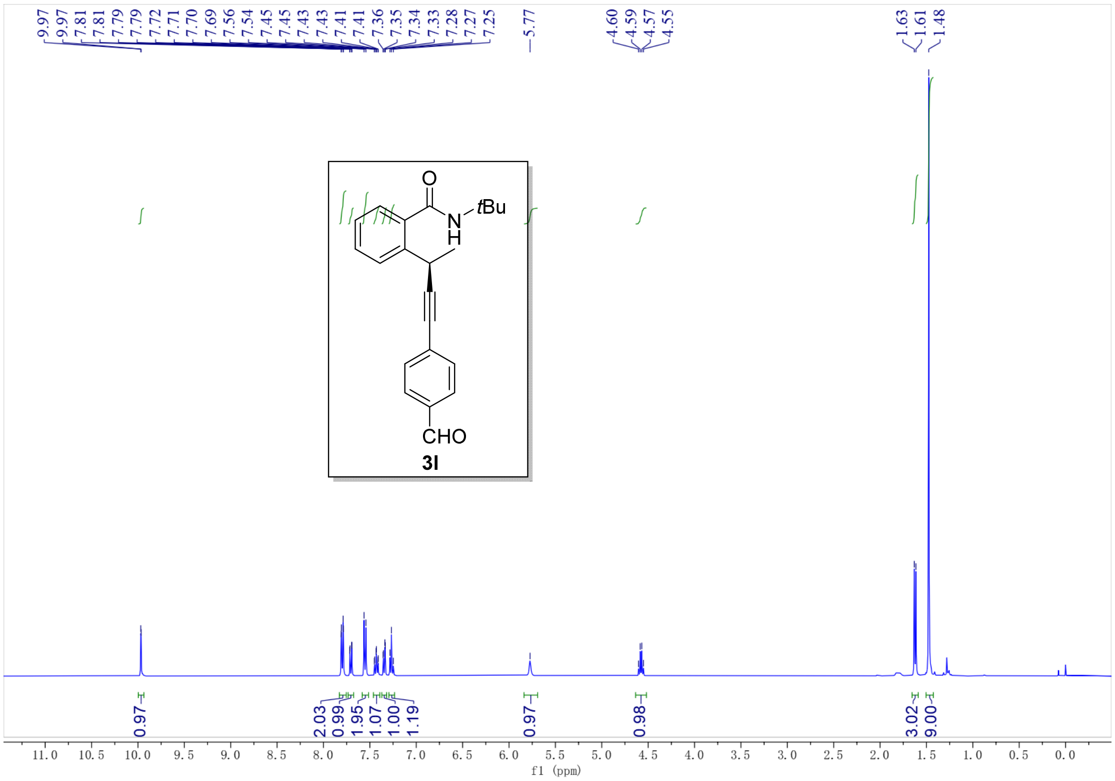

Supplementary Figure 92 ^{1}H NMR of **3l**

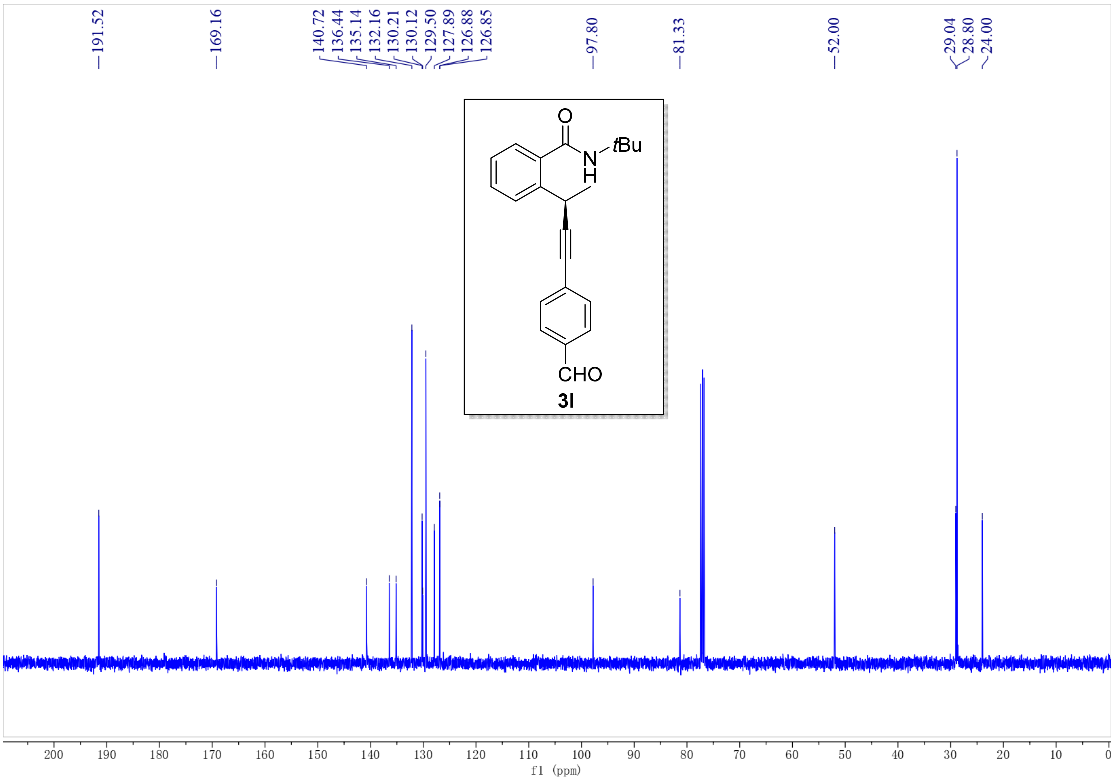

Supplementary Figure 93 ^{13}C NMR of **3l**

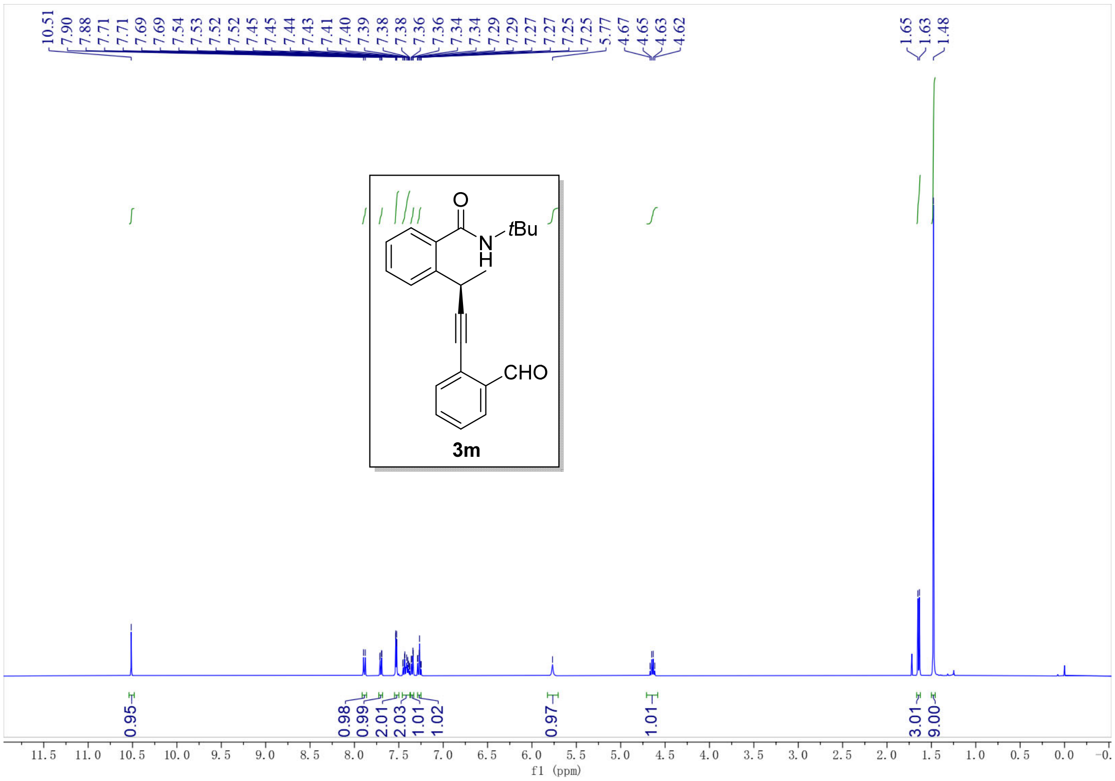

Supplementary Figure 94 ^{1}H NMR of **3m**

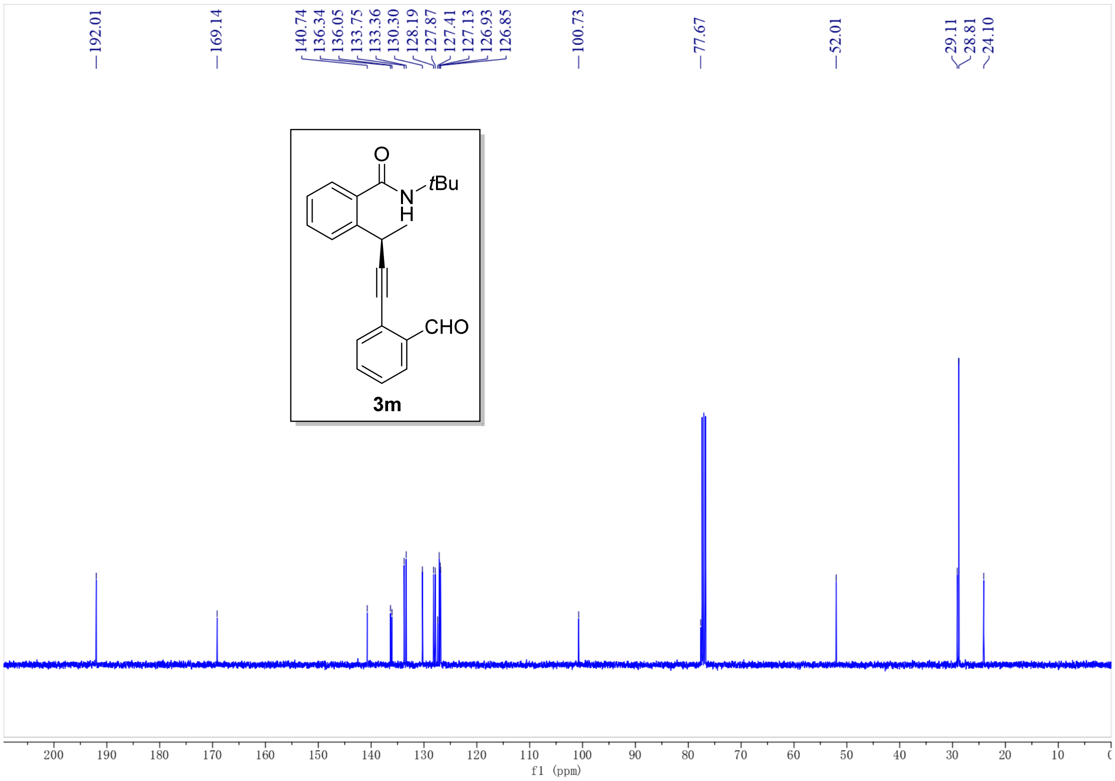

Supplementary Figure 95 ^{13}C NMR of **3m**

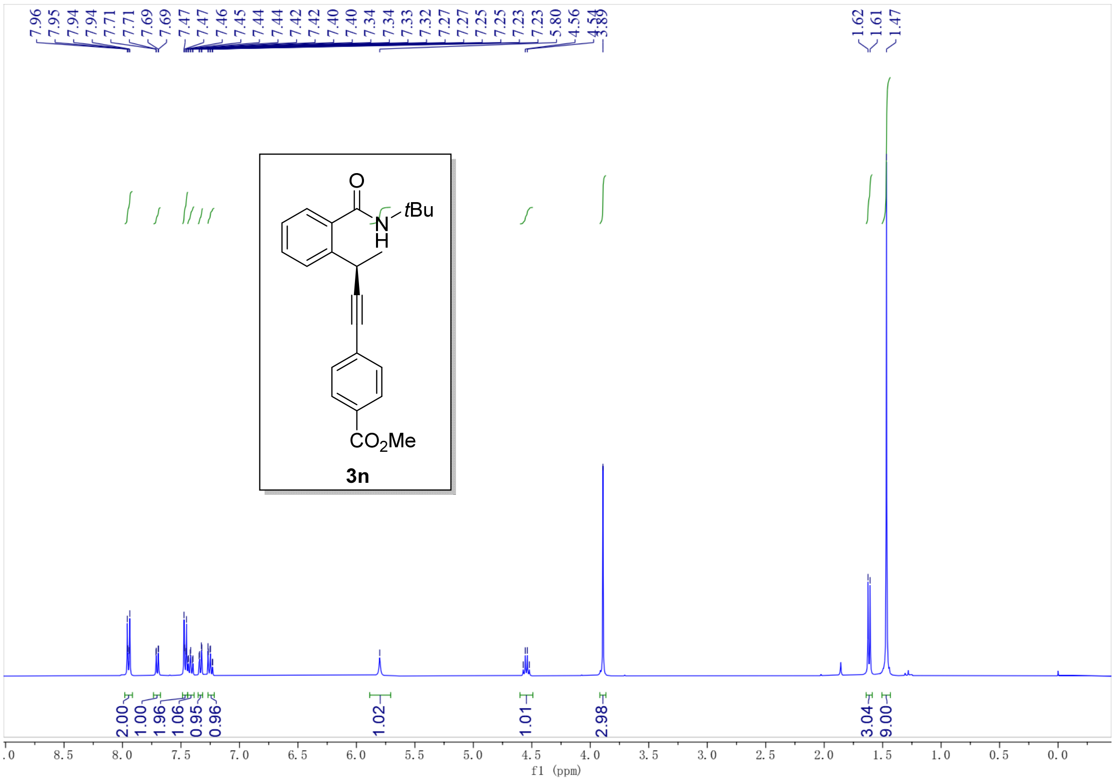

Supplementary Figure 96 ^{1}H NMR of **3n**

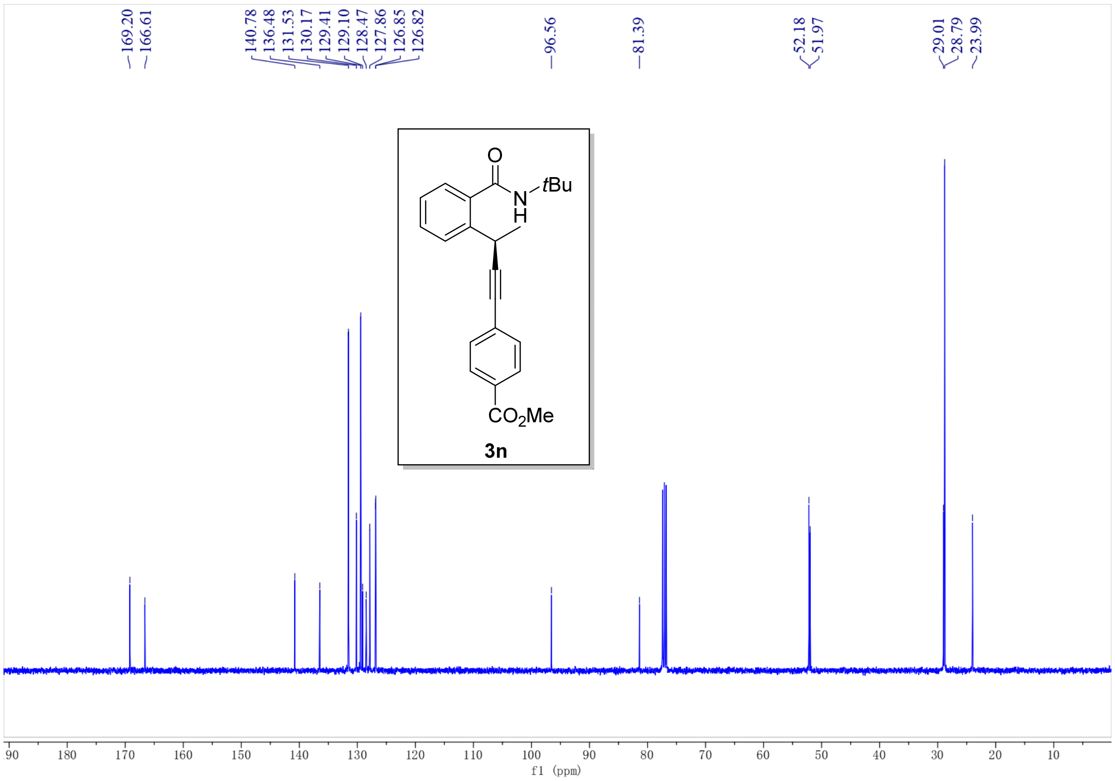

Supplementary Figure 97 ^{13}C NMR of **3n**

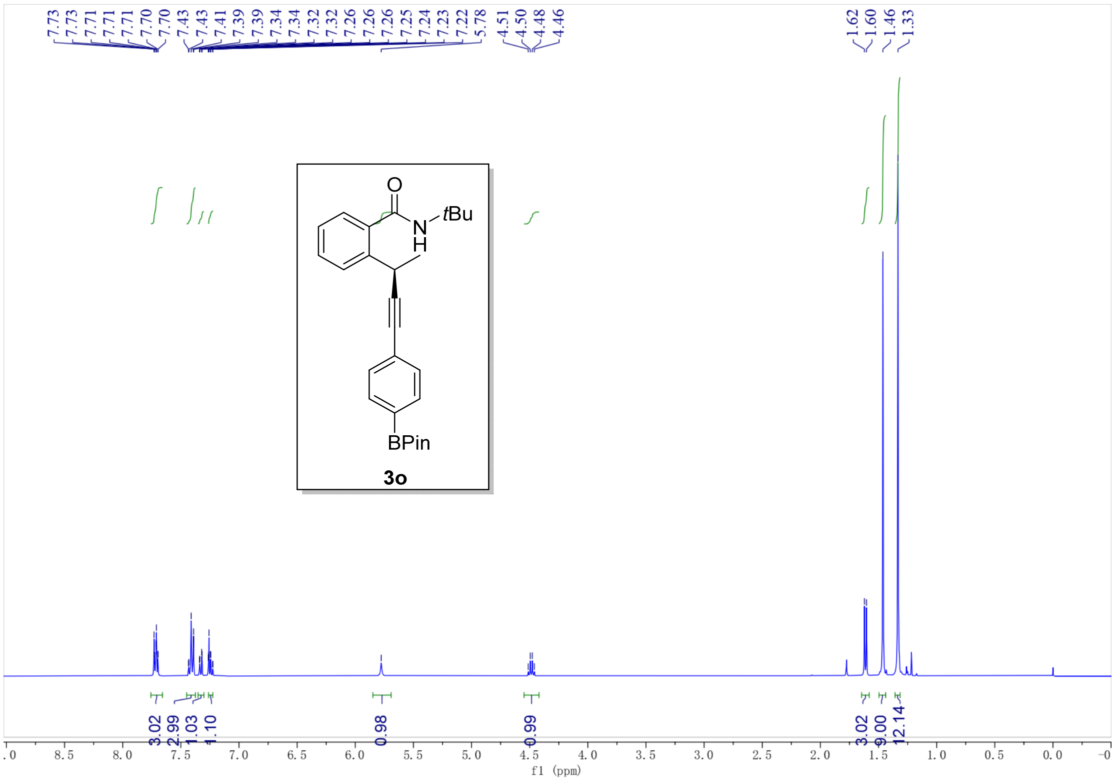

Supplementary Figure 98 ^{1}H NMR of **3o**

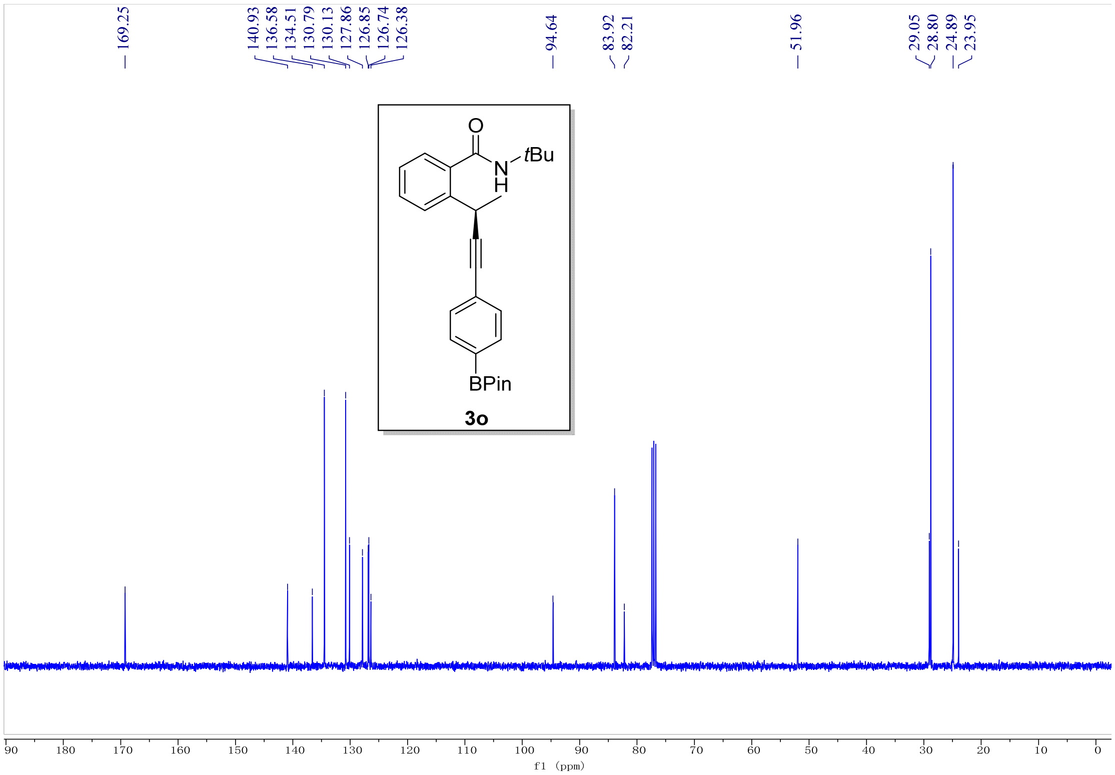

Supplementary Figure 99 ^{13}C NMR of **3o**

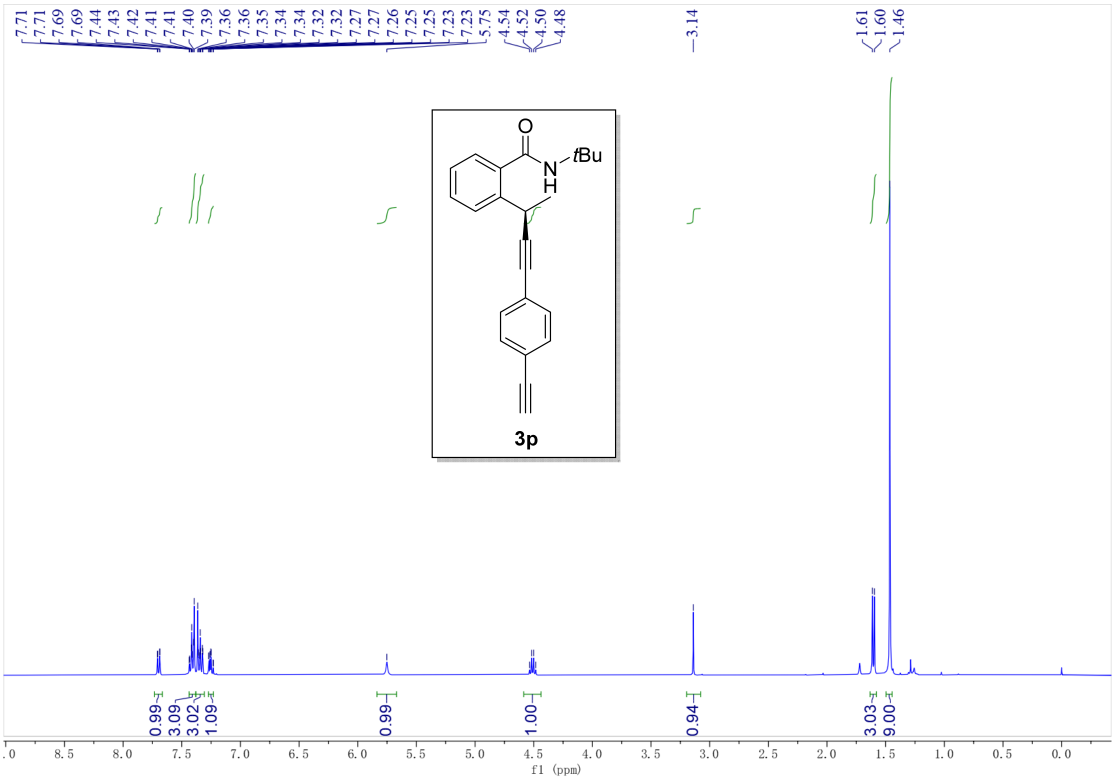

Supplementary Figure 100 ^{1}H NMR of **3p**

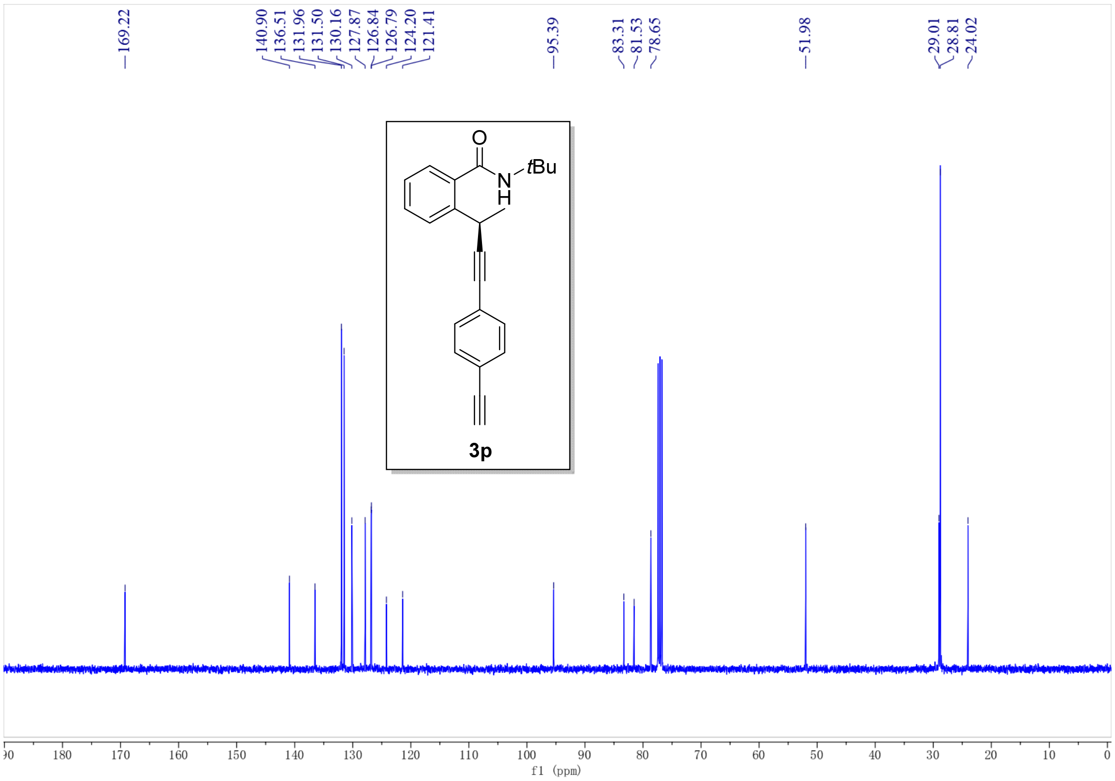

Supplementary Figure 101 ^{13}C NMR of **3p**

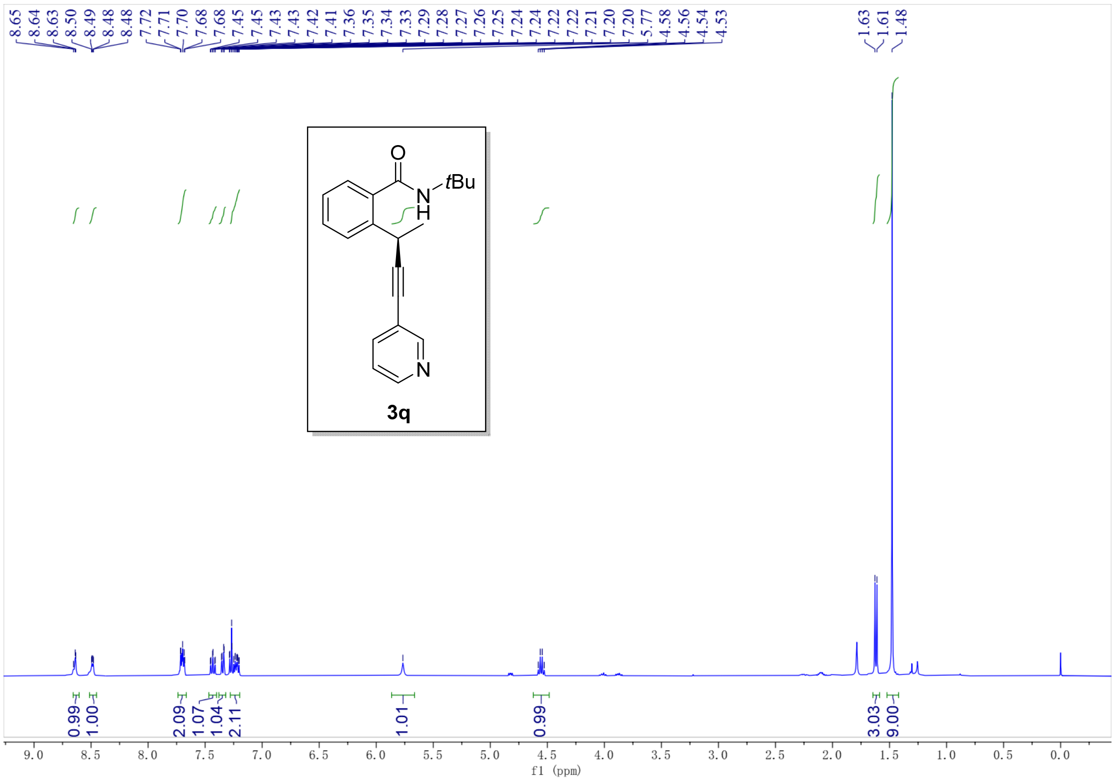

Supplementary Figure 102 ^{1}H NMR of **3q**

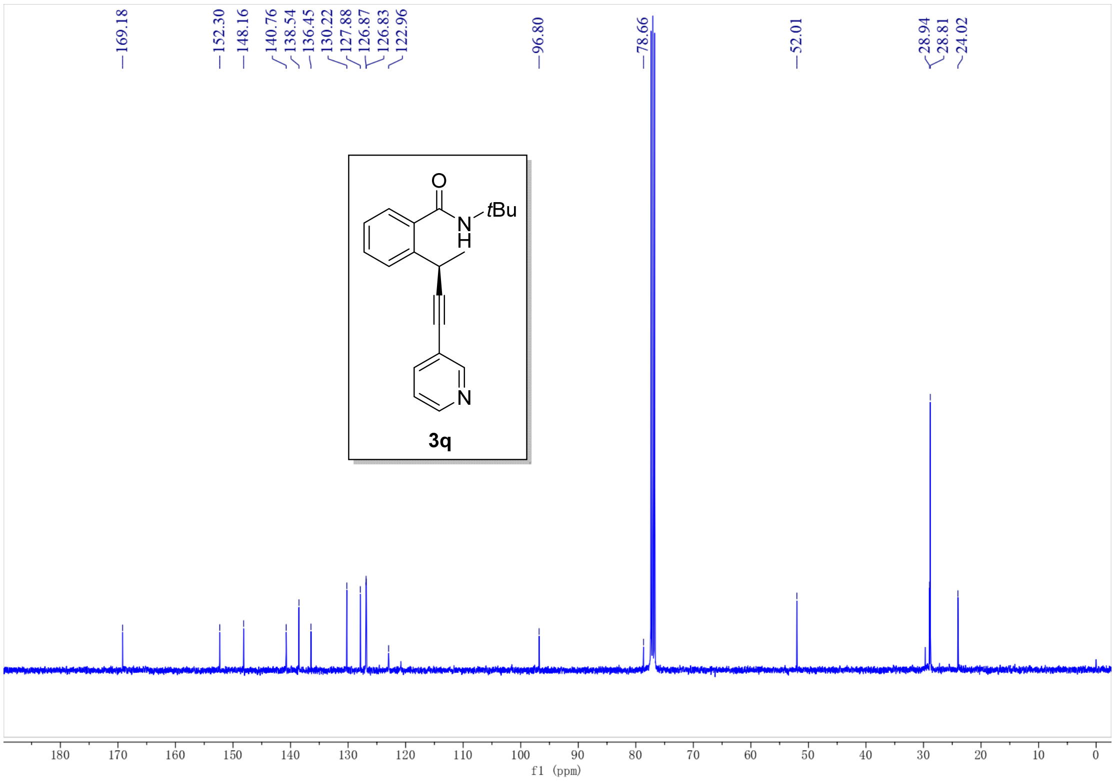

Supplementary Figure 103 ^{13}C NMR of **3q**

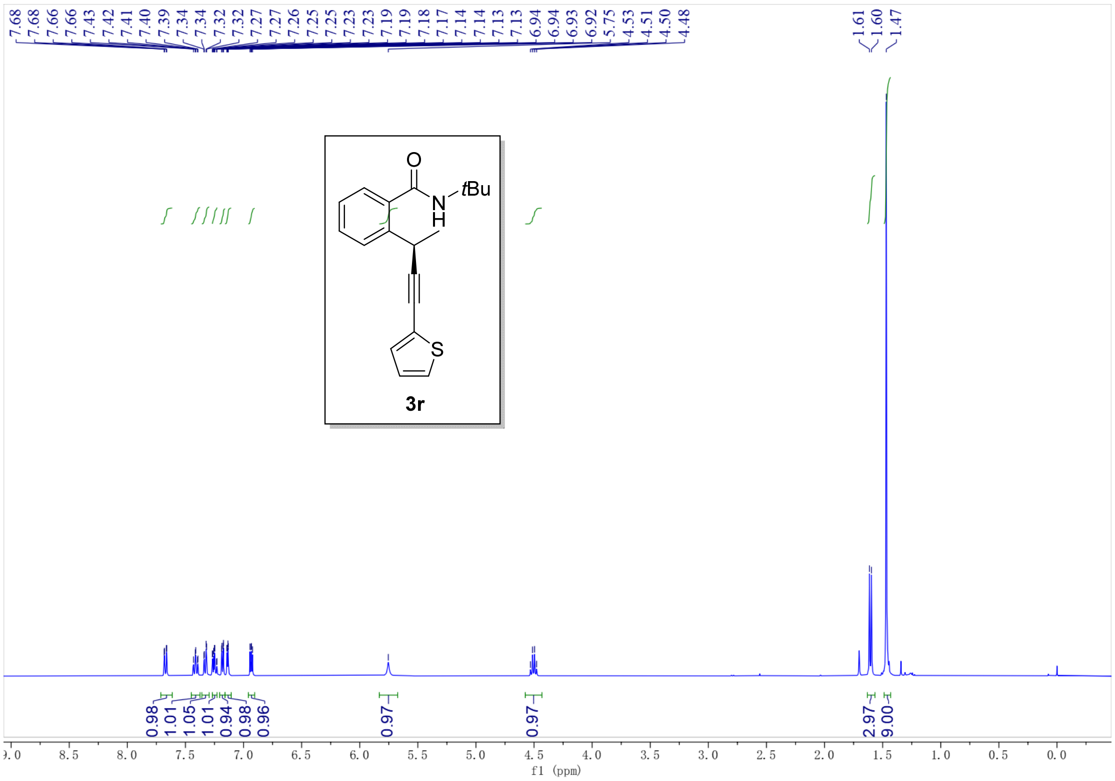

Supplementary Figure 104 ^{1}H NMR of **3r**

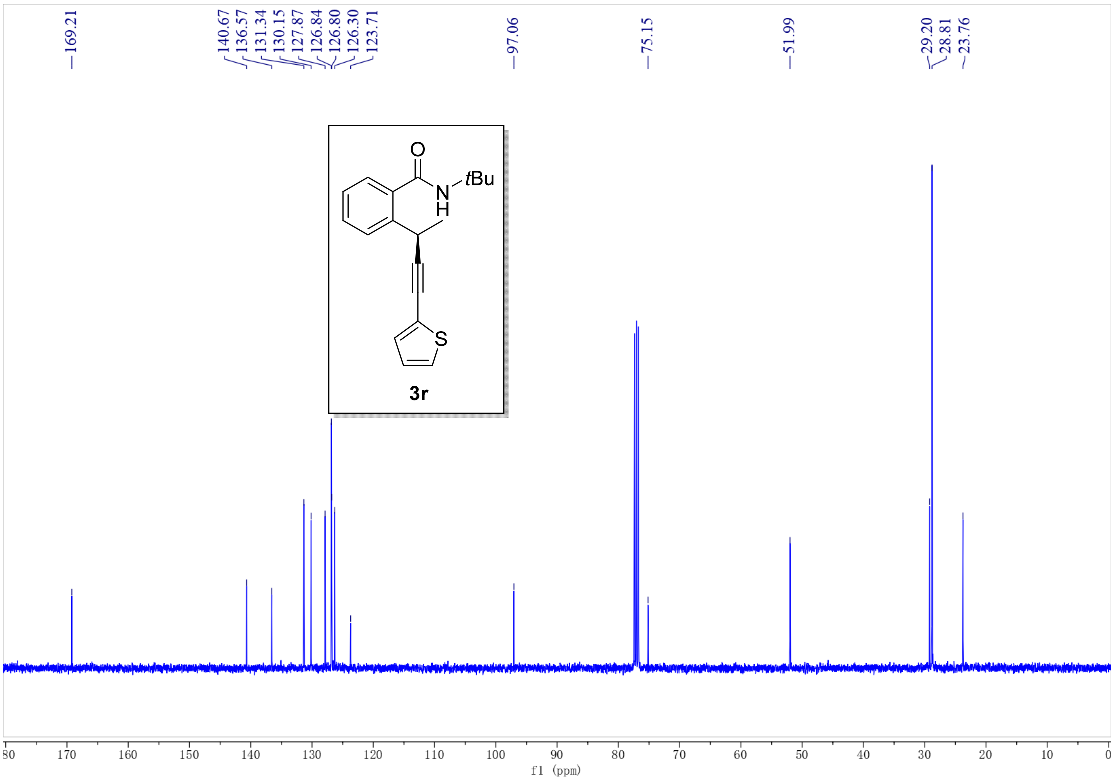

Supplementary Figure 105 ^{13}C NMR of **3r**

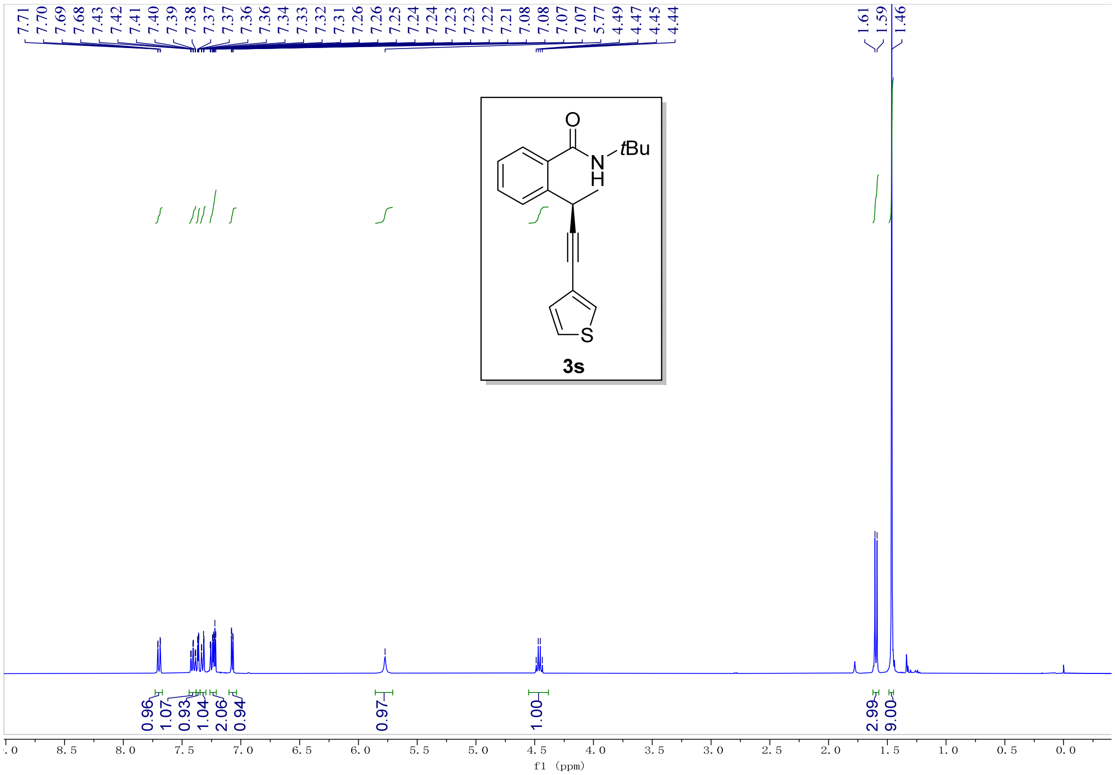

Supplementary Figure 106 ^{1}H NMR of **3s**

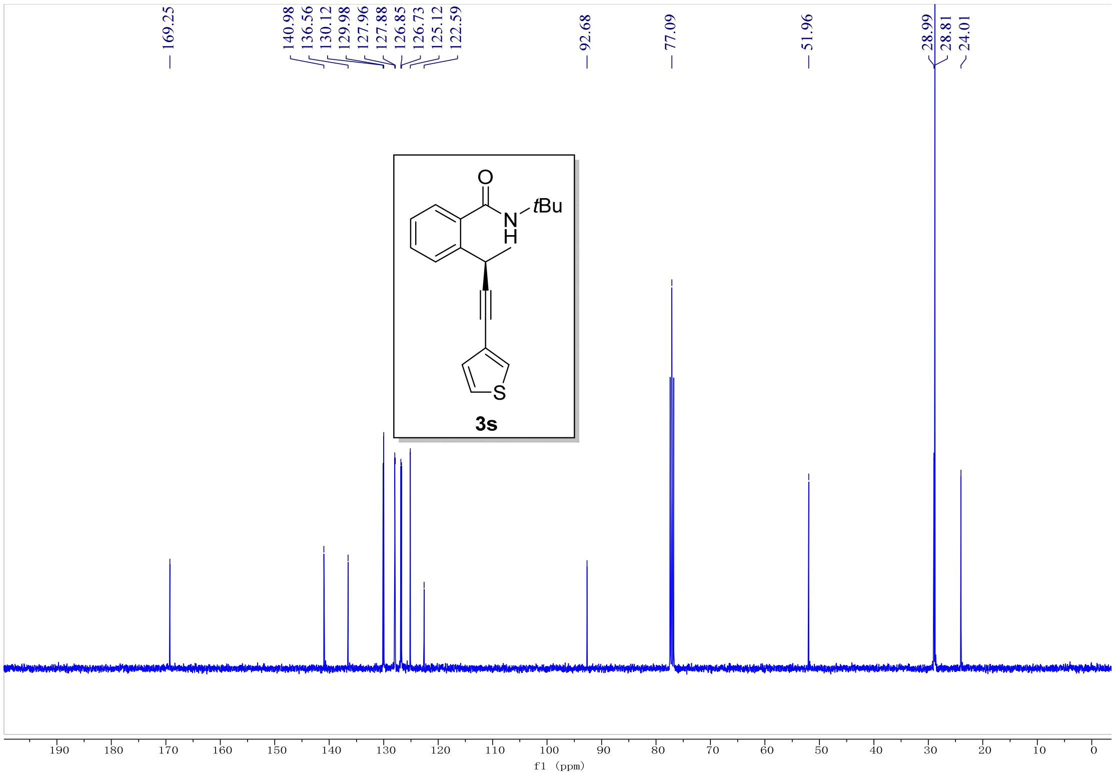

Supplementary Figure 107 ^{13}C NMR of **3s**

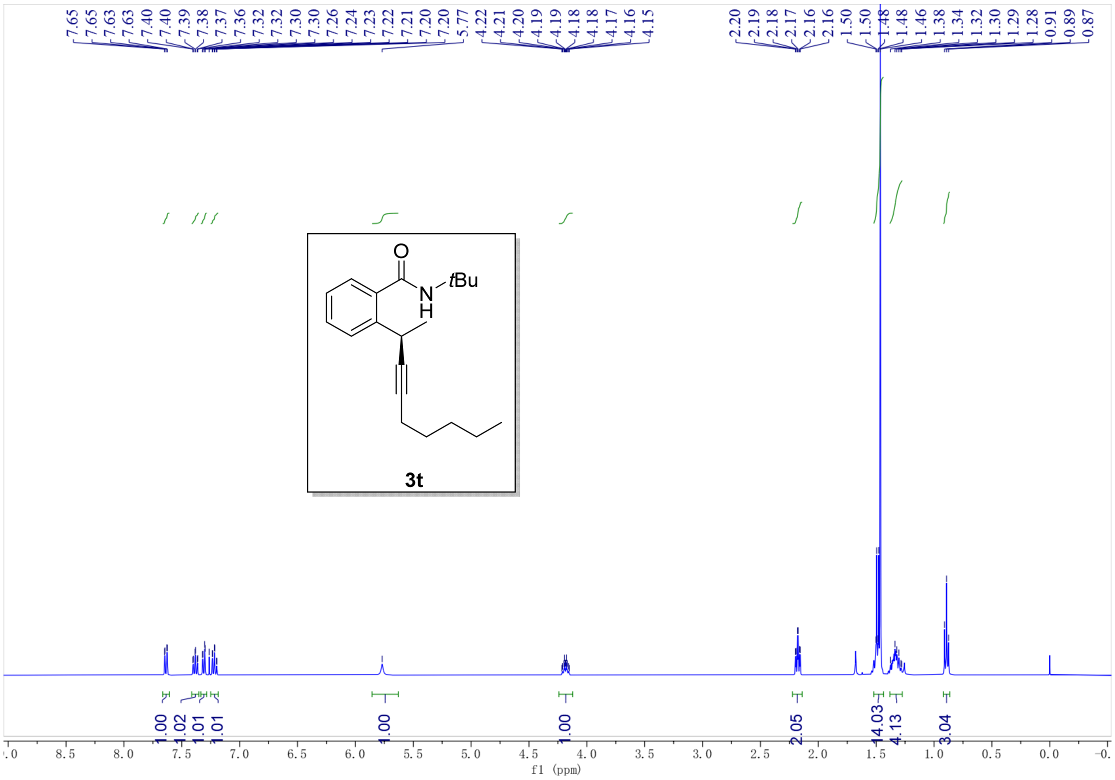

Supplementary Figure 108 ^{1}H NMR of **3t**

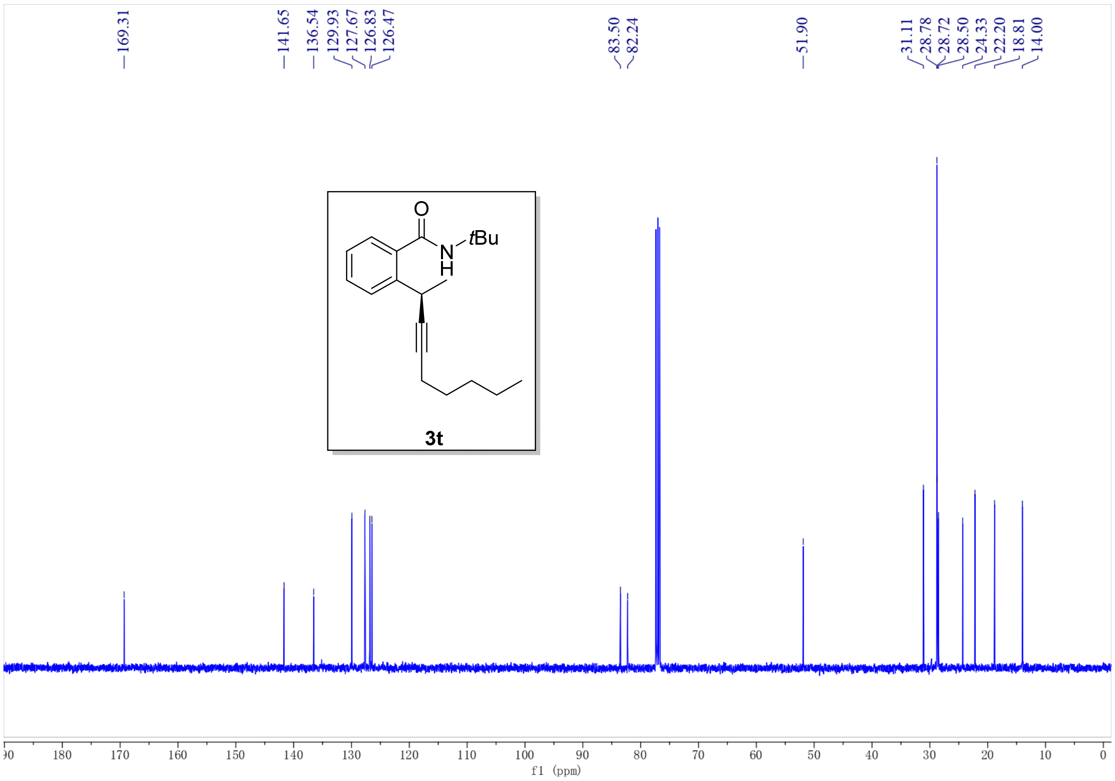

Supplementary Figure 109 ^{13}C NMR of **3t**

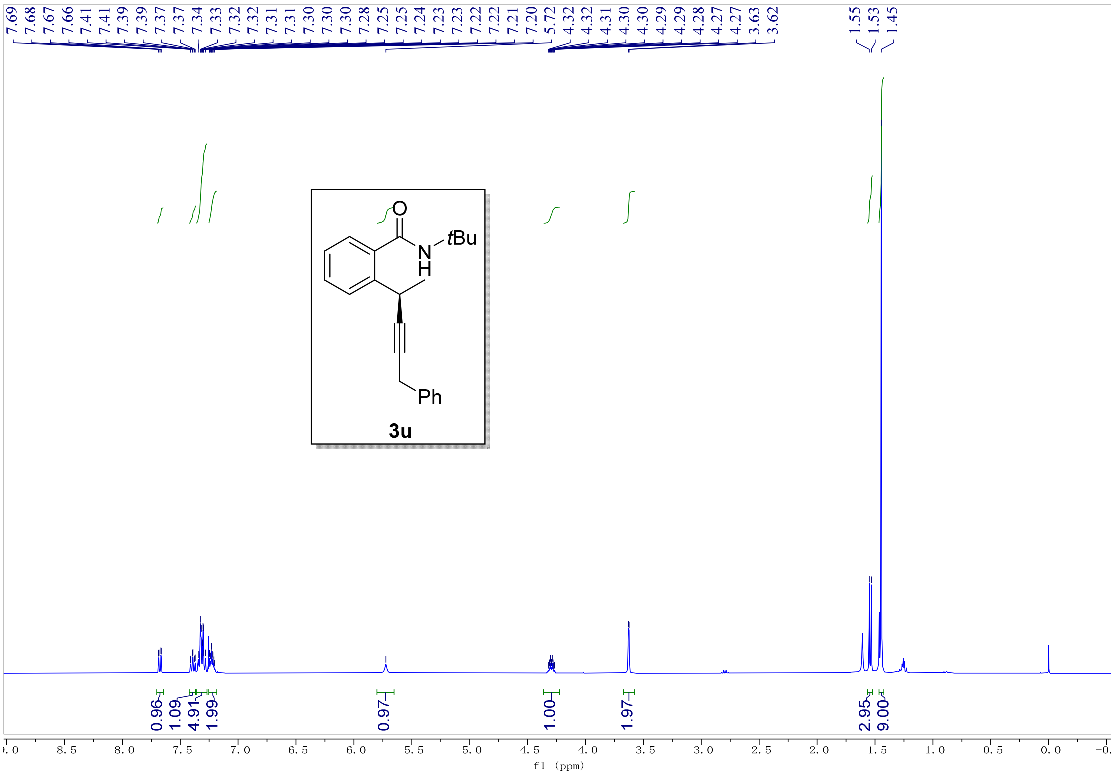

Supplementary Figure 110 ^{1}H NMR of **3u**

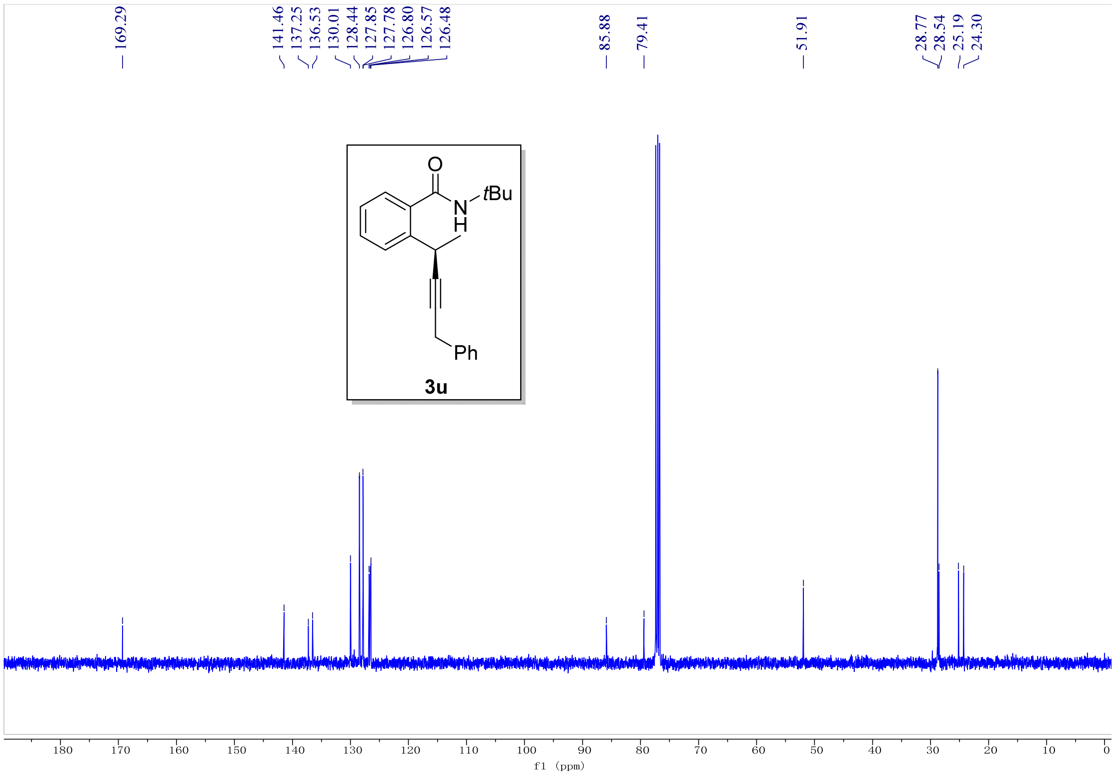

Supplementary Figure 111 ^{13}C NMR of **3u**

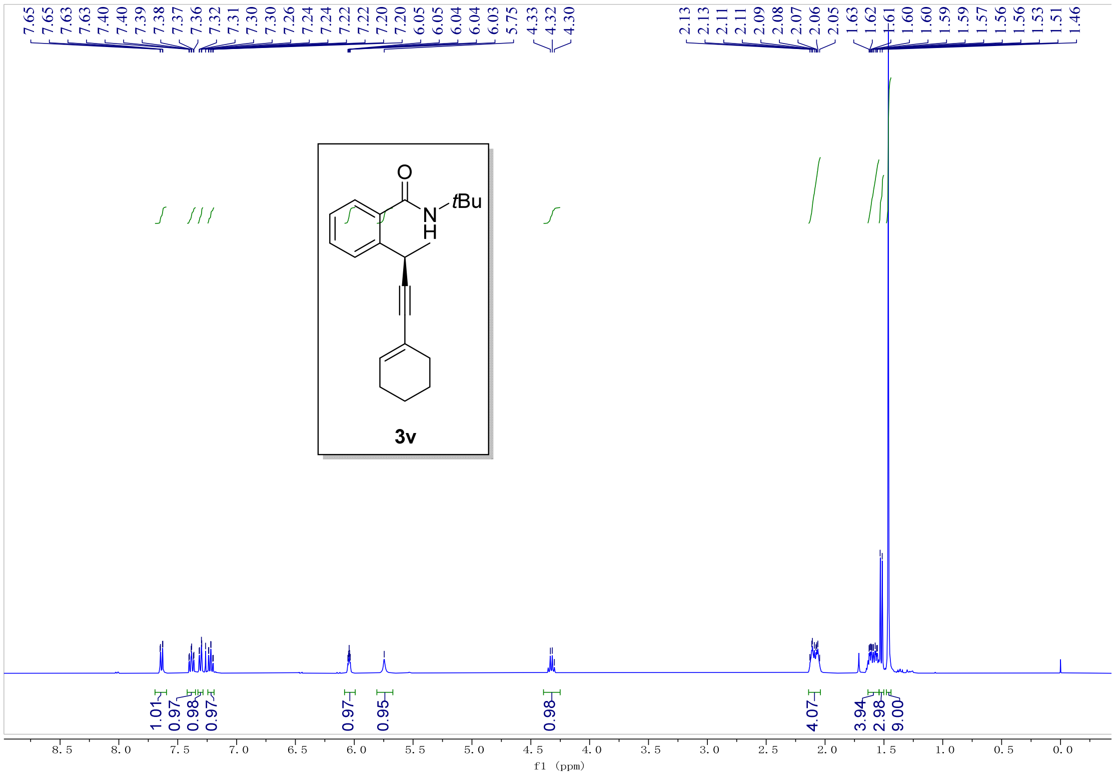

Supplementary Figure 112 ^{1}H NMR of **3v**

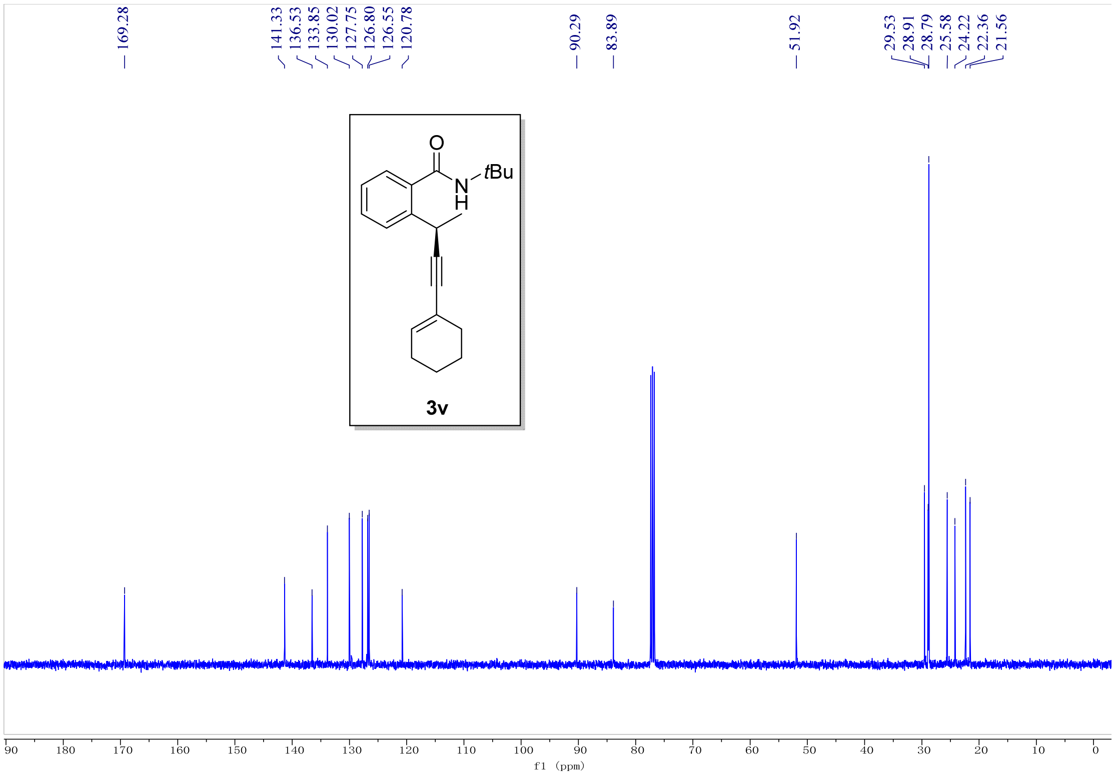

Supplementary Figure 113 ^{13}C NMR of **3v**

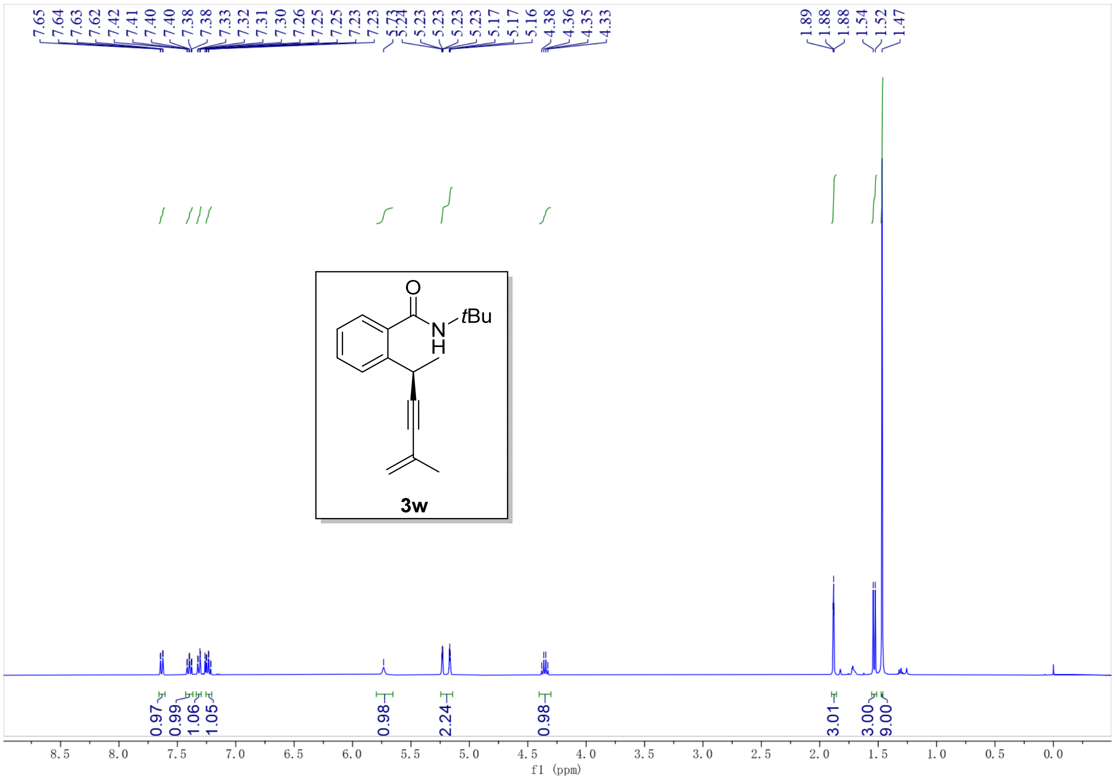

Supplementary Figure 114 ^{1}H NMR of **3w**

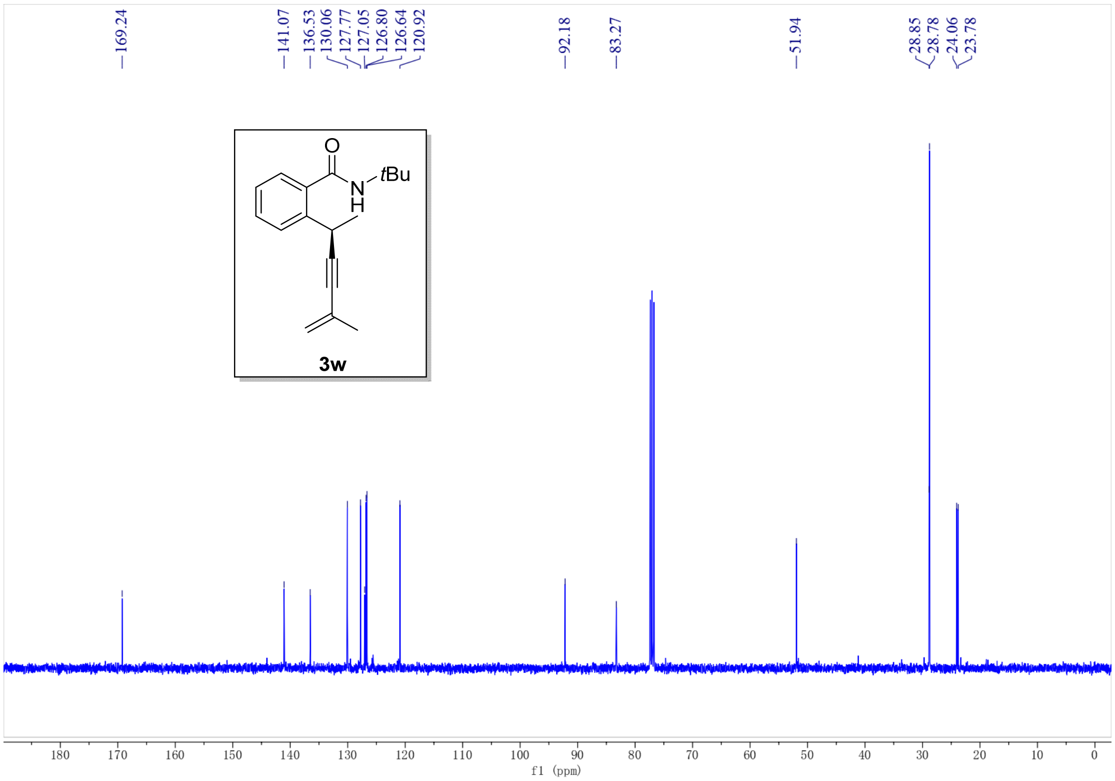

Supplementary Figure 115 ^{13}C NMR of **3w**

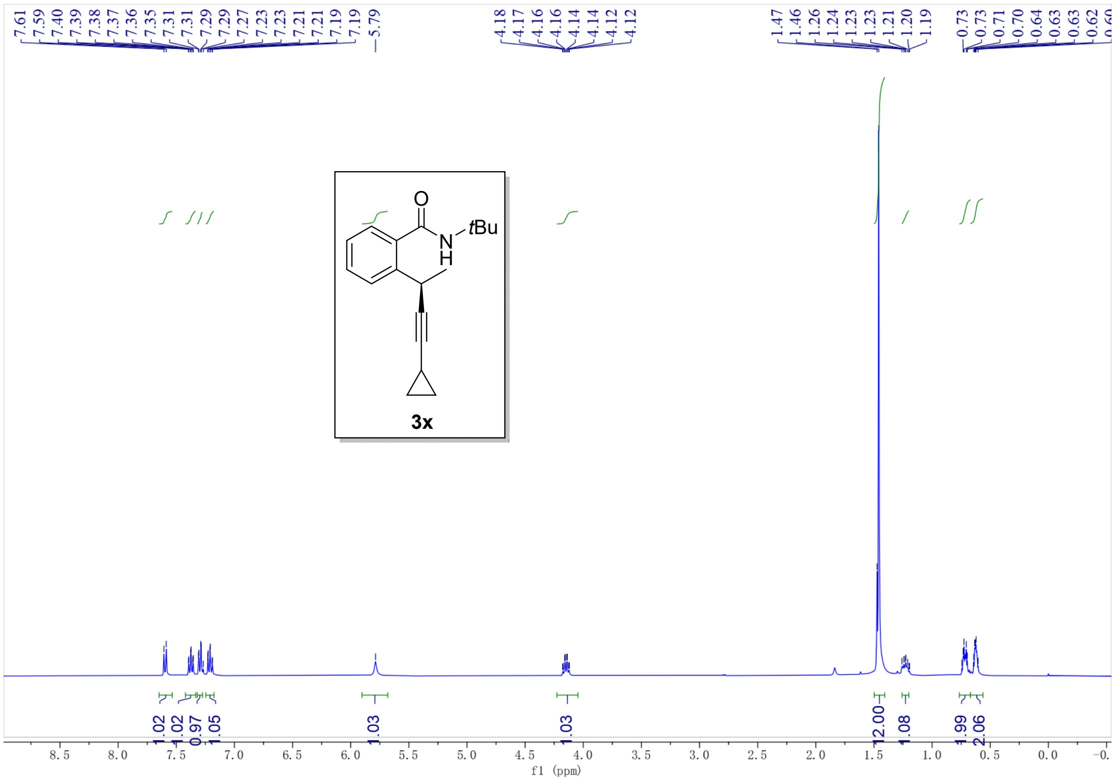

Supplementary Figure 116 ^{1}H NMR of **3x**

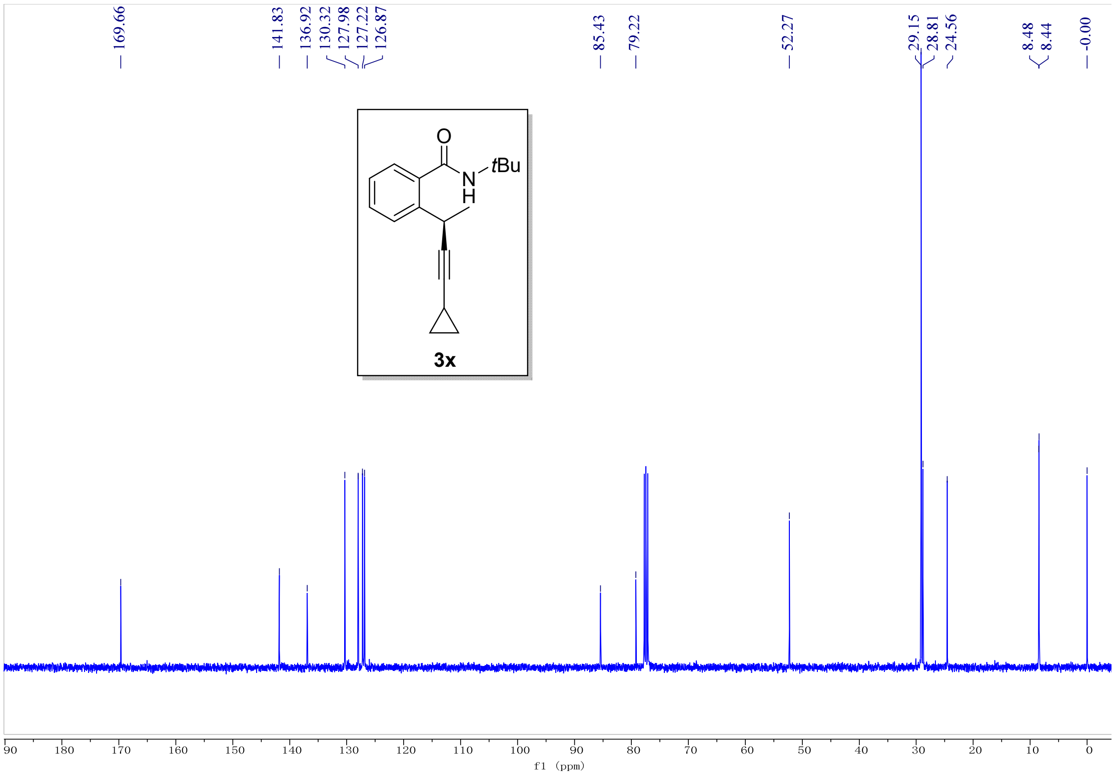

Supplementary Figure 117 ^{13}C NMR of **3x**

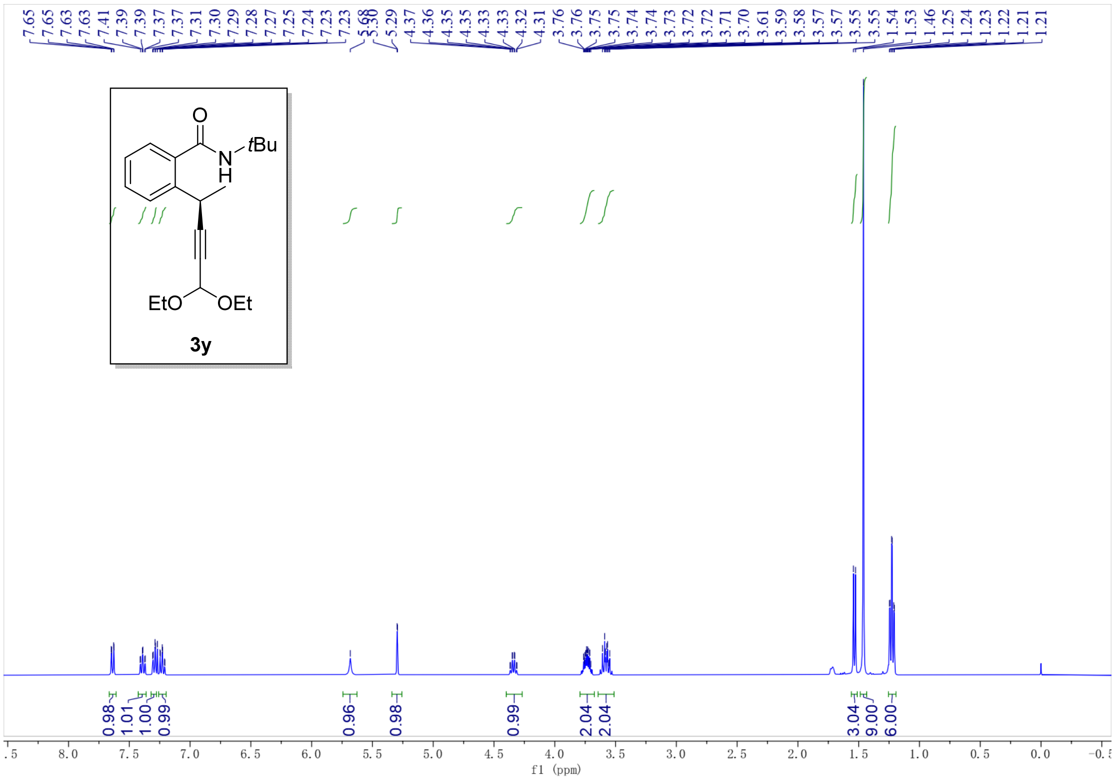

Supplementary Figure 118 ^{1}H NMR of **3y**

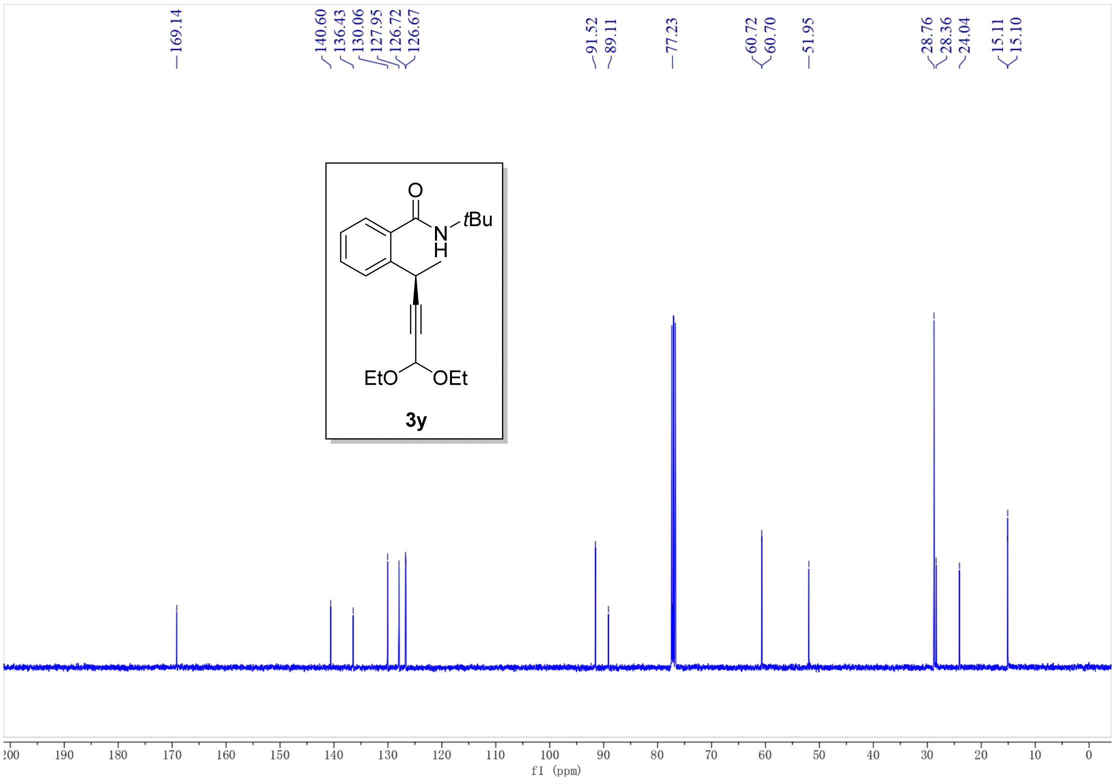

Supplementary Figure 119 ^{13}C NMR of **3y**

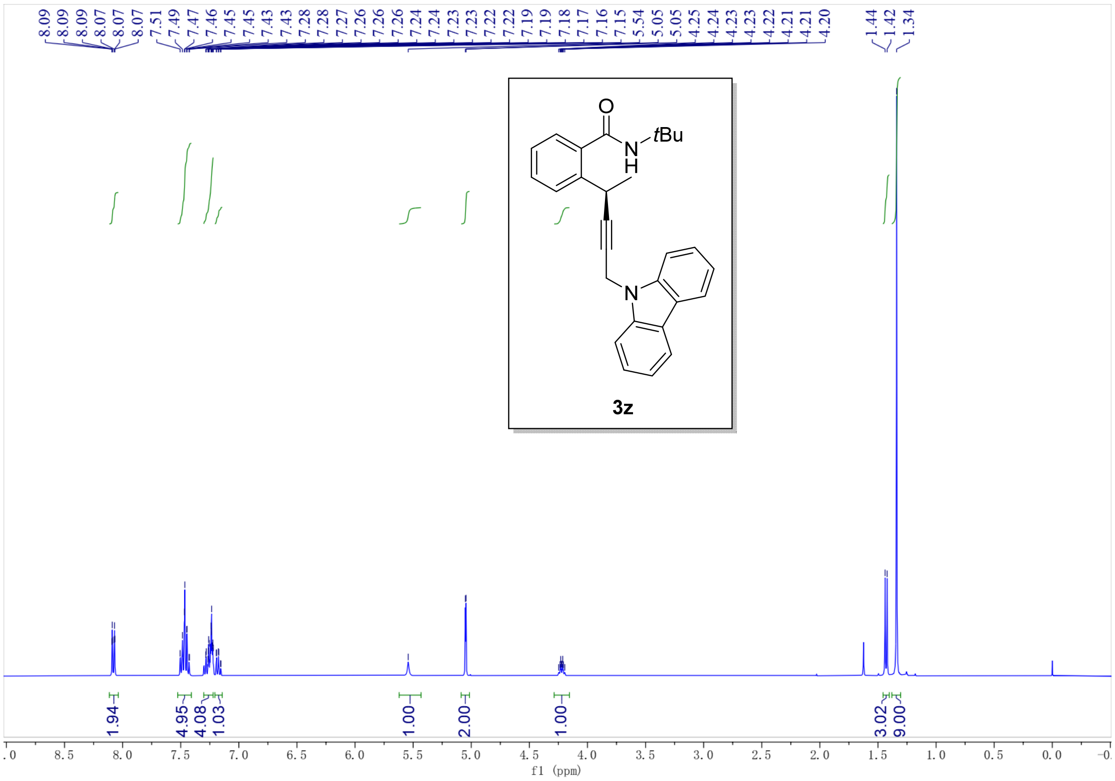

Supplementary Figure 120 ^{1}H NMR of **3z**

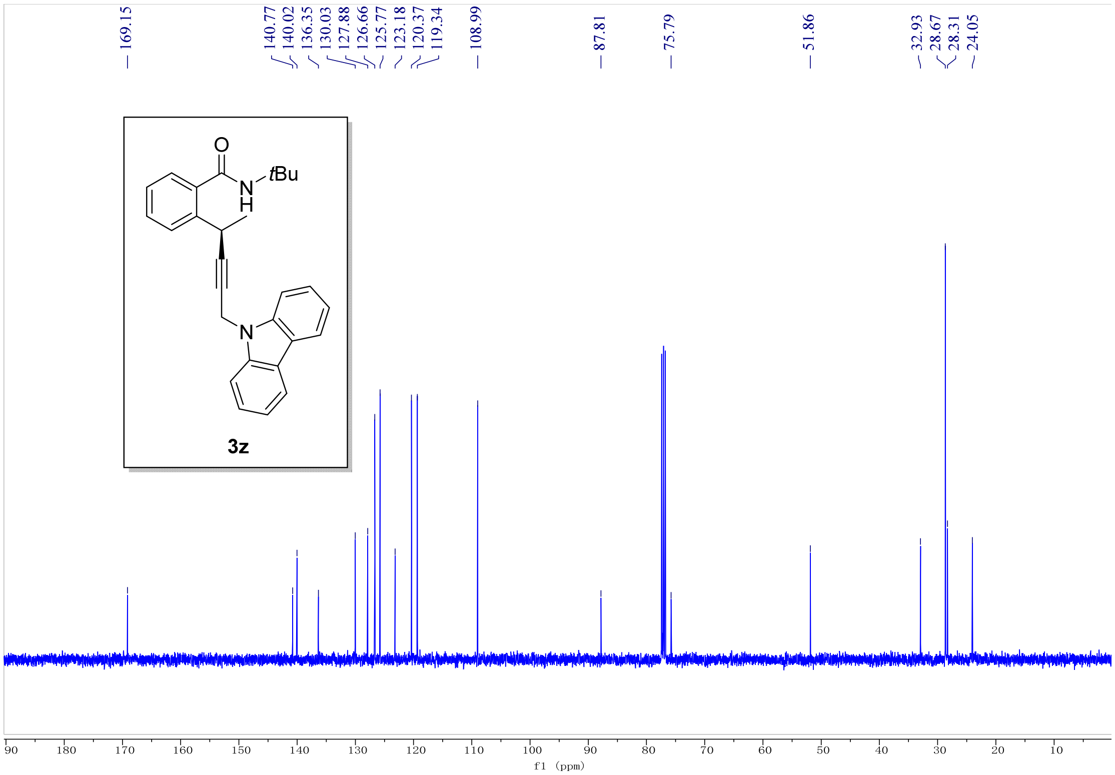

Supplementary Figure 121 ^{13}C NMR of **3z**

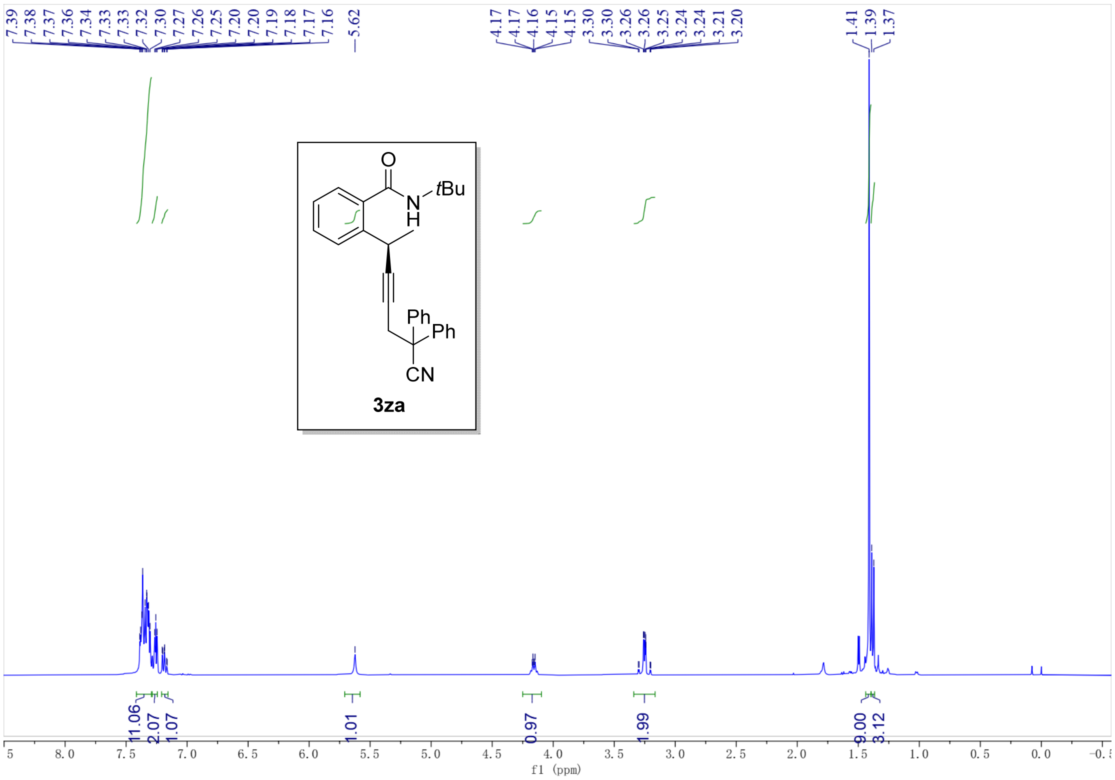

Supplementary Figure 122 ^{1}H NMR of **3za**

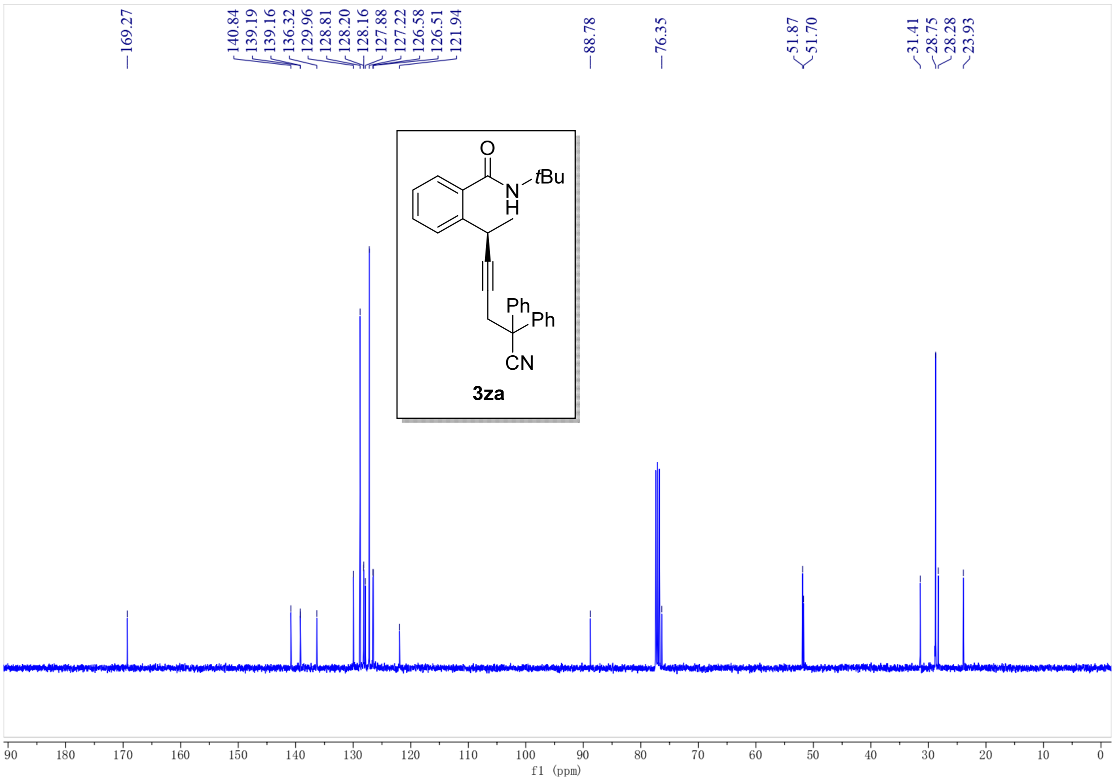

Supplementary Figure 123 ^{13}C NMR of **3za**

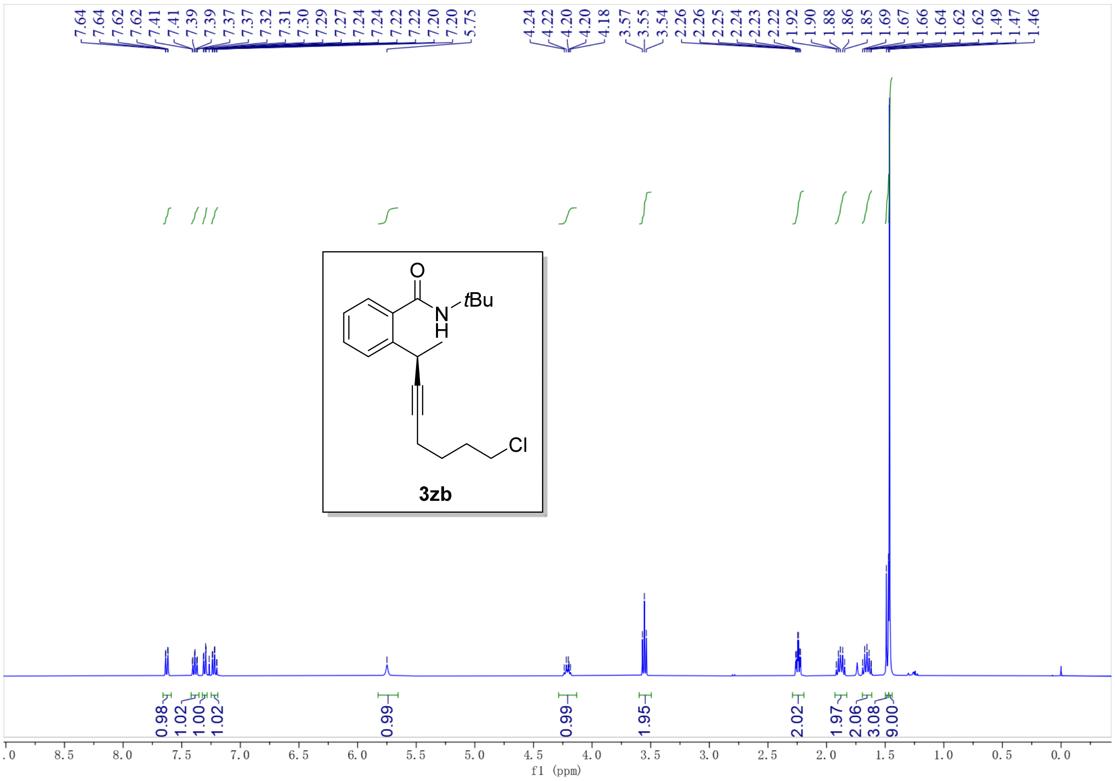

Supplementary Figure 124 ^{1}H NMR of **3zb**

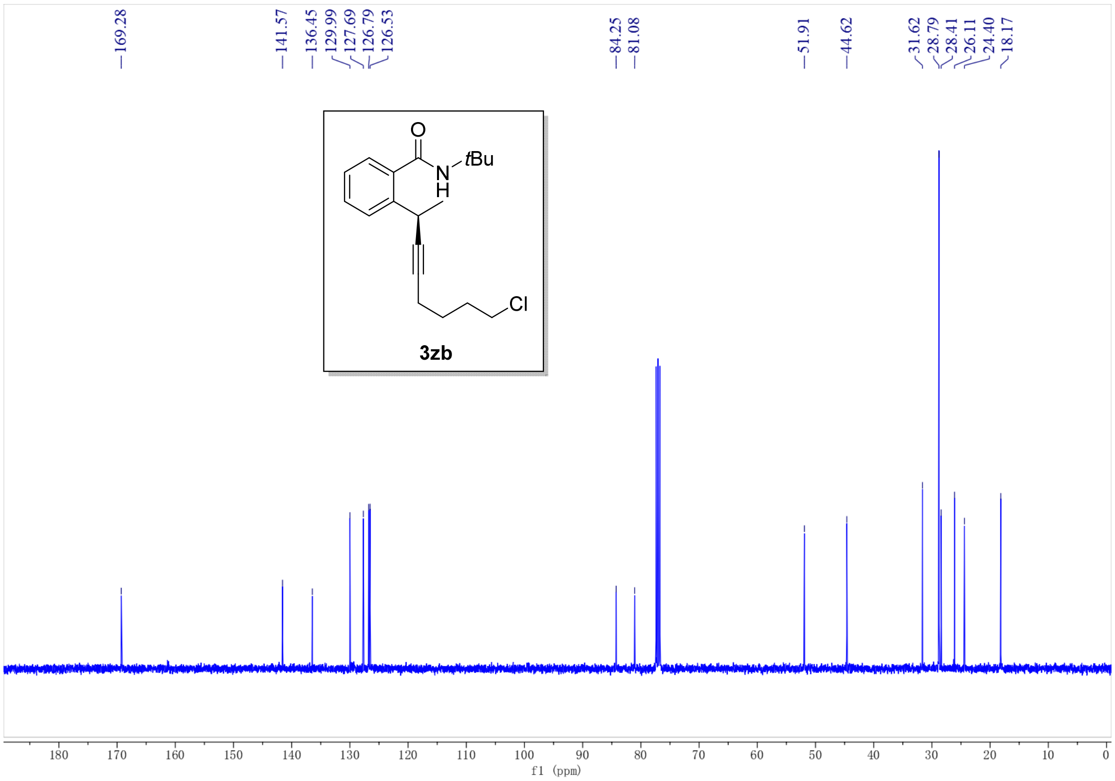

Supplementary Figure 125 ^{13}C NMR of **3zb**

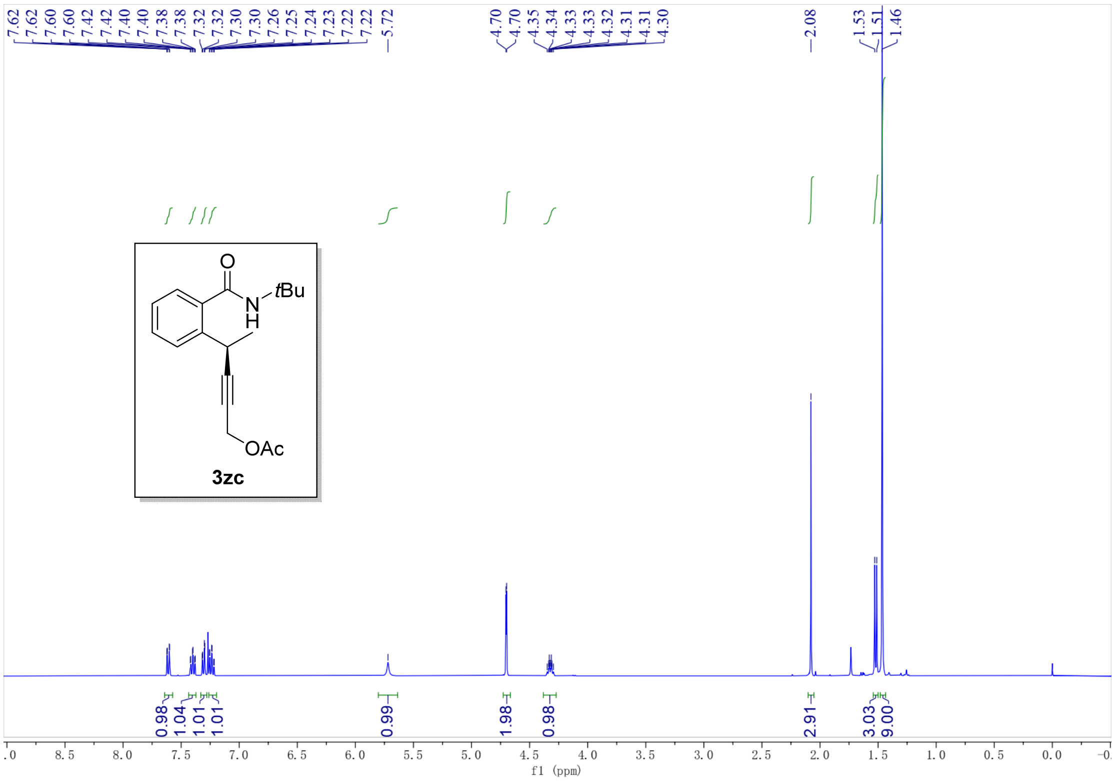

Supplementary Figure 126 ^{1}H NMR of **3zc**

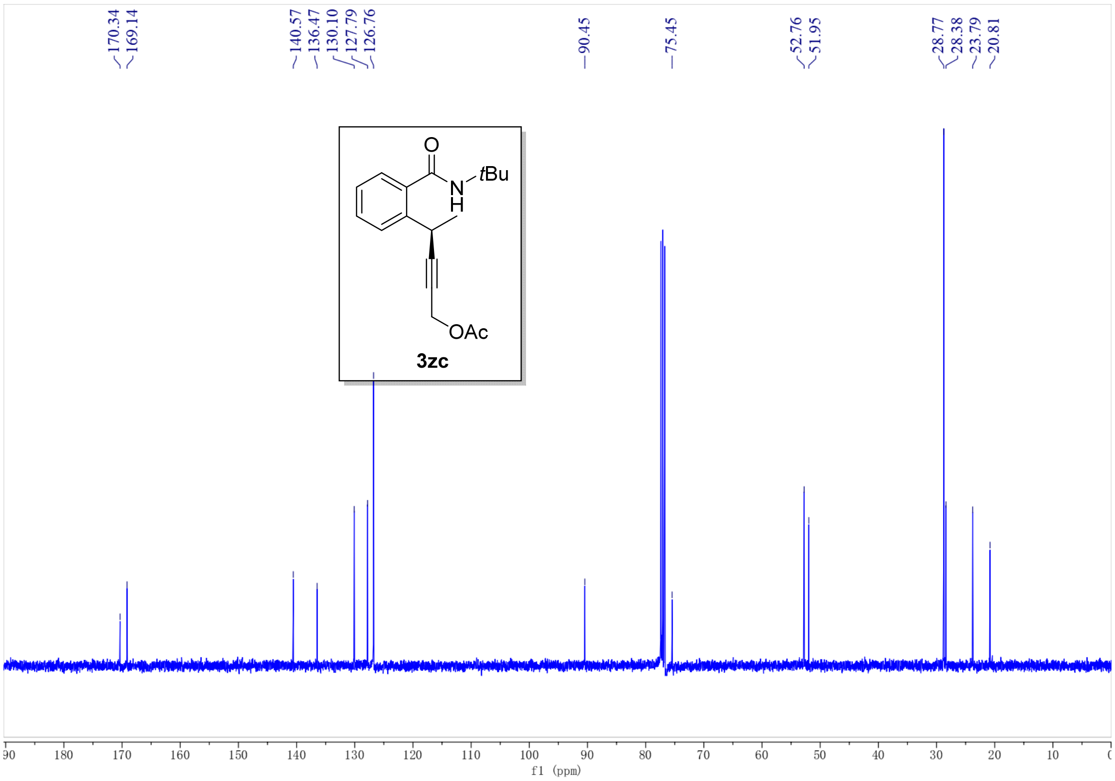

Supplementary Figure 127 ^{13}C NMR of **3zc**

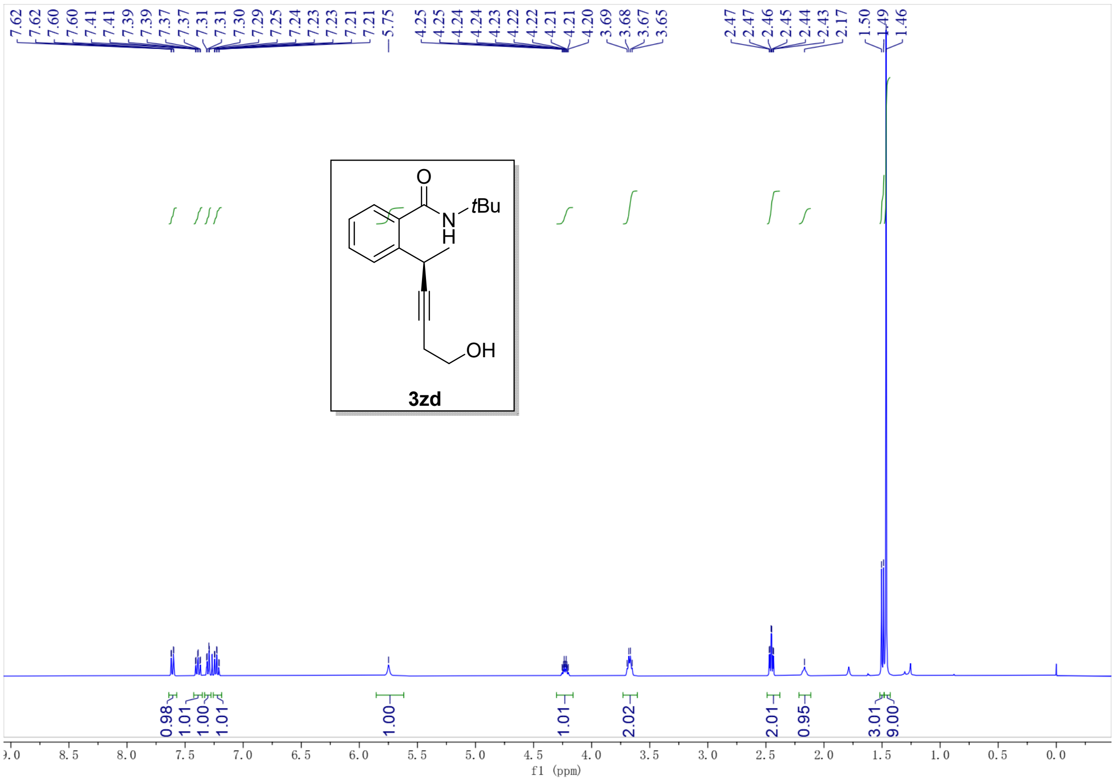

Supplementary Figure 128 ^{1}H NMR of **3zd**

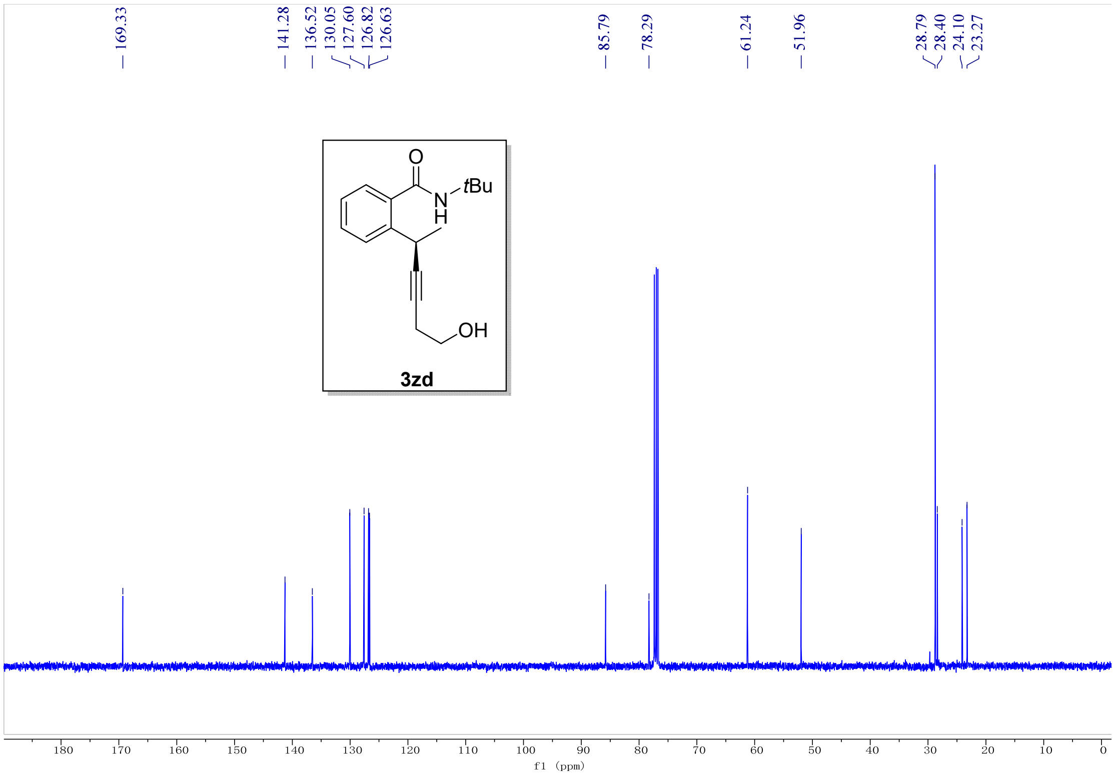

Supplementary Figure 129 ^{13}C NMR of **3zd**

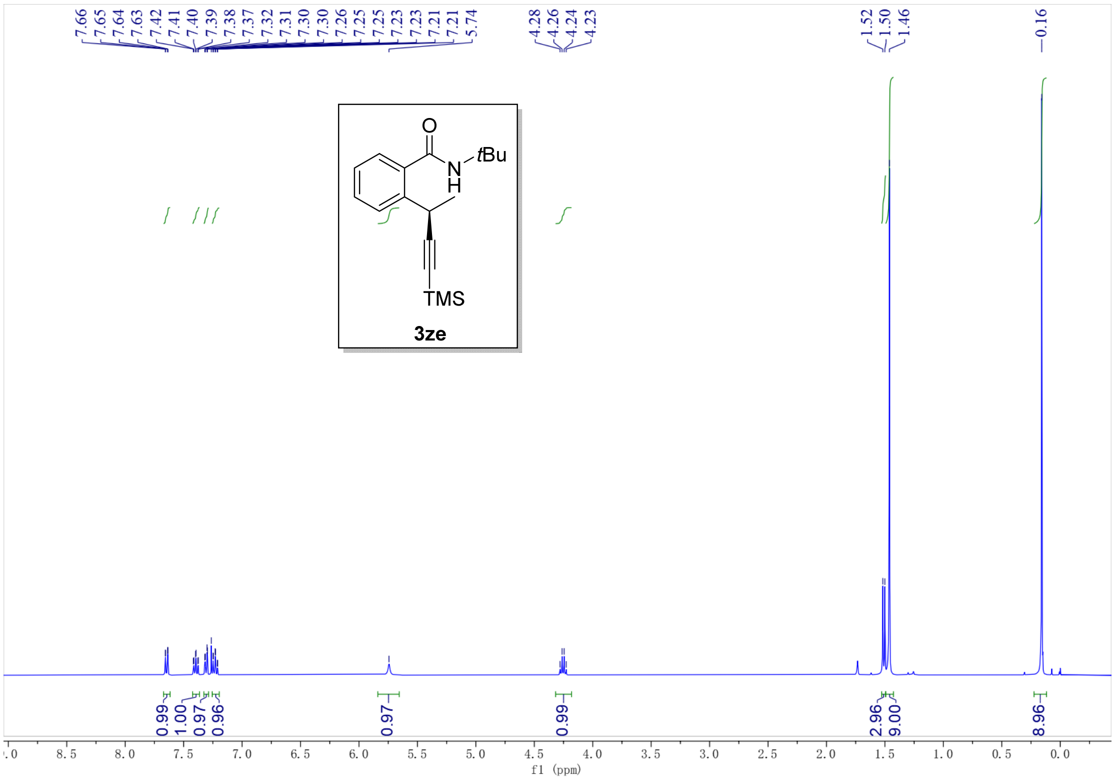

Supplementary Figure 130 ^{1}H NMR of **3ze**

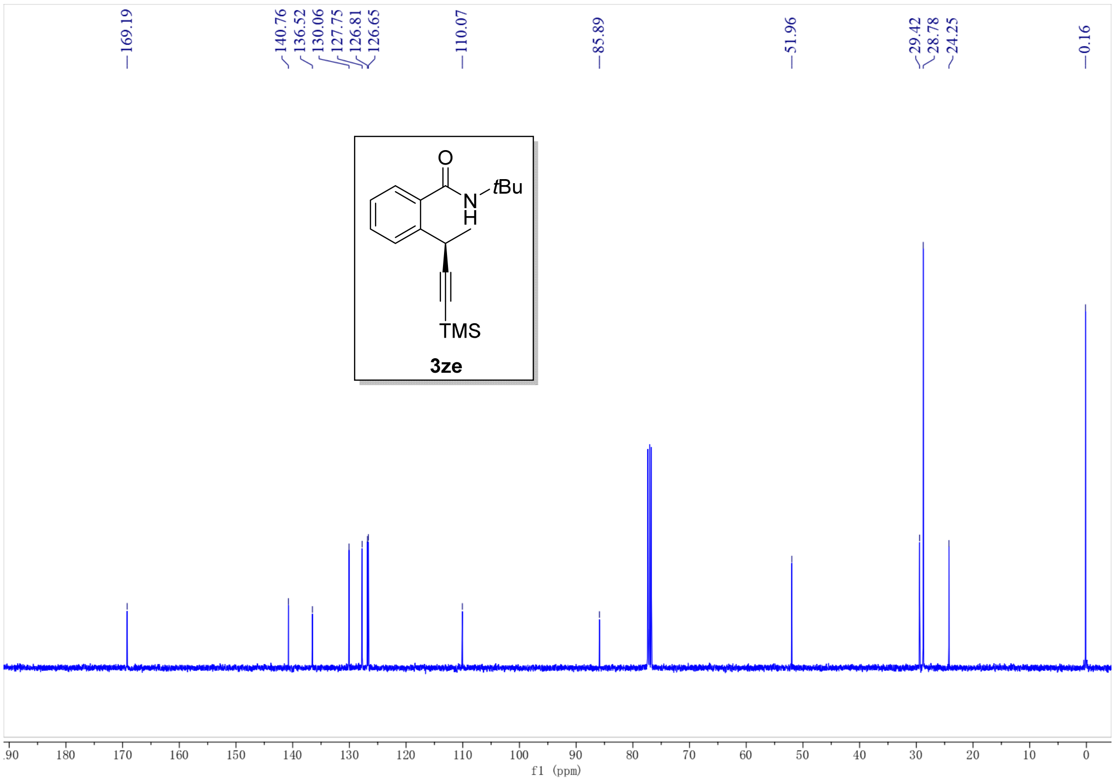

Supplementary Figure 131 ^{13}C NMR of **3ze**

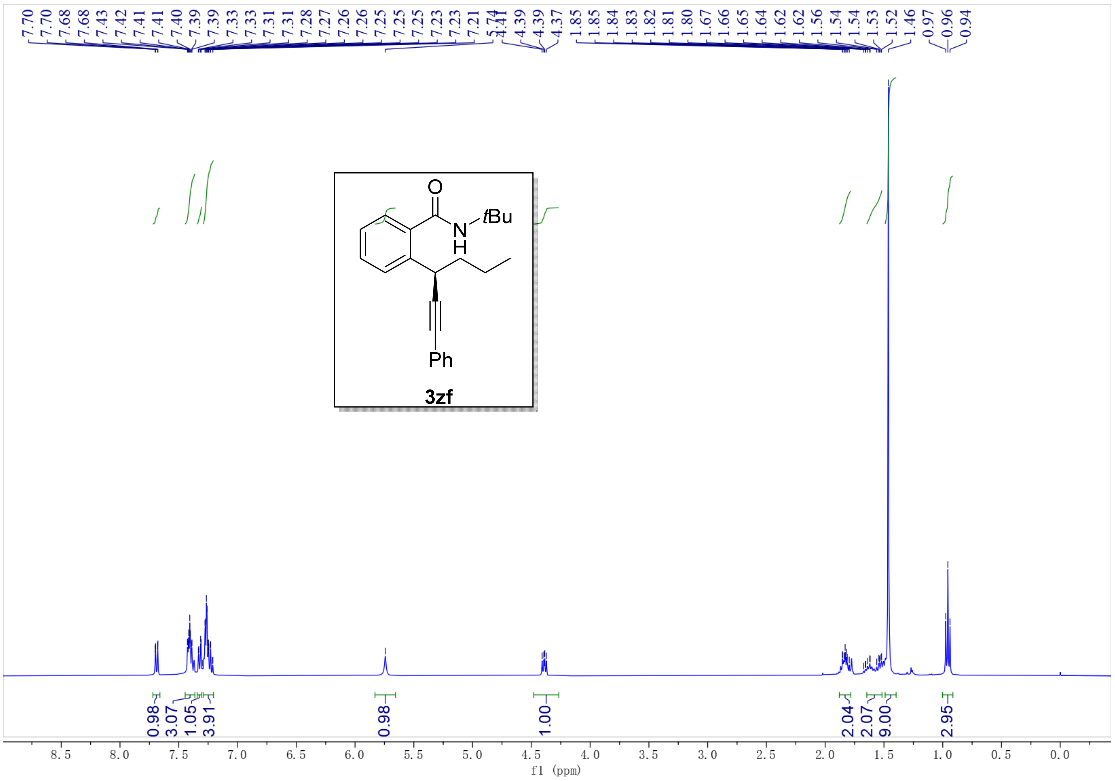

Supplementary Figure 132 ^{1}H NMR of **3zf**

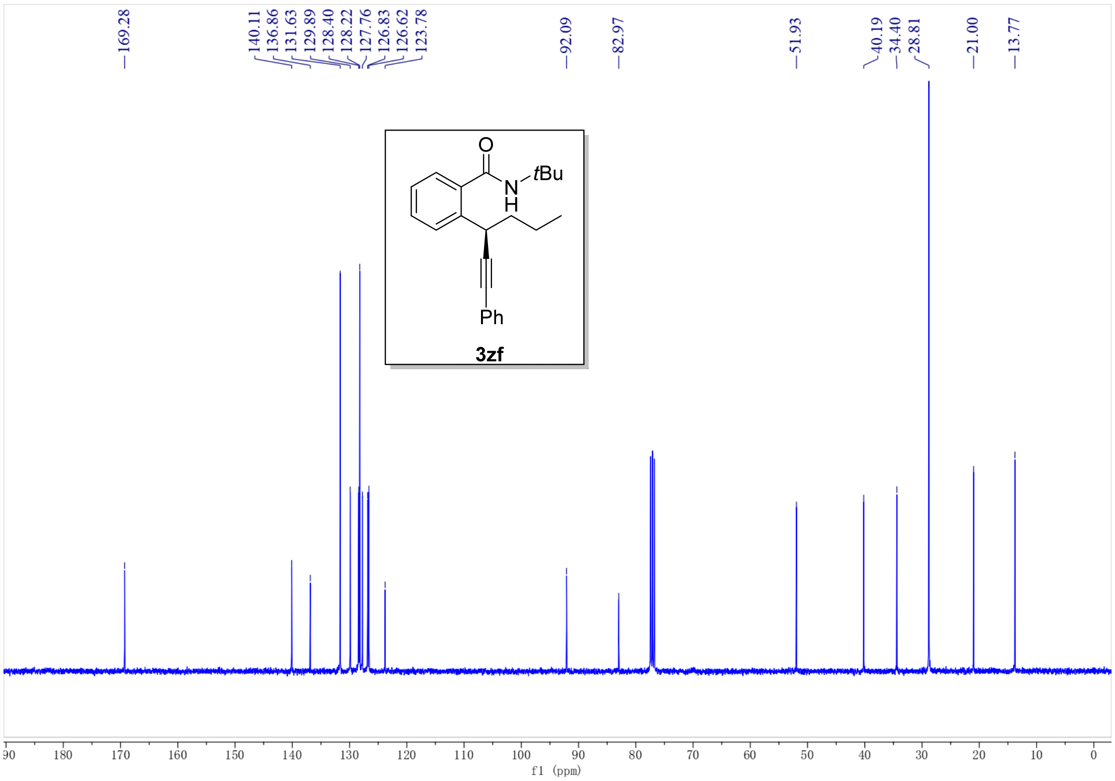

Supplementary Figure 133 ^{13}C NMR of **3zf**

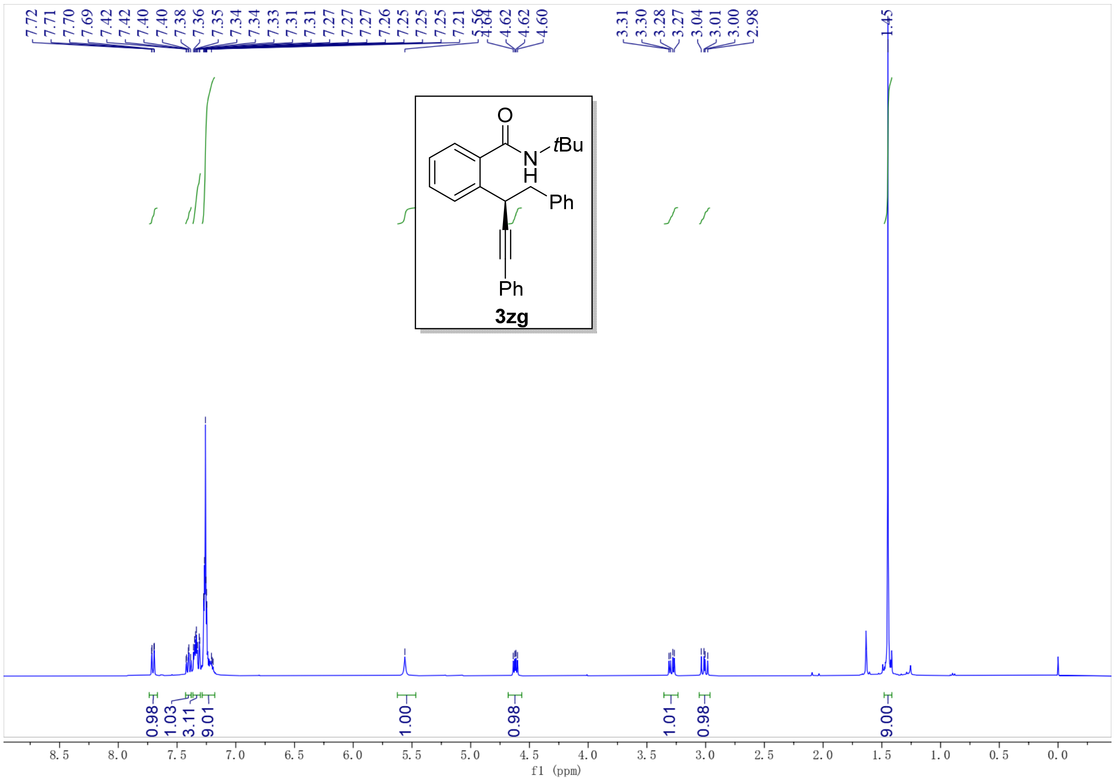

Supplementary Figure 134 ^{1}H NMR of **3zg**

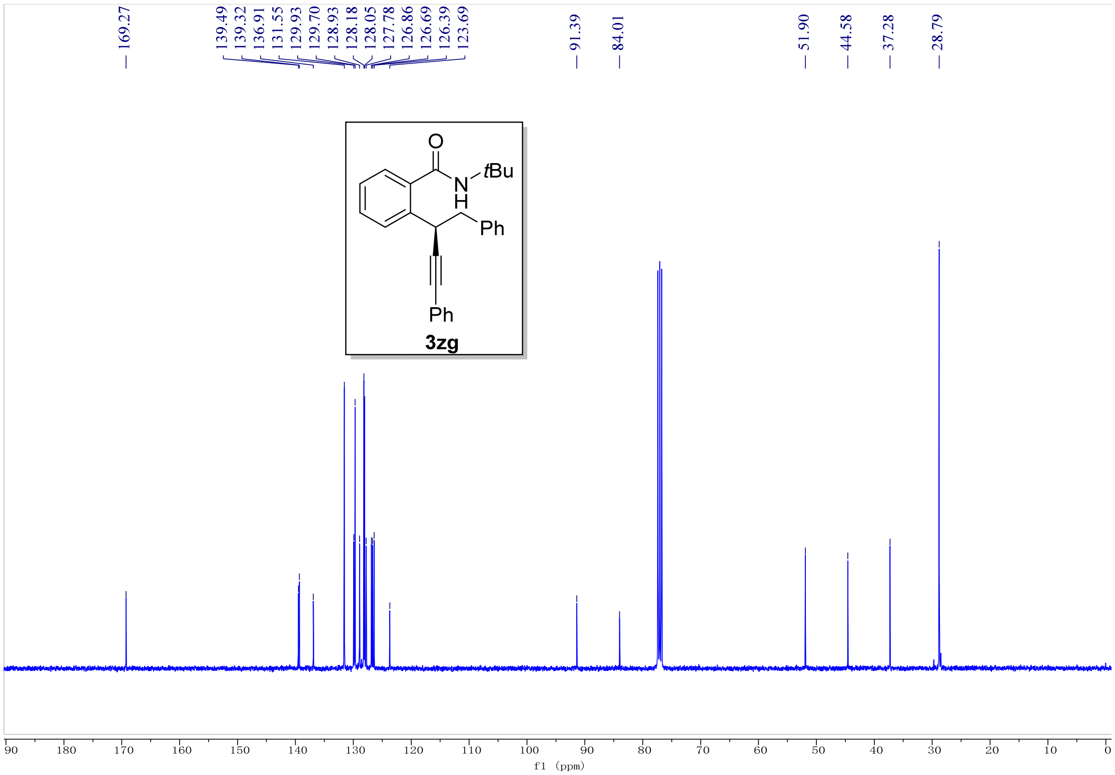

Supplementary Figure 135 ^{13}C NMR of **3zg**

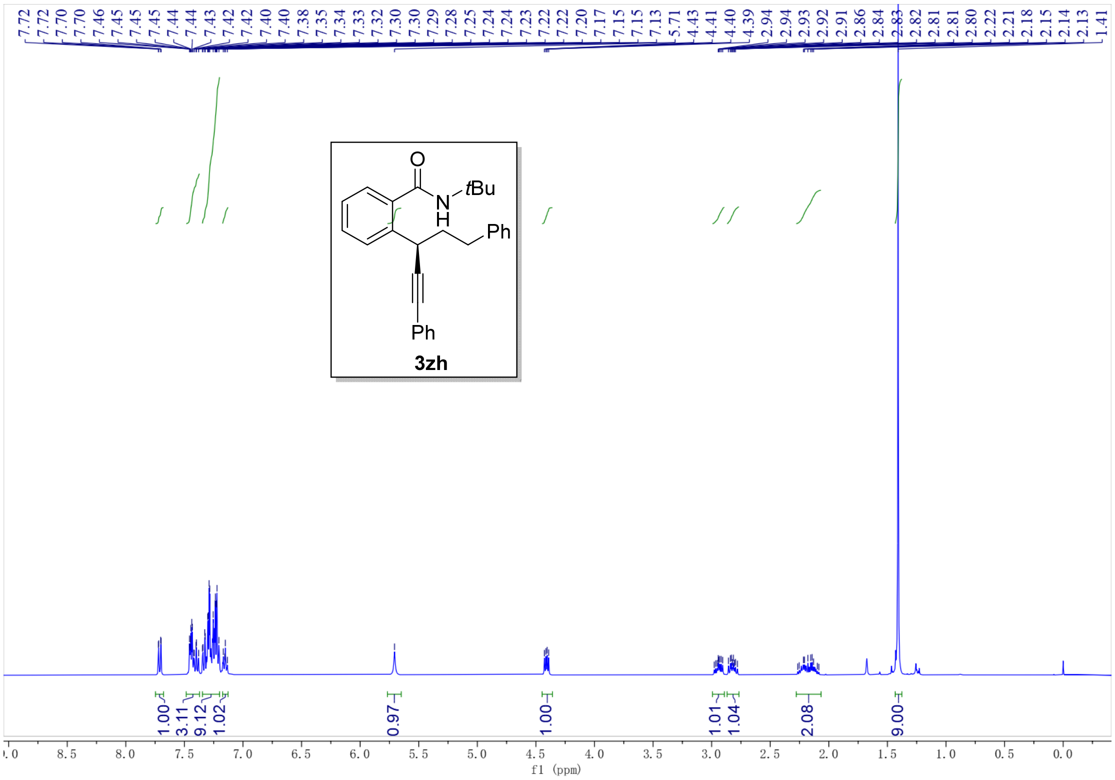

Supplementary Figure 136 ^{1}H NMR of **3zh**

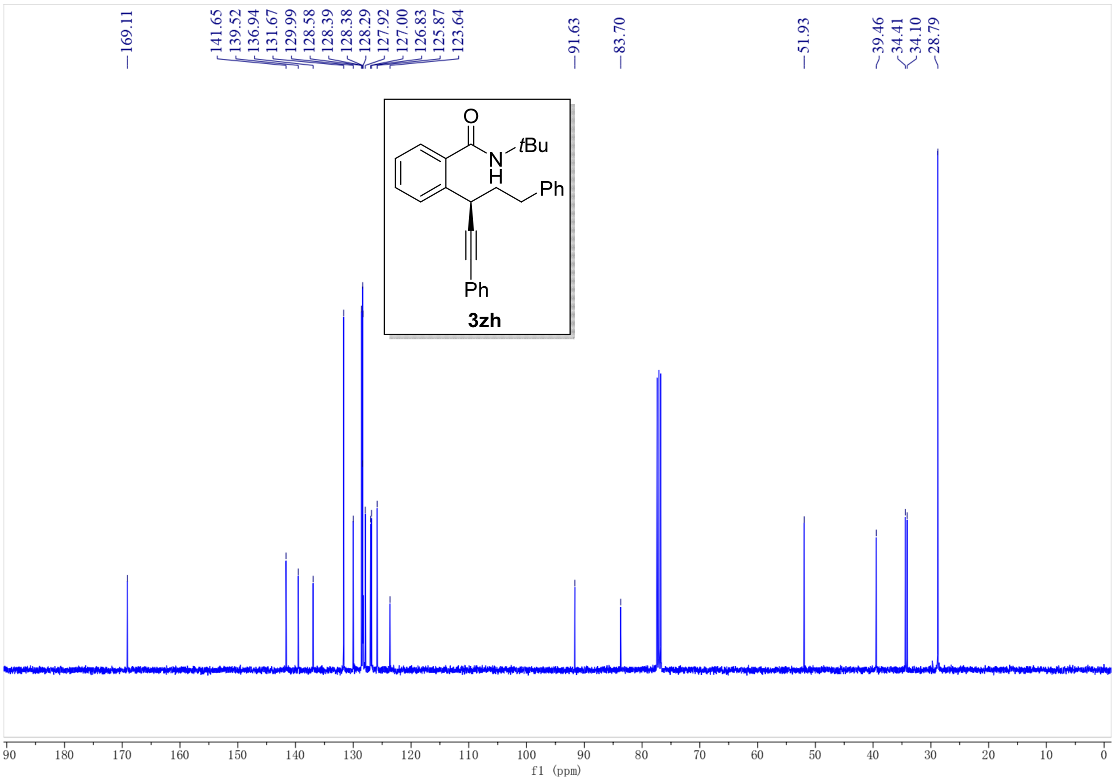

Supplementary Figure 137 ^{13}C NMR of **3zh**

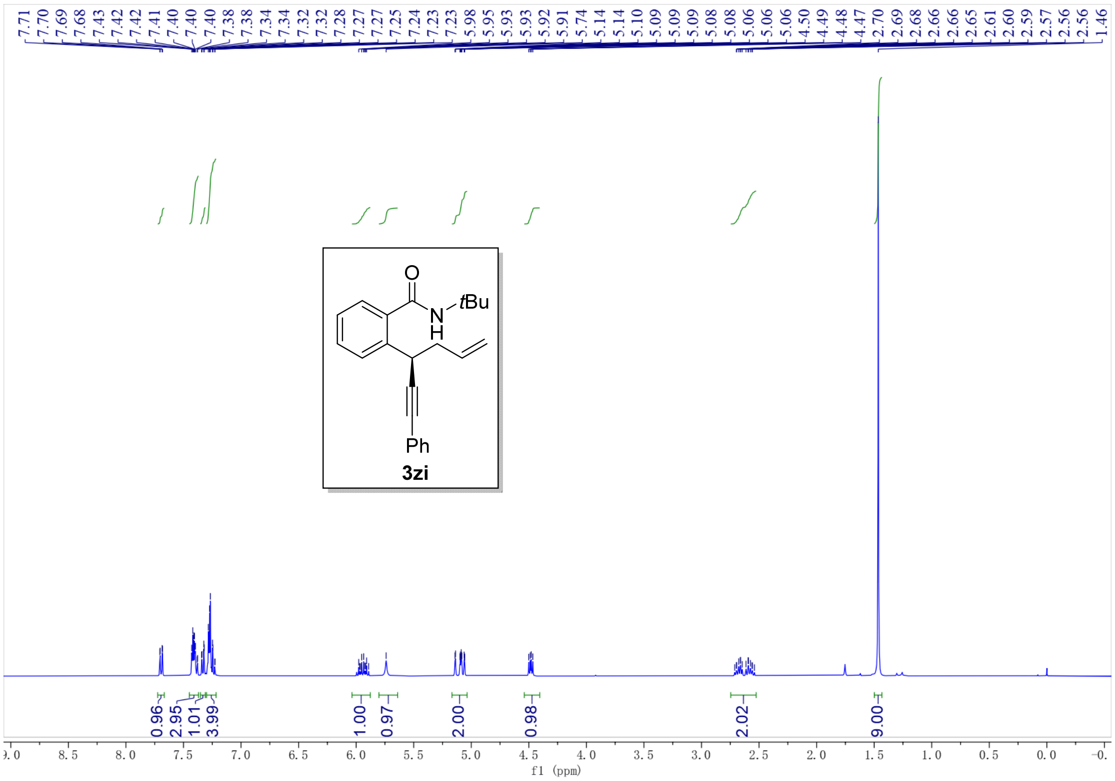

Supplementary Figure 138 ^{1}H NMR of **3zi**

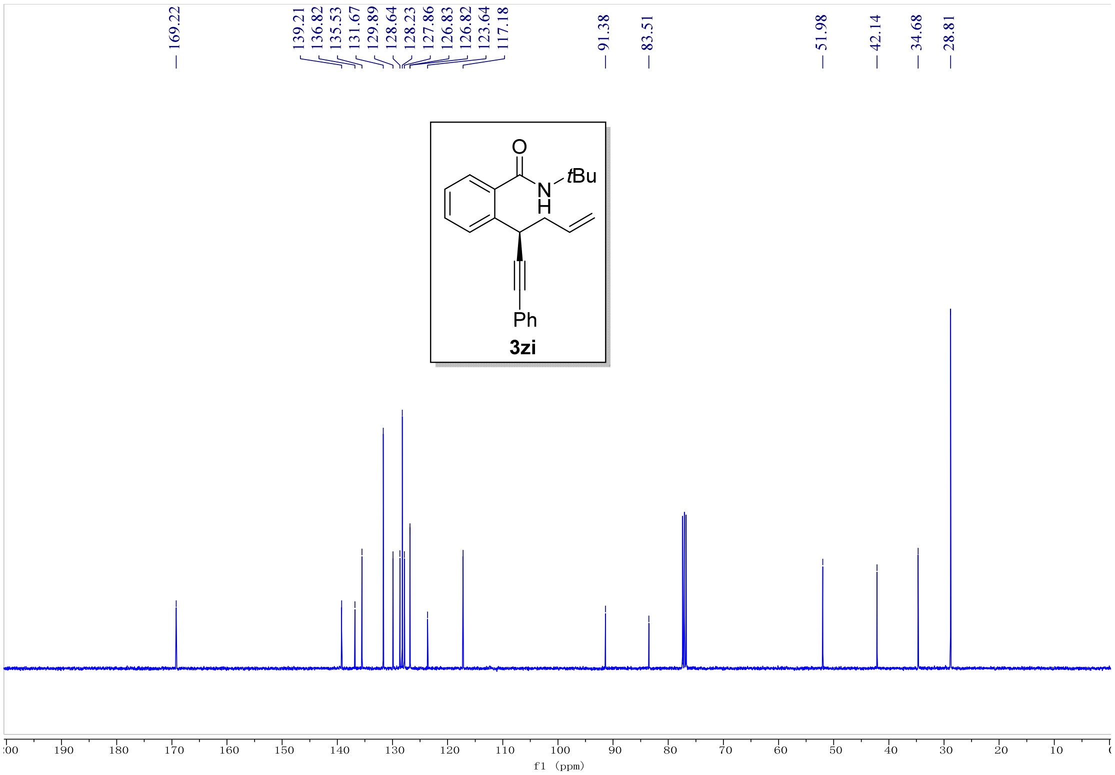

Supplementary Figure 139 ^{13}C NMR of **3zi**

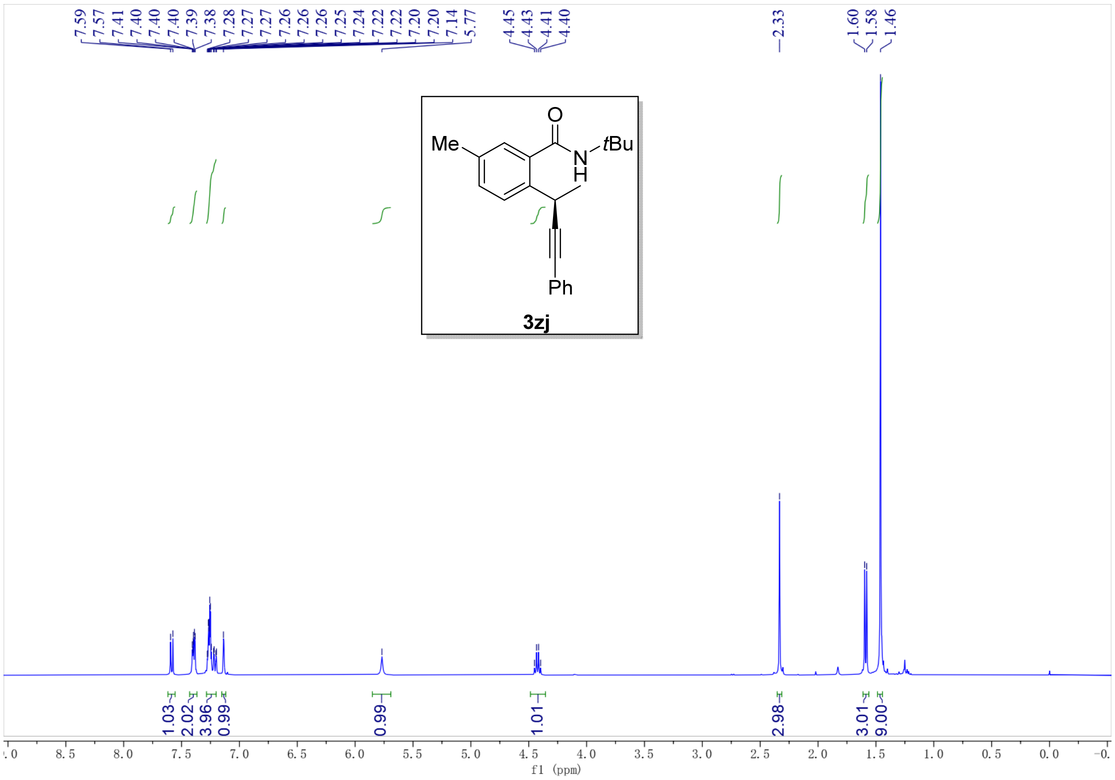

Supplementary Figure 140 ^{1}H NMR of **3zj**

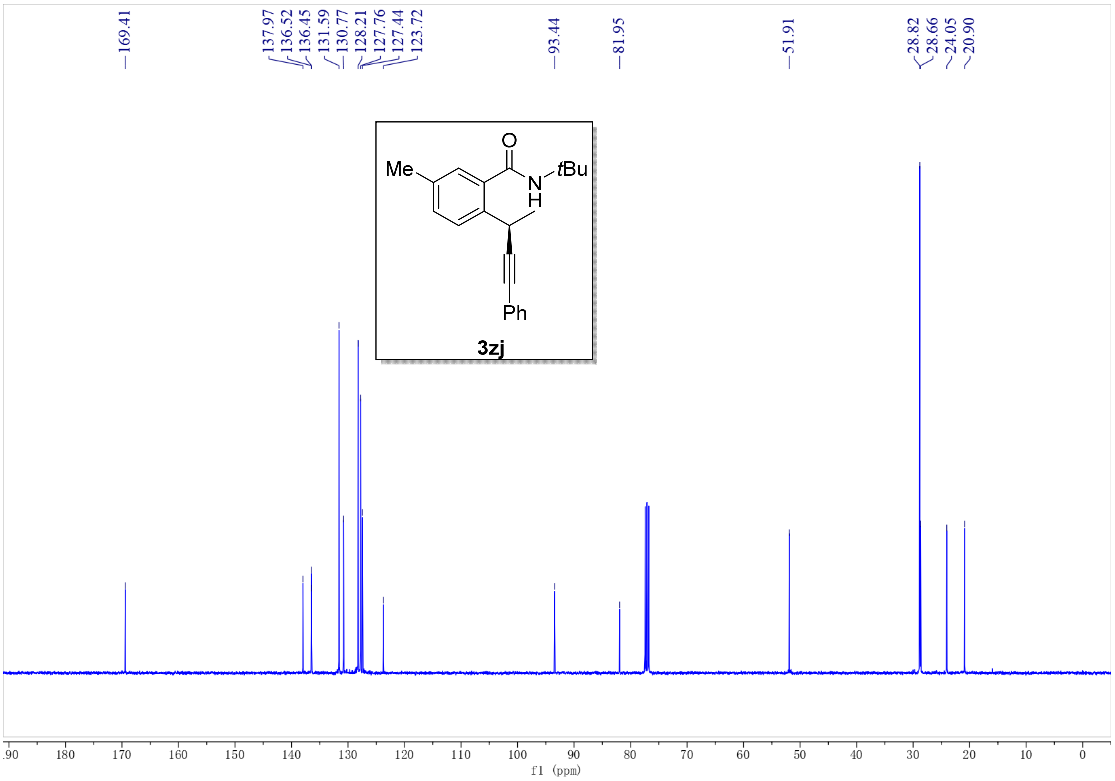

Supplementary Figure 141 ^{13}C NMR of **3zj**

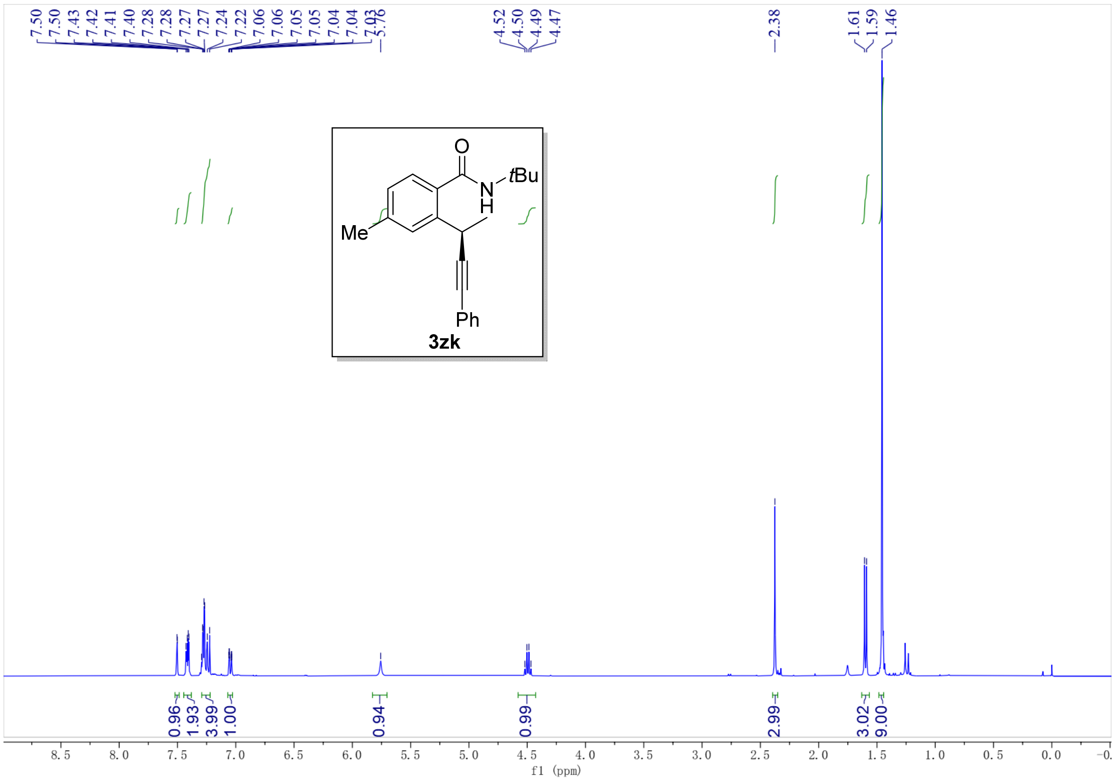

Supplementary Figure 142 ^{1}H NMR of **3zk**

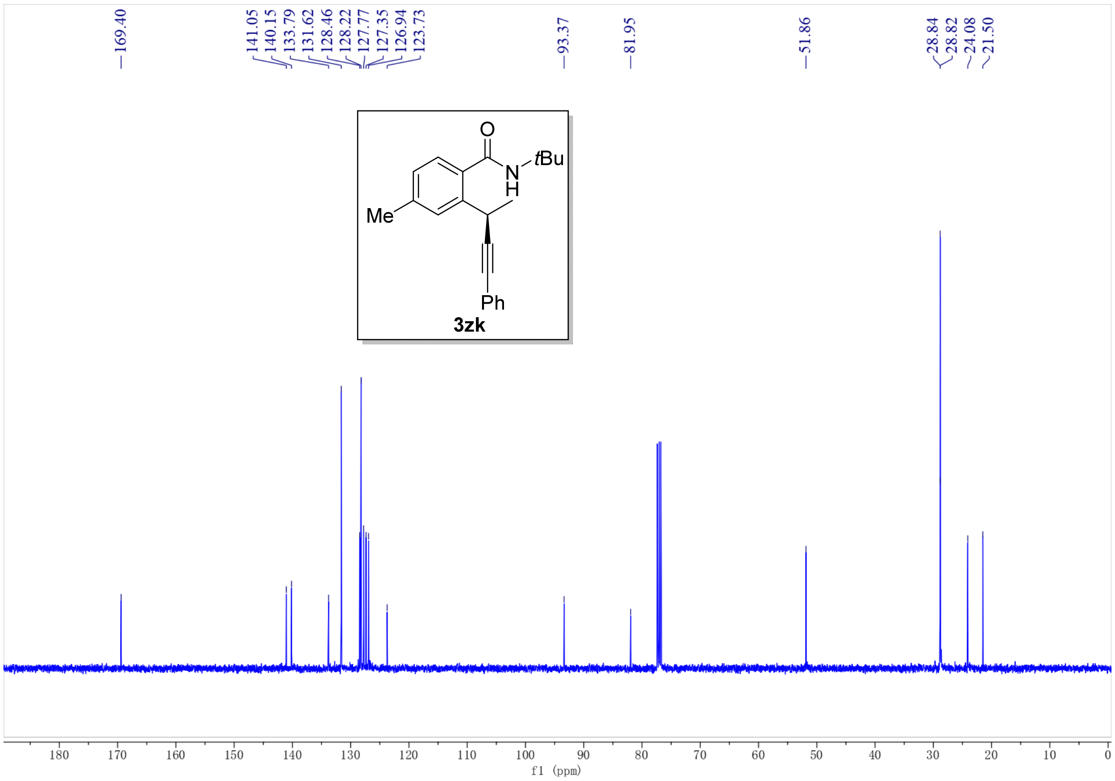

Supplementary Figure 143 ^{13}C NMR of **3zk**

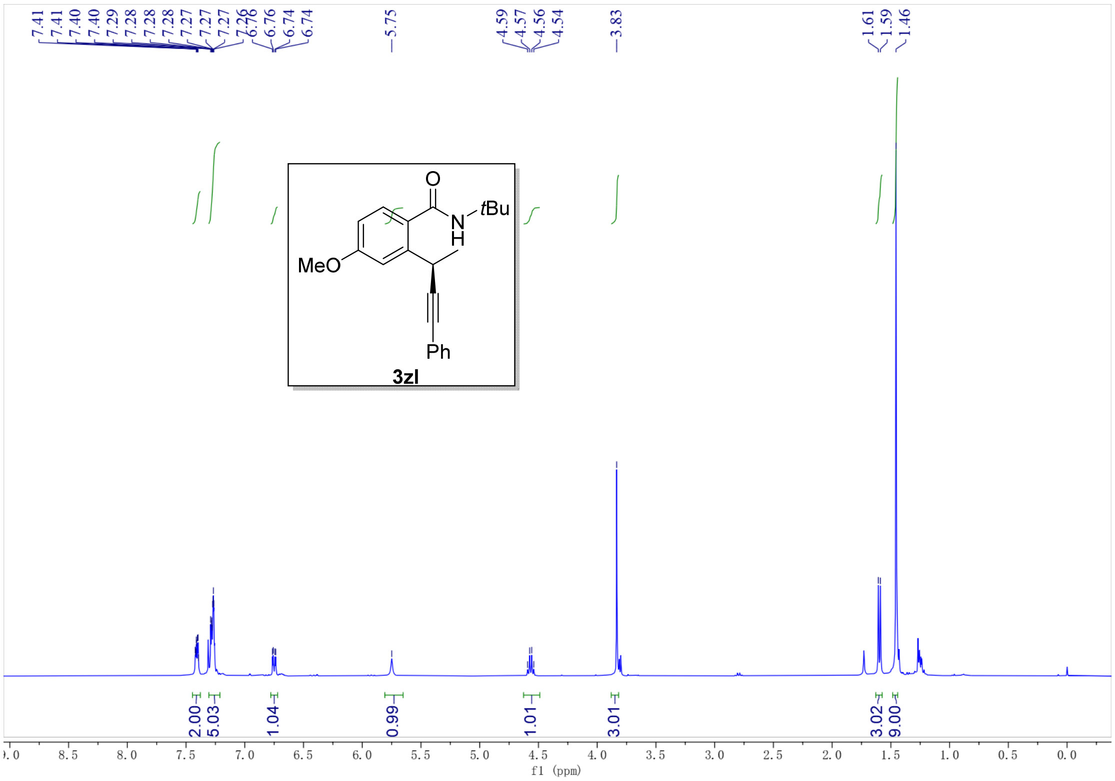

Supplementary Figure 144 ^{1}H NMR of **3zl**

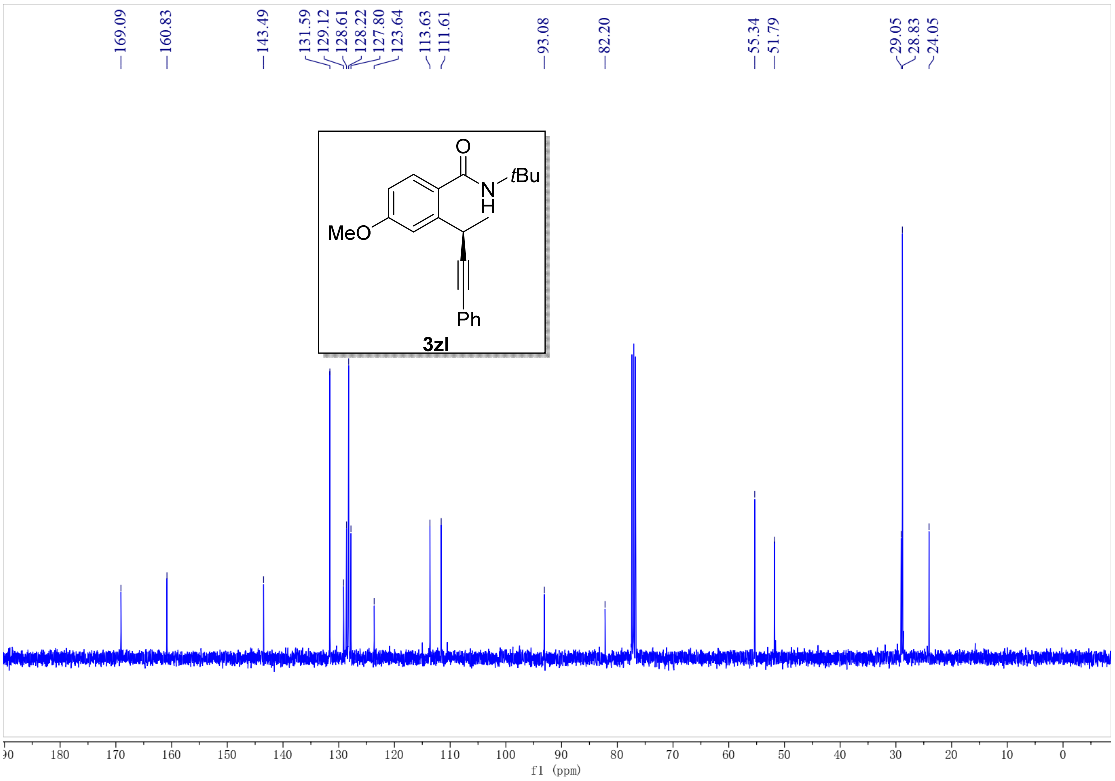

Supplementary Figure 145 ^{13}C NMR of **3zl**

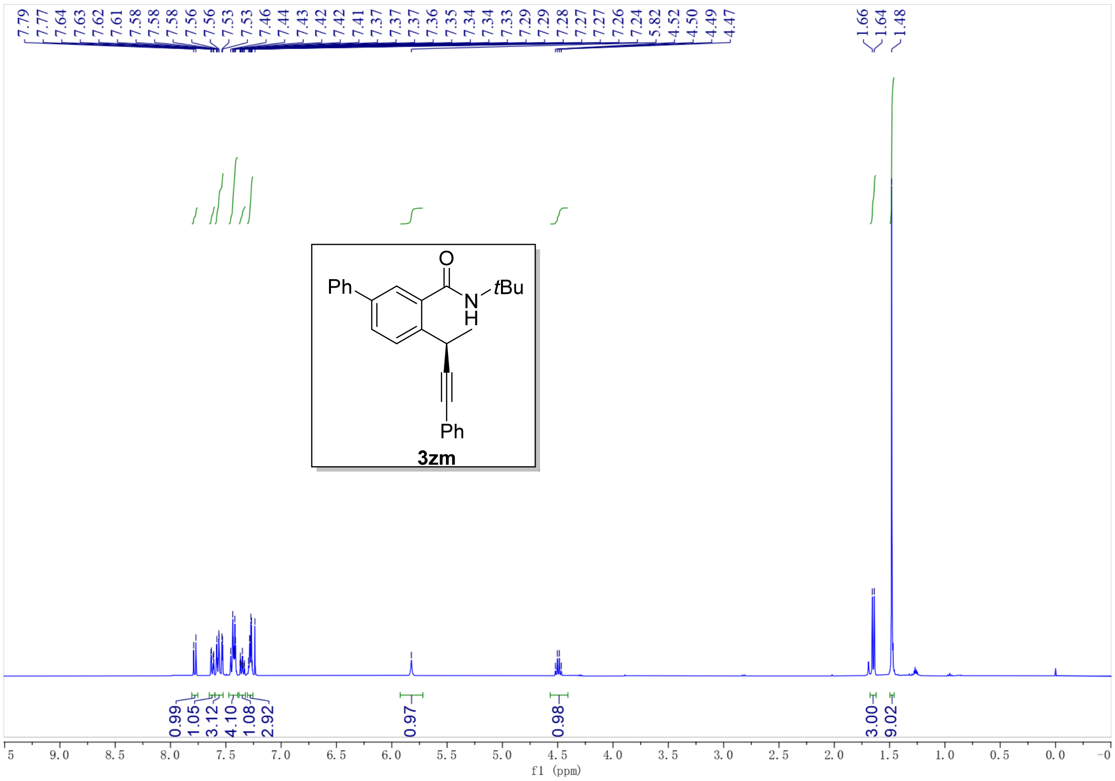

Supplementary Figure 146 ^{1}H NMR of **3zm**

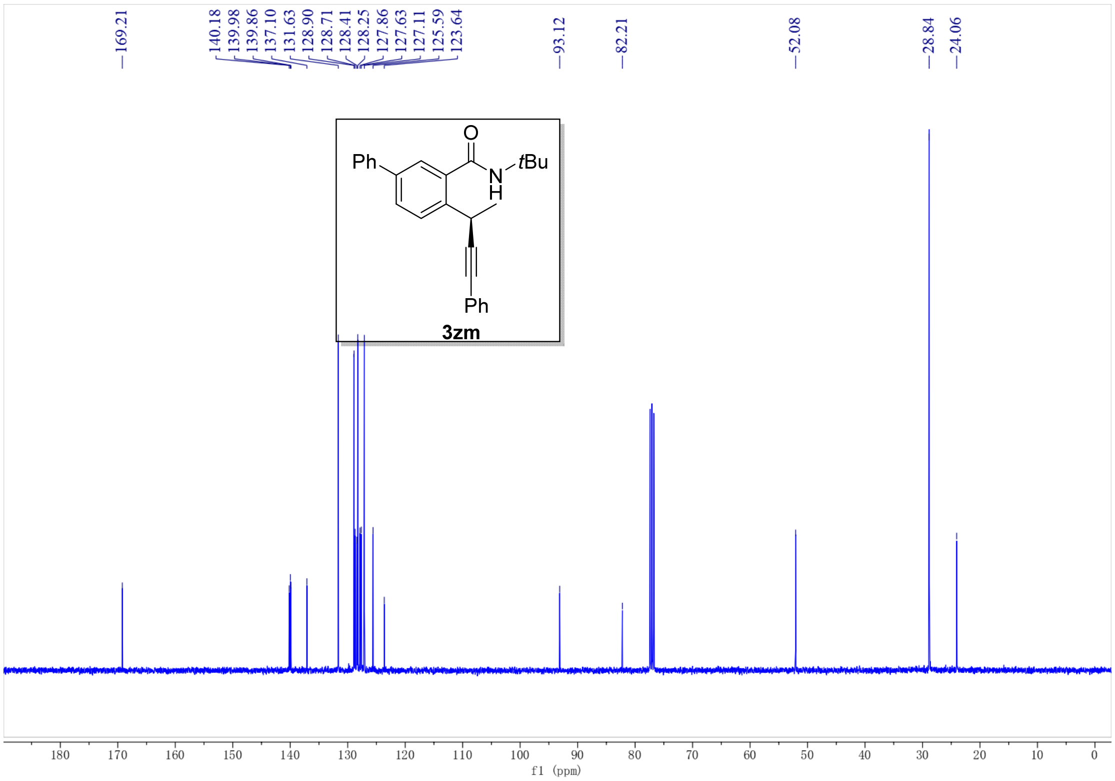

Supplementary Figure 147 ^{13}C NMR of **3zm**

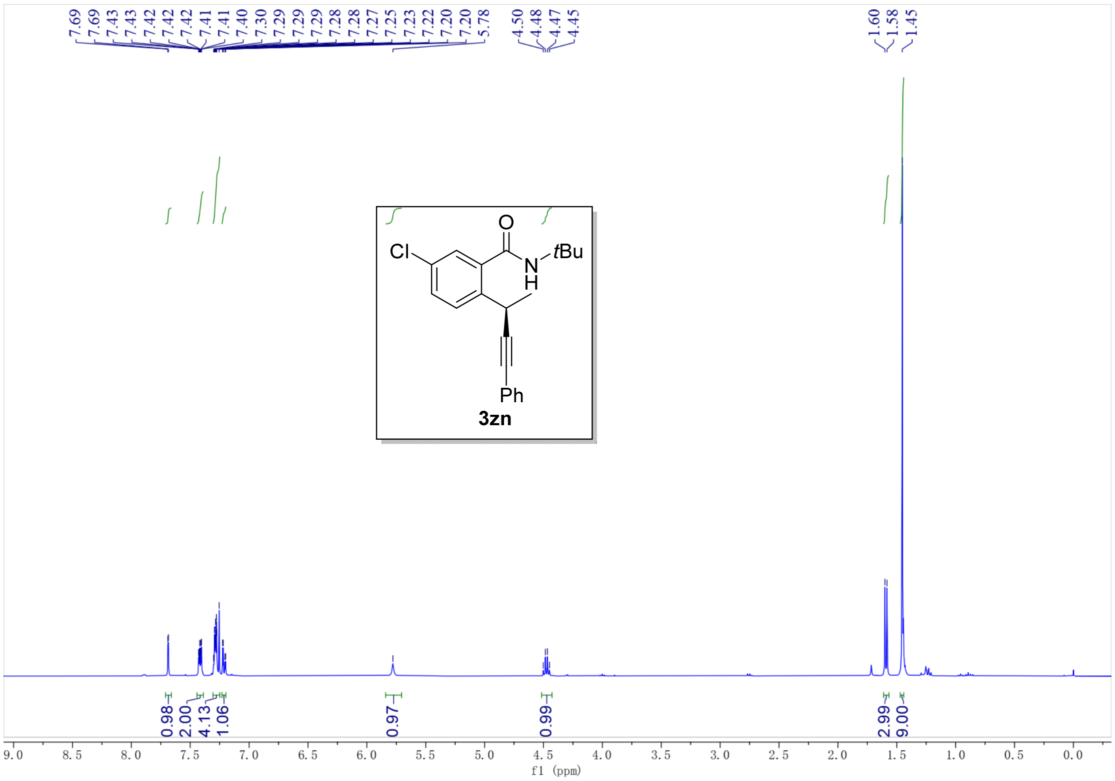

Supplementary Figure 148 ^{1}H NMR of **3zn**

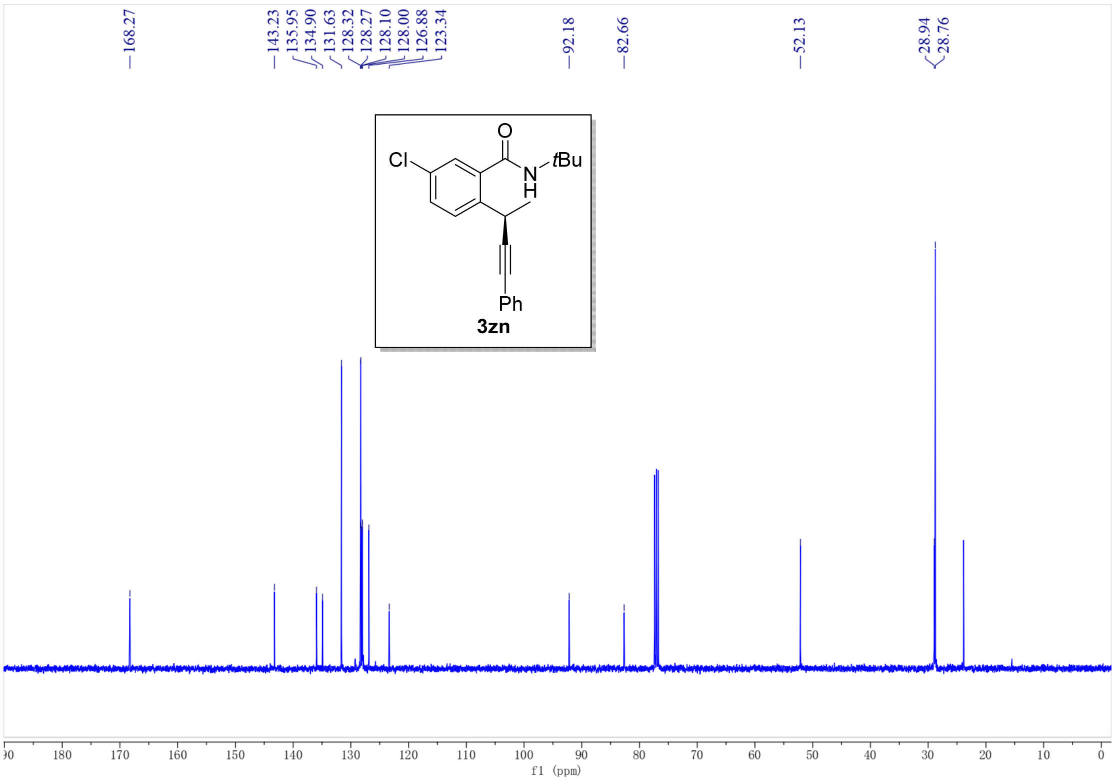

Supplementary Figure 149 ^{13}C NMR of **3zn**

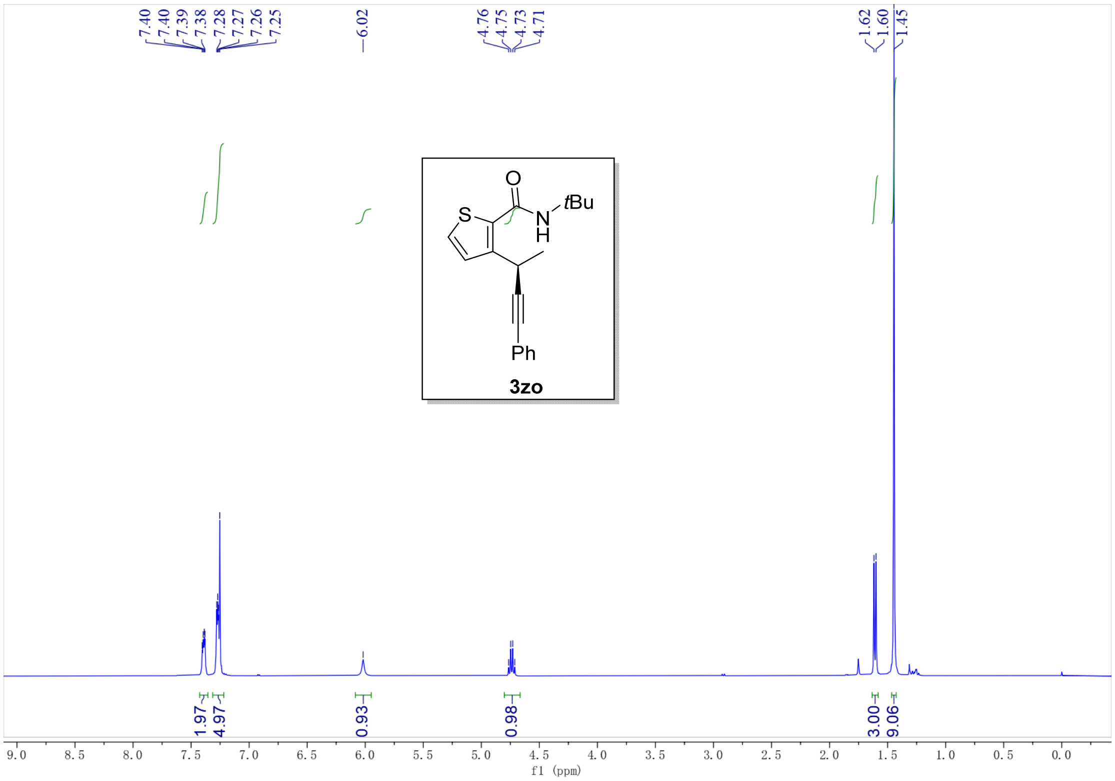

Supplementary Figure 150 ^{1}H NMR of **3zo**

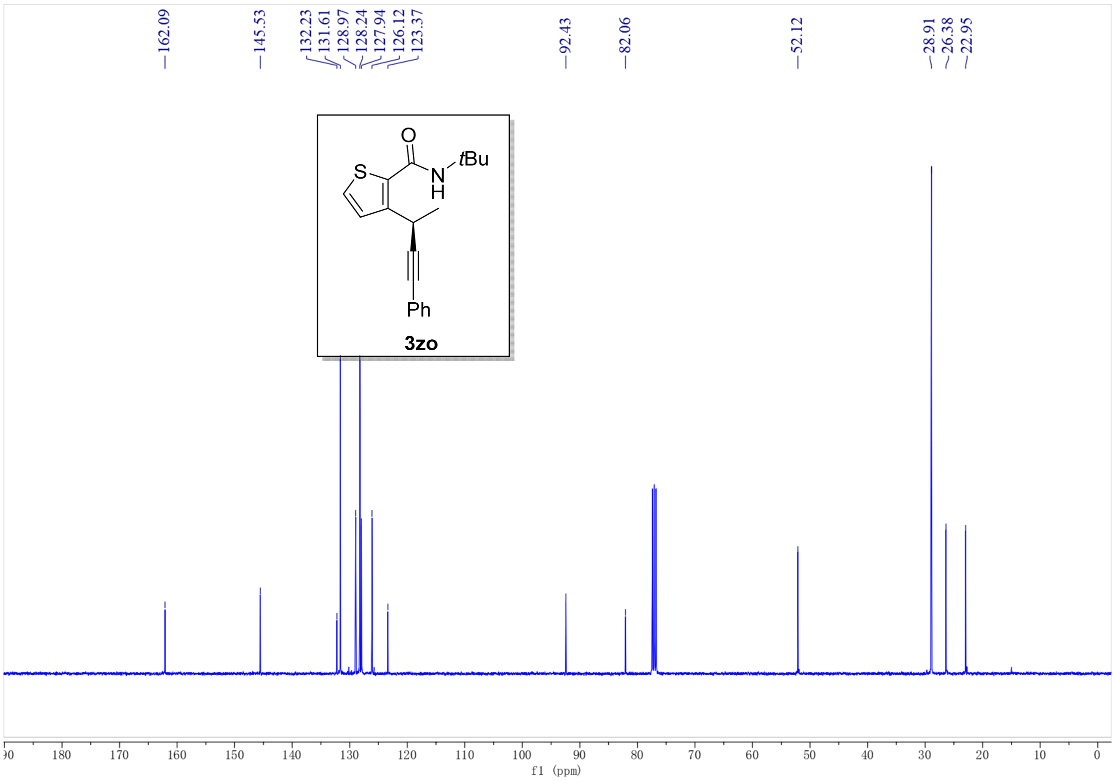

Supplementary Figure 151 ^{13}C NMR of **3zo**

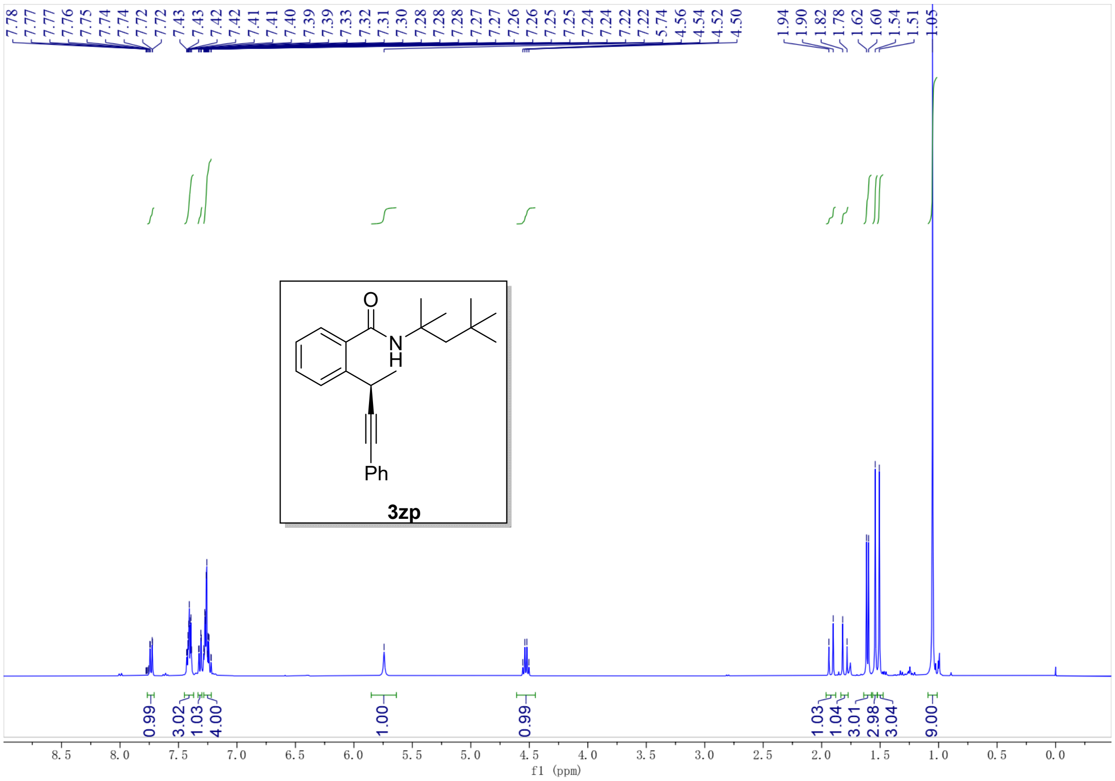

Supplementary Figure 152 ^{1}H NMR of **3zp**

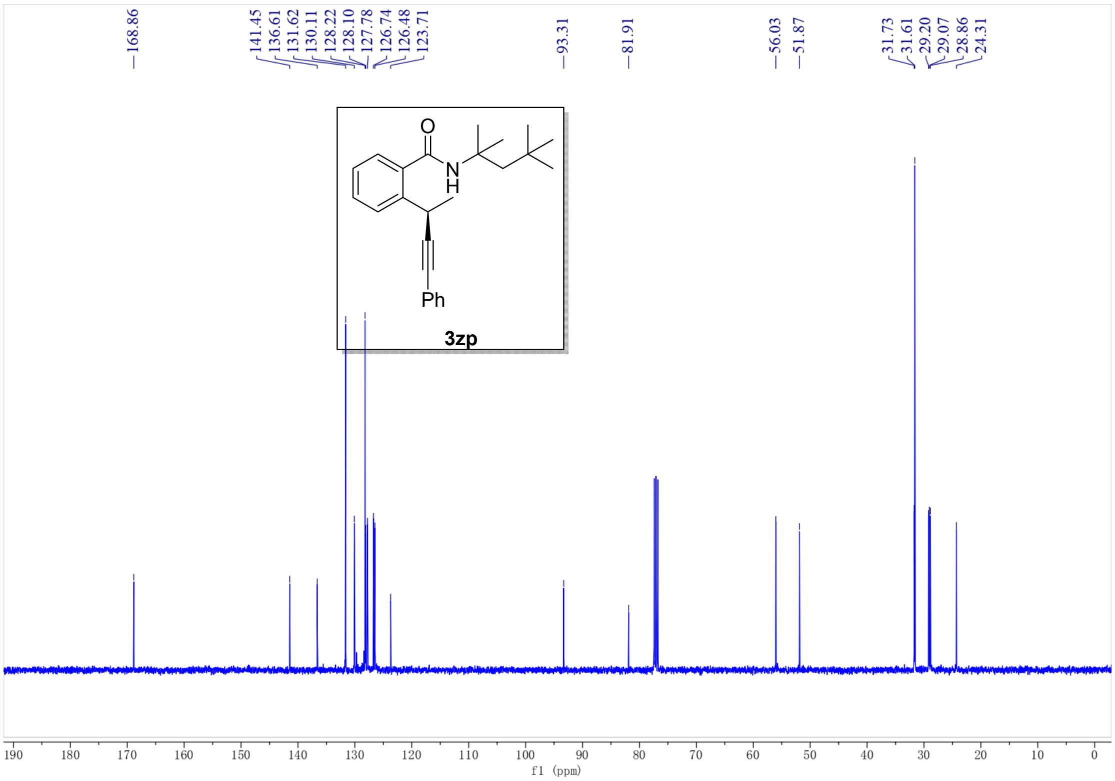

Supplementary Figure 153 ^{13}C NMR of **3zp**

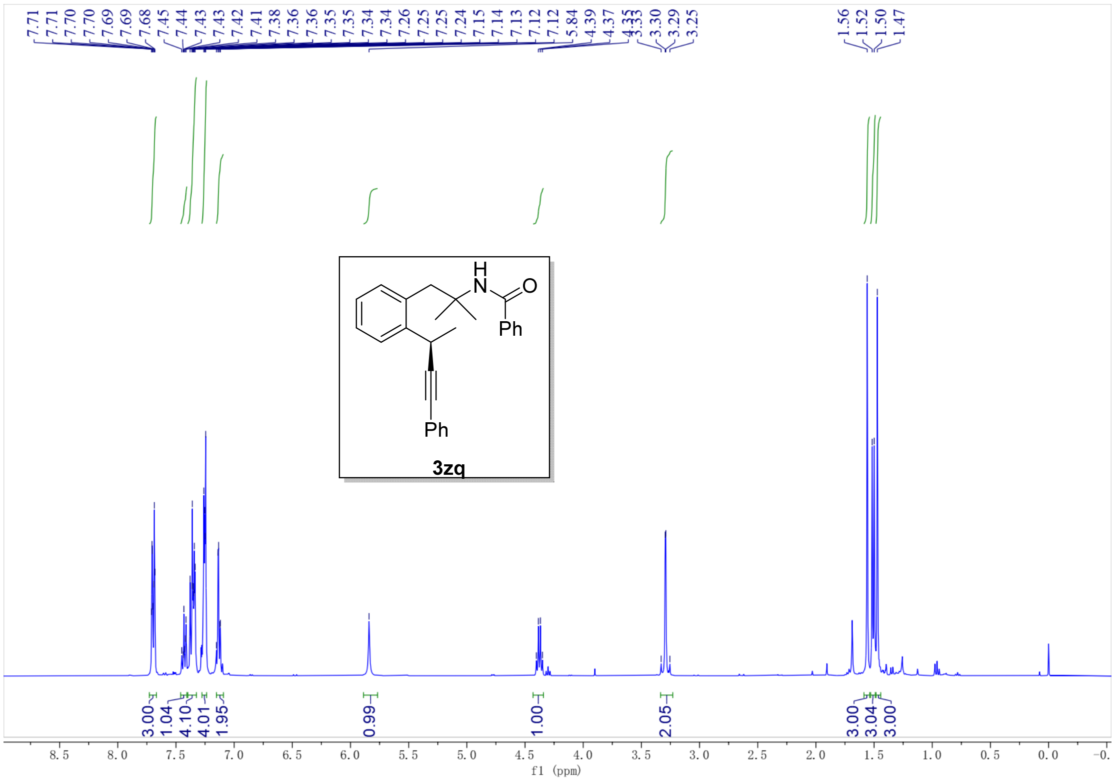

Supplementary Figure 154 ^{1}H NMR of **3zq**

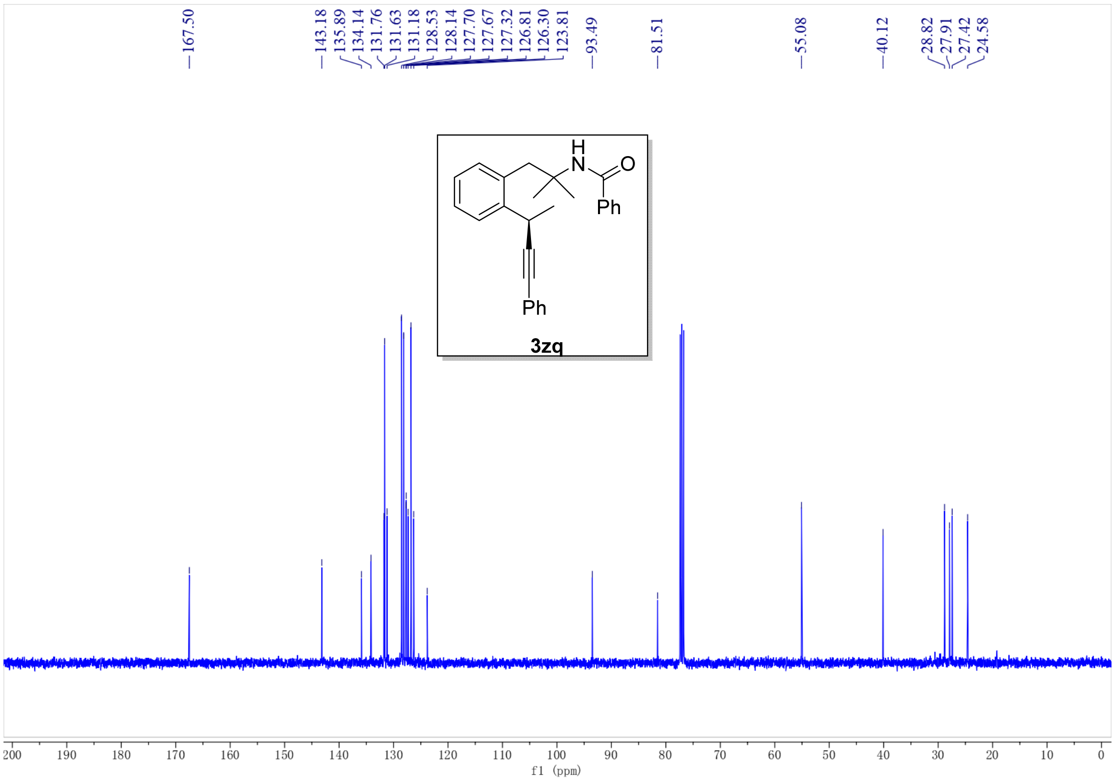

Supplementary Figure 155 ^{13}C NMR of **3zq**

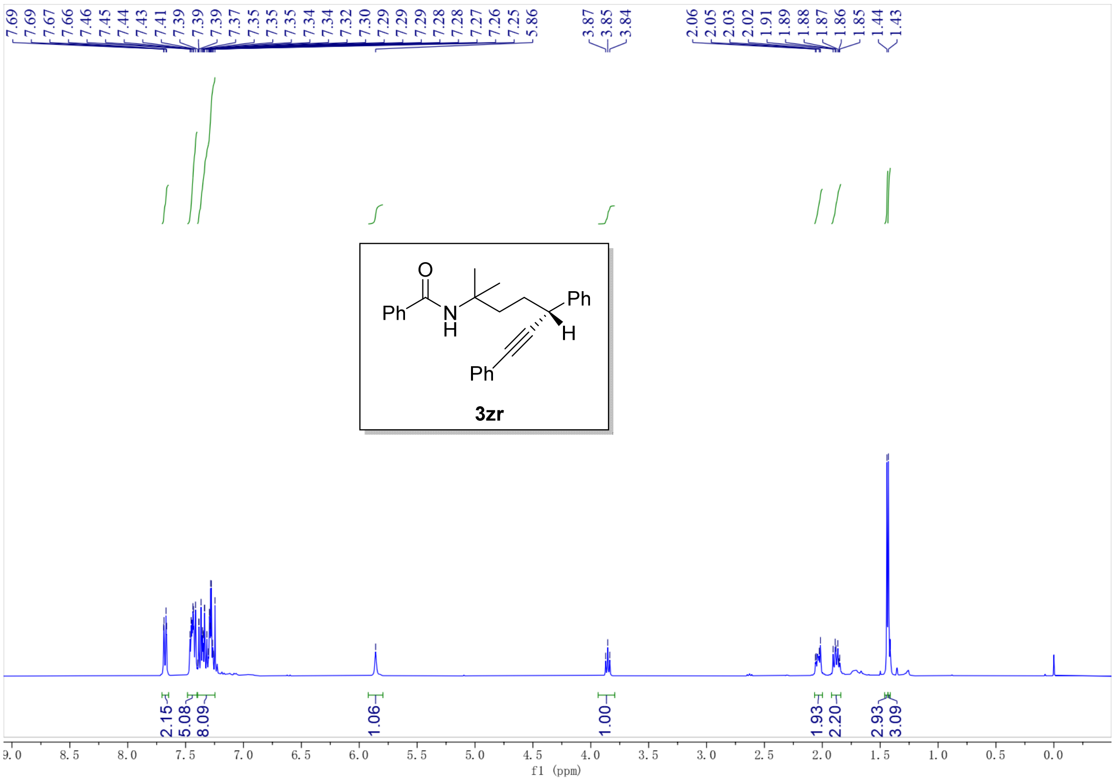

Supplementary Figure 156 ^{1}H NMR of **3zr**

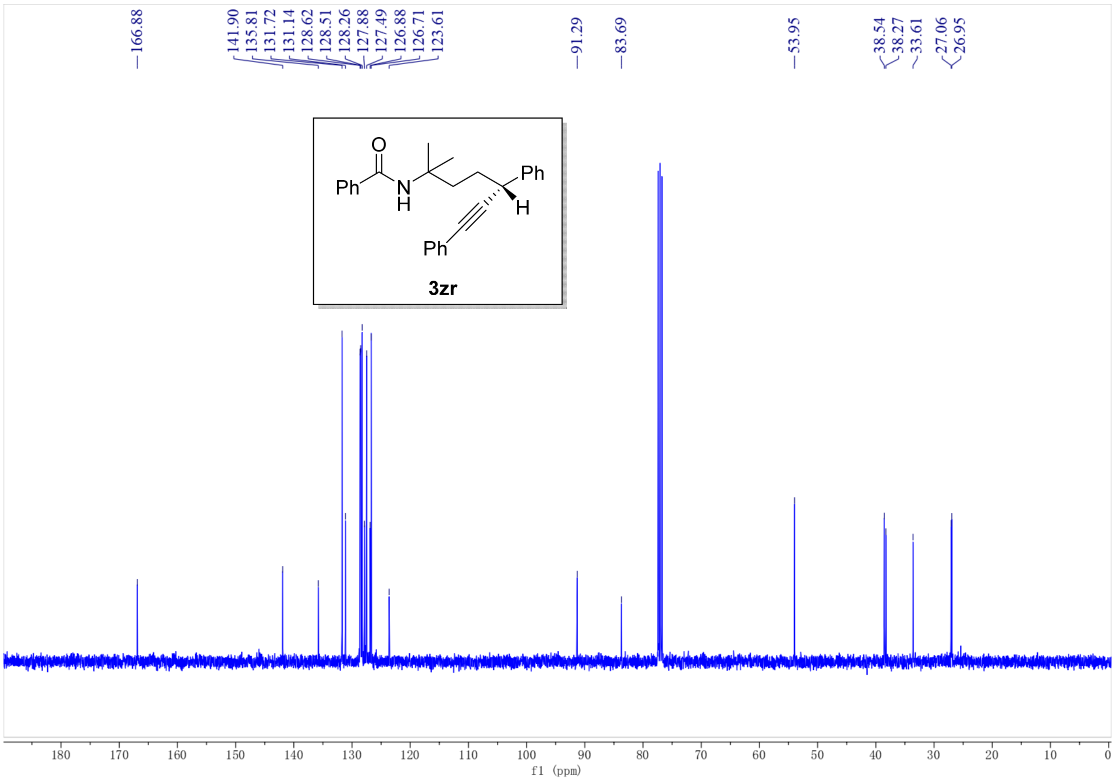

Supplementary Figure 157 ^{13}C NMR of **3zr**

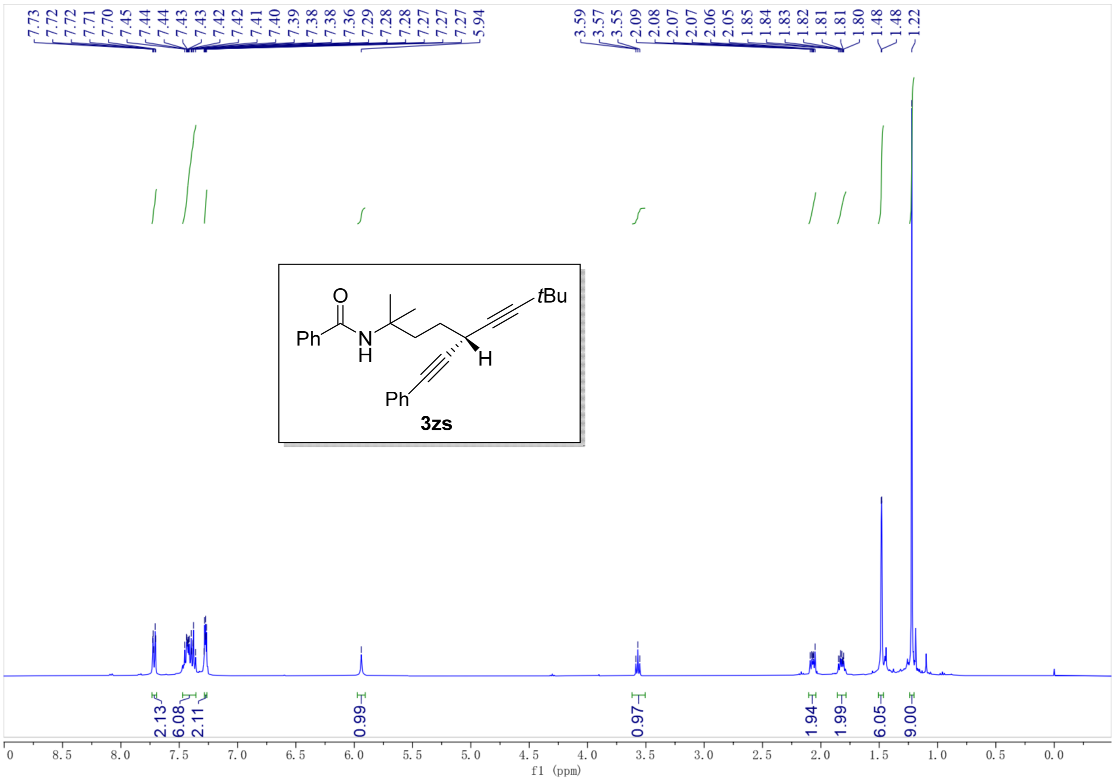

Supplementary Figure 158 ^{1}H NMR of **3zs**

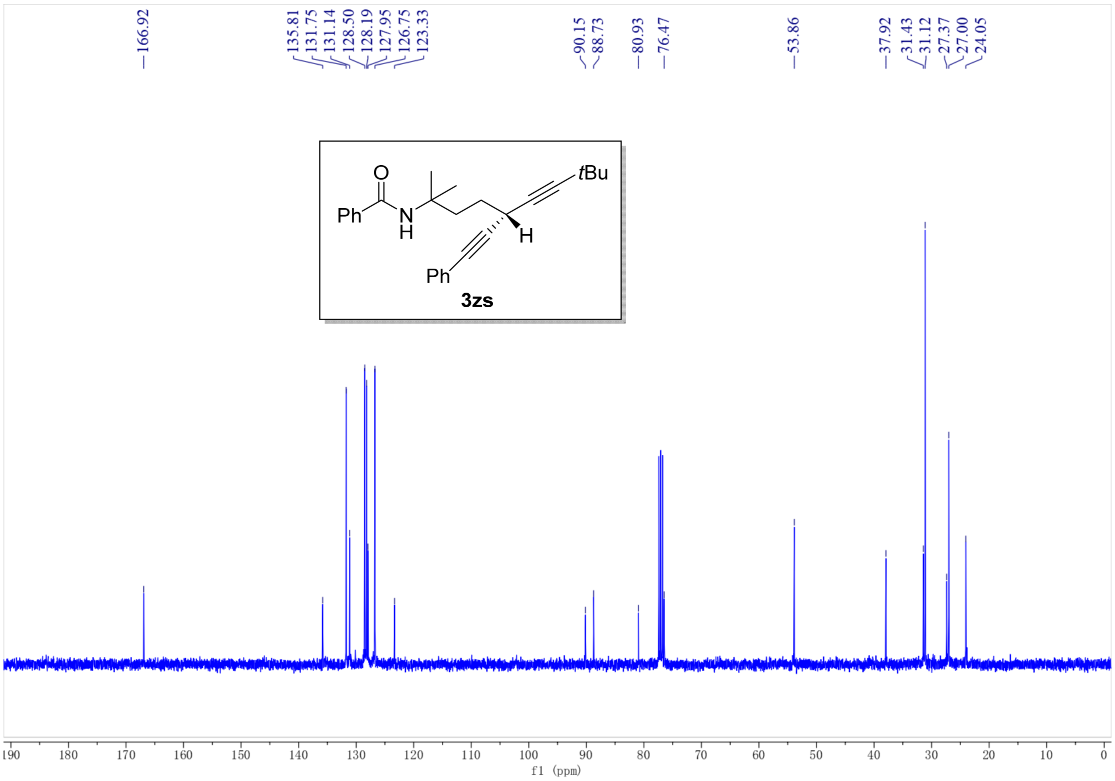

Supplementary Figure 159 ^{13}C NMR of **3zs**

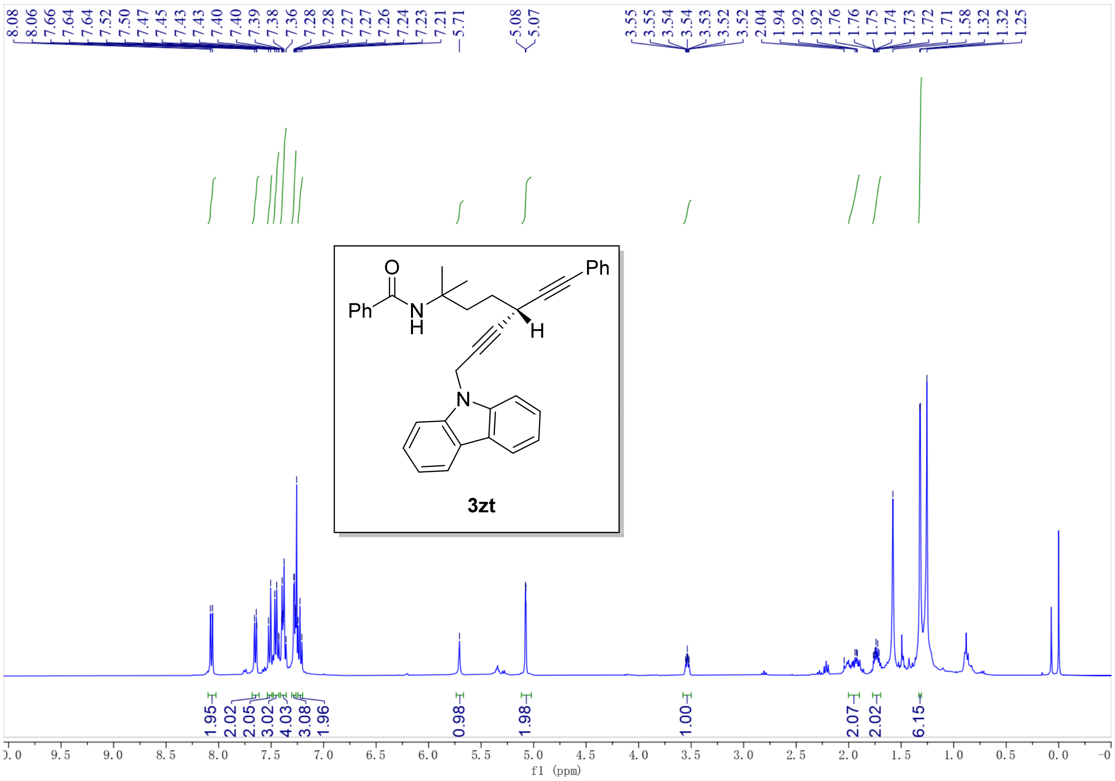

Supplementary Figure 160 ^{1}H NMR of **3zt**

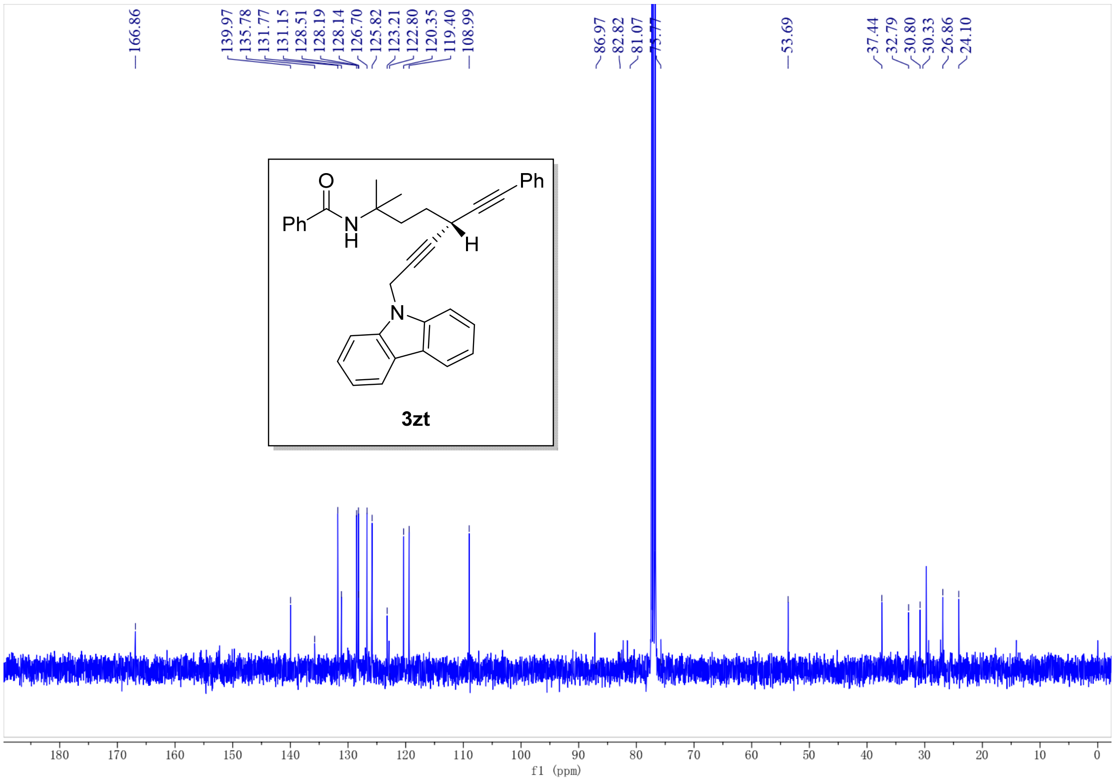

Supplementary Figure 161 ^{13}C NMR of **3zt**

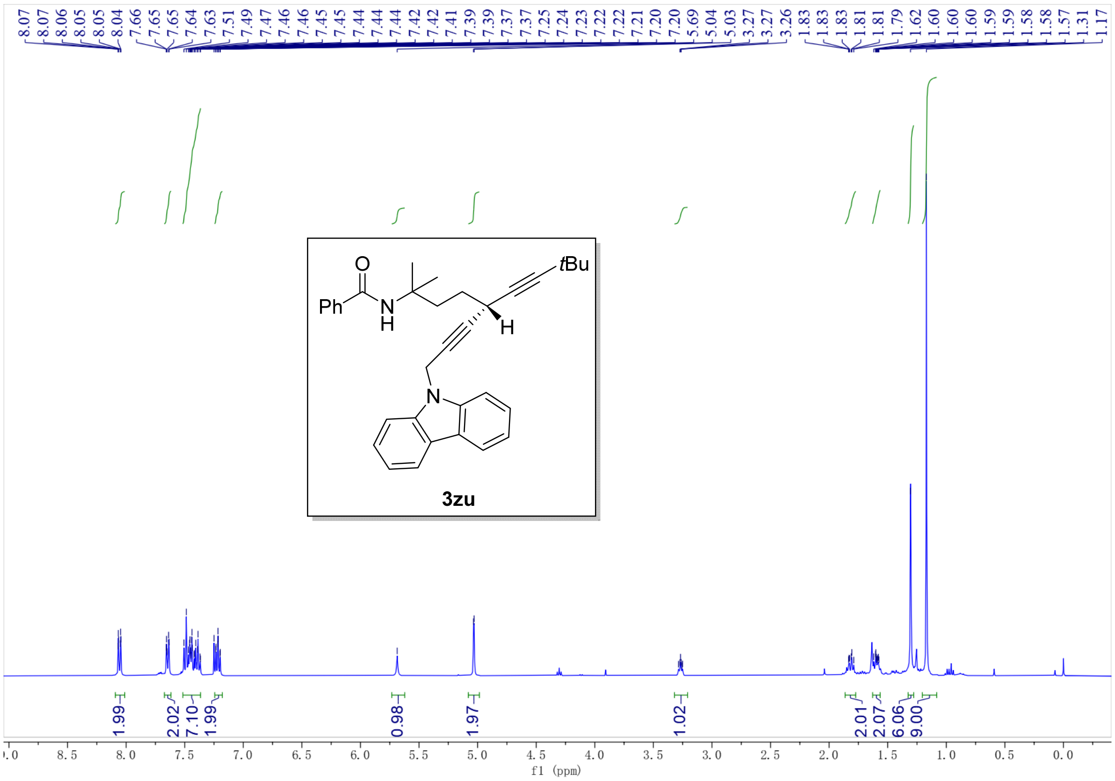

Supplementary Figure 162 ^{1}H NMR of **3zu**

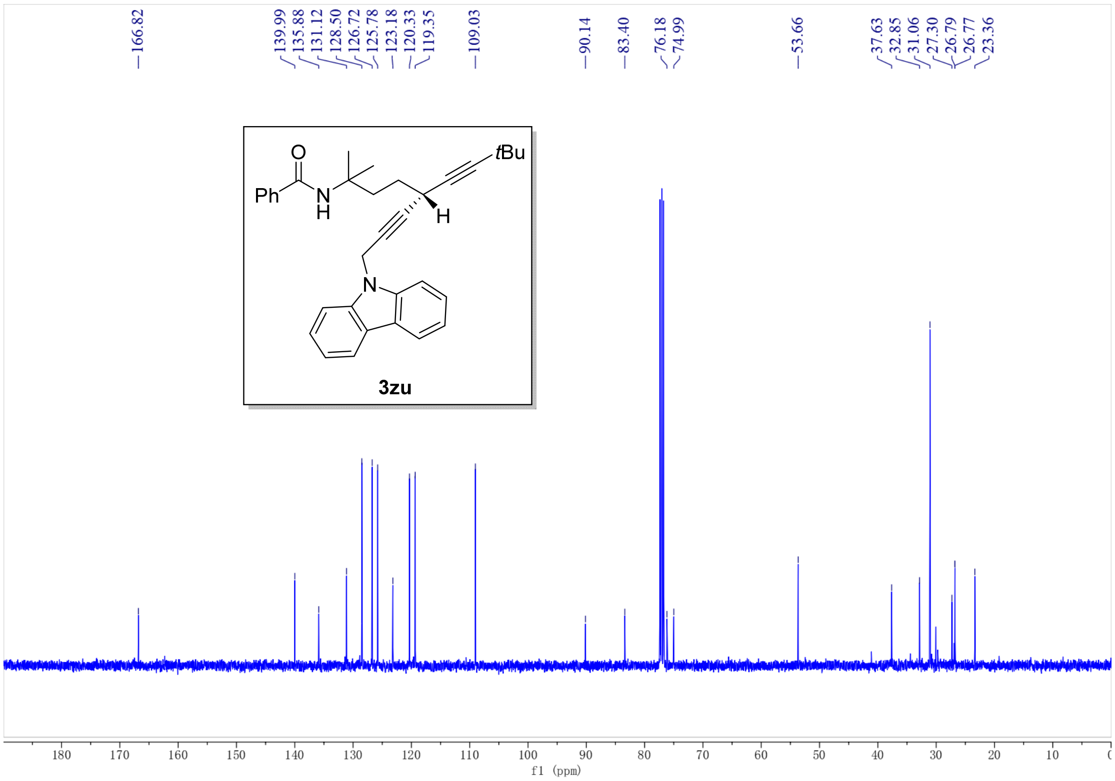

Supplementary Figure 163 ^{13}C NMR of **3zu**

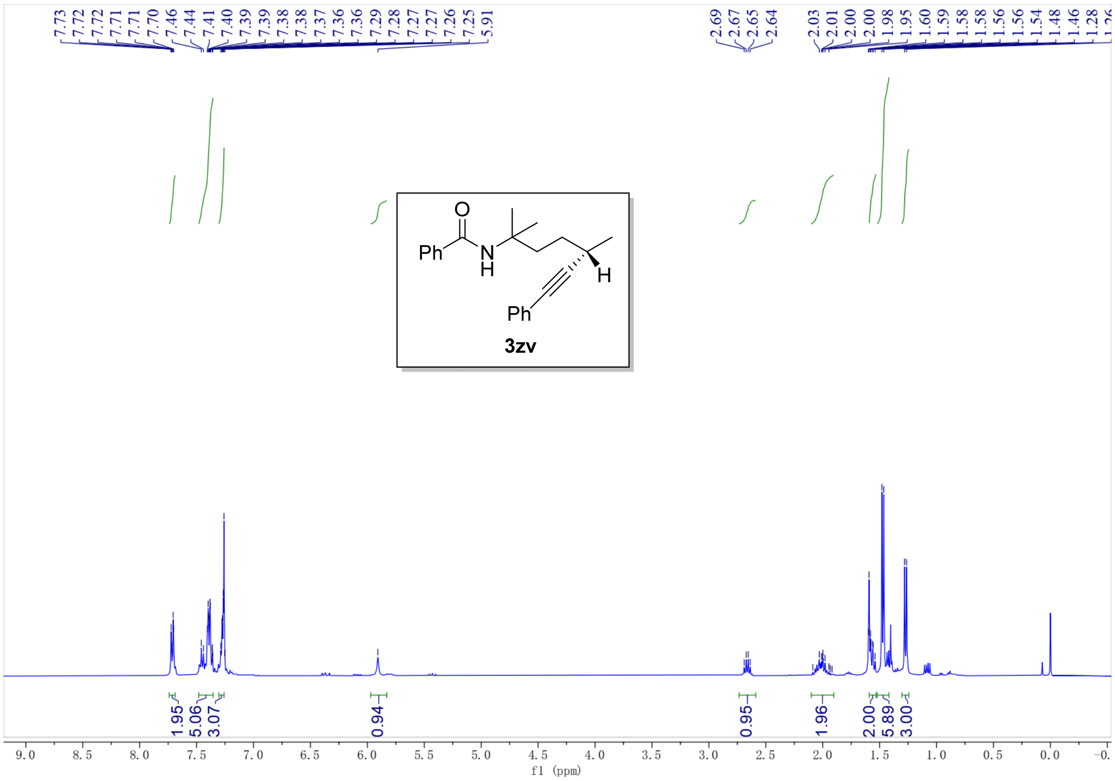

Supplementary Figure 164 ^{1}H NMR of **3zv**

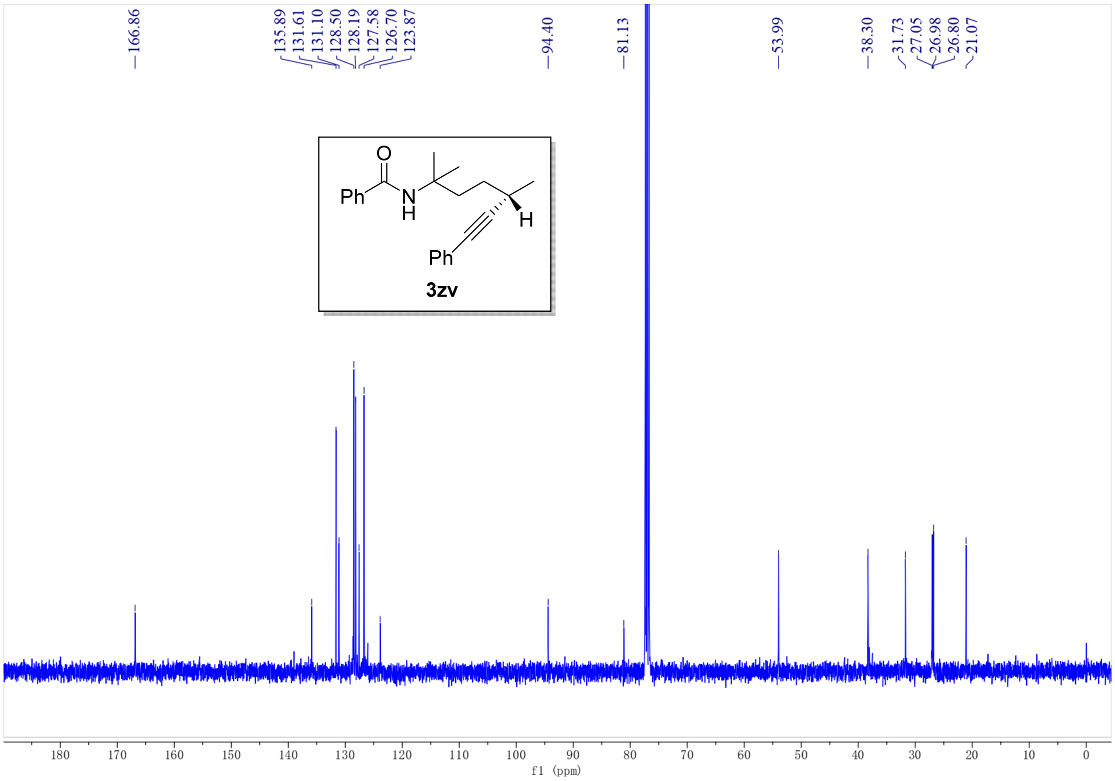

Supplementary Figure 165 ^{13}C NMR of **3zv**

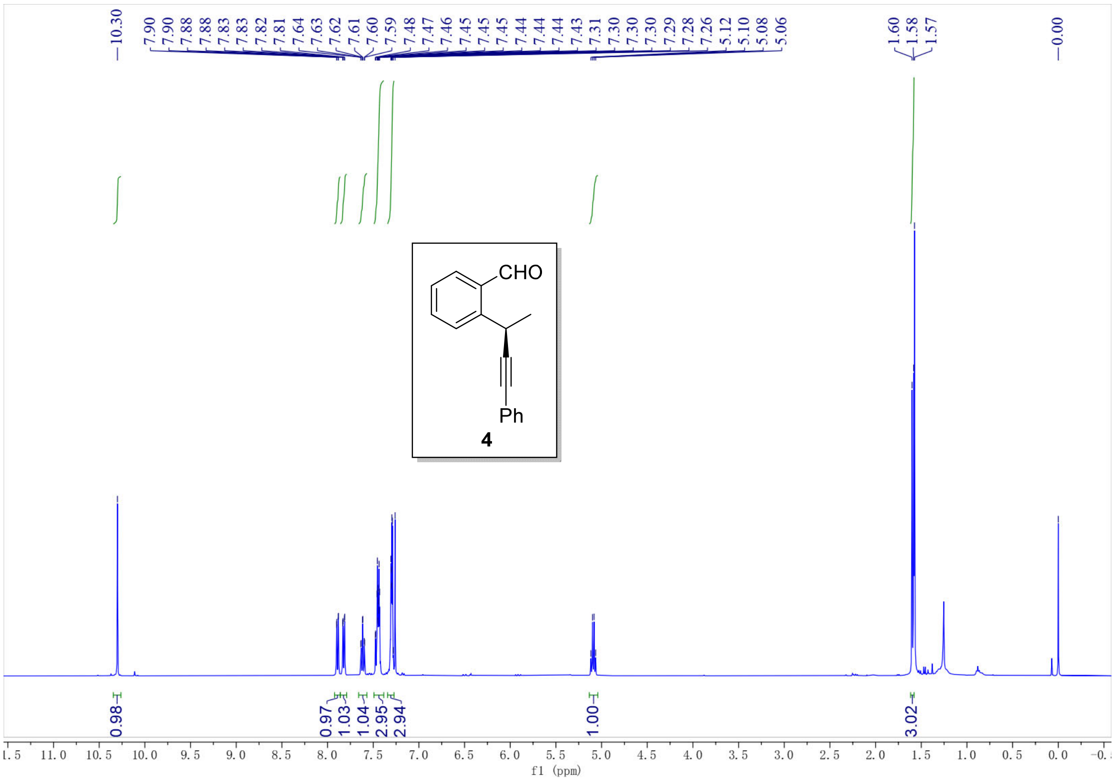

Supplementary Figure 166 ^{1}H NMR of **4**

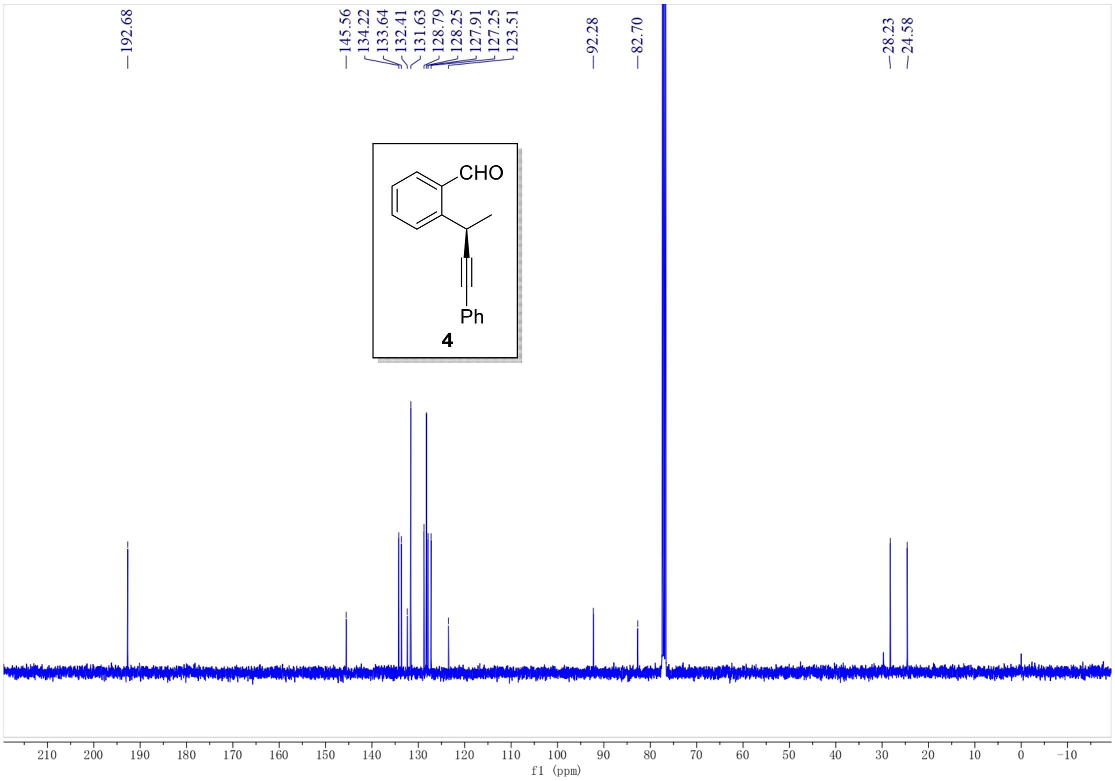

Supplementary Figure 167 ^{13}C NMR of **4**

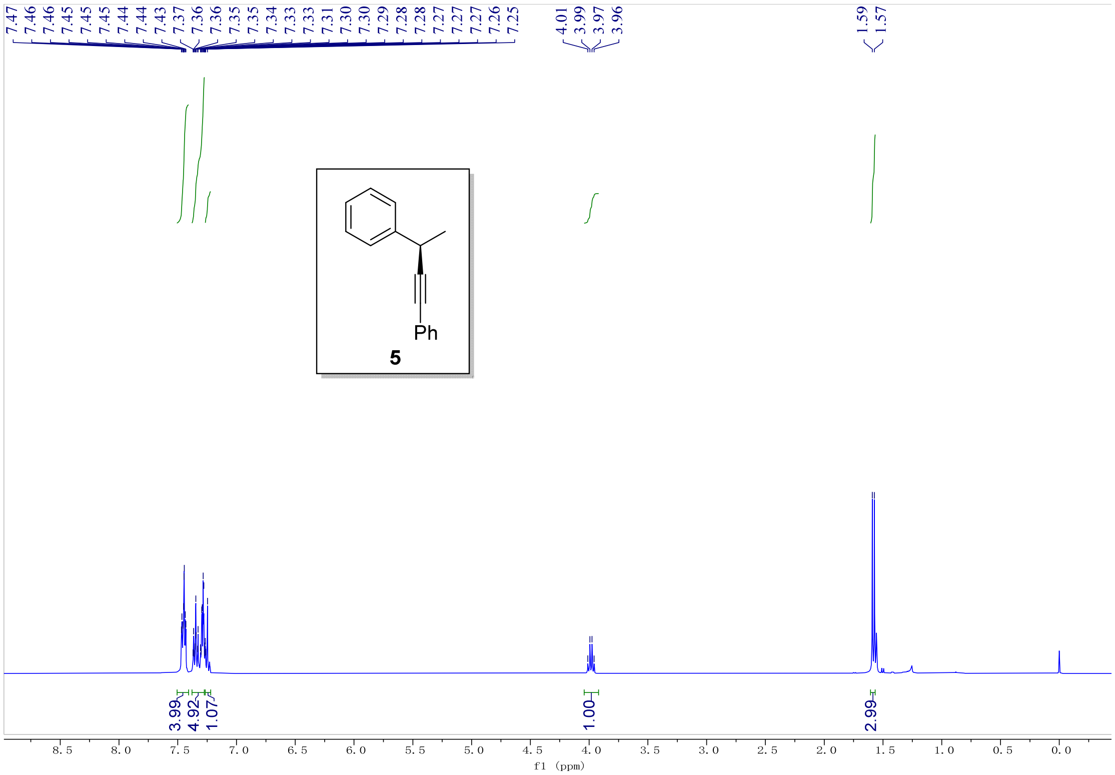

Supplementary Figure 168 ^{1}H NMR of **5**

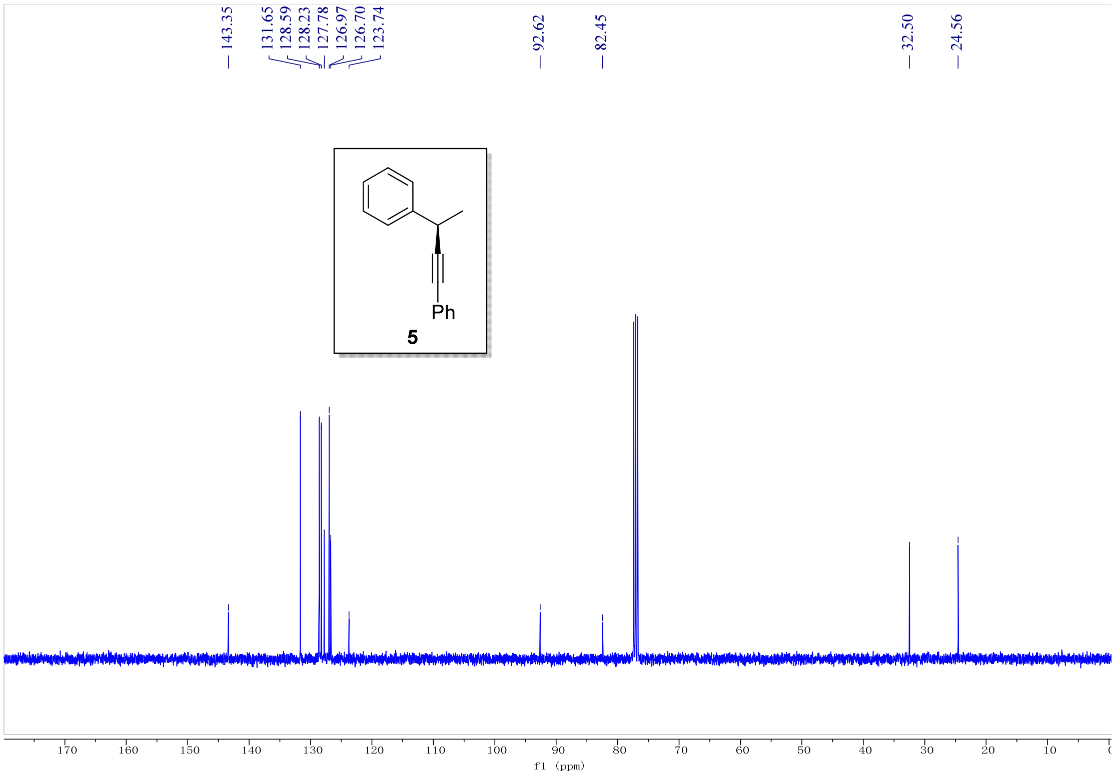

Supplementary Figure 169 ^{13}C NMR of **5**

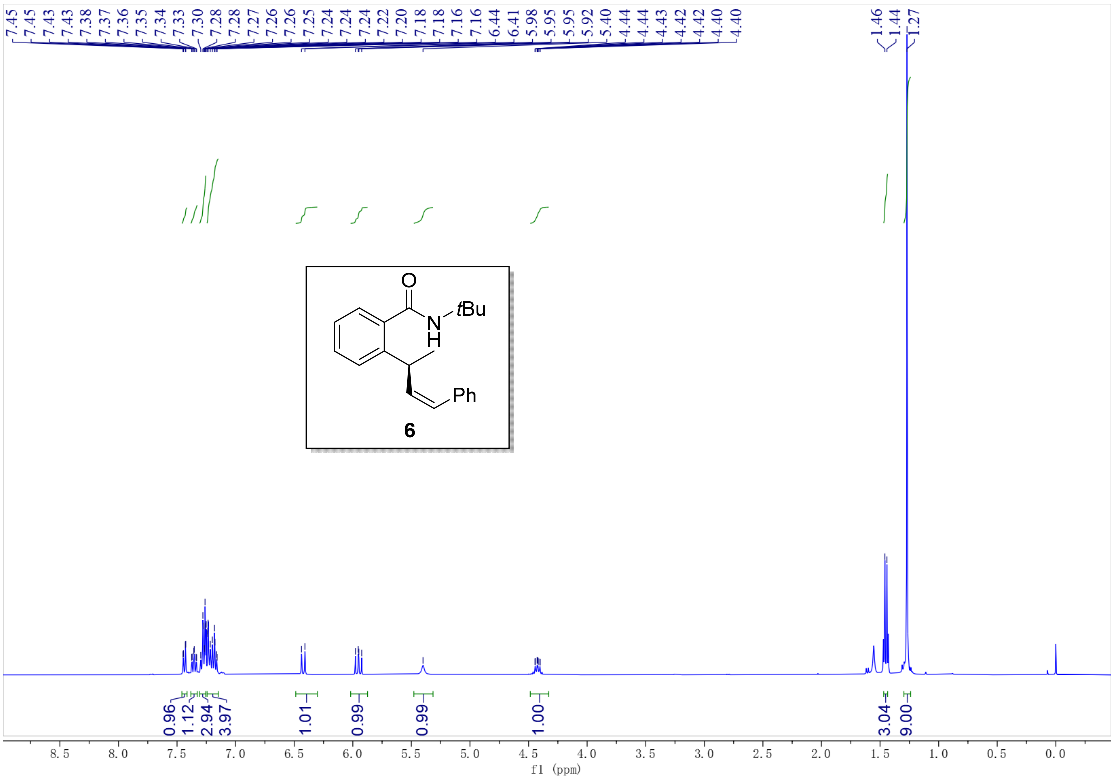

Supplementary Figure 170 ^{1}H NMR of **6**

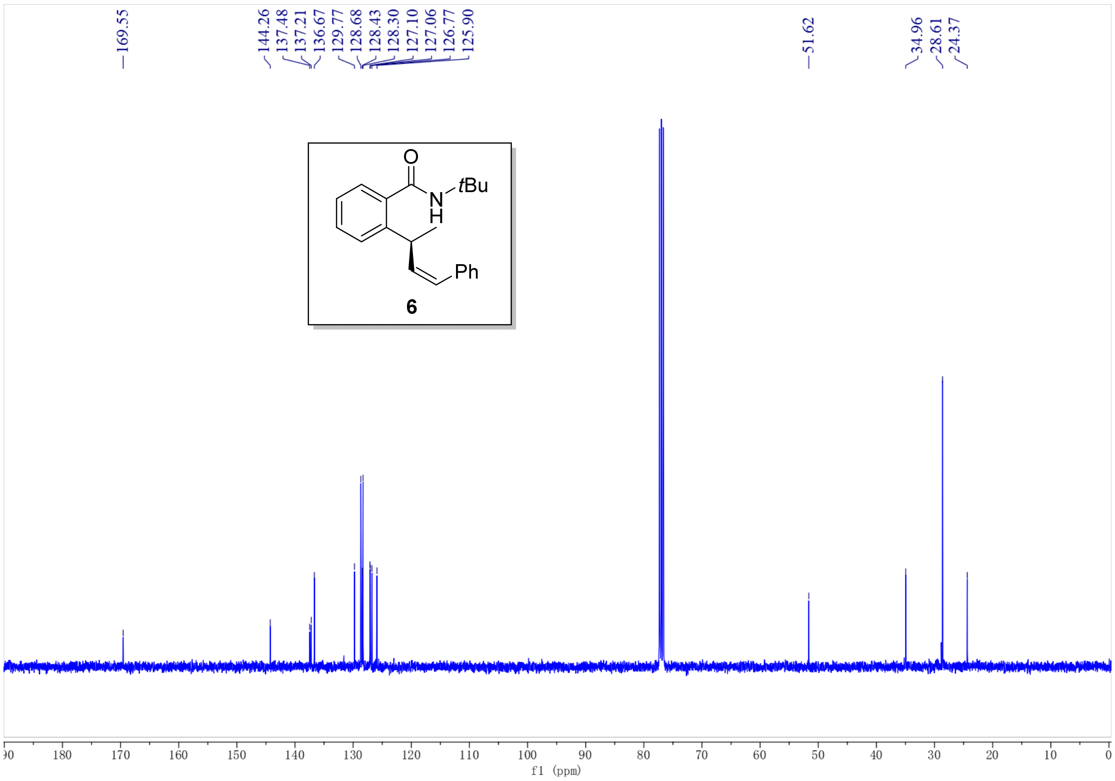

Supplementary Figure 171 ^{13}C NMR of **6**

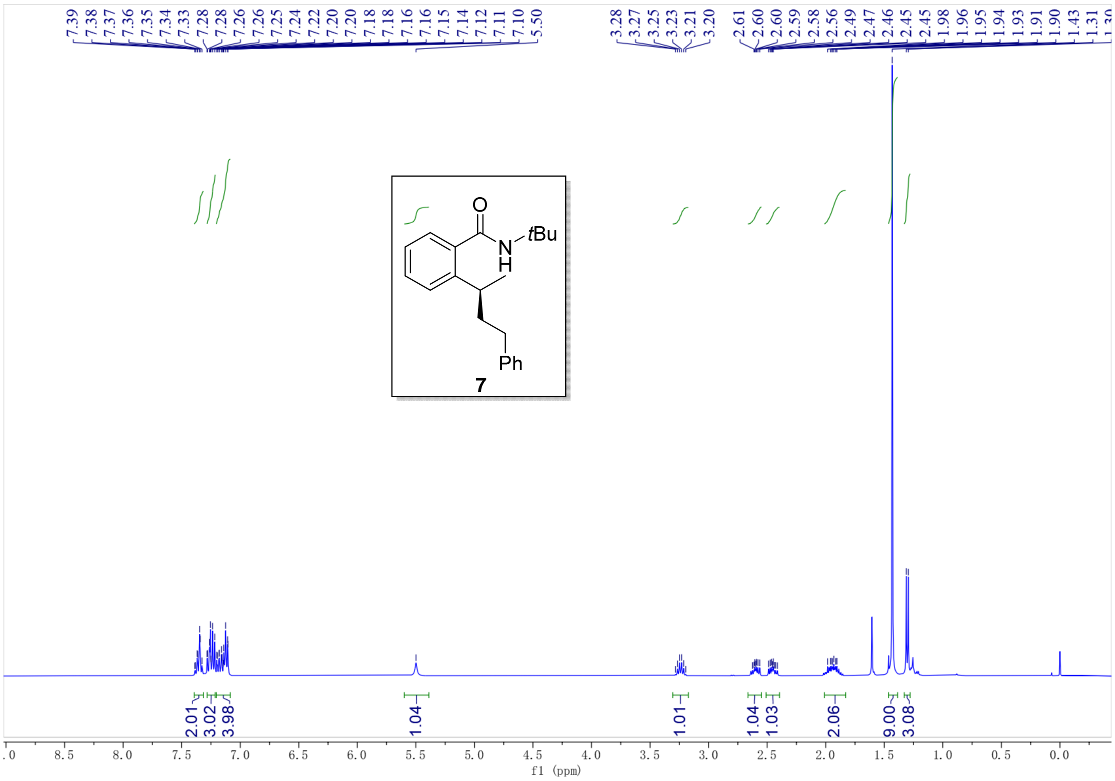

Supplementary Figure 172 ^{1}H NMR of **7**

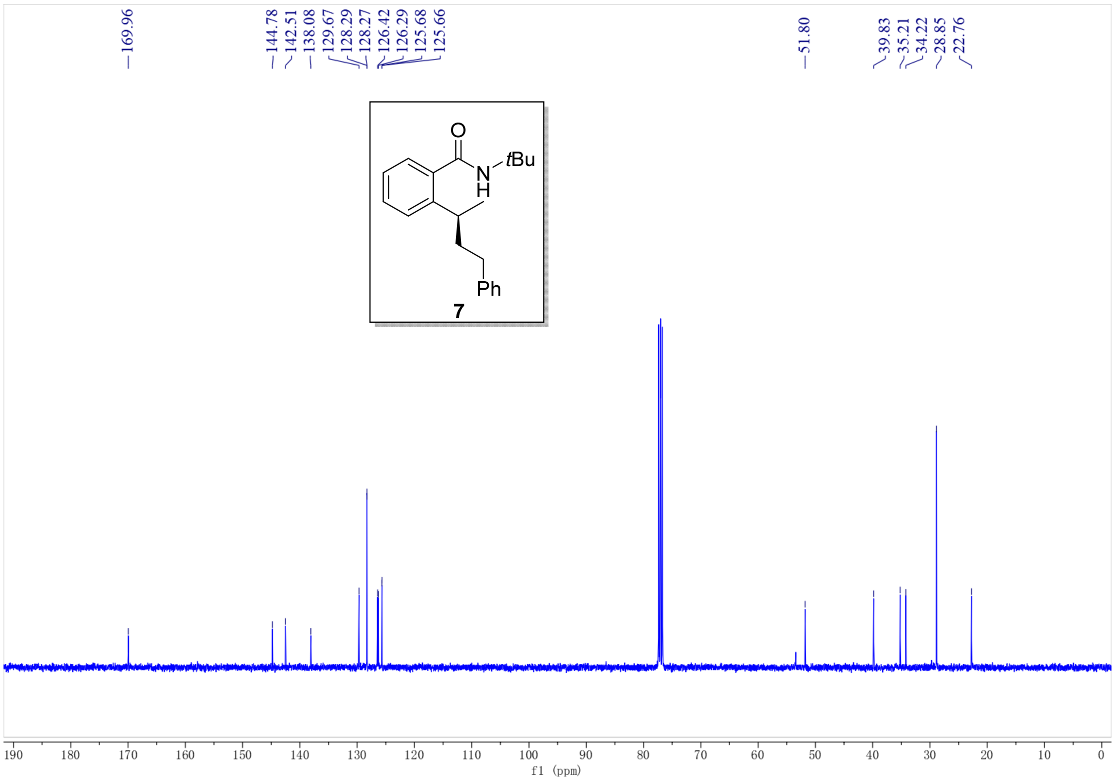

Supplementary Figure 173 ^{13}C NMR of **7**

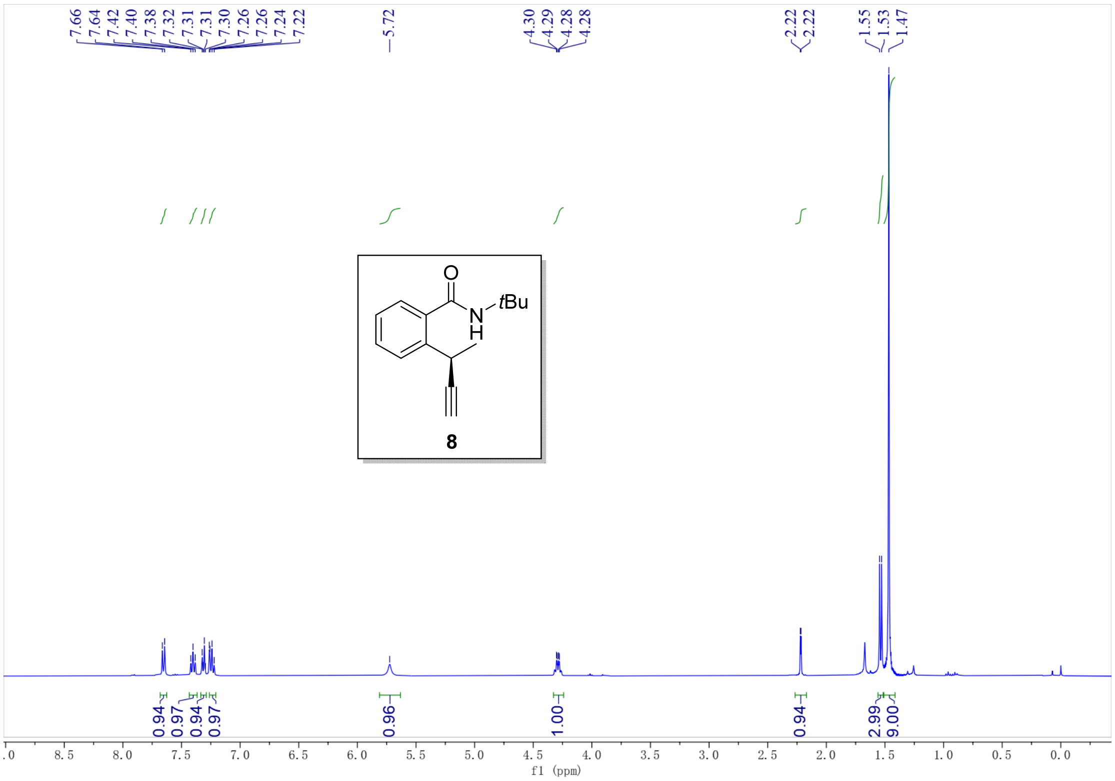

Supplementary Figure 174 ^{1}H NMR of **8**

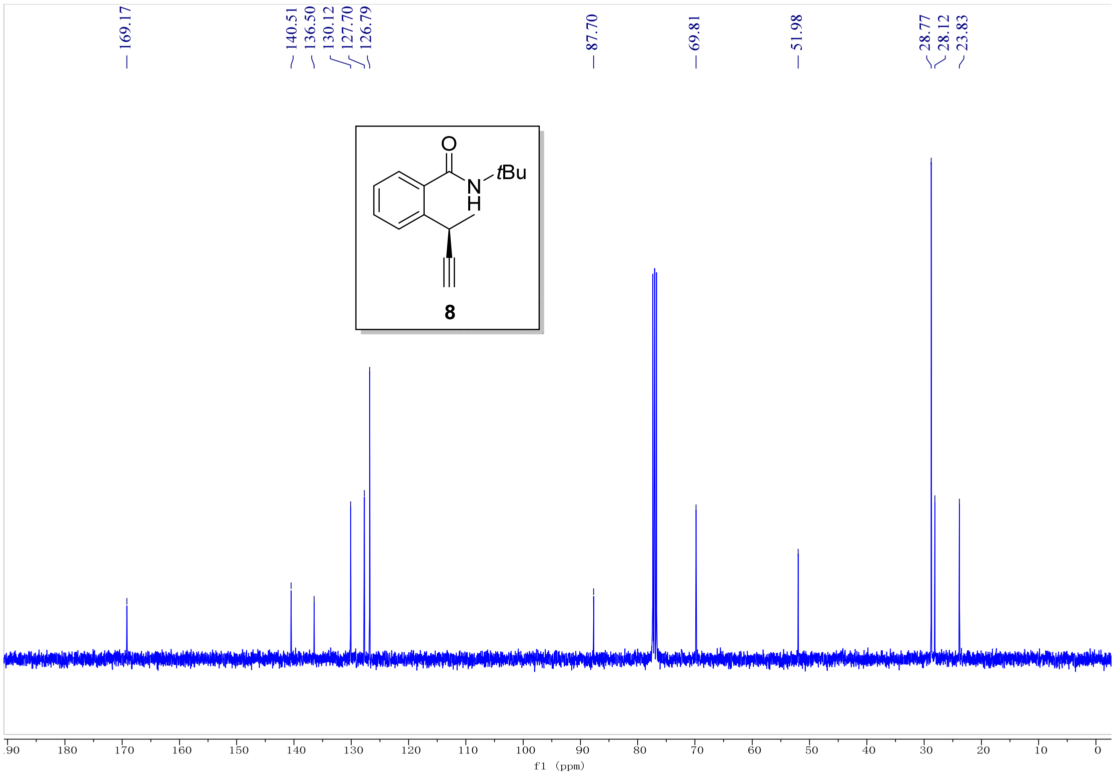

Supplementary Figure 175 ^{13}C NMR of **8**

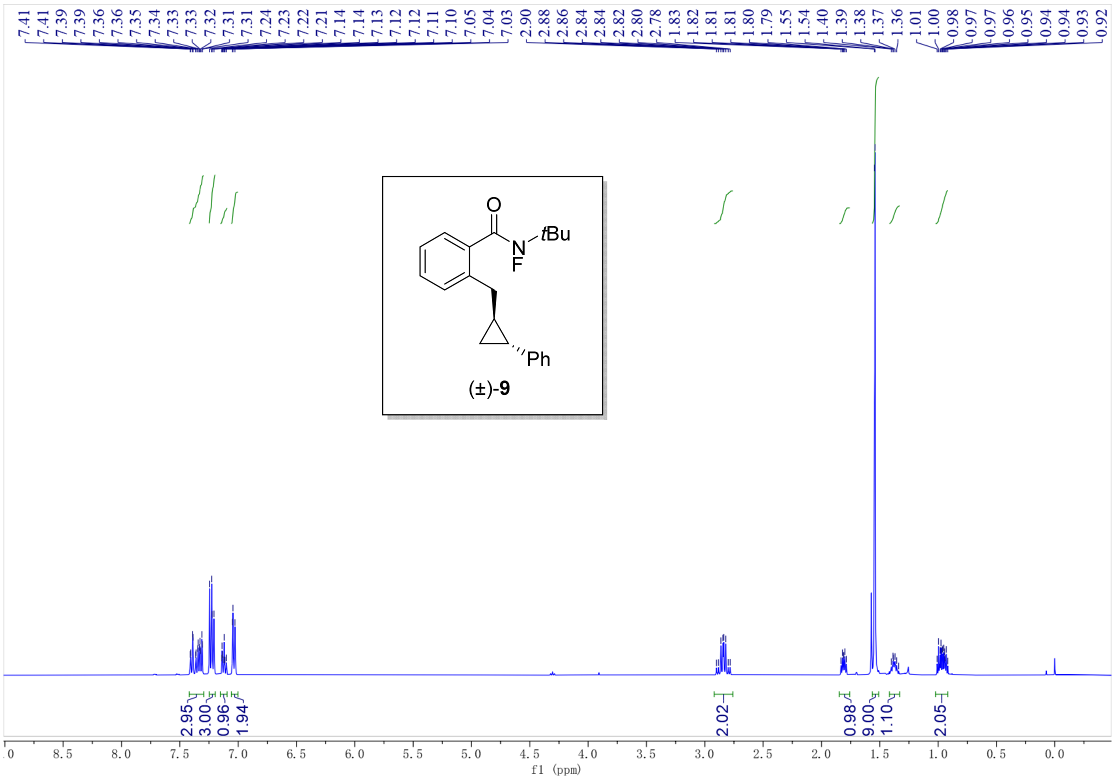

Supplementary Figure 176 ^{1}H NMR of **9**

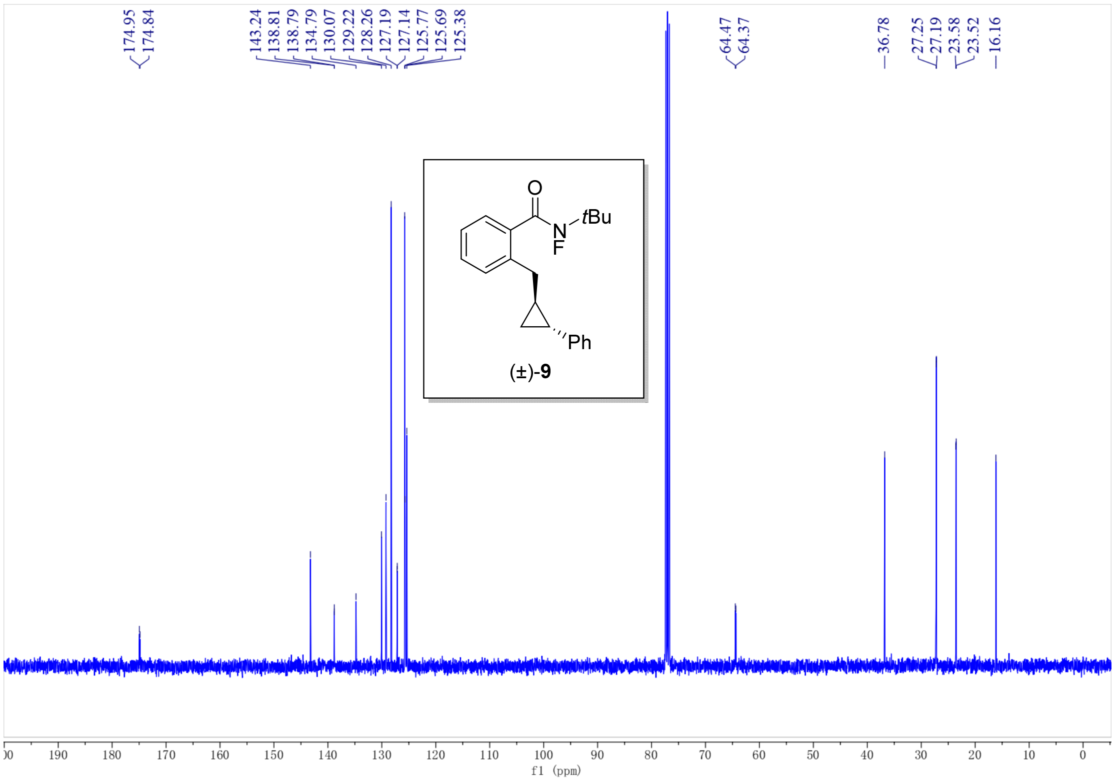

Supplementary Figure 177 ^{13}C NMR of **9**

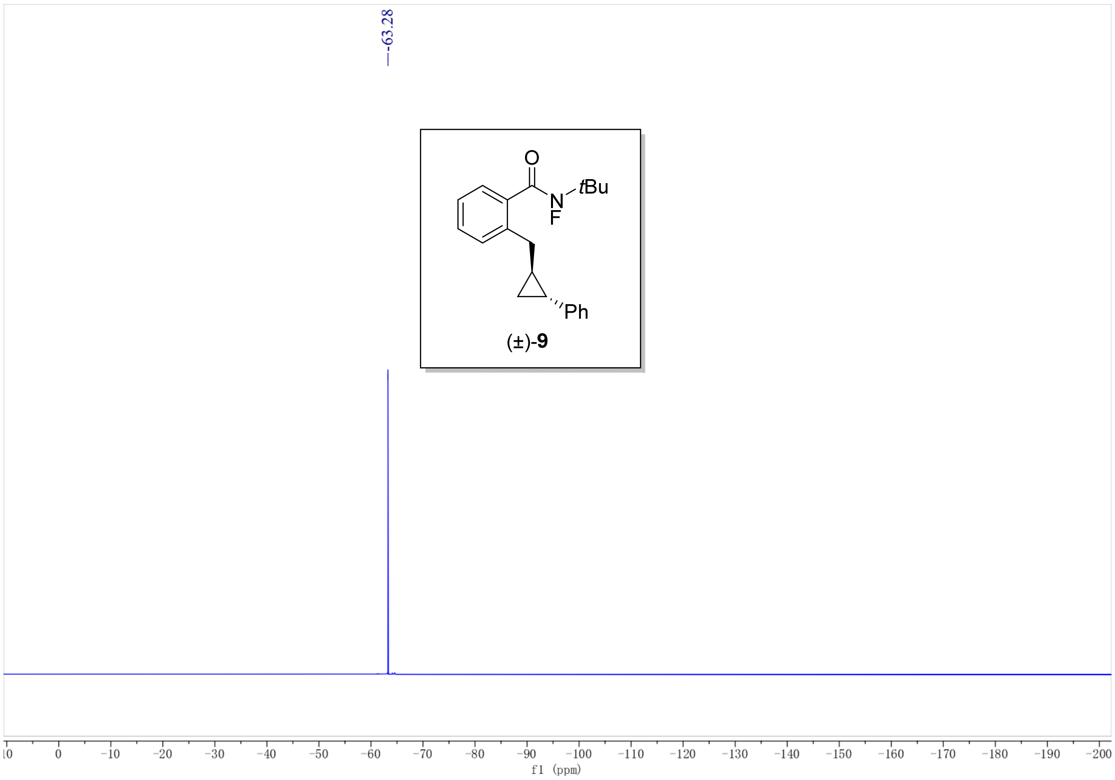

Supplementary Figure 178 ^{19}F NMR of **9**

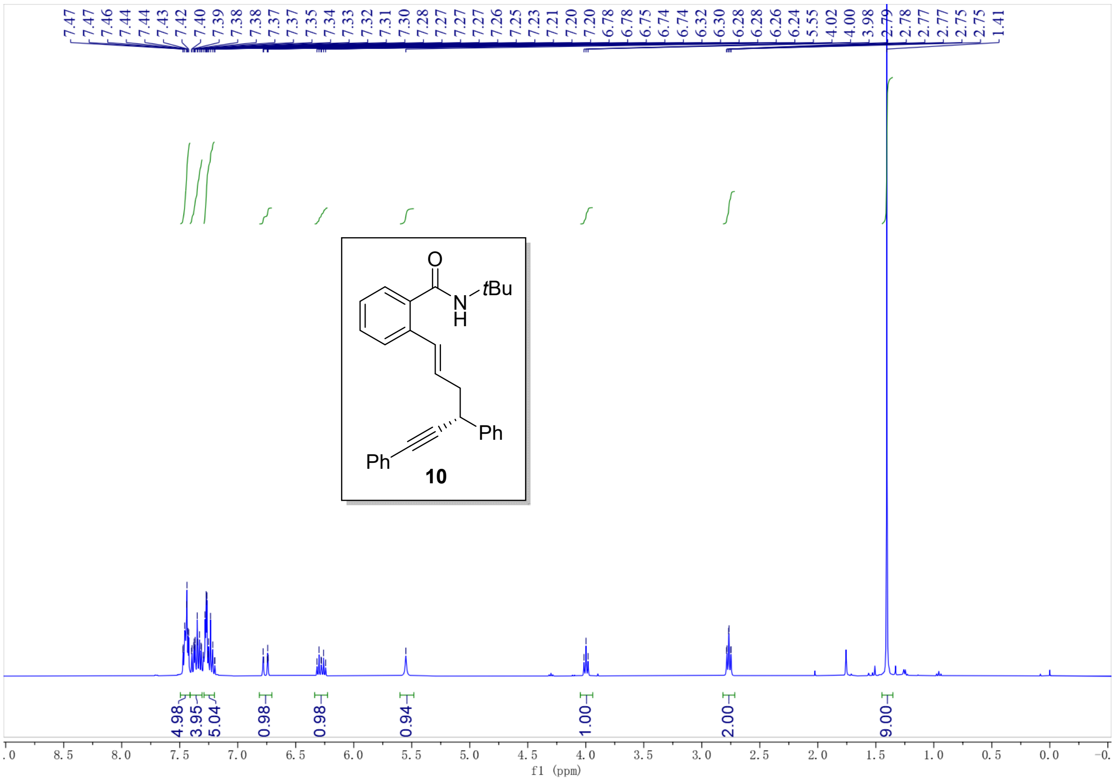

Supplementary Figure 179 ^{1}H NMR of **10**

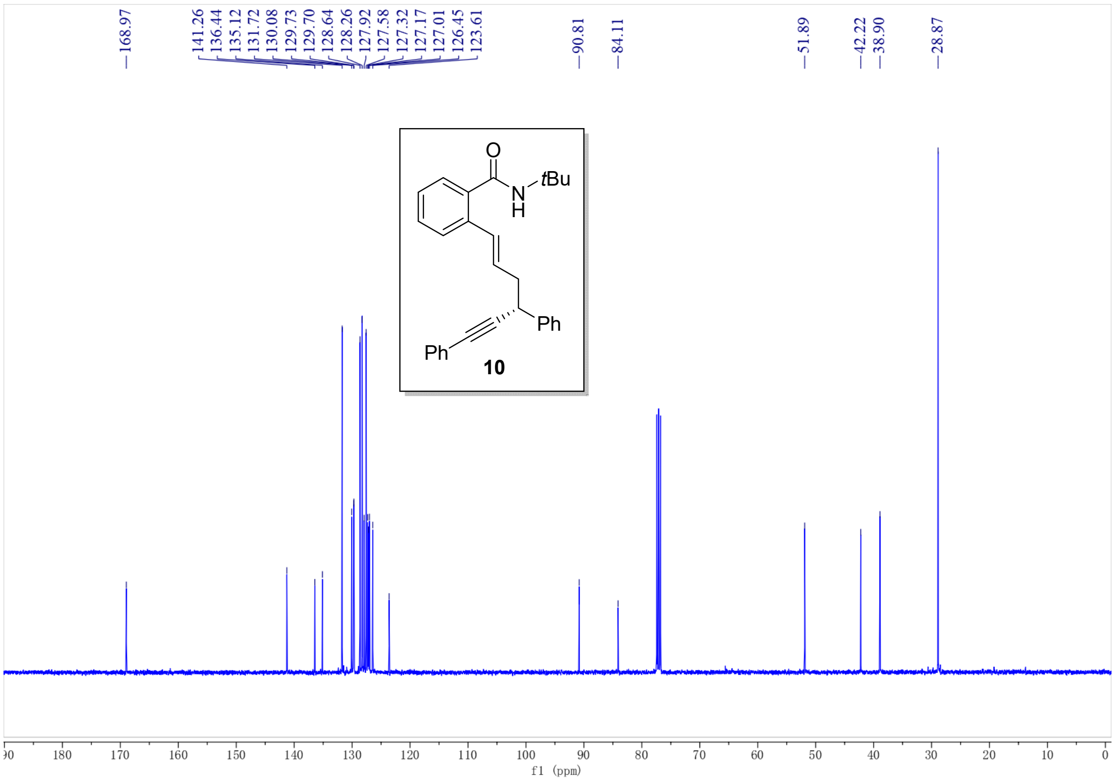

Supplementary Figure 180 ^{13}C NMR of **10**

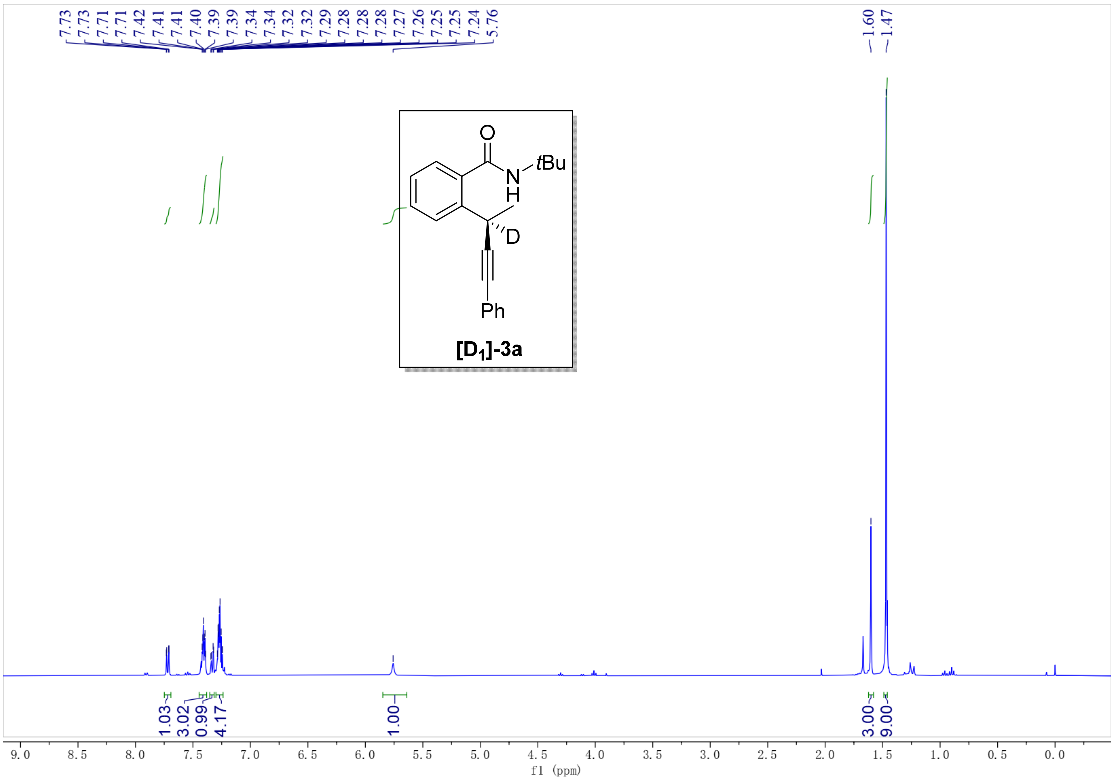

Supplementary Figure 181 ^{1}H NMR of **[D$_1$]-3a**

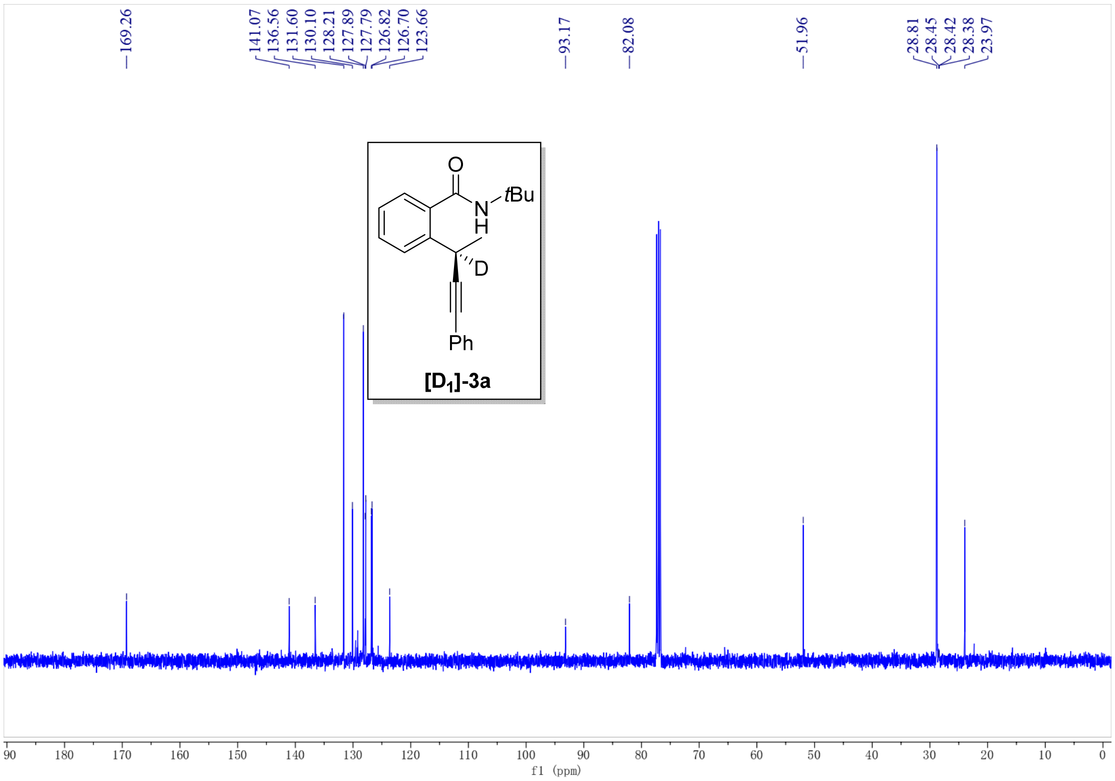

Supplementary Figure 182 ^{13}C NMR of **[D$_1$]-3a**

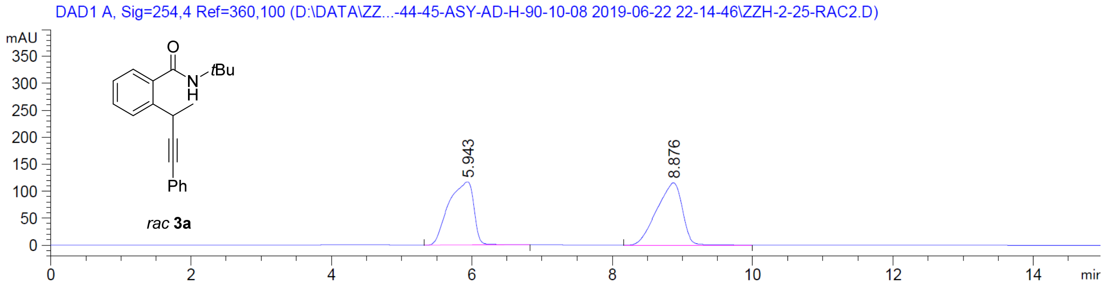

Signal 1: DAD1 A, Sig=254,4 Ref=360,100

Peak #	RetTime [min]	Type	Width [min]	Area [mAU*s]	Height [mAU]	Area %
1	5.943	BB	0.3358	2910.34253	117.15815	49.9144
2	8.876	BB	0.4213	2920.32690	115.36427	50.0856

Totals : 5830.66943 232.52242

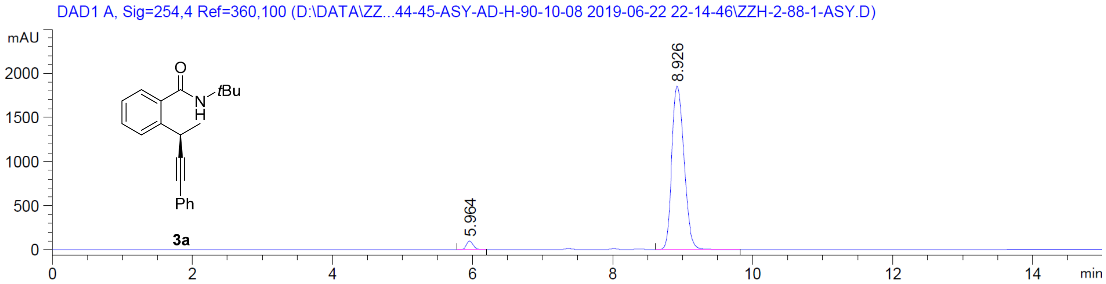

Signal 1: DAD1 A, Sig=254,4 Ref=360,100

Peak #	RetTime [min]	Type	Width [min]	Area [mAU*s]	Height [mAU]	Area %
1	5.964	VV	0.1071	681.42169	98.03461	2.9346
2	8.926	BV	0.1919	2.25392e4	1852.80945	97.0654

Totals : 2.32206e4 1950.84406

Supplementary Figure 183 HPLC spectra for racemic and chiral **3a**. **HPLC analysis: Chiralcel AD-H (n-hexane/i-PrOH = 90/10, flow rate 0.8 mL/min, λ = 254 nm), tR (minor) = 5.96 min, tR (major) = 8.93 min, 94% ee.**

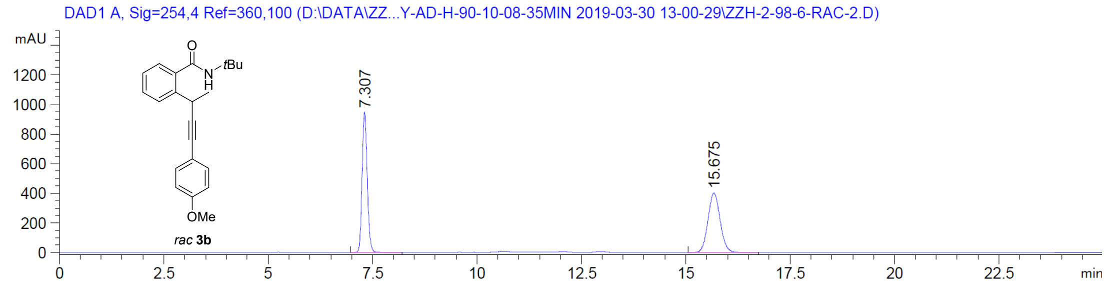

Signal 1: DAD1 A, Sig=254,4 Ref=360,100

Peak #	RetTime [min]	Type	Width [min]	Area [mAU*s]	Height [mAU]	Area %
1	7.307	BB	0.1314	8072.63428	947.80261	49.9081
2	15.675	BB	0.3136	8102.37402	401.16116	50.0919

Totals : 1.61750e4 1348.96378

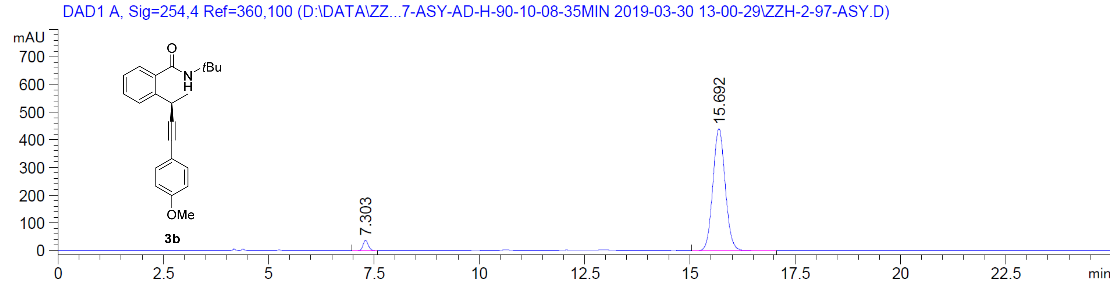

Signal 1: DAD1 A, Sig=254,4 Ref=360,100

Peak #	RetTime [min]	Type	Width [min]	Area [mAU*s]	Height [mAU]	Area %
1	7.303	BB	0.1329	322.71863	37.31412	3.4802
2	15.692	BB	0.3154	8950.29199	439.81516	96.5198

Totals : 9273.01062 477.12927

Supplementary Figure 184 HPLC spectra for racemic and chiral **3b**. **HPLC** analysis: Chiralcel AD-H (*n*-hexane/*i*-PrOH = 90/10, flow rate 0.8 mL/min, λ = 254 nm), t_R (minor) = 7.30 min, t_R (major) = 15.69 min, 93% ee.

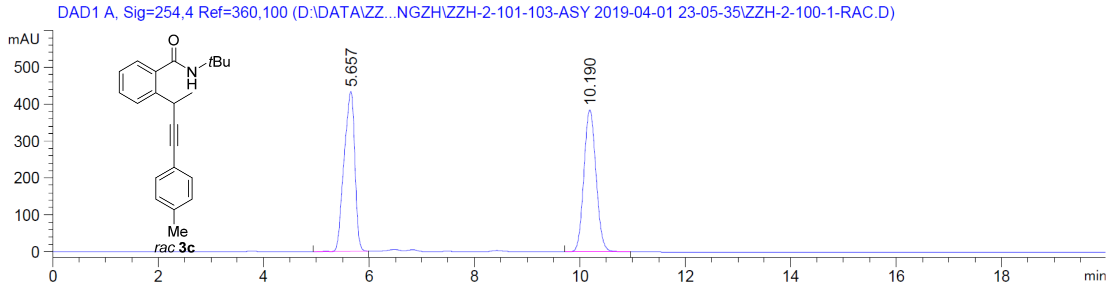

Signal 1: DAD1 A, Sig=254,4 Ref=360,100

Peak #	RetTime [min]	Type	Width [min]	Area [mAU*s]	Height [mAU]	Area %
1	5.657	BB	0.2403	6110.79395	433.93399	49.7760
2	10.190	BB	0.2506	6165.79395	385.46082	50.2240
Totals :				1.22766e4	819.39481	

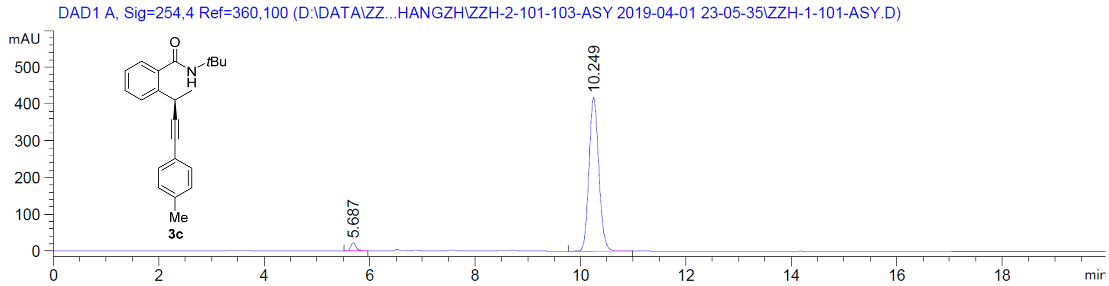

Signal 1: DAD1 A, Sig=254,4 Ref=360,100

Peak #	RetTime [min]	Type	Width [min]	Area [mAU*s]	Height [mAU]	Area %
1	5.687	BB	0.1049	157.79100	22.75697	2.8334
2	10.249	BB	0.1991	5411.21094	417.90417	97.1666
Totals :				5569.00194	440.66115	

Supplementary Figure 185 HPLC spectra for racemic and chiral **3c**. **HPLC** analysis: Chiralcel AD-H (*n*-hexane/*i*-PrOH = 90/10, flow rate 0.8 mL/min, λ = 254 nm), t_R (minor) = 5.69 min, t_R (major) = 10.25 min, 94% ee.

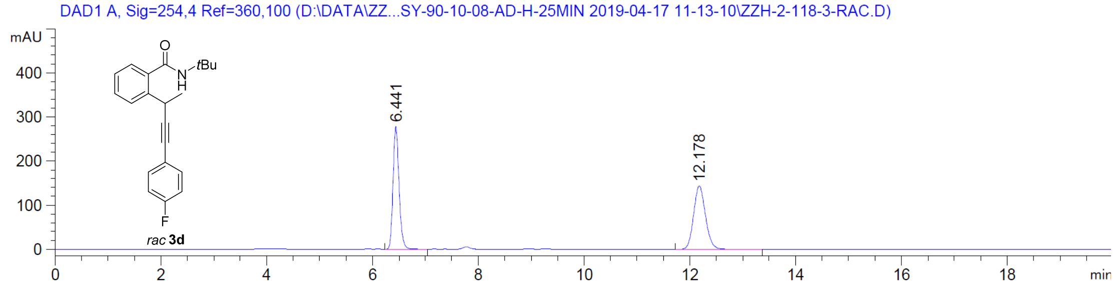

Signal 1: DAD1 A, Sig=254,4 Ref=360,100

Peak #	RetTime [min]	Type	Width [min]	Area [mAU*s]	Height [mAU]	Area %
1	6.441	BB	0.1222	2199.18164	278.14822	49.5947
2	12.178	BB	0.2417	2235.12378	143.52707	50.4053

Totals : 4434.30542 421.67529

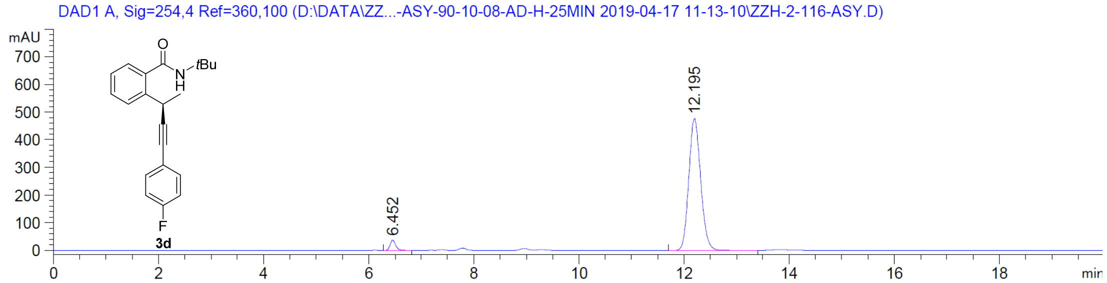

Signal 1: DAD1 A, Sig=254,4 Ref=360,100

Peak #	RetTime [min]	Type	Width [min]	Area [mAU*s]	Height [mAU]	Area %
1	6.452	BB	0.1142	279.57727	36.96203	3.6058
2	12.195	BB	0.2432	7473.99756	476.00305	96.3942

Totals : 7753.57483 512.96508

Supplementary Figure 186 HPLC spectra for racemic and chiral **3d**. **HPLC** analysis: Chiralcel AD-H (*n*-hexane/*i*-PrOH = 90/10, flow rate 0.8 mL/min, λ = 254 nm), t_R (minor) = 6.45 min, t_R (major) = 12.19 min, 93% ee.

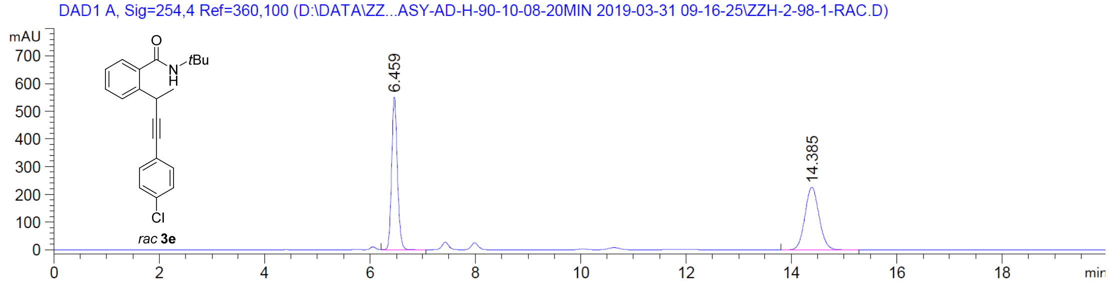

Signal 1: DAD1 A, Sig=254,4 Ref=360,100

Peak #	RetTime [min]	Type	Width [min]	Area [mAU*s]	Height [mAU]	Area %
1	6.459	VB	0.1169	4204.37646	551.75037	50.0770
2	14.385	BB	0.2866	4191.44336	225.72449	49.9230

Totals : 8395.81982 777.47485

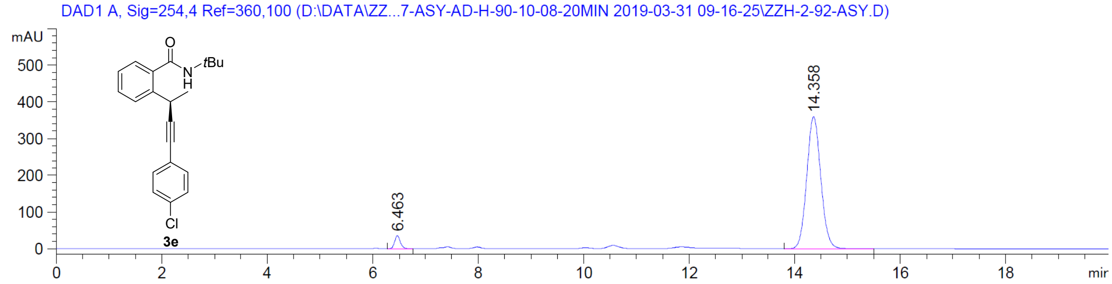

Signal 1: DAD1 A, Sig=254,4 Ref=360,100

Peak #	RetTime [min]	Type	Width [min]	Area [mAU*s]	Height [mAU]	Area %
1	6.463	VB	0.1148	263.11719	35.37709	3.8069
2	14.358	BB	0.2858	6648.39941	359.49002	96.1931

Totals : 6911.51660 394.86712

Supplementary Figure 187 HPLC spectra for racemic and chiral **3e**. **HPLC** analysis: Chiralcel AD-H (*n*-hexane/*i*-PrOH = 90/10, flow rate 0.8 mL/min, λ = 254 nm), t_R (minor) = 6.46 min, t_R (major) = 14.36 min, 92% ee.

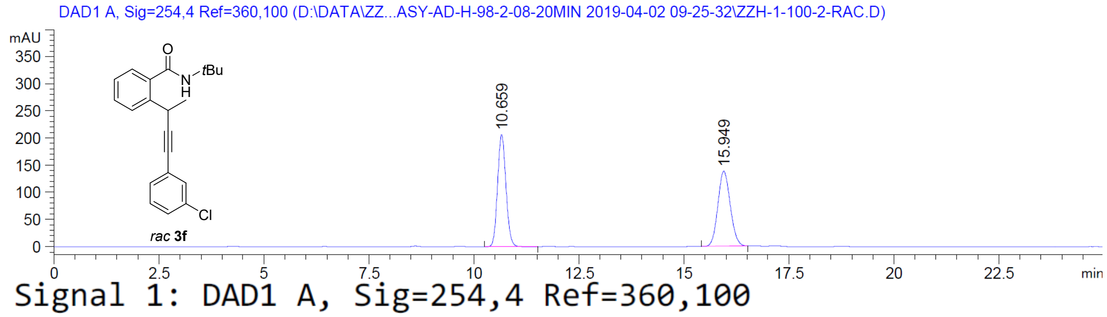

Signal 1: DAD1 A, Sig=254,4 Ref=360,100

Peak #	RetTime [min]	Type	Width [min]	Area [mAU*s]	Height [mAU]	Area %
1	10.659	BB	0.2161	2868.59106	206.51865	50.4112
2	15.949	BB	0.3185	2821.79517	138.05780	49.5888

Totals : 5690.38623 344.57645

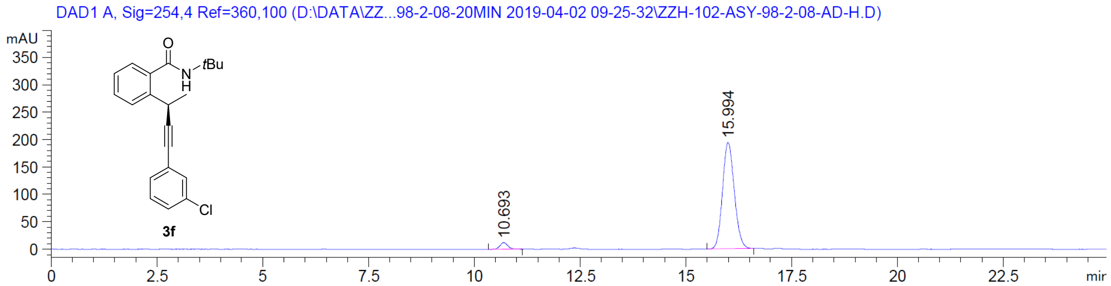

Signal 1: DAD1 A, Sig=254,4 Ref=360,100

Peak #	RetTime [min]	Type	Width [min]	Area [mAU*s]	Height [mAU]	Area %
1	10.693	BB	0.1951	153.26323	12.15823	3.9151
2	15.994	BB	0.2978	3761.40942	194.35658	96.0849

Totals : 3914.67265 206.51481

Supplementary Figure 188 HPLC spectra for racemic and chiral **3f**. **HPLC** analysis: Chiralcel AD-H (*n*-hexane/*i*-PrOH = 98/2, flow rate 0.8 mL/min, λ = 254 nm), t_R (minor) = 10.69 min, t_R (major) = 15.99 min, 92% ee.

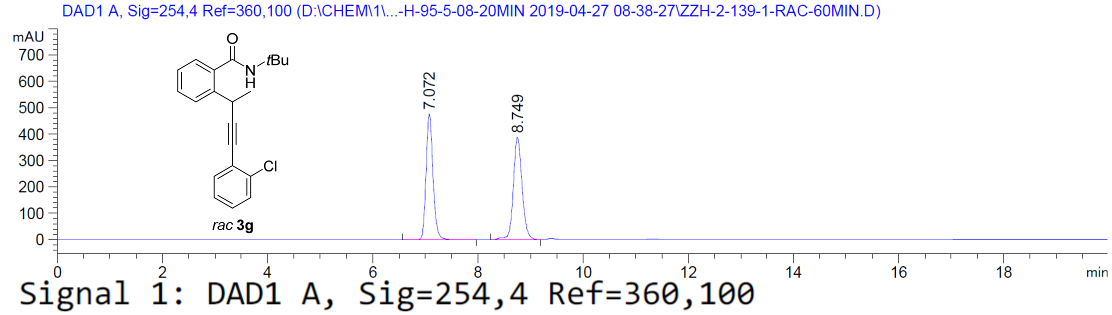

Signal 1: DAD1 A, Sig=254,4 Ref=360,100

Peak #	RetTime [min]	Type	Width [min]	Area [mAU*s]	Height [mAU]	Area %
1	7.072	BB	0.1430	4354.89746	474.90552	49.2257
2	8.749	VV	0.1753	4491.90479	387.19058	50.7743
Totals :				8846.80225	862.09610	

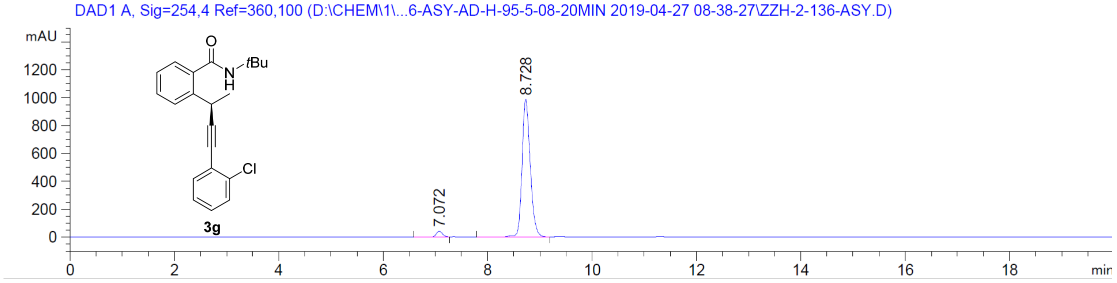

Signal 1: DAD1 A, Sig=254,4 Ref=360,100

Peak #	RetTime [min]	Type	Width [min]	Area [mAU*s]	Height [mAU]	Area %
1	7.072	BV	0.1314	363.04837	42.61234	3.2362
2	8.728	BV	0.1685	1.08555e4	985.88416	96.7638
Totals :				1.12185e4	1028.49649	

Supplementary Figure 189 HPLC spectra for racemic and chiral **3g**. **HPLC** analysis: Chiralcel AD-H (*n*-hexane/*i*-PrOH = 95/5, flow rate 0.8 mL/min, λ = 254 nm), t_R (minor) = 7.07 min, t_R (major) = 8.73 min, 93% ee.

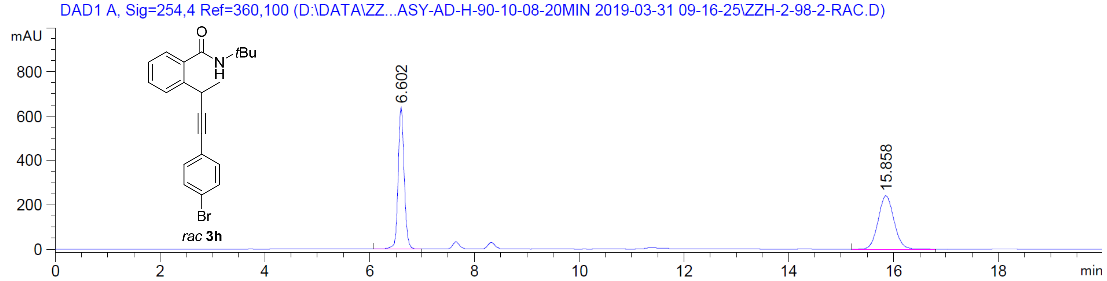

Signal 1: DAD1 A, Sig=254,4 Ref=360,100

Peak #	RetTime [min]	Type	Width [min]	Area [mAU*s]	Height [mAU]	Area %
1	6.602	BB	0.1229	5101.18115	640.47150	50.5365
2	15.858	BB	0.3224	4992.87402	240.33957	49.4635
Totals :				1.00941e4	880.81107	

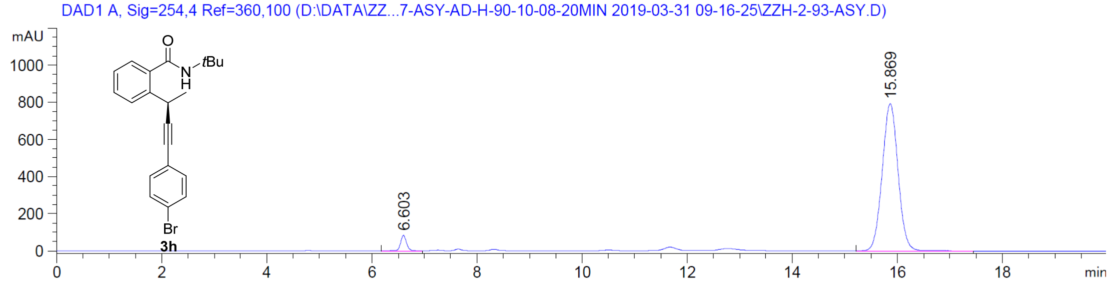

Signal 1: DAD1 A, Sig=254,4 Ref=360,100

Peak #	RetTime [min]	Type	Width [min]	Area [mAU*s]	Height [mAU]	Area %
1	6.603	BB	0.1189	651.59216	83.63357	3.7991
2	15.869	BB	0.3230	1.64995e4	792.12903	96.2009
Totals :				1.71511e4	875.76260	

Supplementary Figure 190 HPLC spectra for racemic and chiral **3h**. **HPLC** analysis: Chiralcel AD-H (*n*-hexane/*i*-PrOH = 90/10, flow rate 0.8 mL/min, λ = 254 nm), t_R (minor) = 6.60 min, t_R (major) = 15.87 min, 92% ee.

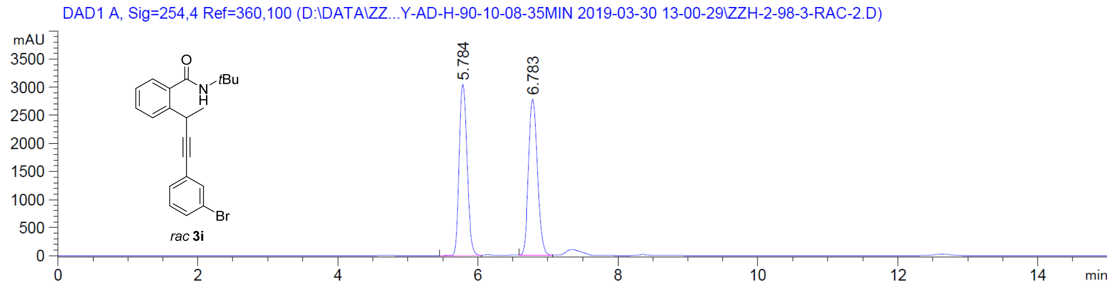

Signal 1: DAD1 A, Sig=254,4 Ref=360,100

Peak #	RetTime [min]	Type	Width [min]	Area [mAU*s]	Height [mAU]	Area %
1	5.784	BV	0.1228	2.36933e4	3043.35791	49.0592
2	6.783	VV	0.1392	2.46021e4	2780.25122	50.9408
Totals :				4.82954e4	5823.60913	

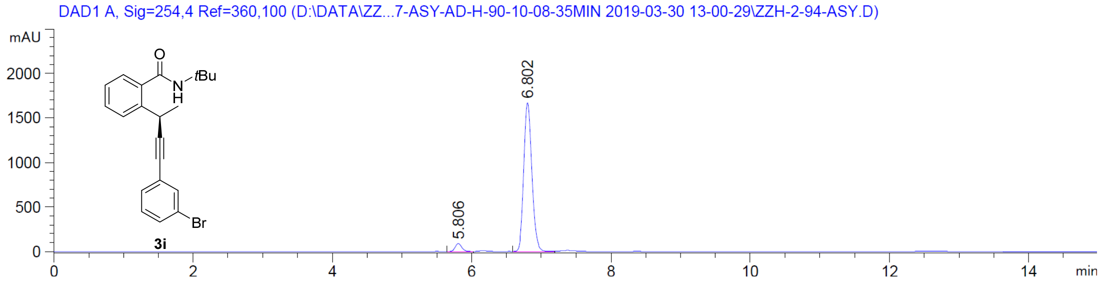

Signal 1: DAD1 A, Sig=254,4 Ref=360,100

Peak #	RetTime [min]	Type	Width [min]	Area [mAU*s]	Height [mAU]	Area %
1	5.806	BV	0.1012	583.71942	88.20984	4.1751
2	6.802	VV	0.1255	1.33971e4	1671.54565	95.8249
Totals :				1.39808e4	1759.75549	

Supplementary Figure 191 HPLC spectra for racemic and chiral **3i**. **HPLC** analysis: Chiralcel AD-H (*n*-hexane/*i*-PrOH = 90/10, flow rate 0.8 mL/min, λ = 254 nm), t_R (minor) = 5.81 min, t_R (major) = 6.80 min, 92% ee.

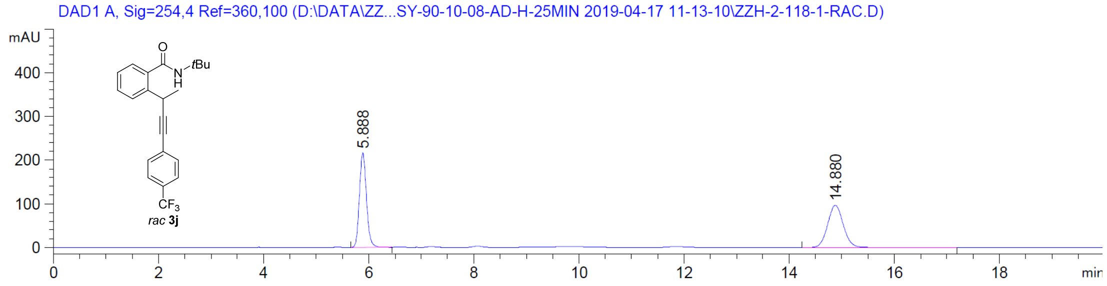

Signal 1: DAD1 A, Sig=254,4 Ref=360,100

Peak #	RetTime [min]	Type	Width [min]	Area [mAU*s]	Height [mAU]	Area %
1	5.888	BB	0.1442	2007.04370	216.41475	50.0773
2	14.880	BB	0.3214	2000.84534	96.70835	49.9227
Totals :				4007.88904	313.12310	

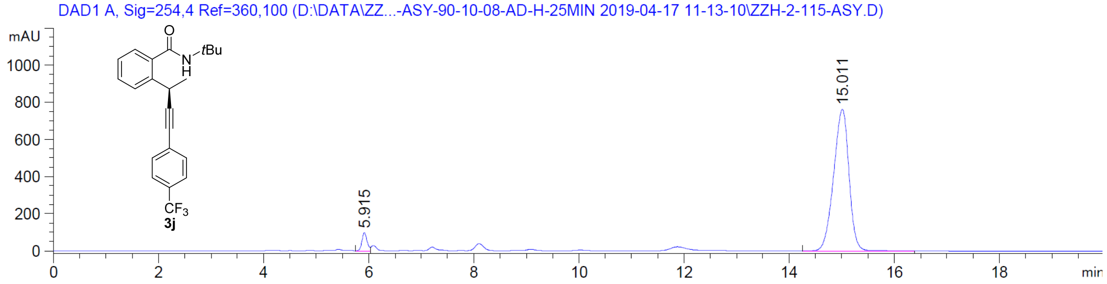

Signal 1: DAD1 A, Sig=254,4 Ref=360,100

Peak #	RetTime [min]	Type	Width [min]	Area [mAU*s]	Height [mAU]	Area %
1	5.915	BV	0.1025	654.16425	97.17193	4.0746
2	15.011	BB	0.3116	1.54006e4	762.59540	95.9254
Totals :				1.60548e4	859.76733	

Supplementary Figure 192 HPLC spectra for racemic and chiral **3j**. **HPLC** analysis: Chiralcel AD-H (*n*-hexane/*i*-PrOH = 90/10, flow rate 0.8 mL/min, λ = 254 nm), t_R (minor) = 5.92 min, t_R (major) = 15.01 min, 92% ee.

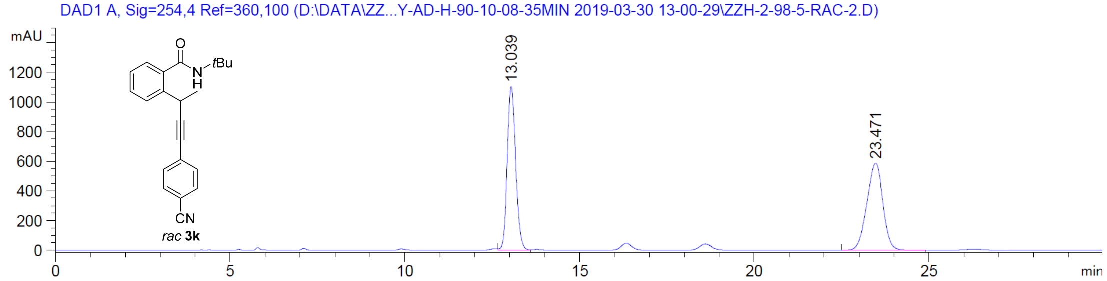

Signal 1: DAD1 A, Sig=254,4 Ref=360,100

Peak #	RetTime [min]	Type	Width [min]	Area [mAU*s]	Height [mAU]	Area %
1	13.039	VV	0.2598	1.85038e4	1102.47131	49.8750
2	23.471	BB	0.4933	1.85966e4	587.53174	50.1250

Totals : 3.71004e4 1690.00305

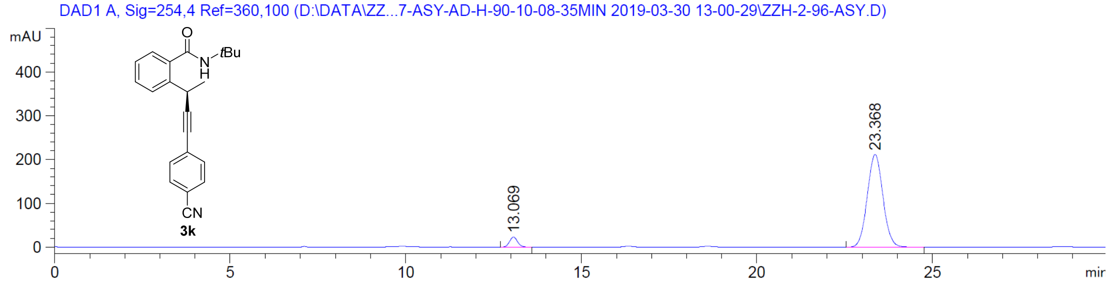

Signal 1: DAD1 A, Sig=254,4 Ref=360,100

Peak #	RetTime [min]	Type	Width [min]	Area [mAU*s]	Height [mAU]	Area %
1	13.069	BB	0.2467	366.11151	22.88248	5.2743
2	23.368	BB	0.4798	6575.30469	212.02191	94.7257

Totals : 6941.41620 234.90439

Supplementary Figure 193 HPLC spectra for racemic and chiral **3k**. **HPLC** analysis: Chiralcel AD-H (*n*-hexane/*i*-PrOH = 90/10, flow rate 0.8 mL/min, λ = 254 nm), t_R (minor) = 13.07 min, t_R (major) = 23.37 min, 89% ee.

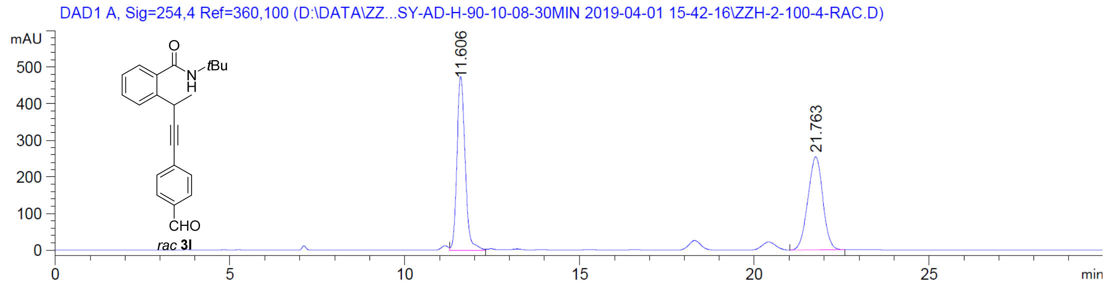

Signal 1: DAD1 A, Sig=254,4 Ref=360,100

Peak #	RetTime [min]	Type	Width [min]	Area [mAU*s]	Height [mAU]	Area %
1	11.606	VV	0.2490	7679.94189	474.14502	50.4314
2	21.763	BB	0.4601	7548.54053	254.44875	49.5686

Totals : 1.52285e4 728.59377

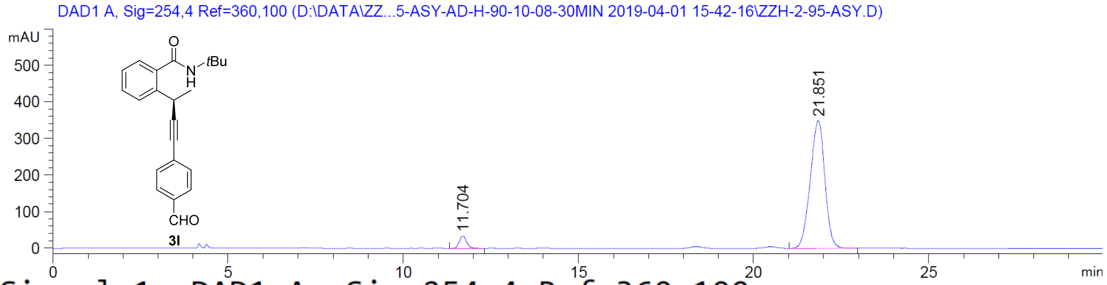

Signal 1: DAD1 A, Sig=254,4 Ref=360,100

Peak #	RetTime [min]	Type	Width [min]	Area [mAU*s]	Height [mAU]	Area %
1	11.704	BB	0.2210	489.27295	34.18181	4.6204
2	21.851	BB	0.4503	1.01001e4	348.33322	95.3796

Totals : 1.05894e4 382.51503

Supplementary Figure 194 HPLC spectra for racemic and chiral **3l**. **HPLC** analysis: Chiralcel AD-H (*n*-hexane/*i*-PrOH = 90/10, flow rate 0.8 mL/min, λ = 254 nm), t_R (minor) = 11.70 min, t_R (major) = 21.85 min, 91% ee.

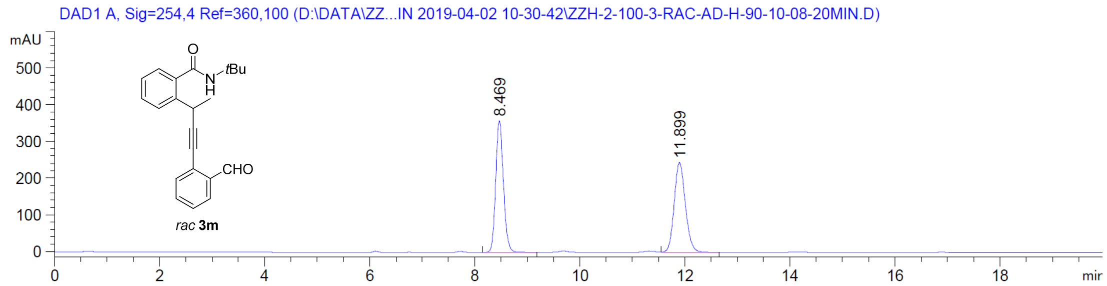

Signal 1: DAD1 A, Sig=254,4 Ref=360,100

Peak #	RetTime [min]	Type	Width [min]	Area [mAU*s]	Height [mAU]	Area %
1	8.469	BB	0.1570	3652.68652	358.19037	50.2625
2	11.899	VB	0.2284	3614.54004	244.61084	49.7375

Totals : 7267.22656 602.80121

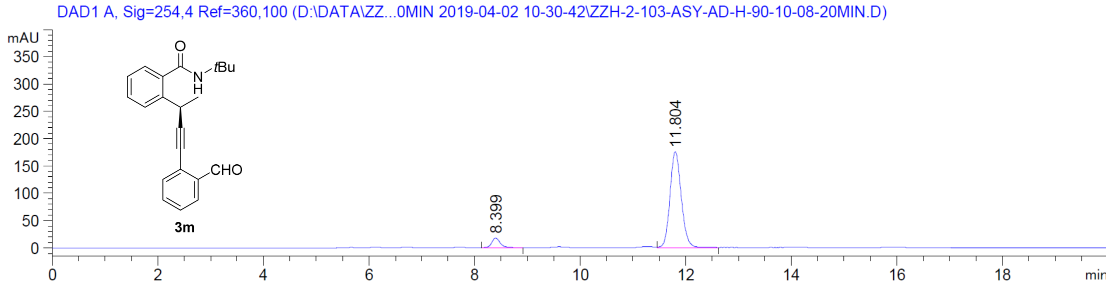

Signal 1: DAD1 A, Sig=254,4 Ref=360,100

Peak #	RetTime [min]	Type	Width [min]	Area [mAU*s]	Height [mAU]	Area %
1	8.399	BB	0.1634	182.23894	17.23092	6.6002
2	11.804	VB	0.2277	2578.88135	175.27034	93.3998

Totals : 2761.12029 192.50126

Supplementary Figure 195 HPLC spectra for racemic and chiral **3m**. **HPLC** analysis: Chiralcel AD-H (*n*-hexane/*i*-PrOH = 90/10, flow rate 0.8 mL/min, λ = 254 nm), t_R (minor) = 8.40 min, t_R (major) = 11.80 min, 87% ee.

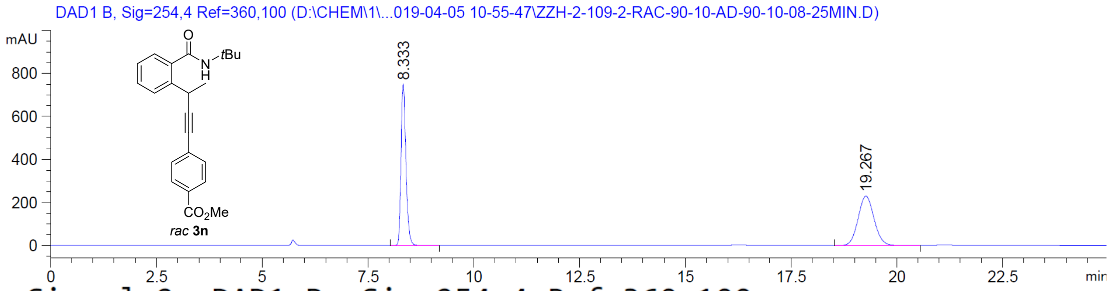

Signal 2: DAD1 B, Sig=254,4 Ref=360,100

Peak #	RetTime [min]	Type	Width [min]	Area [mAU*s]	Height [mAU]	Area %
1	8.333	VV	0.1260	6033.41943	748.89923	50.0259
2	19.267	VV	0.4055	6027.16748	230.17532	49.9741

Totals : 1.20606e4 979.07455

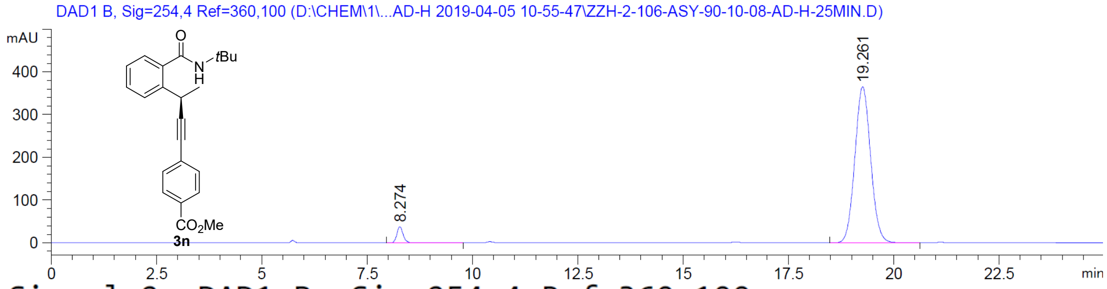

Signal 2: DAD1 B, Sig=254,4 Ref=360,100

Peak #	RetTime [min]	Type	Width [min]	Area [mAU*s]	Height [mAU]	Area %
1	8.274	VB	0.1628	395.72702	37.58322	3.9729
2	19.261	VV	0.4055	9565.05664	365.26340	96.0271

Totals : 9960.78366 402.84661

Supplementary Figure 196 HPLC spectra for racemic and chiral **3n**. **HPLC** analysis: Chiralcel AD-H (*n*-hexane/*i*-PrOH = 90/10, flow rate 0.8 mL/min, λ = 254 nm), t_R (minor) = 8.27 min, t_R (major) = 19.26 min, 92% ee.

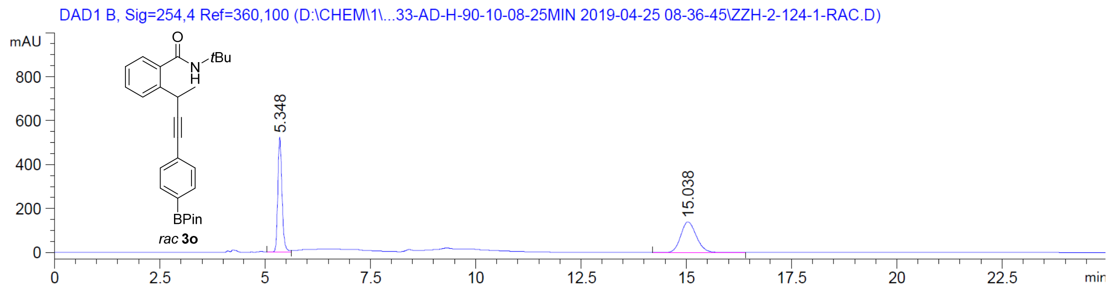

Signal 2: DAD1 B, Sig=254,4 Ref=360,100

Peak #	RetTime [min]	Type	Width [min]	Area [mAU*s]	Height [mAU]	Area %
1	5.348	BV	0.1131	3628.99683	522.17450	50.3195
2	15.038	BV	0.3964	3582.90771	139.16425	49.6805
Totals :				7211.90454	661.33875	

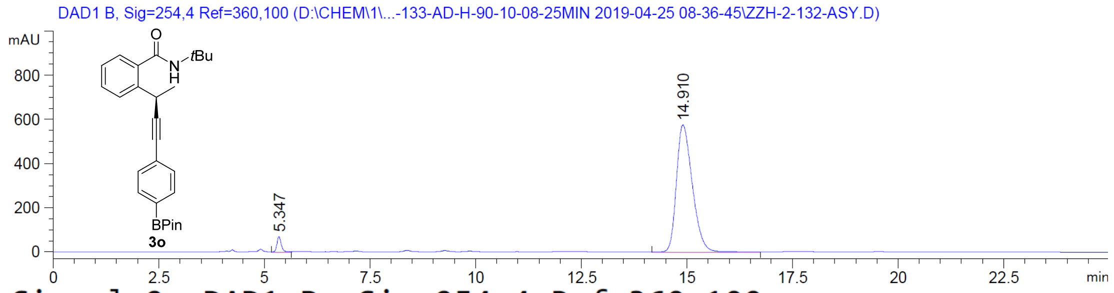

Signal 2: DAD1 B, Sig=254,4 Ref=360,100

Peak #	RetTime [min]	Type	Width [min]	Area [mAU*s]	Height [mAU]	Area %
1	5.347	BV	0.1082	474.53540	69.06821	3.0793
2	14.910	BV	0.3991	1.49362e4	574.88385	96.9207
Totals :				1.54107e4	643.95206	

Supplementary Figure 197 HPLC spectra for racemic and chiral **3o**. **HPLC** analysis: Chiralcel AD-H (*n*-hexane/*i*-PrOH = 90/10, flow rate 0.8 mL/min, λ = 254 nm), t_R (minor) = 5.35 min, t_R (major) = 14.91 min, 94% ee.

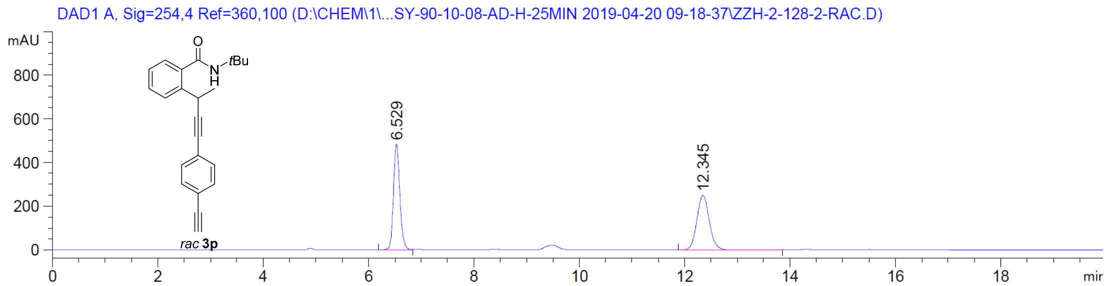

Signal 1: DAD1 A, Sig=254,4 Ref=360,100

Peak #	RetTime [min]	Type	Width [min]	Area [mAU*s]	Height [mAU]	Area %
1	6.529	BV	0.1261	4067.30103	483.75757	50.0881
2	12.345	VV	0.2492	4052.99927	249.94768	49.9119

Totals : 8120.30029 733.70525

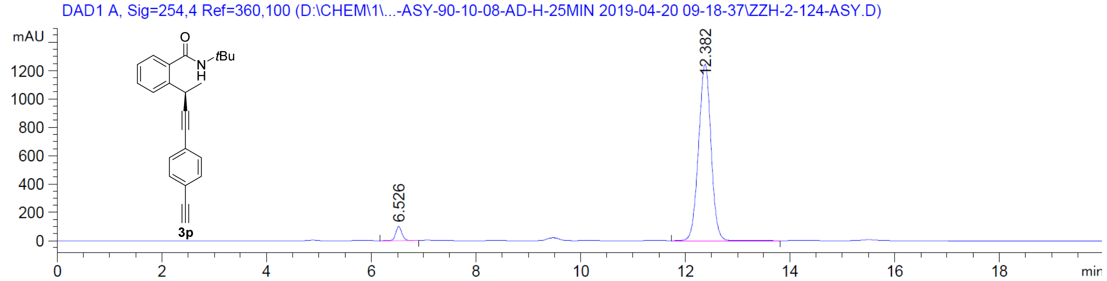

Signal 1: DAD1 A, Sig=254,4 Ref=360,100

Peak #	RetTime [min]	Type	Width [min]	Area [mAU*s]	Height [mAU]	Area %
1	6.526	BB	0.1264	840.41577	99.63342	3.9208
2	12.382	VV	0.2571	2.05944e4	1244.37427	96.0792

Totals : 2.14348e4 1344.00768

Supplementary Figure 198 HPLC spectra for racemic and chiral **3p**. **HPLC** analysis: Chiralcel AD-H (*n*-hexane/*i*-PrOH = 90/10, flow rate 0.8 mL/min, λ = 254 nm), t_R (minor) = 6.53 min, t_R (major) = 12.38 min, 92% ee.

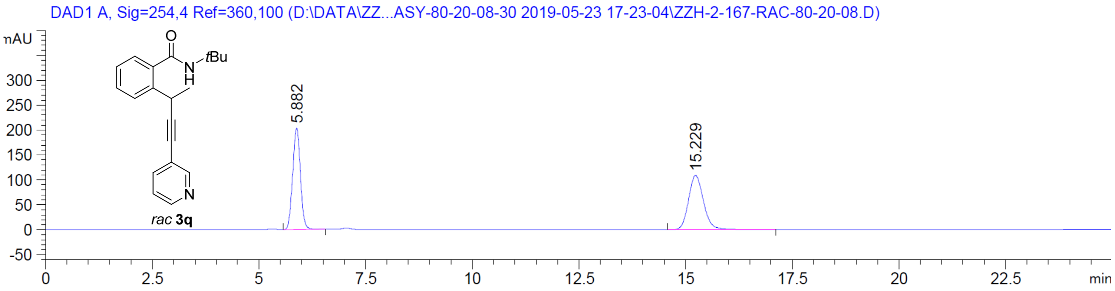

Signal 1: DAD1 A, Sig=254,4 Ref=360,100

Peak #	RetTime [min]	Type	Width [min]	Area [mAU*s]	Height [mAU]	Area %
1	5.882	BB	0.1950	2568.52881	203.79858	49.0300
2	15.229	BB	0.3759	2670.15405	108.94673	50.9700
Totals :				5238.68286	312.74532	

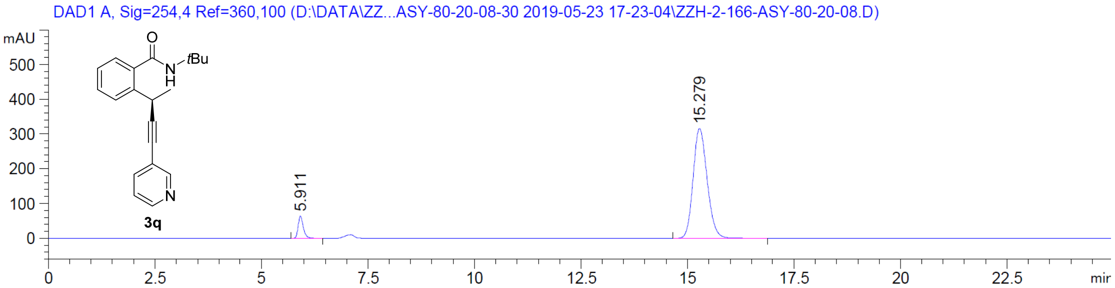

Signal 1: DAD1 A, Sig=254,4 Ref=360,100

Peak #	RetTime [min]	Type	Width [min]	Area [mAU*s]	Height [mAU]	Area %
1	5.911	BB	0.1283	536.31628	63.62977	6.9062
2	15.279	BB	0.3549	7229.45020	316.03403	93.0938
Totals :				7765.76648	379.66380	

Supplementary Figure 199 HPLC spectra for racemic and chiral **3q**. **HPLC** analysis: Chiralcel AD-H (*n*-hexane/*i*-PrOH = 80/20, flow rate 0.8 mL/min, λ = 254 nm), t_R (minor) = 5.91 min, t_R (major) = 15.28 min, 86% ee.

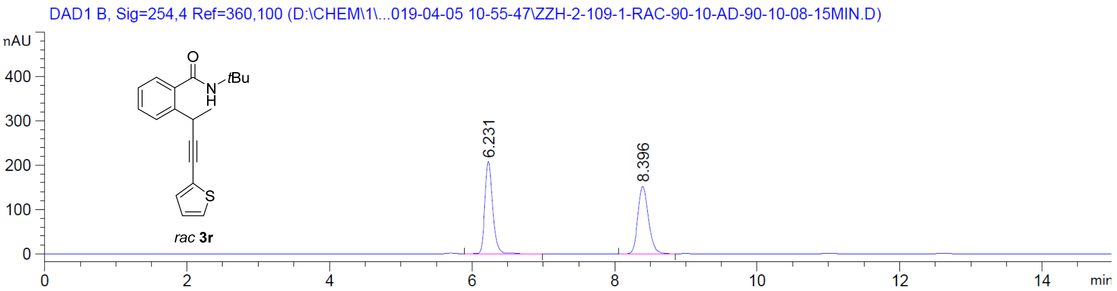

Signal 2: DAD1 B, Sig=254,4 Ref=360,100

Peak #	RetTime [min]	Type	Width [min]	Area [mAU*s]	Height [mAU]	Area %
1	6.231	VV	0.1216	1589.48315	207.05449	50.3620
2	8.396	BV	0.1643	1566.62976	151.90187	49.6380

Totals : 3156.11292 358.95636

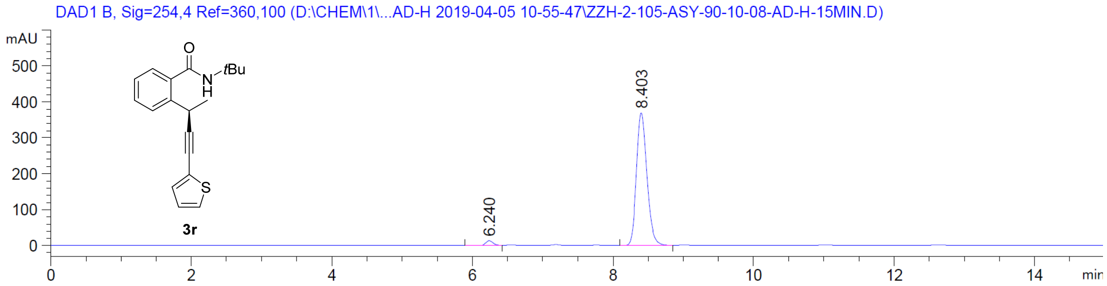

Signal 2: DAD1 B, Sig=254,4 Ref=360,100

Peak #	RetTime [min]	Type	Width [min]	Area [mAU*s]	Height [mAU]	Area %
1	6.240	VV	0.1196	100.92943	13.44950	2.5723
2	8.403	VV	0.1611	3822.72998	368.29492	97.4277

Totals : 3923.65941 381.74442

Supplementary Figure 200 HPLC spectra for racemic and chiral **3r**. **HPLC** analysis: Chiralcel AD-H (*n*-hexane/*i*-PrOH = 90/10, flow rate 0.8 mL/min, λ = 254 nm), t_R (minor) = 6.24 min, t_R (major) = 8.40 min, 95% ee.

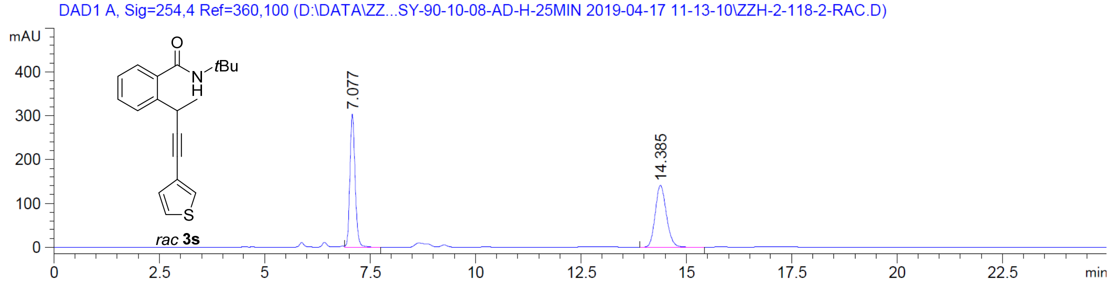

Signal 1: DAD1 A, Sig=254,4 Ref=360,100

Peak #	RetTime [min]	Type	Width [min]	Area [mAU*s]	Height [mAU]	Area %
1	7.077	VB	0.1321	2607.15845	303.77466	50.0038
2	14.385	BB	0.2852	2606.76343	141.33185	49.9962
Totals :				5213.92188	445.10651	

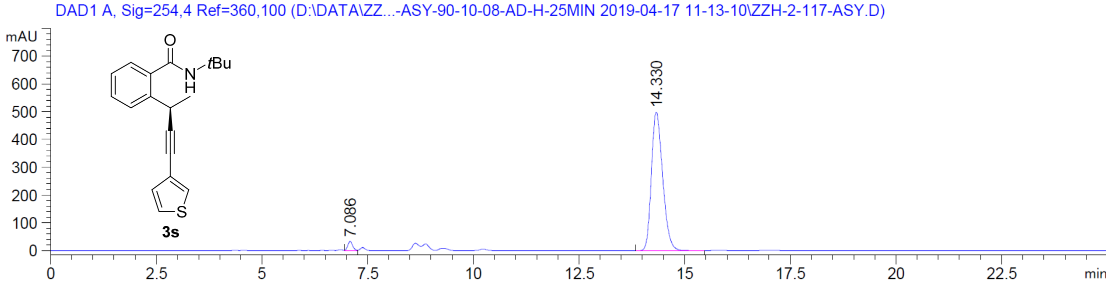

Signal 1: DAD1 A, Sig=254,4 Ref=360,100

Peak #	RetTime [min]	Type	Width [min]	Area [mAU*s]	Height [mAU]	Area %
1	7.086	VV	0.1218	268.52942	33.40827	2.8233
2	14.330	BB	0.2866	9242.80957	497.91702	97.1767
Totals :				9511.33899	531.32529	

Supplementary Figure 201 HPLC spectra for racemic and chiral **3s**. **HPLC** analysis: Chiralcel AD-H (*n*-hexane/*i*-PrOH = 90/10, flow rate 0.8 mL/min, λ = 254 nm), t_R (minor) = 7.09 min, t_R (major) = 14.33 min, 94% ee.

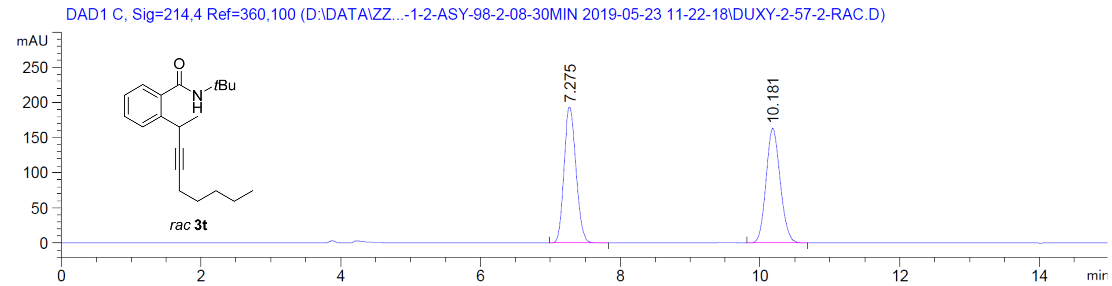

Signal 3: DAD1 C, Sig=214,4 Ref=360,100

Peak #	RetTime [min]	Type	Width [min]	Area [mAU*s]	Height [mAU]	Area %
1	7.275	BB	0.1872	2313.00098	193.92171	50.0208
2	10.181	BB	0.2206	2311.07397	163.82076	49.9792
Totals :				4624.07495	357.74246	

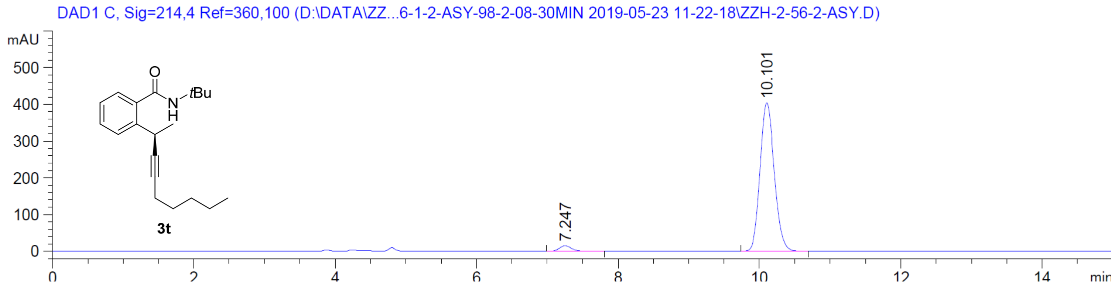

Signal 3: DAD1 C, Sig=214,4 Ref=360,100

Peak #	RetTime [min]	Type	Width [min]	Area [mAU*s]	Height [mAU]	Area %
1	7.247	BB	0.1888	180.85454	14.98338	3.1126
2	10.101	BB	0.2167	5629.51367	403.57828	96.8874
Totals :				5810.36821	418.56166	

Supplementary Figure 202 HPLC spectra for racemic and chiral **3t**. **HPLC** analysis: Chiralcel AD-H (*n*-hexane/*i*-PrOH = 98/2, flow rate 0.8 mL/min, λ = 214 nm), t_R (minor) = 7.25 min, t_R (major) = 10.10 min, 94% ee.

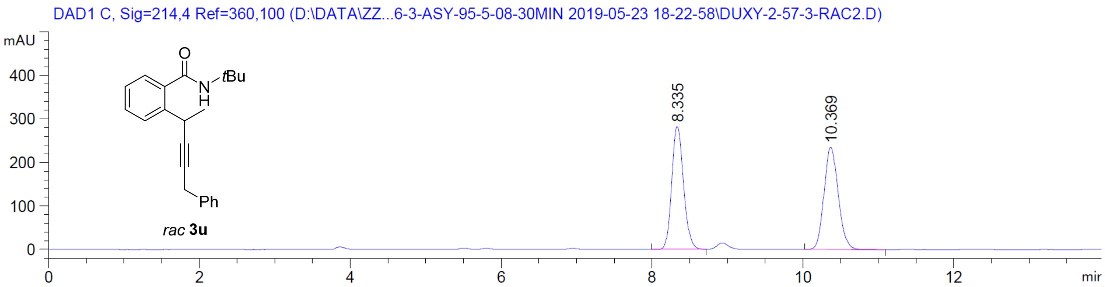

Signal 3: DAD1 C, Sig=214,4 Ref=360,100

Peak #	RetTime [min]	Type	Width [min]	Area [mAU*s]	Height [mAU]	Area %
1	8.335	BV	0.1690	3071.35986	282.14682	49.8586
2	10.369	BB	0.2030	3088.77710	235.53348	50.1414
Totals :				6160.13696	517.68030	

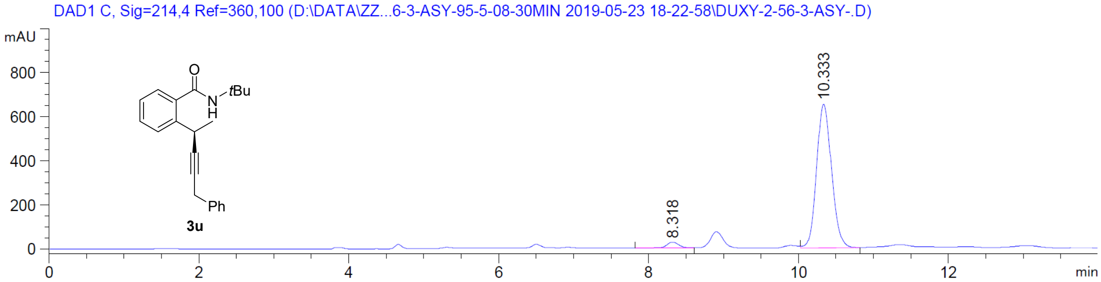

Signal 3: DAD1 C, Sig=214,4 Ref=360,100

Peak #	RetTime [min]	Type	Width [min]	Area [mAU*s]	Height [mAU]	Area %
1	8.318	BV	0.1850	333.60049	27.21399	3.5642
2	10.333	VB	0.2141	9026.22070	649.77014	96.4358
Totals :				9359.82120	676.98413	

Supplementary Figure 203 HPLC spectra for racemic and chiral **3u**. **HPLC** analysis: Chiralcel AD-H (*n*-hexane/*i*-PrOH = 95/5, flow rate 0.8 mL/min, λ = 214 nm), t_R (minor) = 8.32 min, t_R (major) = 10.33 min, 93% ee.

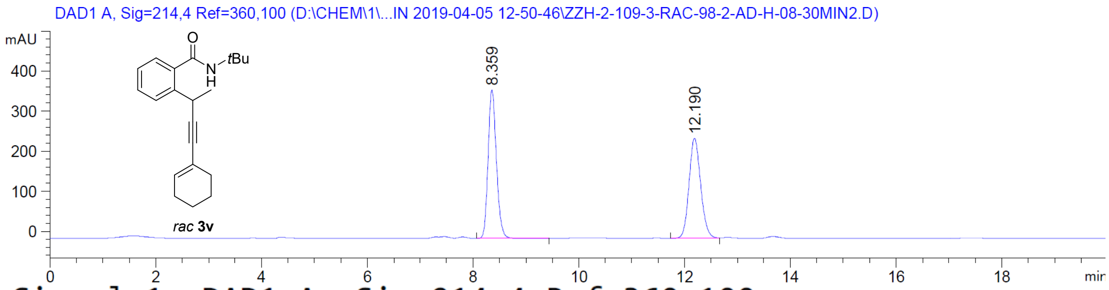

Signal 1: DAD1 A, Sig=214,4 Ref=360,100

Peak #	RetTime [min]	Type	Width [min]	Area [mAU*s]	Height [mAU]	Area %
1	8.359	BB	0.1679	4051.36938	369.50290	51.4057
2	12.190	BV	0.2392	3829.79834	249.37503	48.5943
Totals :				7881.16772	618.87793	

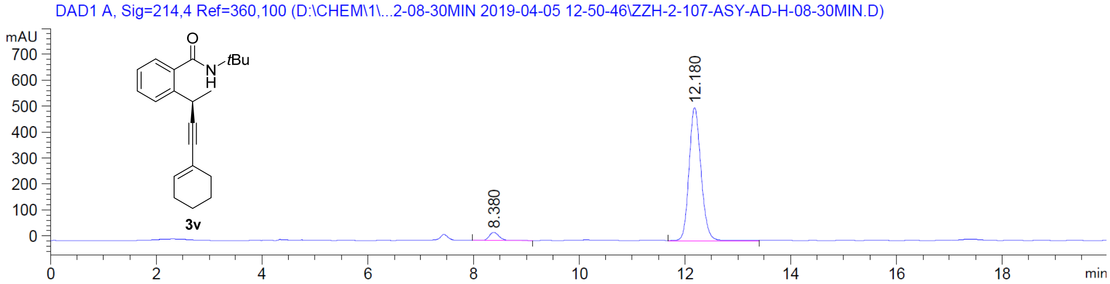

Signal 1: DAD1 A, Sig=214,4 Ref=360,100

Peak #	RetTime [min]	Type	Width [min]	Area [mAU*s]	Height [mAU]	Area %
1	8.380	BB	0.2020	399.70309	31.09023	4.7519
2	12.180	VV	0.2425	8011.64893	512.19305	95.2481
Totals :				8411.35202	543.28329	

Supplementary Figure 204 HPLC spectra for racemic and chiral **3v**. **HPLC** analysis: Chiralcel AD-H (*n*-hexane/*i*-PrOH = 98/2, flow rate 0.8 mL/min, λ = 214 nm), t_R (minor) = 8.38 min, t_R (major) = 12.18 min, 90% ee.

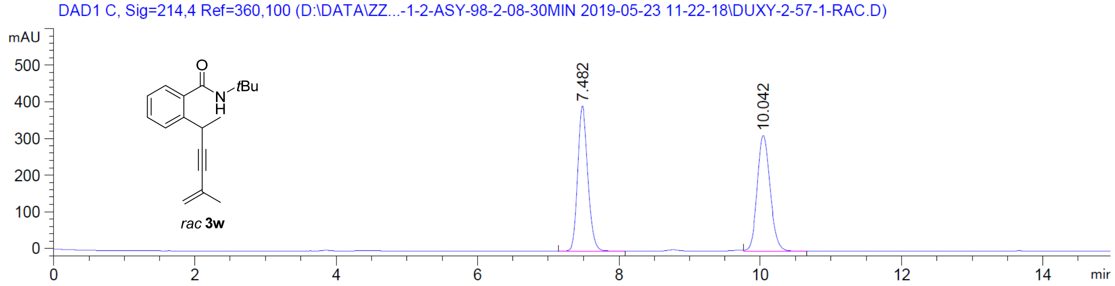

Signal 3: DAD1 C, Sig=214,4 Ref=360,100

Peak #	RetTime [min]	Type	Width [min]	Area [mAU*s]	Height [mAU]	Area %
1	7.482	BB	0.1549	3972.04346	396.56424	49.8320
2	10.042	VB	0.1957	3998.82251	315.92395	50.1680
Totals :				7970.86597	712.48819	

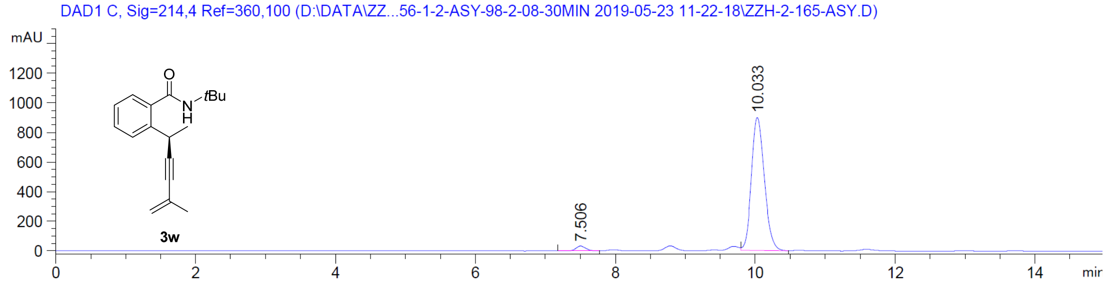

Signal 3: DAD1 C, Sig=214,4 Ref=360,100

Peak #	RetTime [min]	Type	Width [min]	Area [mAU*s]	Height [mAU]	Area %
1	7.506	BV	0.1558	336.45709	33.31292	2.8356
2	10.033	VV	0.1976	1.15292e4	899.01721	97.1644
Totals :				1.18657e4	932.33013	

Supplementary Figure 205 HPLC spectra for racemic and chiral **3w**. **HPLC** analysis: Chiralcel AD-H (*n*-hexane/*i*-PrOH = 98/2, flow rate 0.8 mL/min, λ = 214 nm), t_R (minor) = 7.51 min, t_R (major) = 10.03 min, 94% ee.

DAD1 C, Sig=214,4 Ref=360,100 (D:\DATA\ZZ...SY-AD-H-95-5-08-30MIN 2019-05-15 15-58-21\ZZH-2-110-1-RAC-.D)

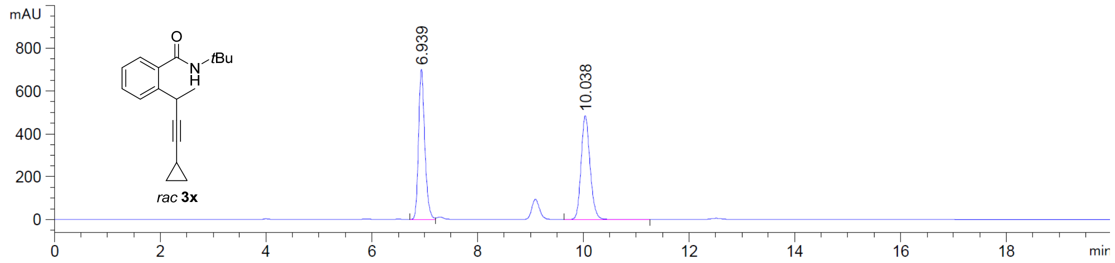

Signal 3: DAD1 C, Sig=214,4 Ref=360,100

Peak #	RetTime [min]	Type	Width [min]	Area [mAU*s]	Height [mAU]	Area %
1	6.939	VV	0.1241	5777.97900	701.26483	50.2953
2	10.038	BB	0.1815	5710.13916	484.40717	49.7047

Totals : 1.14881e4 1185.67200

DAD1 C, Sig=214,4 Ref=360,100 (D:\DATA\ZZ...161ASY-AD-H-95-5-08-30MIN 2019-05-15 15-58-21\ZZH-159-ASY-.D)

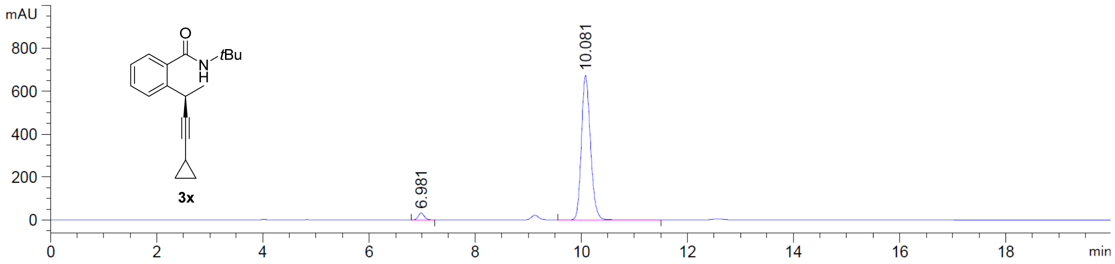

Signal 3: DAD1 C, Sig=214,4 Ref=360,100

Peak #	RetTime [min]	Type	Width [min]	Area [mAU*s]	Height [mAU]	Area %
1	6.981	BB	0.1205	256.05124	33.02571	3.0946
2	10.081	BB	0.1826	8017.95850	674.86096	96.9054

Totals : 8274.00974 707.88667

Supplementary Figure 206 HPLC spectra for racemic and chiral **3x**. **HPLC** analysis: Chiralcel AD-H (*n*-hexane/*i*-PrOH = 95/5, flow rate 0.8 mL/min, λ = 214 nm), t_R (minor) = 6.98 min, t_R (major) = 10.08 min, 94% ee.

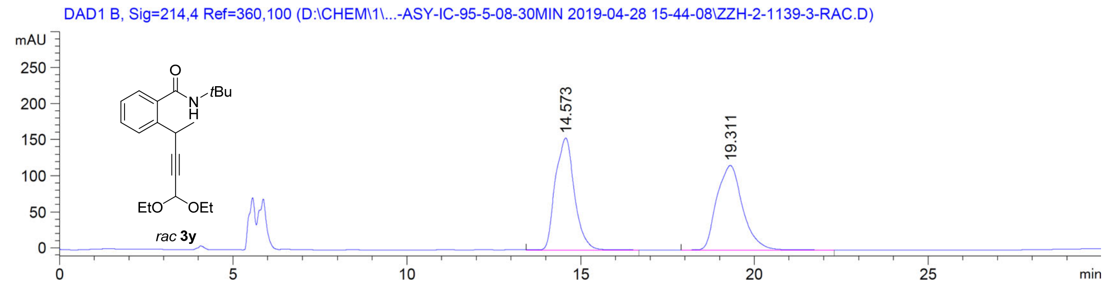

Signal 2: DAD1 B, Sig=214,4 Ref=360,100

Peak #	RetTime [min]	Type	Width [min]	Area [mAU*s]	Height [mAU]	Area %
1	14.573	VB	0.5378	5924.29590	155.03220	49.4853
2	19.311	BB	0.8252	6047.53223	117.36452	50.5147
Totals :				1.19718e4	272.39672	

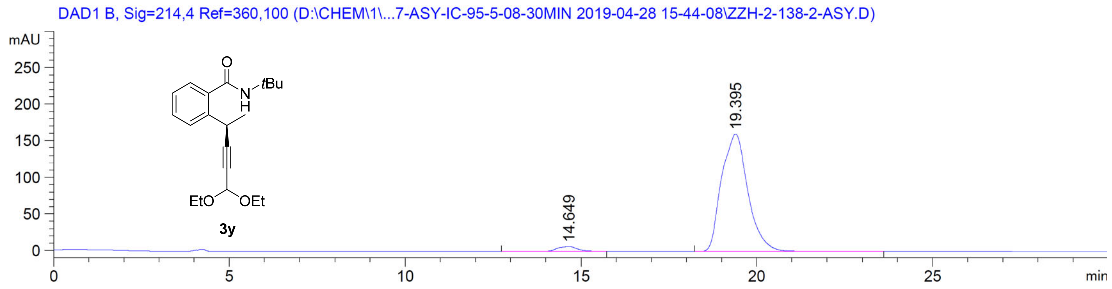

Signal 2: DAD1 B, Sig=214,4 Ref=360,100

Peak #	RetTime [min]	Type	Width [min]	Area [mAU*s]	Height [mAU]	Area %
1	14.649	BB	0.5582	274.41687	6.80877	3.1757
2	19.395	BB	0.7326	8366.66699	160.38777	96.8243
Totals :				8641.08386	167.19654	

Supplementary Figure 207 HPLC spectra for racemic and chiral **3y**. **HPLC** analysis: Chiralcel IC (*n*-hexane/*i*-PrOH = 95/5, flow rate 0.8 mL/min, λ = 214 nm), t_R (minor) = 14.65 min, t_R (major) = 19.40 min, 94% ee.

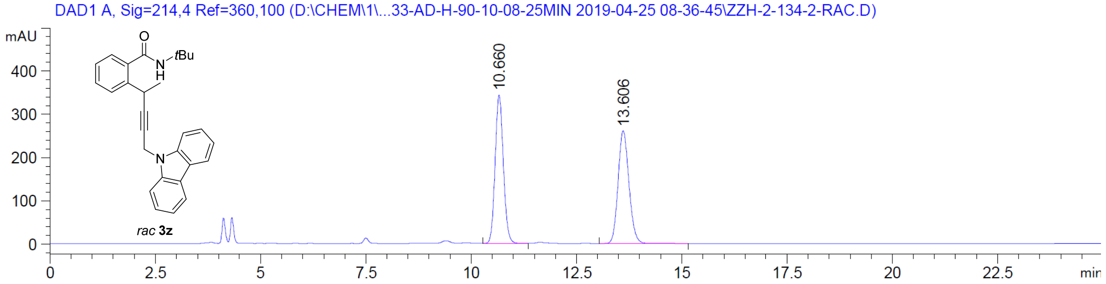

Signal 1: DAD1 A, Sig=214,4 Ref=360,100

Peak #	RetTime [min]	Type	Width [min]	Area [mAU*s]	Height [mAU]	Area %
1	10.660	BV	0.2109	4661.41553	342.29874	49.8675
2	13.606	BV	0.2780	4686.18750	260.40308	50.1325

Totals : 9347.60303 602.70181

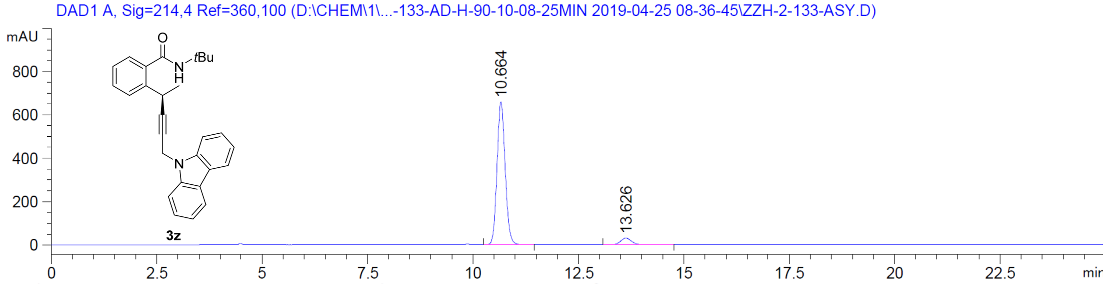

Signal 1: DAD1 A, Sig=214,4 Ref=360,100

Peak #	RetTime [min]	Type	Width [min]	Area [mAU*s]	Height [mAU]	Area %
1	10.664	BV	0.2048	8828.30566	656.84717	94.2907
2	13.626	BV	0.2710	534.54926	30.12840	5.7093

Totals : 9362.85492 686.97557

Supplementary Figure 208 HPLC spectra for racemic and chiral **3z**. **HPLC** analysis: Chiralcel AD-H (*n*-hexane/*i*-PrOH = 90/10, flow rate 0.8 mL/min, λ = 214 nm), t_R (major) = 10.66 min, t_R (minor) = 13.63 min, 89% ee.

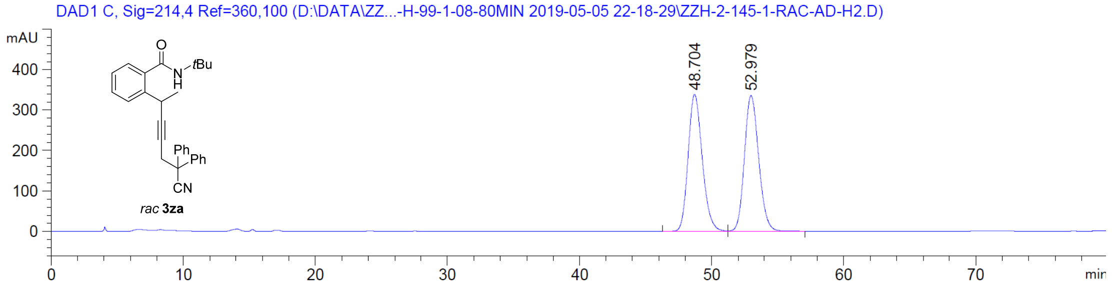

Signal 3: DAD1 C, Sig=214,4 Ref=360,100

Peak #	RetTime [min]	Type	Width [min]	Area [mAU*s]	Height [mAU]	Area %
1	48.704	BV	1.1449	2.51372e4	338.29974	49.9748
2	52.979	VB	1.1620	2.51625e4	335.12097	50.0252
Totals :				5.02996e4	673.42072	

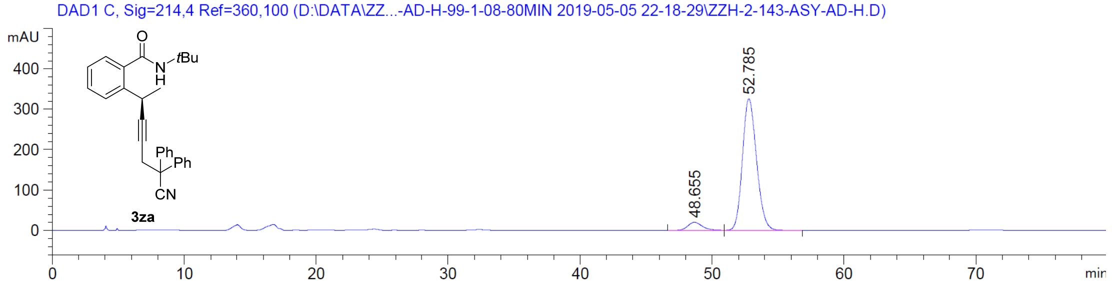

Signal 3: DAD1 C, Sig=214,4 Ref=360,100

Peak #	RetTime [min]	Type	Width [min]	Area [mAU*s]	Height [mAU]	Area %
1	48.655	BB	1.1131	1494.55237	19.83427	5.8422
2	52.785	BB	1.1516	2.40873e4	325.39319	94.1578
Totals :				2.55819e4	345.22746	

Supplementary Figure 209 HPLC spectra for racemic and chiral **3za**. **HPLC** analysis: Chiralcel AD-H (*n*-hexane/*i*-PrOH = 99/1, flow rate 0.8 mL/min, λ = 214 nm), t_R (minor) = 48.66 min, t_R (major) = 52.79 min, 88% ee.

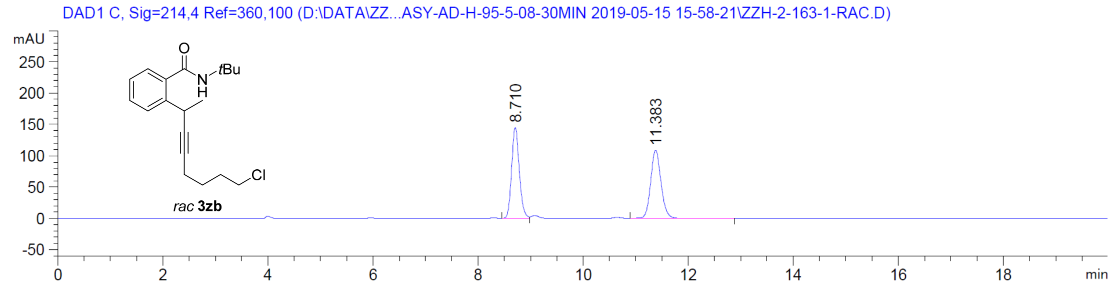

Signal 3: DAD1 C, Sig=214,4 Ref=360,100

Peak #	RetTime [min]	Type	Width [min]	Area [mAU*s]	Height [mAU]	Area %
1	8.710	VV	0.1566	1475.95056	145.24469	49.7225
2	11.383	BB	0.2099	1492.42676	108.86433	50.2775
Totals :				2968.37732	254.10902	

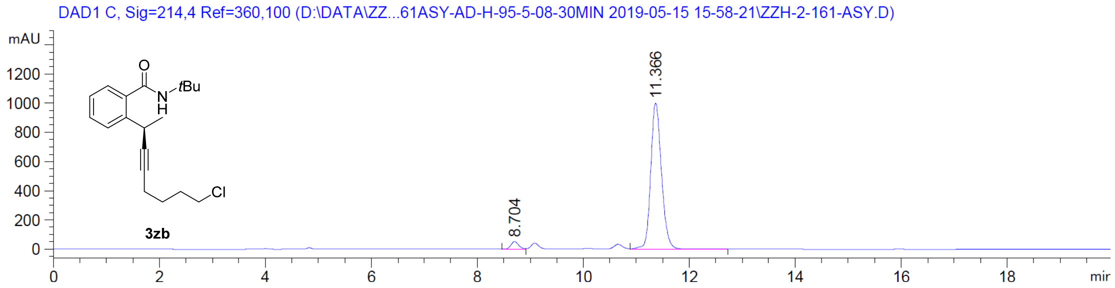

Signal 3: DAD1 C, Sig=214,4 Ref=360,100

Peak #	RetTime [min]	Type	Width [min]	Area [mAU*s]	Height [mAU]	Area %
1	8.704	BV	0.1569	528.73047	51.86445	3.6122
2	11.366	VB	0.2163	1.41086e4	1001.79272	96.3878
Totals :				1.46373e4	1053.65717	

Supplementary Figure 210 HPLC spectra for racemic and chiral **3zb**. **HPLC** analysis: Chiralcel AD-H (*n*-hexane/*i*-PrOH = 95/5, flow rate 0.8 mL/min, λ = 214 nm), t_R (minor) = 8.70 min, t_R (major) = 11.37 min, 93% ee.

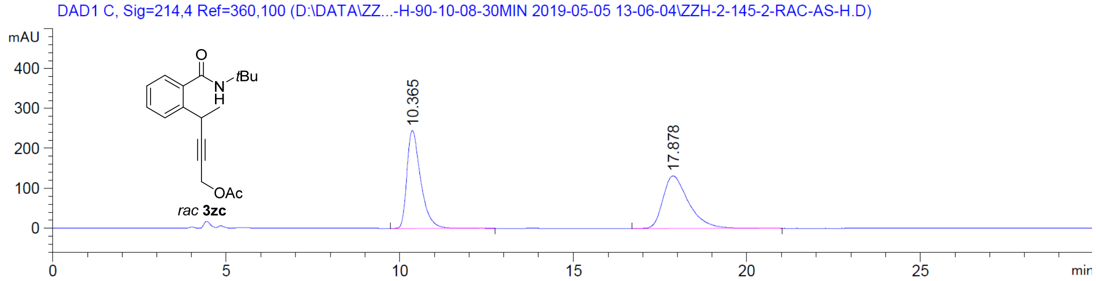

Signal 3: DAD1 C, Sig=214,4 Ref=360,100

Peak #	RetTime [min]	Type	Width [min]	Area [mAU*s]	Height [mAU]	Area %
1	10.365	BB	0.4162	6727.83057	245.13141	49.9614
2	17.878	BB	0.7747	6738.22803	131.30544	50.0386
Totals :				1.34661e4	376.43684	

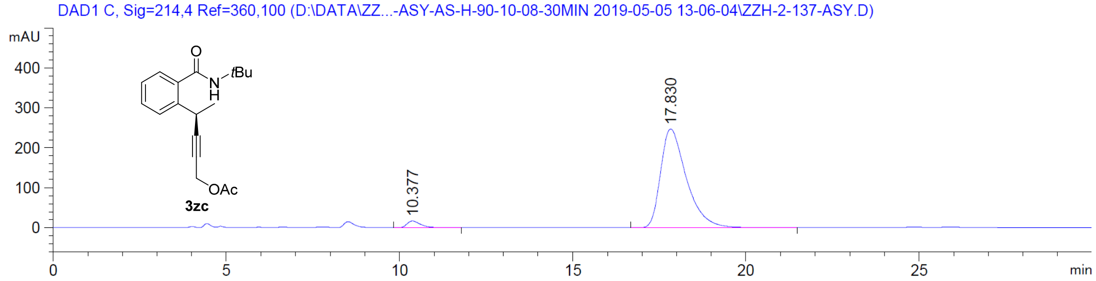

Signal 3: DAD1 C, Sig=214,4 Ref=360,100

Peak #	RetTime [min]	Type	Width [min]	Area [mAU*s]	Height [mAU]	Area %
1	10.377	BB	0.4135	455.76947	16.74649	3.4313
2	17.830	BB	0.7849	1.28268e4	247.39139	96.5687
Totals :				1.32826e4	264.13787	

Supplementary Figure 211 HPLC spectra for racemic and chiral **3zc**. **HPLC** analysis: Chiralcel AD-H (*n*-hexane/*i*-PrOH = 90/10, flow rate 0.8 mL/min, λ = 214 nm), t_R (minor) = 10.38 min, t_R (major) = 17.83 min, 93% ee.

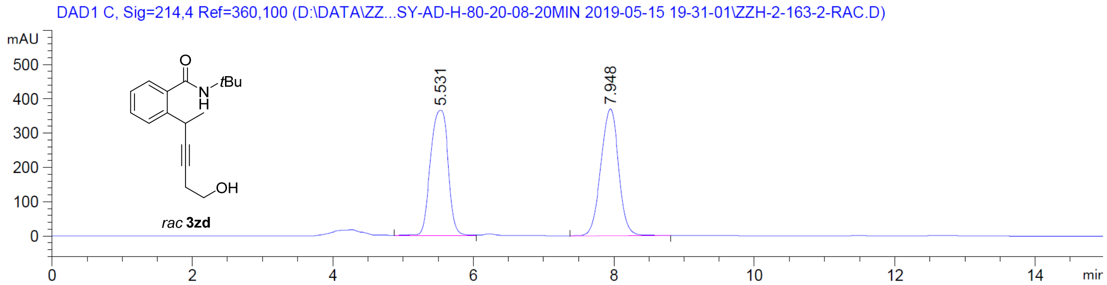

Signal 3: DAD1 C, Sig=214,4 Ref=360,100

Peak #	RetTime [min]	Type	Width [min]	Area [mAU*s]	Height [mAU]	Area %
1	5.531	BV	0.2845	6340.88037	365.30557	49.4466
2	7.948	BB	0.2746	6482.80859	369.68283	50.5534

Totals : 1.28237e4 734.98840

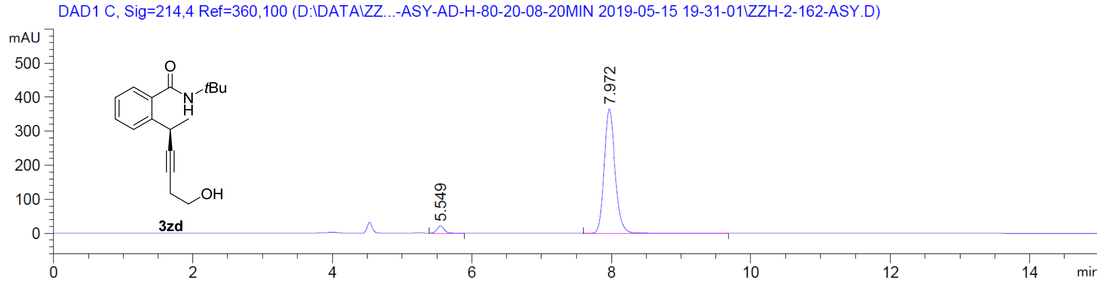

Signal 3: DAD1 C, Sig=214,4 Ref=360,100

Peak #	RetTime [min]	Type	Width [min]	Area [mAU*s]	Height [mAU]	Area %
1	5.549	VB	0.1085	148.62312	21.02804	3.6233
2	7.972	BB	0.1663	3953.20776	365.30444	96.3767

Totals : 4101.83089 386.33248

Supplementary Figure 212 HPLC spectra for racemic and chiral **3zd**. **HPLC** analysis: Chiralcel AD-H (*n*-hexane/*i*-PrOH = 80/20, flow rate 0.8 mL/min, λ = 214 nm), t_R (minor) = 5.55 min, t_R (major) = 7.97 min, 93% ee.

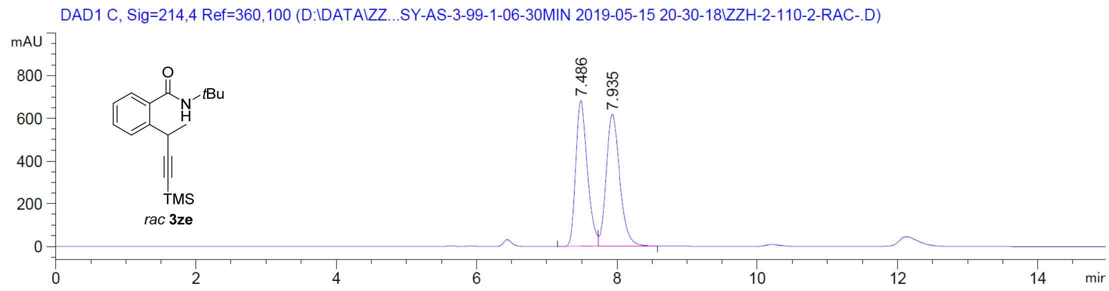

Signal 3: DAD1 C, Sig=214,4 Ref=360,100

Peak #	RetTime [min]	Type	Width [min]	Area [mAU*s]	Height [mAU]	Area %
1	7.486	BV	0.1795	7926.44043	682.64960	49.0323
2	7.935	VB	0.2061	8239.32813	615.90424	50.9677

Totals : 1.61658e4 1298.55383

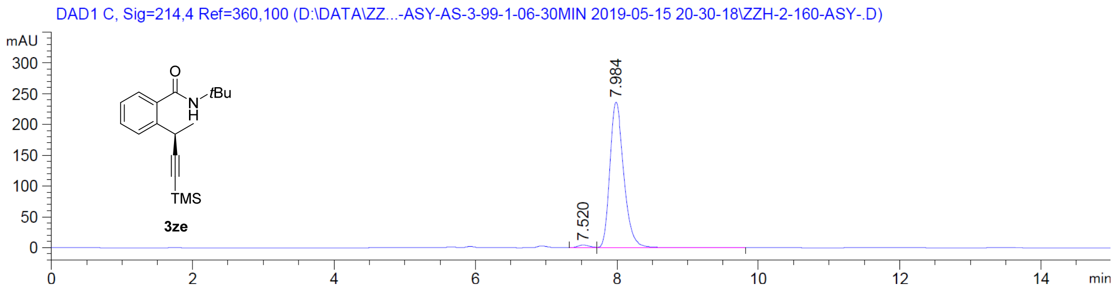

Signal 3: DAD1 C, Sig=214,4 Ref=360,100

Peak #	RetTime [min]	Type	Width [min]	Area [mAU*s]	Height [mAU]	Area %
1	7.520	VV	0.1778	50.25810	4.38182	1.5313
2	7.984	VB	0.2092	3231.69824	236.82805	98.4687

Totals : 3281.95634 241.20987

Supplementary Figure 213 HPLC spectra for racemic and chiral **3ze**. **HPLC** analysis: Chiralcel AS3 (*n*-hexane/*i*-PrOH = 99/1, flow rate 0.6 mL/min, λ = 214 nm), t_R (minor) = 7.52 min, t_R (major) = 7.98 min, 97% ee.

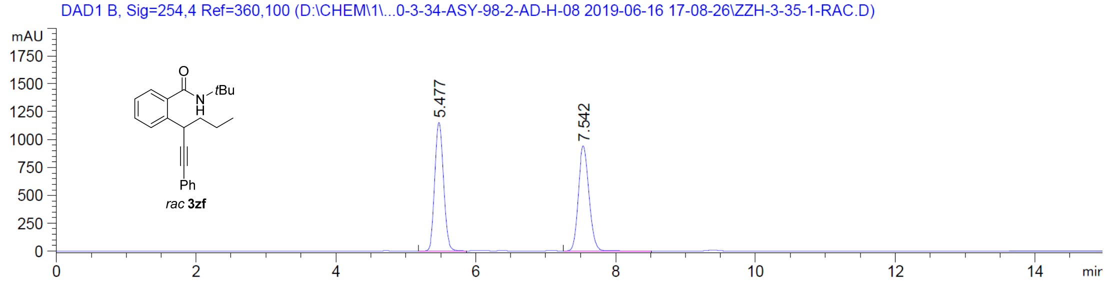

Signal 2: DAD1 B, Sig=254,4 Ref=360,100

Peak #	RetTime [min]	Type	Width [min]	Area [mAU*s]	Height [mAU]	Area %
1	5.477	BV	0.1371	9987.53027	1153.00891	49.8651
2	7.542	VV	0.1682	1.00416e4	943.48816	50.1349

Totals : 2.00291e4 2096.49707

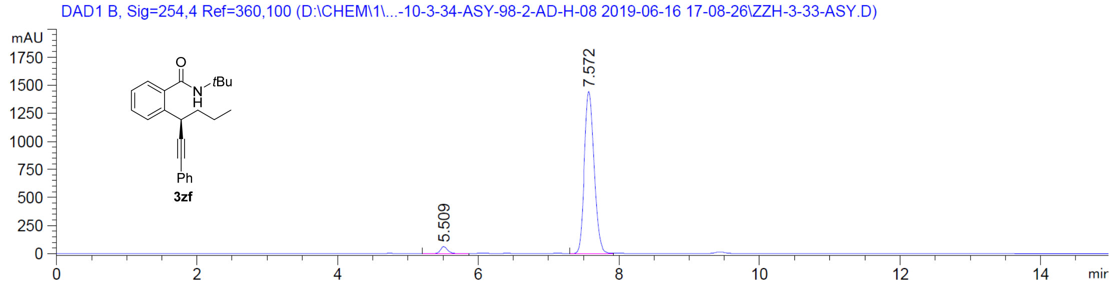

Signal 2: DAD1 B, Sig=254,4 Ref=360,100

Peak #	RetTime [min]	Type	Width [min]	Area [mAU*s]	Height [mAU]	Area %
1	5.509	BB	0.1156	443.27203	61.83281	3.0955
2	7.572	BV	0.1522	1.38767e4	1442.86646	96.9045

Totals : 1.43200e4 1504.69927

Supplementary Figure 214 HPLC spectra for racemic and chiral **3zf**. **HPLC** analysis: Chiralcel AD-H (*n*-hexane/*i*-PrOH = 90/10, flow rate 0.8 mL/min, λ = 254 nm), t_R (minor) = 5.51 min, t_R (major) = 7.57 min, 94% ee.

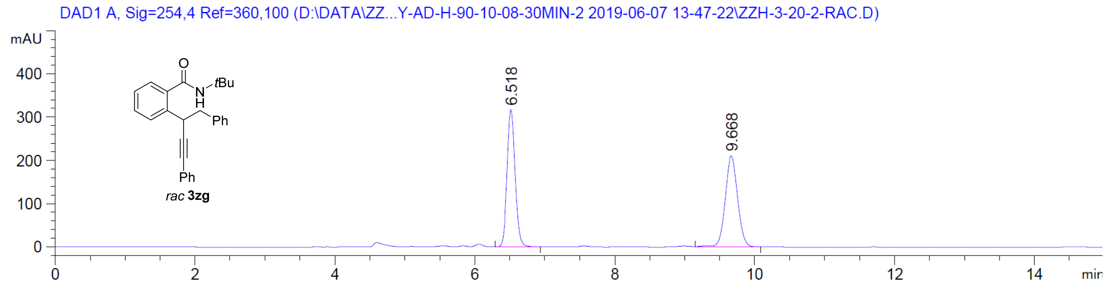

Signal 1: DAD1 A, Sig=254,4 Ref=360,100

Peak #	RetTime [min]	Type	Width [min]	Area [mAU*s]	Height [mAU]	Area %
1	6.518	BB	0.1226	2513.44531	316.70026	49.2219
2	9.668	VB	0.1896	2592.91064	210.65535	50.7781

Totals : 5106.35596 527.35561

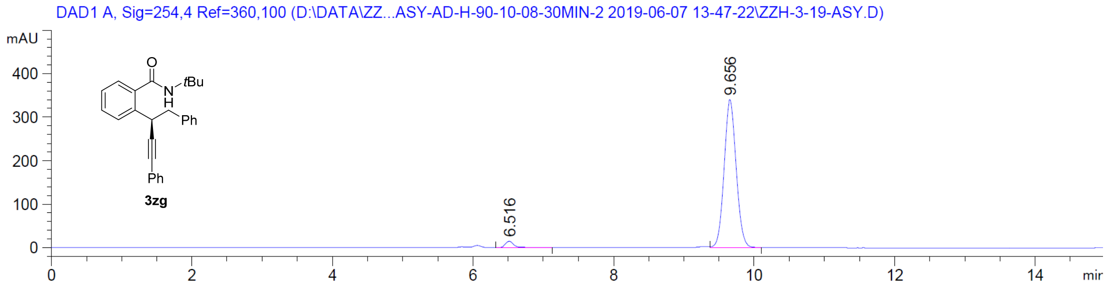

Signal 1: DAD1 A, Sig=254,4 Ref=360,100

Peak #	RetTime [min]	Type	Width [min]	Area [mAU*s]	Height [mAU]	Area %
1	6.516	BB	0.1278	127.23595	14.87852	3.0367
2	9.656	VB	0.1874	4062.74463	339.96881	96.9633

Totals : 4189.98058 354.84733

Supplementary Figure 215 HPLC spectra for racemic and chiral **3zg**. **HPLC** analysis: Chiralcel AD-H (*n*-hexane/*i*-PrOH = 90/10, flow rate 0.8 mL/min, λ = 254 nm), t_R (minor) = 6.52 min, t_R (major) = 9.66 min, 94% ee.

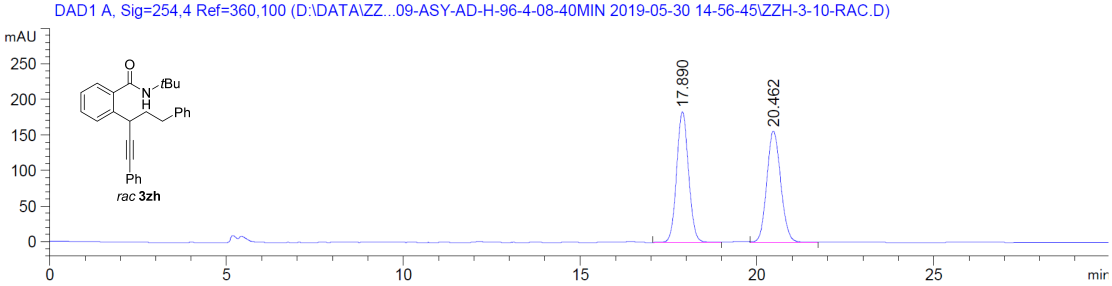

Signal 1: DAD1 A, Sig=254,4 Ref=360,100

Peak #	RetTime [min]	Type	Width [min]	Area [mAU*s]	Height [mAU]	Area %
1	17.890	BB	0.3627	4282.02637	183.18259	50.1923
2	20.462	BB	0.4236	4249.21143	156.07678	49.8077

Totals : 8531.23779 339.25937

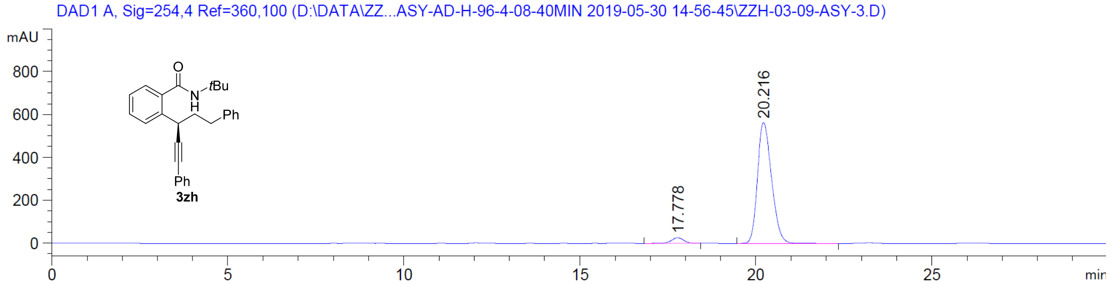

Signal 1: DAD1 A, Sig=254,4 Ref=360,100

Peak #	RetTime [min]	Type	Width [min]	Area [mAU*s]	Height [mAU]	Area %
1	17.778	BB	0.3764	649.96436	26.47435	3.9931
2	20.216	BB	0.4315	1.56274e4	563.59027	96.0069

Totals : 1.62774e4 590.06462

Supplementary Figure 216 HPLC spectra for racemic and chiral **3zh**. **HPLC** analysis: Chiralcel AD-H (*n*-hexane/*i*-PrOH = 96/4, flow rate 0.8 mL/min, λ = 254 nm), t_R (minor) = 17.78 min, t_R (major) = 20.22 min, 92% ee.

DAD1 A, Sig=254,4 Ref=360,100 (D:\DATA\ZZ...165-ASY-90-10-08-30MIN 2019-05-23 20-46-23\ZZH-2-177-1-RAC.D)

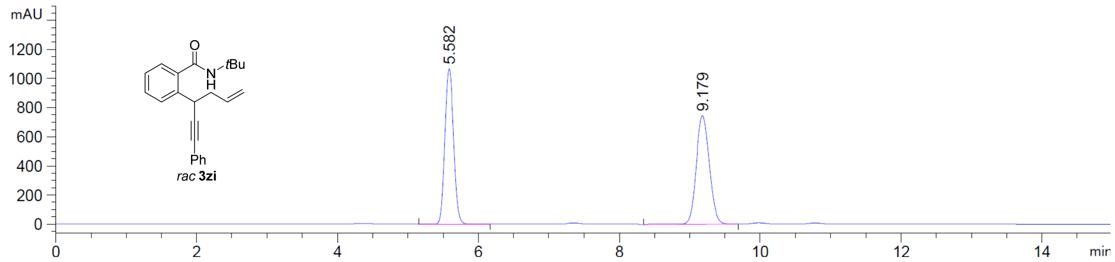

Signal 1: DAD1 A, Sig=254,4 Ref=360,100

Peak #	RetTime [min]	Type	Width [min]	Area [mAU*s]	Height [mAU]	Area %
1	5.582	BB	0.1371	9250.52734	1067.25024	49.1251
2	9.179	BB	0.2020	9580.03418	745.23218	50.8749

Totals : 1.88306e4 1812.48242

DAD1 A, Sig=254,4 Ref=360,100 (D:\DATA\ZZ...2-165-ASY-90-10-08-30MIN 2019-05-23 20-46-23\ZZH-2-165-ASY.D)

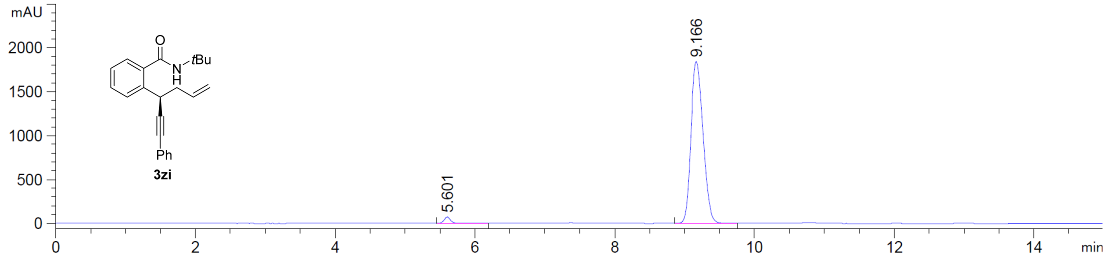

Signal 1: DAD1 A, Sig=254,4 Ref=360,100

Peak #	RetTime [min]	Type	Width [min]	Area [mAU*s]	Height [mAU]	Area %
1	5.601	VB	0.1007	476.08694	72.37279	2.1031
2	9.166	VV	0.1904	2.21615e4	1842.17822	97.8969

Totals : 2.26376e4 1914.55101

Supplementary Figure 217 HPLC spectra for racemic and chiral **3zi**. **HPLC** analysis: Chiralcel AD-H (*n*-hexane/*i*-PrOH = 90/10, flow rate 0.8 mL/min, λ = 254 nm), t_R (minor) = 5.60 min, t_R (major) = 9.17 min, 96% ee.

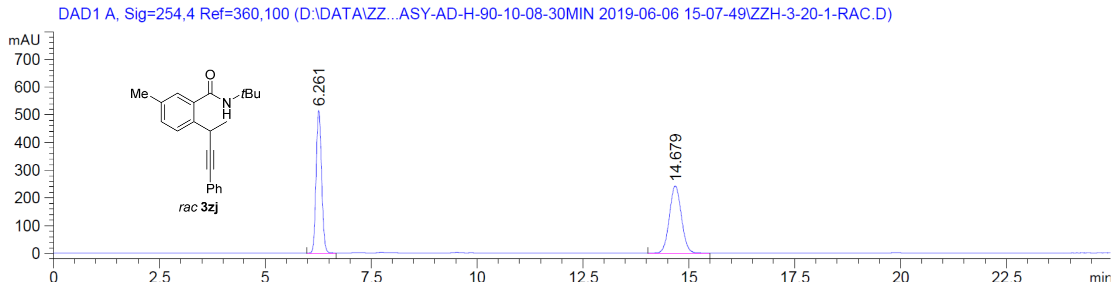

Signal 1: DAD1 A, Sig=254,4 Ref=360,100

Peak #	RetTime [min]	Type	Width [min]	Area [mAU*s]	Height [mAU]	Area %
1	6.261	BB	0.1412	4640.98584	514.88904	49.4506
2	14.679	BB	0.3066	4744.10986	242.11598	50.5494

Totals : 9385.09570 757.00502

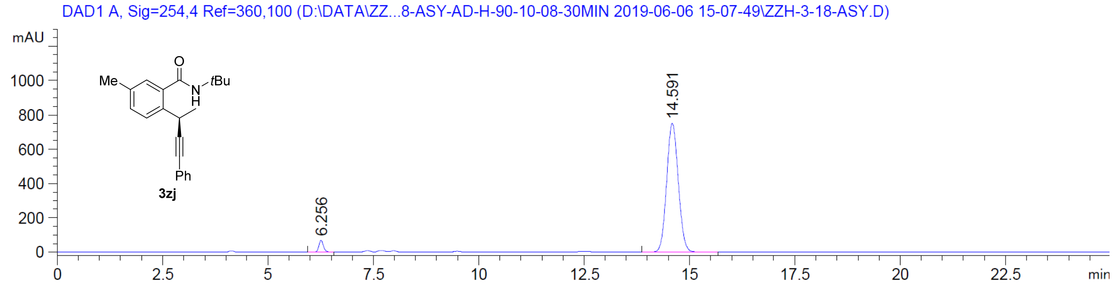

Signal 1: DAD1 A, Sig=254,4 Ref=360,100

Peak #	RetTime [min]	Type	Width [min]	Area [mAU*s]	Height [mAU]	Area %
1	6.256	BB	0.1148	517.28705	69.49290	3.4689
2	14.591	BB	0.3019	1.43950e4	750.17084	96.5311

Totals : 1.49123e4 819.66373

Supplementary Figure 218 HPLC spectra for racemic and chiral **3zj**. **HPLC** analysis: Chiralcel AD-H (*n*-hexane/*i*-PrOH = 90/10, flow rate 0.8 mL/min, λ = 254 nm), t_R (minor) = 6.26 min, t_R (major) = 14.59 min, 93% ee.

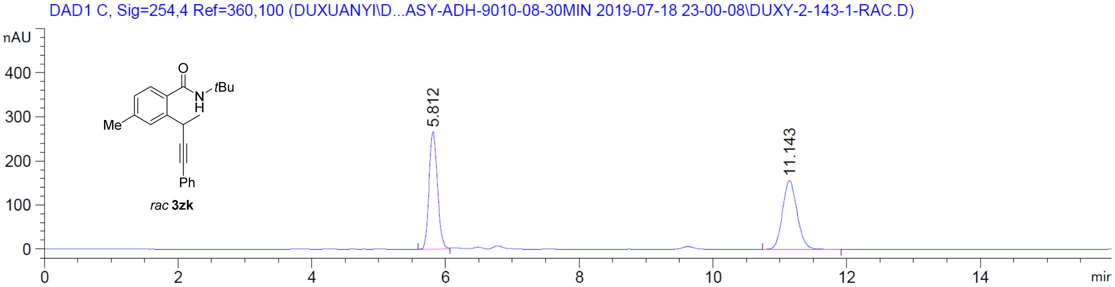

Signal 3: DAD1 C, Sig=254,4 Ref=360,100

Peak #	RetTime [min]	Type	Width [min]	Area [mAU*s]	Height [mAU]	Area %
1	5.812	BV	0.1377	2321.93970	266.27017	49.8250
2	11.143	BB	0.2334	2338.25000	155.50862	50.1750
Totals :				4660.18970	421.77879	

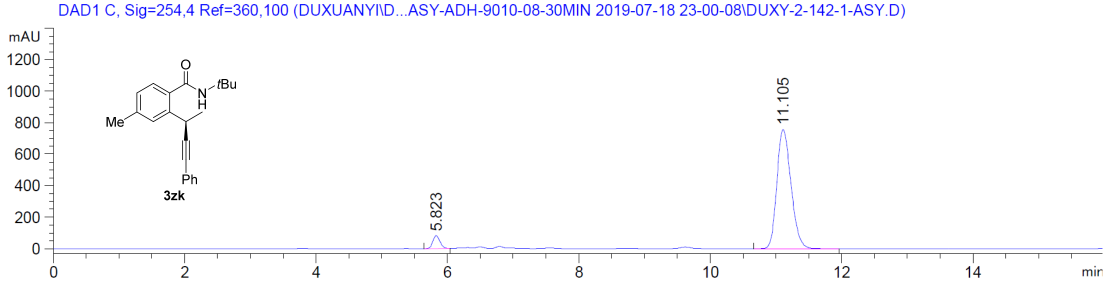

Signal 3: DAD1 C, Sig=254,4 Ref=360,100

Peak #	RetTime [min]	Type	Width [min]	Area [mAU*s]	Height [mAU]	Area %
1	5.823	BV	0.1184	624.56110	82.46076	5.2342
2	11.105	BB	0.2321	1.13078e4	757.89447	94.7658
Totals :				1.19323e4	840.35523	

Supplementary Figure 219 HPLC spectra for racemic and chiral **3zk**. **HPLC** analysis: Chiralcel AD-H (*n*-hexane/*i*-PrOH = 90/10, flow rate 0.8 mL/min, λ = 254 nm), t_R (minor) = 5.82 min, t_R (major) = 11.10 min, 90% ee.

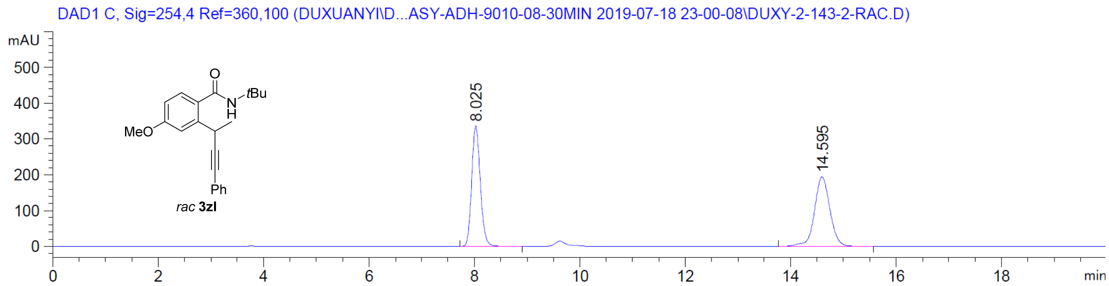

Signal 3: DAD1 C, Sig=254,4 Ref=360,100

Peak #	RetTime [min]	Type	Width [min]	Area [mAU*s]	Height [mAU]	Area %
1	8.025	BB	0.1768	3821.20508	335.73892	49.3065
2	14.595	BB	0.3127	3928.69360	193.64345	50.6935

Totals : 7749.89868 529.38237

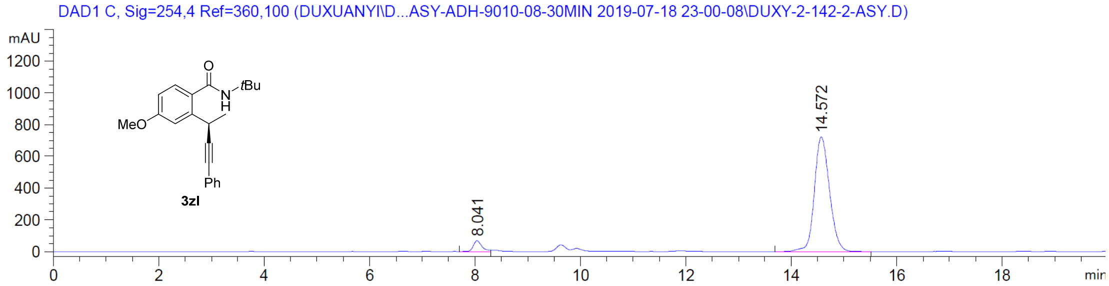

Signal 3: DAD1 C, Sig=254,4 Ref=360,100

Peak #	RetTime [min]	Type	Width [min]	Area [mAU*s]	Height [mAU]	Area %
1	8.041	BV	0.1667	770.25372	68.74343	5.0804
2	14.572	BB	0.3082	1.43911e4	723.08429	94.9196

Totals : 1.51614e4 791.82772

Supplementary Figure 220 HPLC spectra for racemic and chiral **3zl**. **HPLC** analysis: Chiralcel AD-H (*n*-hexane/*i*-PrOH = 90/10, flow rate 0.8 mL/min, λ = 254 nm), t_R (minor) = 8.04 min, t_R (major) = 14.57 min, 90% ee.

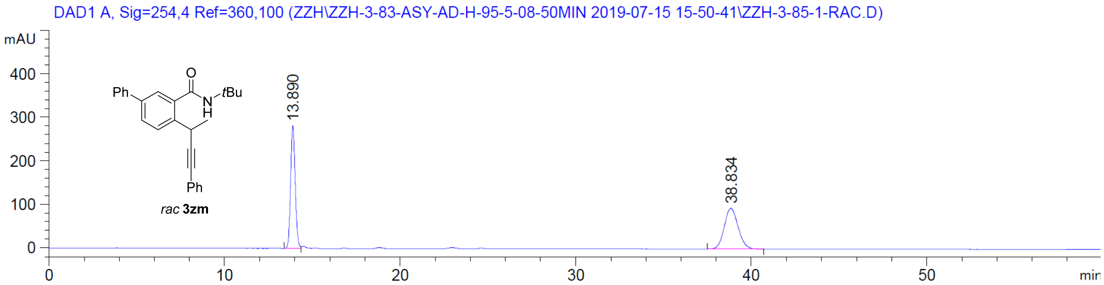

Signal 1: DAD1 A, Sig=254,4 Ref=360,100

Peak #	RetTime [min]	Type	Width [min]	Area [mAU*s]	Height [mAU]	Area %
1	13.890	BV	0.2793	5093.66309	281.22104	50.0560
2	38.834	BB	0.8311	5082.27295	93.36119	49.9440

Totals : 1.01759e4 374.58223

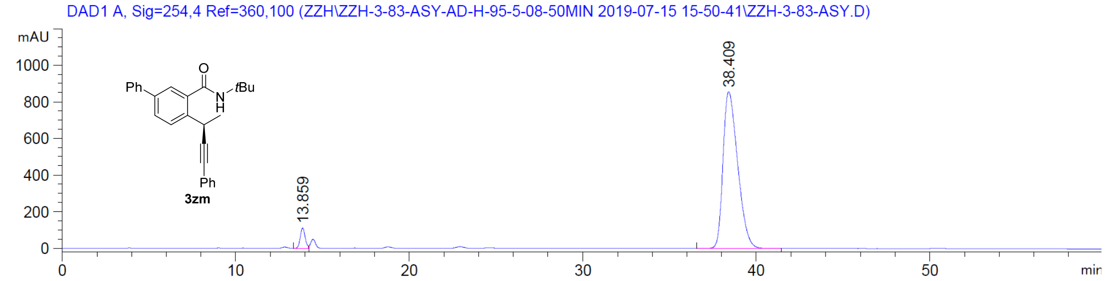

Signal 1: DAD1 A, Sig=254,4 Ref=360,100

Peak #	RetTime [min]	Type	Width [min]	Area [mAU*s]	Height [mAU]	Area %
1	13.859	BV	0.3070	2177.76416	110.93793	4.1554
2	38.409	BB	0.8829	5.02298e4	855.58966	95.8446

Totals : 5.24076e4 966.52759

Supplementary Figure 221 HPLC spectra for racemic and chiral **3zm**. **HPLC** analysis: Chiralcel AD-H (*n*-hexane/*i*-PrOH = 95/5, flow rate 0.8 mL/min, λ = 254 nm), t_R (minor) = 13.86 min, t_R (major) = 38.41 min, 92% ee.

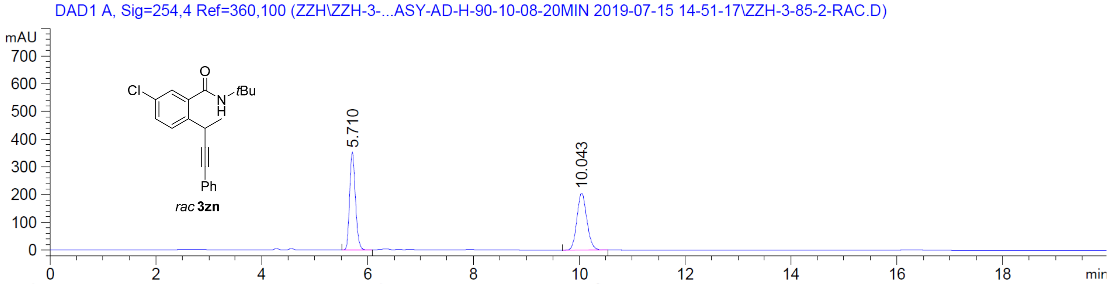

Signal 1: DAD1 A, Sig=254,4 Ref=360,100

Peak #	RetTime [min]	Type	Width [min]	Area [mAU*s]	Height [mAU]	Area %
1	5.710	BB	0.1163	2674.99731	353.58524	49.7163
2	10.043	BB	0.2053	2705.52637	205.85645	50.2837

Totals : 5380.52368 559.44168

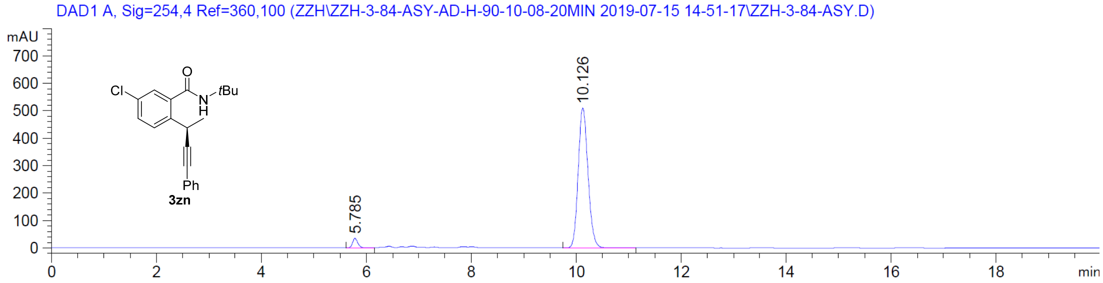

Signal 1: DAD1 A, Sig=254,4 Ref=360,100

Peak #	RetTime [min]	Type	Width [min]	Area [mAU*s]	Height [mAU]	Area %
1	5.785	BB	0.1074	240.85872	34.54226	3.5047
2	10.126	BB	0.2016	6631.53076	510.18918	96.4953

Totals : 6872.38948 544.73144

Supplementary Figure 222 HPLC spectra for racemic and chiral **3zn**. **HPLC** analysis: Chiralcel AD-H (*n*-hexane/*i*-PrOH = 90/10, flow rate 0.8 mL/min, λ = 254 nm), t_R (minor) = 5.79 min, t_R (major) = 10.13 min, 93% ee.

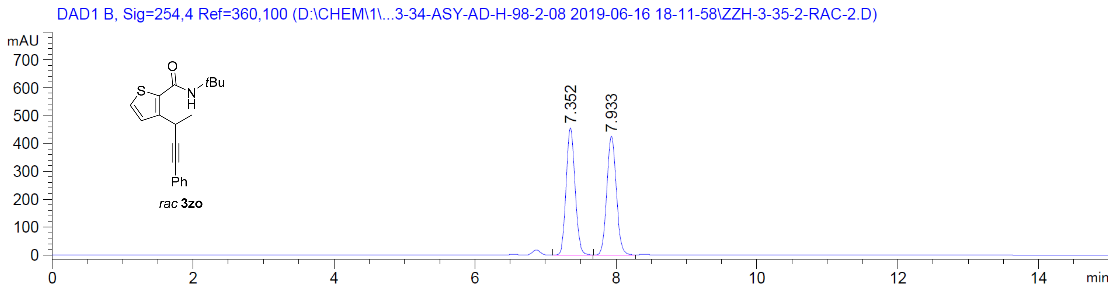

Signal 2: DAD1 B, Sig=254,4 Ref=360,100

Peak #	RetTime [min]	Type	Width [min]	Area [mAU*s]	Height [mAU]	Area %
1	7.352	BB	0.1370	3942.03809	455.34286	49.9680
2	7.933	BV	0.1482	3947.08984	425.52725	50.0320

Totals : 7889.12793 880.87012

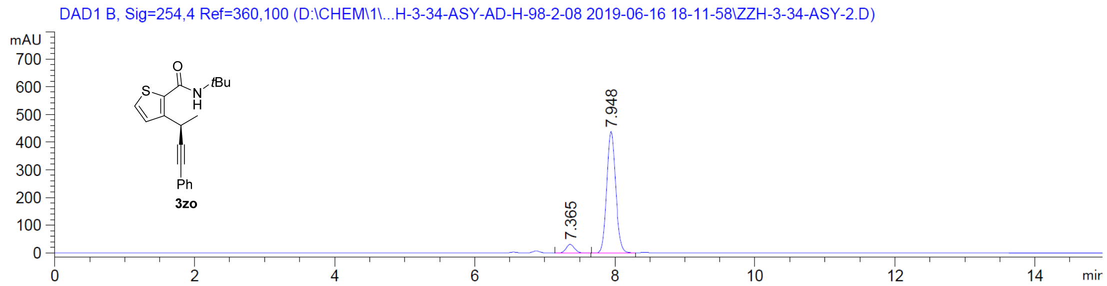

Signal 2: DAD1 B, Sig=254,4 Ref=360,100

Peak #	RetTime [min]	Type	Width [min]	Area [mAU*s]	Height [mAU]	Area %
1	7.365	BB	0.1314	257.32443	31.45772	6.2037
2	7.948	BV	0.1397	3890.62354	437.50522	93.7963

Totals : 4147.94797 468.96293

Supplementary Figure 223 HPLC spectra for racemic and chiral **3zo**. **HPLC** analysis: Chiralcel AD-H (*n*-hexane/*i*-PrOH = 98/2, flow rate 0.8 mL/min, λ = 254 nm), t_R (minor) = 7.37 min, t_R (major) = 7.95 min, 88% ee.

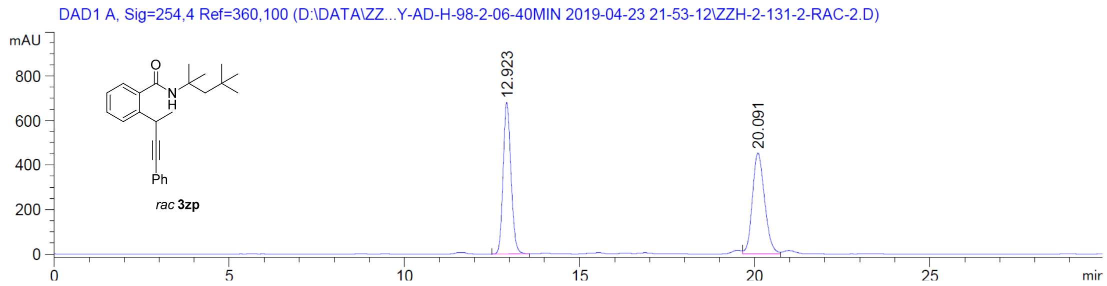

Signal 1: DAD1 A, Sig=254,4 Ref=360,100

Peak #	RetTime [min]	Type	Width [min]	Area [mAU*s]	Height [mAU]	Area %
1	12.923	BB	0.2412	1.05707e4	680.53595	48.9935
2	20.091	VV	0.3769	1.10050e4	453.78110	51.0065
Totals :				2.15757e4	1134.31705	

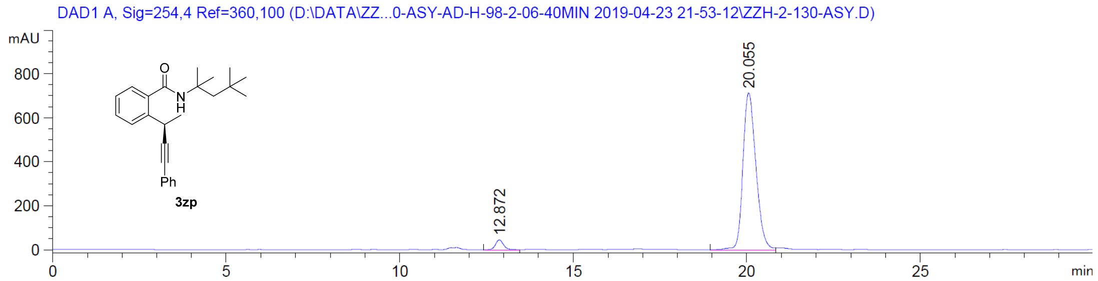

Signal 1: DAD1 A, Sig=254,4 Ref=360,100

Peak #	RetTime [min]	Type	Width [min]	Area [mAU*s]	Height [mAU]	Area %
1	12.872	BB	0.2372	684.79474	44.57124	3.5769
2	20.055	BV	0.4019	1.84599e4	713.32214	96.4231
Totals :				1.91447e4	757.89339	

Supplementary Figure 224 HPLC spectra for racemic and chiral **3zp**. **HPLC** analysis: Chiralcel AD-H (*n*-hexane/*i*-PrOH = 98/2, flow rate 0.8 mL/min, λ = 254 nm), t_R (minor) = 12.87 min, t_R (major) = 20.06 min, 93% ee.

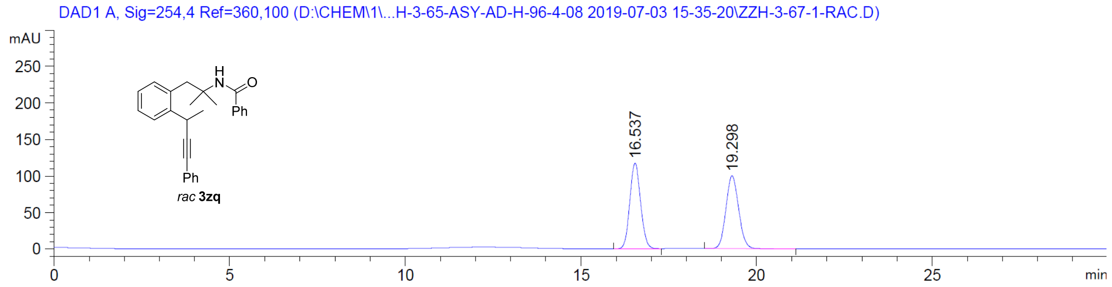

Signal 1: DAD1 A, Sig=254,4 Ref=360,100

Peak #	RetTime [min]	Type	Width [min]	Area [mAU*s]	Height [mAU]	Area %
1	16.537	BB	0.3304	2499.99121	117.39439	49.8355
2	19.298	BB	0.3892	2516.49951	100.14473	50.1645
Totals :				5016.49072	217.53912	

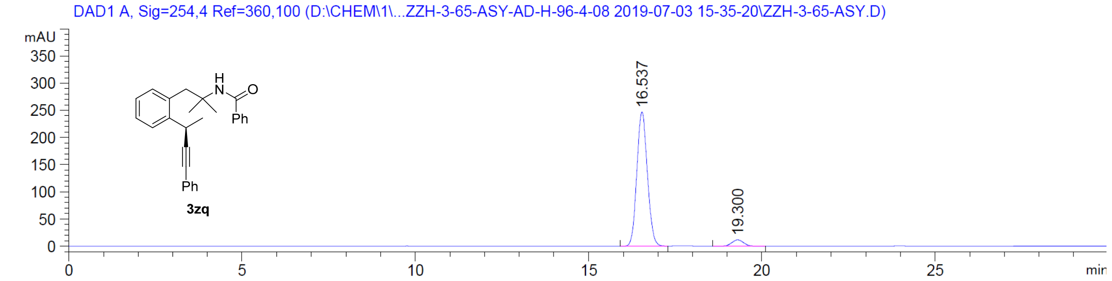

Signal 1: DAD1 A, Sig=254,4 Ref=360,100

Peak #	RetTime [min]	Type	Width [min]	Area [mAU*s]	Height [mAU]	Area %
1	16.537	BB	0.3290	5227.24170	246.87074	94.6319
2	19.300	BB	0.3789	296.52252	12.14314	5.3681
Totals :				5523.76422	259.01388	

Supplementary Figure 225 HPLC spectra for racemic and chiral **3zq**. **HPLC** analysis: Chiralcel AD-H (*n*-hexane/*i*-PrOH = 96/4, flow rate 0.8 mL/min, λ = 254 nm), t_R (major) = 16.54 min, t_R (minor) = 19.30 min, 89% ee.

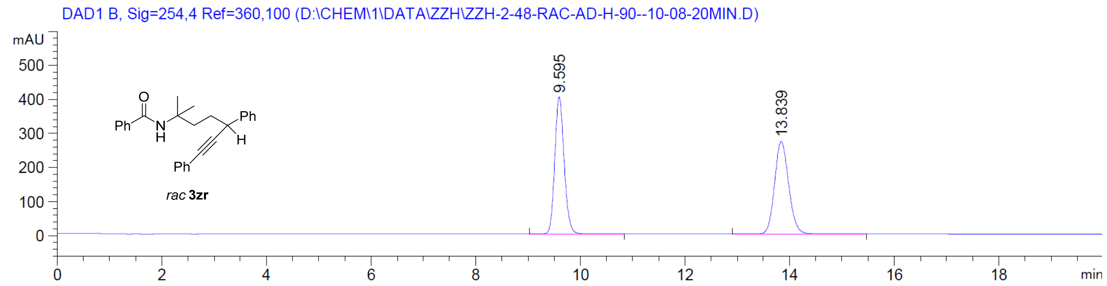

Signal 2: DAD1 B, Sig=254,4 Ref=360,100

Peak #	RetTime [min]	Type	Width [min]	Area [mAU*s]	Height [mAU]	Area %
1	9.595	VV	0.1980	5029.87793	401.99277	49.9012
2	13.839	BV	0.2893	5049.78662	271.11566	50.0988

Totals : 1.00797e4 673.10843

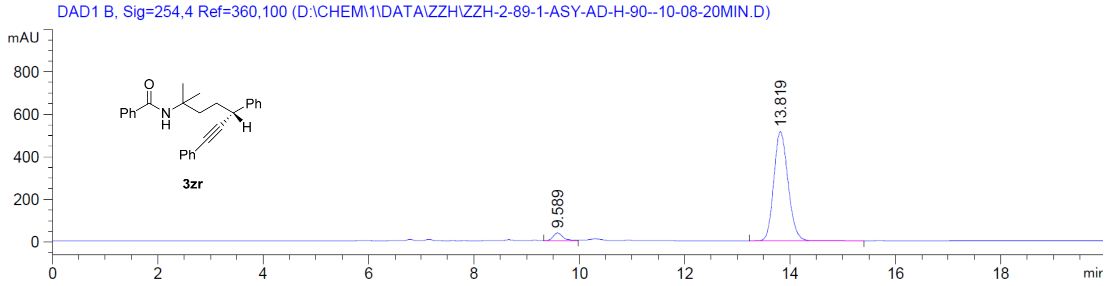

Signal 2: DAD1 B, Sig=254,4 Ref=360,100

Peak #	RetTime [min]	Type	Width [min]	Area [mAU*s]	Height [mAU]	Area %
1	9.589	BV	0.2020	474.17163	35.91711	4.6895
2	13.819	BV	0.2955	9637.12305	512.21460	95.3105

Totals : 1.01113e4 548.13171

Supplementary Figure 226 HPLC spectra for racemic and chiral **3zr**. **HPLC** analysis: Chiralcel AD-H (*n*-hexane/*i*-PrOH = 90/10, flow rate 0.8 mL/min, λ = 254 nm), t_R (minor) = 9.59 min, t_R (major) = 13.82 min, 91% ee.

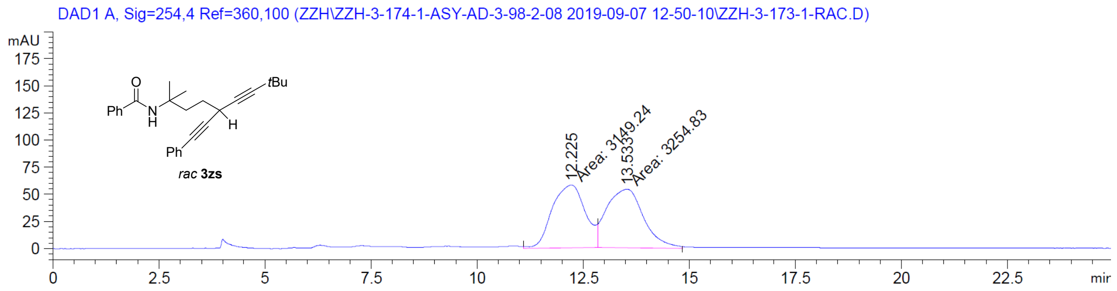

Signal 1: DAD1 A, Sig=254,4 Ref=360,100

Peak #	RetTime [min]	Type	Width [min]	Area [mAU*s]	Height [mAU]	Area %
1	12.225	MM	0.9071	3149.24463	57.86389	49.1756
2	13.533	MM	1.0061	3254.83252	53.91599	50.8244

Totals : 6404.07715 111.77988

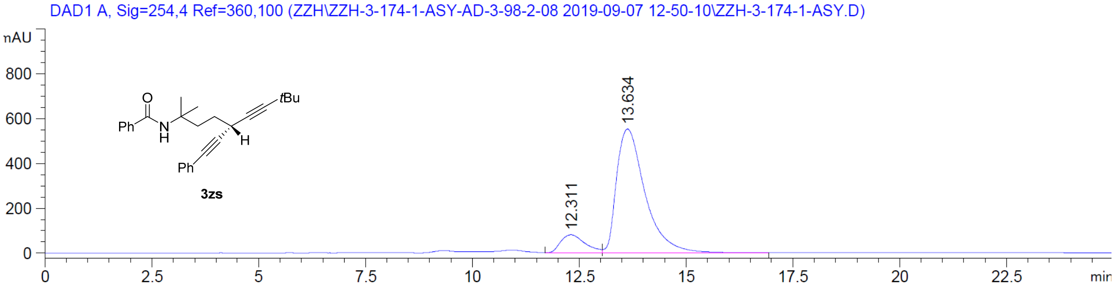

Signal 1: DAD1 A, Sig=254,4 Ref=360,100

Peak #	RetTime [min]	Type	Width [min]	Area [mAU*s]	Height [mAU]	Area %
1	12.311	BV	0.6122	3121.80127	79.49506	10.9177
2	13.634	VB	0.7138	2.54722e4	550.57019	89.0823

Totals : 2.85940e4 630.06525

Supplementary Figure 227 HPLC spectra for racemic and chiral **3zs**. **HPLC** analysis: Chiralcel AD3 (*n*-hexane/*i*-PrOH = 98/2, flow rate 0.8 mL/min, λ = 254 nm), t_R (minor) = 13.31 min, t_R (major) = 13.63 min, 78% ee.

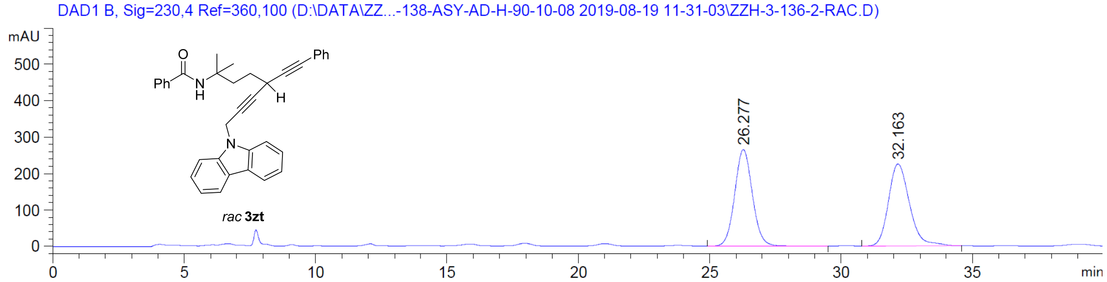

Signal 2: DAD1 B, Sig=230,4 Ref=360,100

Peak #	RetTime [min]	Type	Width [min]	Area [mAU*s]	Height [mAU]	Area %
1	26.277	BB	0.7259	1.24698e4	265.50247	49.8599
2	32.163	BB	0.8607	1.25399e4	225.50816	50.1401

Totals : 2.50096e4 491.01064

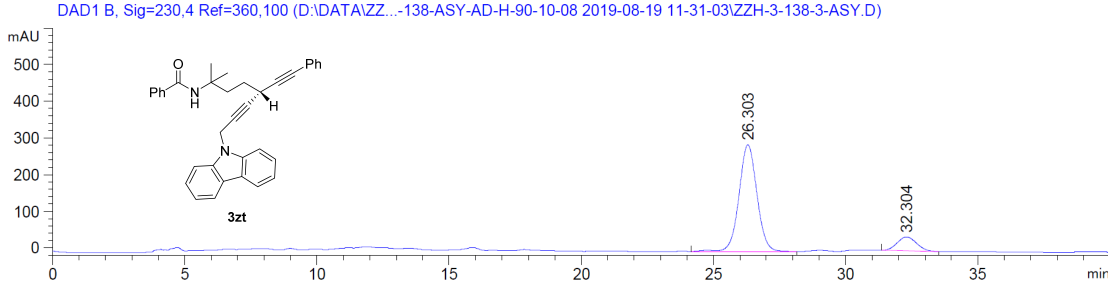

Signal 2: DAD1 B, Sig=230,4 Ref=360,100

Peak #	RetTime [min]	Type	Width [min]	Area [mAU*s]	Height [mAU]	Area %
1	26.303	BB	0.7277	1.37594e4	290.98029	87.6642
2	32.304	BB	0.8036	1936.18591	38.05697	12.3358

Totals : 1.56956e4 329.03725

Supplementary Figure 228 HPLC spectra for racemic and chiral **3zt**. **HPLC** analysis: Chiralcel AD-H (*n*-hexane/*i*-PrOH = 90/10, flow rate 0.8 mL/min, λ = 230 nm), t_R (major) = 26.30 min, t_R (minor) = 32.30 min, 75% ee.

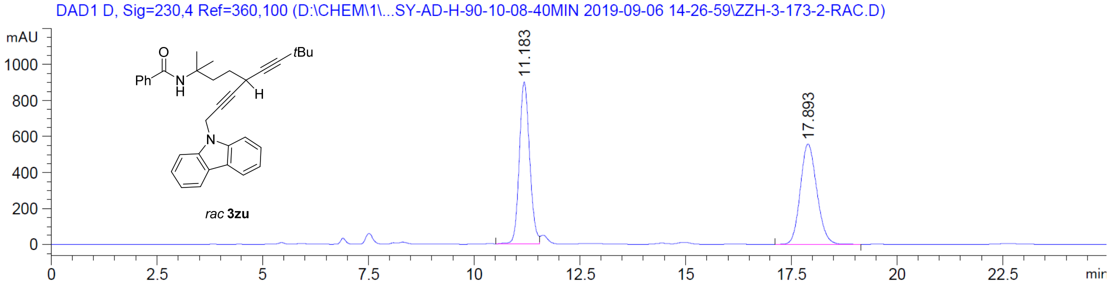

Signal 4: DAD1 D, Sig=230,4 Ref=360,100

Peak #	RetTime [min]	Type	Width [min]	Area [mAU*s]	Height [mAU]	Area %
1	11.183	VV	0.2626	1.53298e4	900.45593	50.3546
2	17.893	BB	0.4205	1.51138e4	557.18671	49.6454

Totals :						
Totals :				3.04436e4	1457.64264	

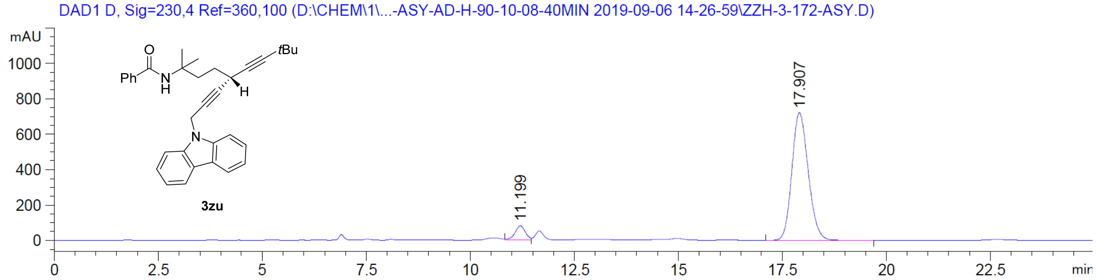

Signal 4: DAD1 D, Sig=230,4 Ref=360,100

Peak #	RetTime [min]	Type	Width [min]	Area [mAU*s]	Height [mAU]	Area %
1	11.199	VV	0.2689	1408.55432	80.19509	6.6620
2	17.907	BB	0.4256	1.97344e4	720.48608	93.3380

Totals :				2.11430e4	800.68118	

Supplementary Figure 229 HPLC spectra for racemic and chiral **3zu**. **HPLC** analysis: Chiralcel AD-H (*n*-hexane/*i*-PrOH = 90/10, flow rate 0.8 mL/min, λ = 230 nm), t_R (minor) = 11.20 min, t_R (major) = 17.91 min, 87% ee.

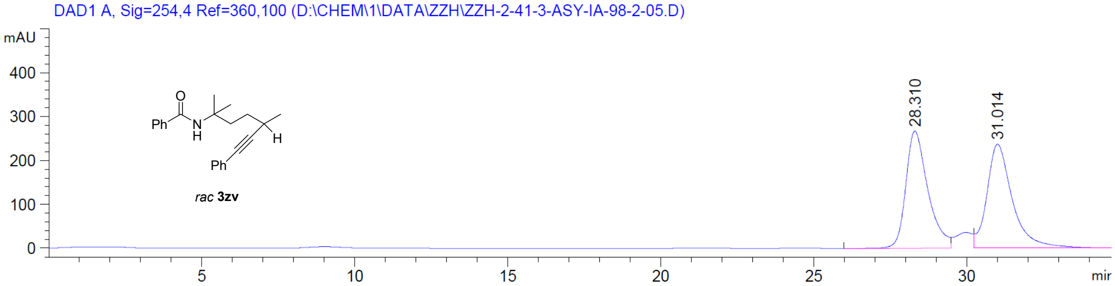

Signal 1: DAD1 A, Sig=254,4 Ref=360,100

Peak #	RetTime [min]	Type	Width [min]	Area [mAU*s]	Height [mAU]	Area %
1	28.310	BV	0.7529	1.34369e4	267.11981	49.3258
2	31.014	VBA	0.8636	1.38042e4	236.98091	50.6742
Totals :				2.72412e4	504.10072	

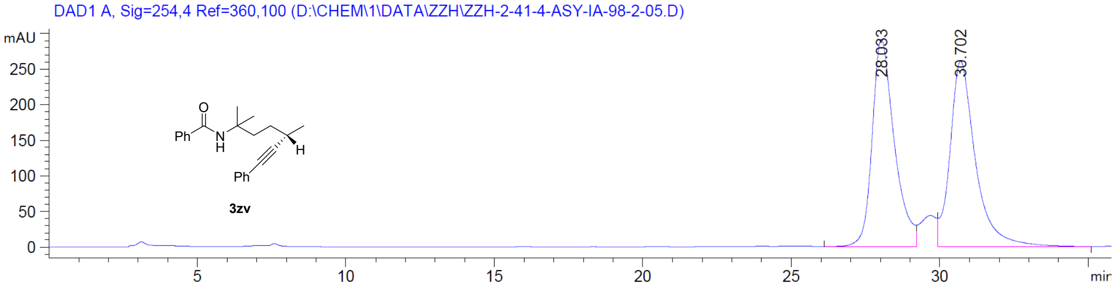

Signal 1: DAD1 A, Sig=254,4 Ref=360,100

Peak #	RetTime [min]	Type	Width [min]	Area [mAU*s]	Height [mAU]	Area %
1	28.033	BV	0.7756	1.51256e4	291.38507	49.3574
2	30.702	VB	0.8790	1.55195e4	260.51135	50.6426
Totals :				3.06452e4	551.89642	

Supplementary Figure 230 HPLC spectra for racemic and chiral **3zv**. **HPLC** analysis: Chiralcel IA (*n*-hexane/*i*-PrOH = 98/2, flow rate 0.8 mL/min, λ = 254 nm), t_R (minor) = 28.03 min, t_R (major) = 30.70 min, 0% ee.

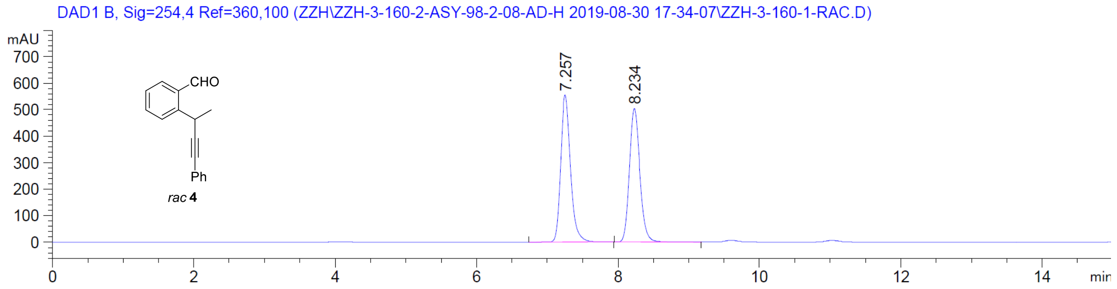

Signal 2: DAD1 B, Sig=254,4 Ref=360,100

Peak #	RetTime [min]	Type	Width [min]	Area [mAU*s]	Height [mAU]	Area %
1	7.257	BB	0.1530	5367.48486	554.18671	50.9526
2	8.234	BV	0.1638	5166.77881	503.35782	49.0474

Totals : 1.05343e4 1057.54453

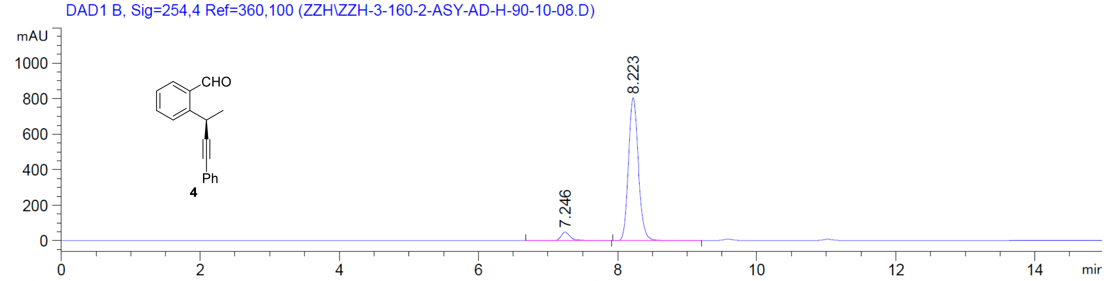

Signal 2: DAD1 B, Sig=254,4 Ref=360,100

Peak #	RetTime [min]	Type	Width [min]	Area [mAU*s]	Height [mAU]	Area %
1	7.246	BB	0.1478	480.85794	50.19764	5.6807
2	8.223	BV	0.1519	7983.93896	803.61041	94.3193

Totals : 8464.79691 853.80806

Supplementary Figure 231 HPLC spectra for racemic and chiral **4**. **HPLC** analysis: Chiralcel AD-H (*n*-hexane/*i*-PrOH = 90/10, flow rate 0.8 mL/min, λ = 254 nm), t_R (minor) = 7.25 min, t_R (major) = 8.22 min, 89% ee.

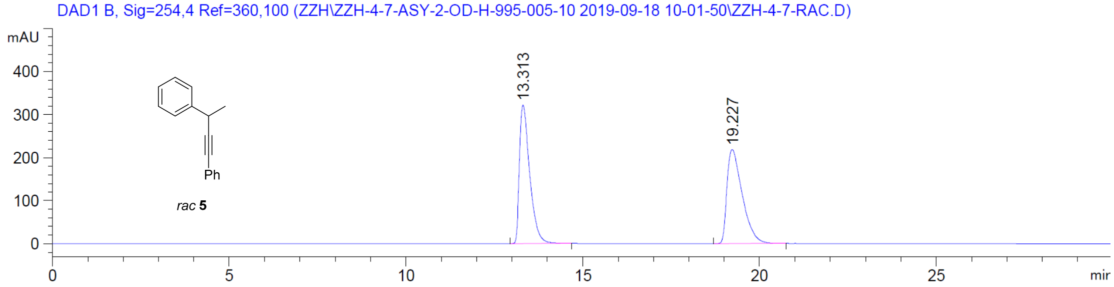

Signal 2: DAD1 B, Sig=254,4 Ref=360,100

Peak #	RetTime [min]	Type	Width [min]	Area [mAU*s]	Height [mAU]	Area %
1	13.313	BB	0.3076	6513.43213	322.48468	49.7764
2	19.227	BB	0.4488	6571.94531	218.56802	50.2236

Totals : 1.30854e4 541.05270

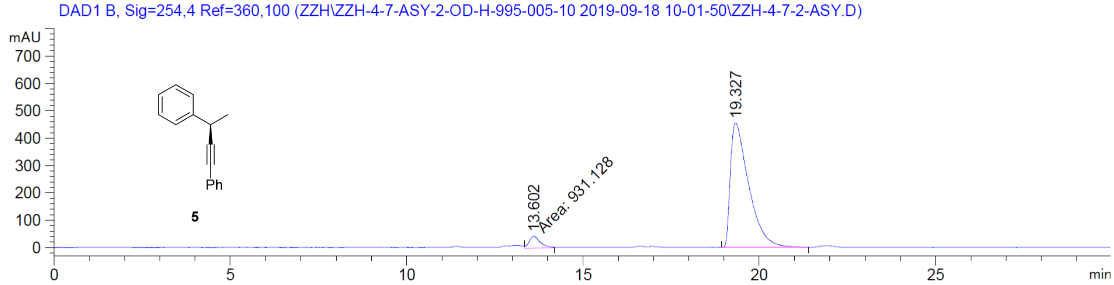

Signal 2: DAD1 B, Sig=254,4 Ref=360,100

Peak #	RetTime [min]	Type	Width [min]	Area [mAU*s]	Height [mAU]	Area %
1	13.602	MM	0.3598	931.12799	43.13540	5.3580
2	19.327	BB	0.5217	1.64473e4	455.01566	94.6420

Totals : 1.73784e4 498.15106

Supplementary Figure 232 HPLC spectra for racemic and chiral **5**. **HPLC** analysis: Chiralcel OD-H (*n*-hexane/*i*-PrOH = 99.5/0.5, flow rate 1.0 mL/min, λ = 254 nm), t_R (minor) = 13.60 min, t_R (major) = 19.33 min, 89% ee.

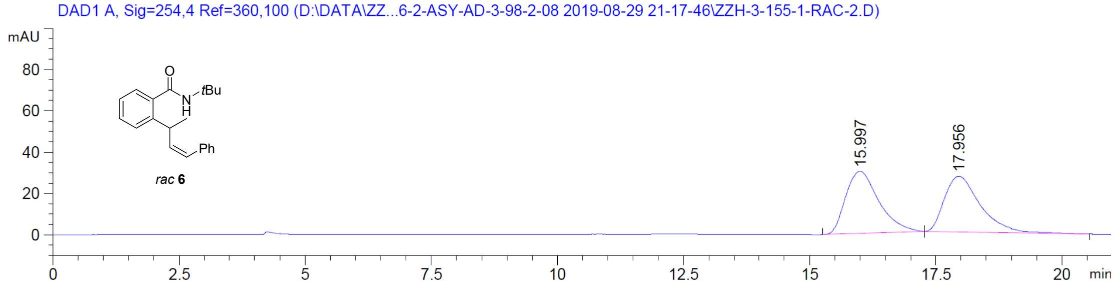

Signal 1: DAD1 A, Sig=254,4 Ref=360,100

Peak #	RetTime [min]	Type	Width [min]	Area [mAU*s]	Height [mAU]	Area %
1	15.997	BB	0.7107	1372.46777	30.05979	50.6360
2	17.956	BB	0.7599	1337.98816	26.91745	49.3640
Totals :				2710.45593	56.97723	

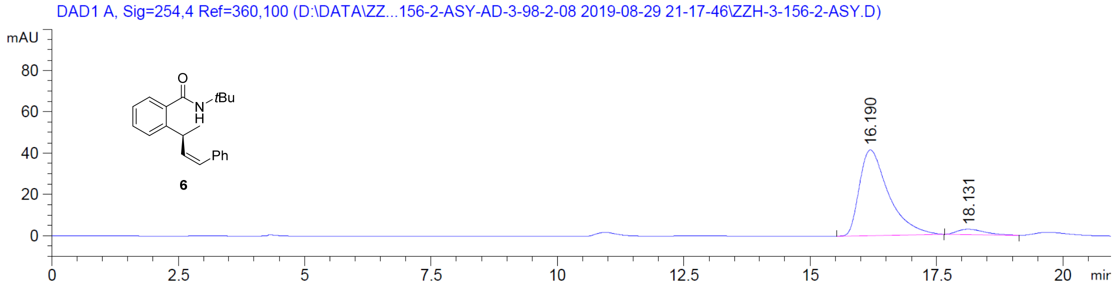

Signal 1: DAD1 A, Sig=254,4 Ref=360,100

Peak #	RetTime [min]	Type	Width [min]	Area [mAU*s]	Height [mAU]	Area %
1	16.190	BB	0.6033	1660.53430	41.45330	94.4607
2	18.131	BB	0.5127	97.37547	2.62540	5.5393
Totals :				1757.90977	44.07869	

Supplementary Figure 233 HPLC spectra for racemic and chiral **6**. **HPLC** analysis: Chiralcel AD3 (*n*-hexane/*i*-PrOH = 98/2, flow rate 0.8 mL/min, λ = 254 nm), t_R (minor) = 16.19 min, t_R (major) = 18.13 min, 89% ee.

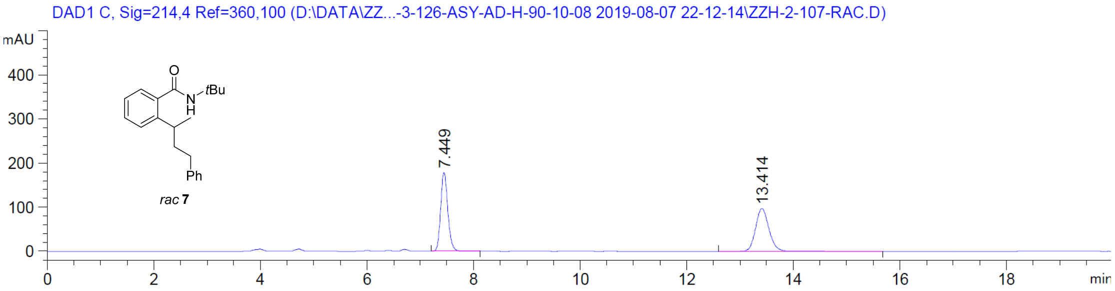

Signal 3: DAD1 C, Sig=214,4 Ref=360,100

Peak #	RetTime [min]	Type	Width [min]	Area [mAU*s]	Height [mAU]	Area %
1	7.449	BV	0.1450	1669.81152	178.74265	49.6147
2	13.414	BB	0.2674	1695.74951	97.26457	50.3853

Totals : 3365.56104 276.00722

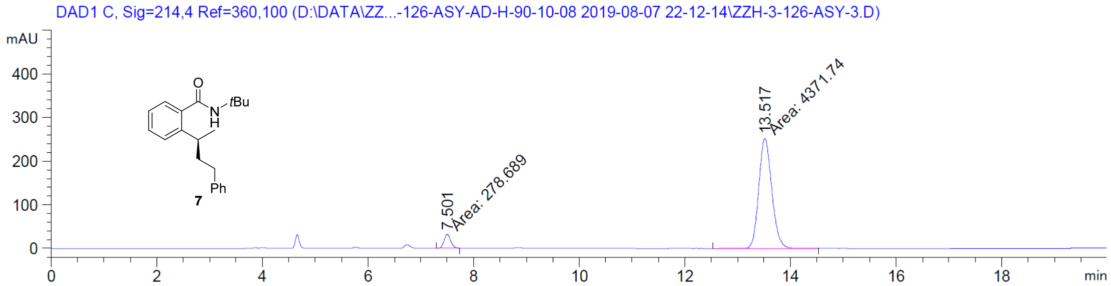

Signal 3: DAD1 C, Sig=214,4 Ref=360,100

Peak #	RetTime [min]	Type	Width [min]	Area [mAU*s]	Height [mAU]	Area %
1	7.501	MM	0.1449	278.68906	32.05468	5.9928
2	13.517	MM	0.2882	4371.73779	252.79807	94.0072

Totals : 4650.42685 284.85275

Supplementary Figure 234 HPLC spectra for racemic and chiral **7**. **HPLC** analysis: Chiralcel AD-H (*n*-hexane/*i*-PrOH = 90/10, flow rate 0.8 mL/min, λ = 214 nm), t_R (minor) = 7.50 min, t_R (major) = 13.52 min, 88% ee.

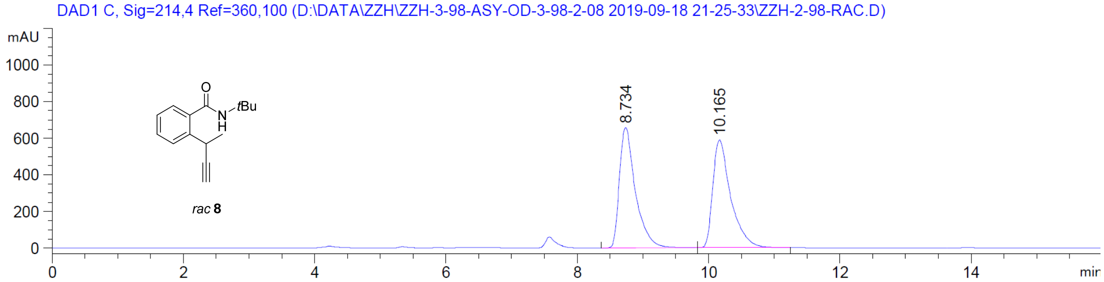

Signal 3: DAD1 C, Sig=214,4 Ref=360,100

Peak #	RetTime [min]	Type	Width [min]	Area [mAU*s]	Height [mAU]	Area %
1	8.734	BB	0.2538	1.11358e4	656.71204	49.9839
2	10.165	BB	0.2828	1.11429e4	588.80389	50.0161

Totals : 2.22787e4 1245.51593

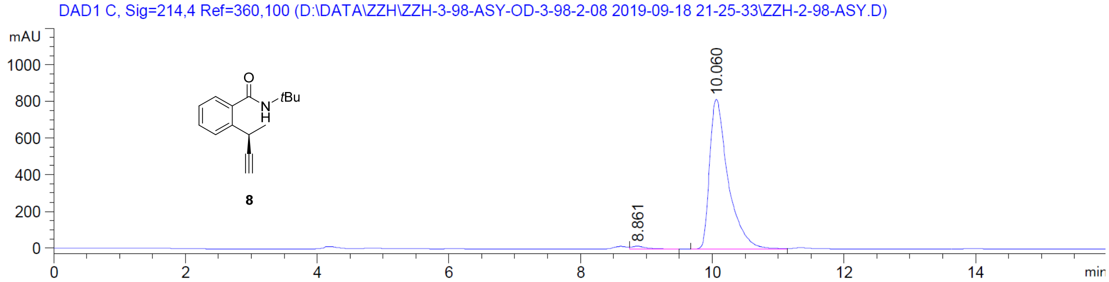

Signal 3: DAD1 C, Sig=214,4 Ref=360,100

Peak #	RetTime [min]	Type	Width [min]	Area [mAU*s]	Height [mAU]	Area %
1	8.861	VB	0.2127	225.50345	15.23015	1.4011
2	10.060	BV	0.2891	1.58697e4	815.42804	98.5989

Totals : 1.60952e4 830.65819

Supplementary Figure 235 HPLC spectra for racemic and chiral **8**. **HPLC** analysis: Chiralcel OD-3 (*n*-hexane/*i*-PrOH = 98/2, flow rate 0.8 mL/min, λ = 214 nm), t_R (minor) = 8.86 min, t_R (major) = 10.06 min, 97% ee.

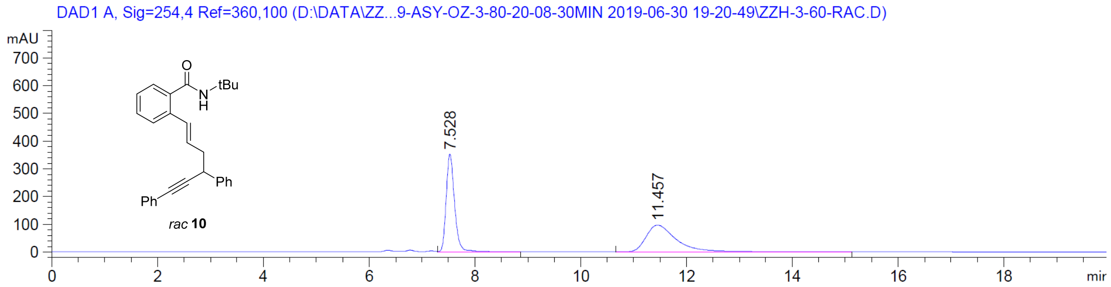

Signal 1: DAD1 A, Sig=254,4 Ref=360,100

Peak #	RetTime [min]	Type	Width [min]	Area [mAU*s]	Height [mAU]	Area %
1	7.528	VB	0.1675	3847.63843	352.16974	50.1541
2	11.457	BB	0.5948	3823.98901	96.39348	49.8459

Totals : 7671.62744 448.56322

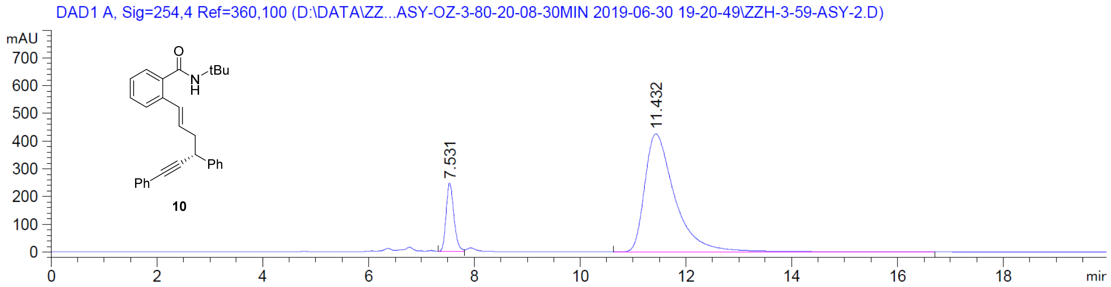

Signal 1: DAD1 A, Sig=254,4 Ref=360,100

Peak #	RetTime [min]	Type	Width [min]	Area [mAU*s]	Height [mAU]	Area %
1	7.531	BV	0.1574	2524.48267	246.66464	13.0781
2	11.432	BB	0.5902	1.67786e4	425.35782	86.9219

Totals : 1.93031e4 672.02246

Supplementary Figure 236 HPLC spectra for racemic and chiral **10**. **HPLC** analysis: Chiralcel OZ3 (*n*-hexane/*i*-PrOH = 80/20, flow rate 0.8 mL/min, λ = 254 nm), t_R (minor) = 7.53 min, t_R (major) = 11.43 min, 74% ee.

Supplementary Methods

Most of reactions were carried out under argon atmosphere using Schlenk techniques. Reagents were purchased at the highest commercial quality and used without further purification, unless otherwise stated. CuI was purchased from Sigma-Aldrich. Analytical thin layer chromatography (TLC) was performed on precoated silica gel 60 GF254 plates. Flash column chromatography was performed using Tsingdao silica gel (60, particle size 0.040–0.063 mm). Visualization on TLC was achieved by use of UV light (254 nm) or iodine. NMR spectra were recorded on Bruker DRX-400 and DPX-500 spectrometers at 400 or 500 MHz for ^{1}H NMR, 100 or 125 MHz for ^{13}C NMR and 376 MHz for ^{19}F NMR, respectively, in $CDCl_3$ with tetramethylsilane (TMS) as internal standard. The chemical shifts are expressed in ppm and coupling constants are given in Hz. Data for ^{1}H NMR are recorded as follows: chemical shift (ppm), multiplicity (s, singlet; d, doublet; t, triplet; q, quarter; p, pentet, m, multiplet; br, broad), coupling constant (Hz), integration. Data for ^{13}C NMR are reported in terms of chemical shift (δ, ppm). Mass spectrometric data were obtained using Bruker Apex IV RTMS. Enantiomeric excess (ee) was determined using Agilent high-performance liquid chromatography (HPLC) with a Hatachi detector (at appropriate wavelength). Column conditions are reported in the experimental section below. Specific optical rotation was measured on a Rudolph-Autopol I.

General synthesis of *N*-(*tert*-butyl)benzamides S-1a–S-1l.

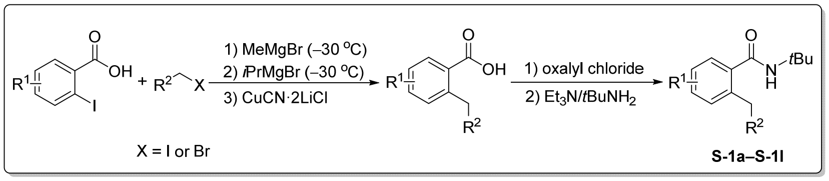

Supplementary Figure 237 General synthesis of *N*-(*tert*-butyl)benzamides **S-1a–S-1l**

According to the literature procedures.[5,6]

To a stirred solution of 2-iodobenzoic acid in THF (0.33 M) in an oven-dried flask was added MeMgBr (1 equiv.) at –30 °C under argon and the reaction mixture was stirred under the same conditions for 5 mins. Next, *i*PrMgCl (1.2 equiv.) was added slowly and the stirring was continued under the same conditions for another 1 h. Then, the reaction mixture was cooled to –40 °C and a solution of CuCN·2LiCl in THF (5 mol%, 0.34 M) was added slowly. The reaction mixture was stirred for 10 mins while being warmed to –30 °C. Subsequently, alkyl bromide or alkyl iodide (3.0 equiv.) was added in one portion and the reaction was allowed to warm to ambient temperature overnight while stirring. Upon completion, the reaction was diluted with EtOAc, acidified with 1 M HCl to pH = 3, and extracted with EtOAc. The combined organic layers were washed with brine and dried over Na_2SO_4. The crude mixture was concentrated and purified by column chromatography on silica gel to yield 2-subsituted benzoic acid.

To a solution of 2-subsituted benzoic acid (1.0 equiv.) and DMF (0.05 equiv.) in DCM (0.3 M) was added oxalyl chloride (1.50 equiv.) dropwise at rt. The reaction was stirred under the same conditions until bubbling stopped. Then, volatiles were removed by rotary evaporation under high vacuum. The crude reaction product was dissolved in DCM (0.3 M) and *tert*-butylamine (1.5 equiv.) as well as triethylamine (2.0 equiv.) were sequentially added at room temperature. The reaction was stirred for 1 to 3 h before being quenched with 1.0 M aqueous HCl. The crude mixture was diluted with DCM (0.1 M) and water (0.1 M). The organic layer was removed and then, the aqueous layer was extracted with DCM. The combined organic layers were washed with saturated aqueous $NaHCO_3$ and brine, dried with $NaSO_4$, filtered, and concentrated by rotary evaporation. The residue thus obtained was purified by silica gel column chromatography (typically 20% EtOAc in hexanes) to afford the pure amide.

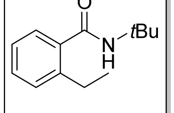

S-1a

***N*-(*tert*-butyl)-2-ethylbenzamide (S-1a)**

White powder, 1.86 g (91% yield). **^{1}H NMR** (400 MHz, $CDCl_3$): δ 7.35-7.27 (m, 2H), 7.23 (dd, *J* = 7.6, 1.4 Hz, 1H), 7.17 (td, *J* = 7.4, 1.4 Hz, 1H), 5.57 (s, 1H), 2.79 (q, *J* = 7.5 Hz, 2H), 1.46 (s, 9H), 1.25 (t, *J* = 7.6 Hz, 3H); **^{13}C NMR** (100 MHz, $CDCl_3$): δ 169.8, 141.8, 137.5, 129.5, 129.3, 126.6, 125.7, 51.8, 28.8, 26.3, 15.9.

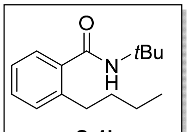

S-1b

***N*-(*tert*-butyl)-2-butylbenzamide (S-1b)**

White powder, 1.00 g (43% yield). **^{1}H NMR** (400 MHz, $CDCl_3$): δ 7.33-7.25 (m, 2H), 7.23-7.10 (m, 2H), 5.57 (s, 1H), 2.81-2.71 (m, 2H), 1.64-1.54 (m, 2H), 1.46 (s, 9H), 1.42-1.35 (m, 2H), 0.92 (t, J = 7.4 Hz, 3H); **^{13}C NMR** (100 MHz, $CDCl_3$): δ 169.9, 140.4, 137.8, 130.0, 129.3, 126.6, 125.6, 51.7, 33.9, 33.0, 28.8, 22.8, 14.0.
HRMS (ESI) m/z calcd. for $C_{15}H_{24}NO$ $[M+H]^+$ 234.1852, found 234.1851.

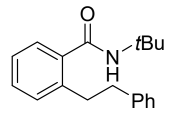

S-1c

***N*-(*tert*-butyl)-2-phenethylbenzamide (S-1c)**
White powder, 1.55 g (55% yield). **^{1}H NMR** (400 MHz, $CDCl_3$): δ 7.33-7.23 (m, 4H), 7.22-7.14 (m, 5H), 5.47 (s, 1H), 3.11-3.00 (m, 2H), 2.96-2.88 (m, 2H), 1.44 (s, 9H); **^{13}C NMR** (100 MHz, $CDCl_3$): δ 169.8, 141.9, 139.5, 138.0, 130.2, 129.5, 128.6, 128.3, 126.6, 126.0, 125.9, 51.7, 38.1, 35.5, 28.8.

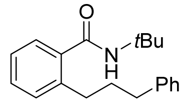

S-1d

***N*-(*tert*-butyl)-2-(3-phenylpropyl)benzamide (S-1d)**
White powder, 1.13 g (38% yield). **^{1}H NMR** (400 MHz, $CDCl_3$): δ 7.33-7.24 (m, 4H), 7.23-7.14 (m, 5H), 5.53 (s, 1H), 2.86-2.78 (m, 2H), 2.71-2.63 (m, 2H), 2.01-1.90 (m, 2H), 1.44 (s, 9H); **^{13}C NMR** (100 MHz, $CDCl_3$): δ 169.8, 142.2, 140.0, 137.9, 130.0, 129.4, 128.4, 128.3, 126.6, 125.8, 125.7, 51.8, 35.9, 33.2, 32.9, 28.8.

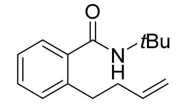

S-1e

2-(but-3-en-1-yl)-*N*-(*tert*-butyl)benzamide (S-1e)
White powder, 0.80 g (35% yield). **^{1}H NMR** (400 MHz, $CDCl_3$): δ 7.38-7.27 (m, 2H), 7.25-7.11 (m, 2H), 5.91-5.79 (m, 1H), 5.57 (s, 1H), 5.06-4.99 (m, 1H), 4.98-4.93 (m, 1H), 2.94-2.82 (m, 2H), 2.45-2.29 (m, 2H), 1.46 (s, 9H); **^{13}C NMR** (100 MHz, $CDCl_3$): δ 169.8, 139.4, 138.1, 137.9, 130.1, 129.4, 126.6, 125.9, 115.0, 51.8, 35.6, 32.6, 28.8.

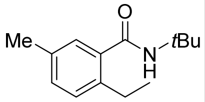

S-1f

***N*-(*tert*-butyl)-2-ethyl-5-methylbenzamide (S-1f)**
White powder, 1.60 g (73% yield). **^{1}H NMR** (400 MHz, $CDCl_3$): δ 7.14-7.08 (m, 3H), 5.55 (s, 1H), 2.74 (q, J = 7.5 Hz, 2H), 2.31 (s, 3H), 1.46 (s, 9H), 1.22 (t, J = 7.6 Hz, 3H); **^{13}C NMR** (100 MHz, $CDCl_3$): δ 170.0, 138.7, 137.5, 135.2, 130.2, 129.2, 127.2, 51.7, 28.8, 25.8, 20.8, 16.0.
HRMS (ESI) m/z calcd. for $C_{14}H_{22}NO$ $[M+H]^+$ 220.1696, found 220.1695.

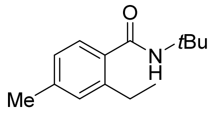

S-1g

***N*-(*tert*-butyl)-2-ethyl-4-methylbenzamide (S-1g)**
White powder, 1.51 g (69% yield). **^{1}H NMR** (400 MHz, $CDCl_3$): δ 7.17 (d, J = 7.8 Hz, 1H), 7.02 (d, J = 1.8 Hz, 1H), 6.95 (dd, J = 7.8, 1.8 Hz, 1H), 5.61 (s, 1H), 2.76 (q, J = 7.6 Hz, 2H), 2.32 (s, 3H), 1.44 (s, 9H), 1.22 (t, J = 7.6 Hz, 3H); **^{13}C NMR** (100 MHz, $CDCl_3$): δ 169.9, 141.9, 139.4, 134.8, 130.1, 126.7, 126.2, 51.6, 28.8, 26.3, 21.3, 15.9.
HRMS (ESI) m/z calcd. for $C_{14}H_{22}NO$ $[M+H]^+$ 220.1696, found 220.1695.

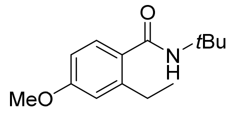

S-1h

N-(*tert*-butyl)-2-ethyl-4-methoxybenzamide (S-1h)

White powder, 1.12 g (48% yield). **^{1}H NMR** (400 MHz, $CDCl_3$): δ 7.24 (d, J = 8.4 Hz, 1H), 6.74 (d, J = 2.6 Hz, 1H), 6.67 (dd, J = 8.4, 2.6 Hz, 1H), 5.59 (s, 1H), 3.79 (s, 3H), 2.79 (q, J = 7.6 Hz, 2H), 1.44 (s, 9H), 1.23 (t, J = 7.6 Hz, 3H); **^{13}C NMR** (100 MHz, $CDCl_3$): δ 169.6, 160.4, 144.2, 130.2, 128.3, 115.0, 110.5, 55.2, 51.6, 28.8, 26.6, 15.7.

HRMS (ESI) m/z calcd. for $C_{14}H_{22}NO_2$ $[M+H]^+$ 236.1645, found 236.1643.

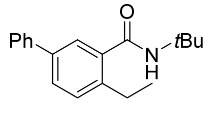

S-1i

N-(*tert*-butyl)-4-ethyl-[1,1'-biphenyl]-3-carboxamide (S-1i)

Orange powder, 0.67 g (48 % yield). **^{1}H NMR** (400 MHz, $CDCl_3$): δ 7.57-7.48 (m, 4H), 7.46-7.39 (m, 2H), 7.37-7.31 (m, 1H), 7.29 (d, J = 8.0 Hz, 1H), 5.65 (s, 1H), 2.82 (q, J = 7.6 Hz, 2H), 1.47 (s, 9H), 1.28 (t, J = 7.6 Hz, 3H); **^{13}C NMR** (100 MHz, $CDCl_3$): δ 169.8, 140.8, 140.4, 138.8, 138.1, 129.8, 128.8, 128.1, 127.4, 127.0, 125.3, 51.9, 28.9, 26.0, 15.8.

HRMS (ESI) m/z calcd. for $C_{19}H_{24}NO$ $[M+H]^+$ 282.1852, found 282.1851.

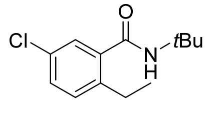

S-1j

N-(*tert*-butyl)-5-chloro-2-ethylbenzamide (S-1j)

White powder, 1.25 g (53% yield). **^{1}H NMR** (400 MHz, $CDCl_3$): δ 7.23-7.19 (m, 2H), 7.14 (dd, J = 8.2, 2.0 Hz, 1H), 5.57 (s, 1H), 2.76 (q, J = 7.6 Hz, 2H), 1.45 (s, 9H), 1.23 (t, J = 7.6 Hz, 3H); **^{13}C NMR** (100 MHz, $CDCl_3$): δ 168.8, 144.0, 135.9, 135.3, 129.3, 127.9, 125.8, 51.9, 28.8, 26.1, 15.5.

HRMS (ESI) m/z calcd. for $C_{13}H_{19}{}^{34.9689}ClNO$ $[M+H]^+$ 240.1150, found 240.1149; calcd. for $C_{13}H_{19}{}^{36.9659}ClNO$ $[M+H]^+$ 242.1120, found 240.1118.

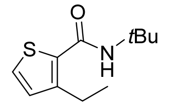

S-1k

N-(*tert*-butyl)-3-ethylthiophene-2-carboxamide (S-1k)

Orange powder, 1.40 g (66% yield). **^{1}H NMR** (400 MHz, $CDCl_3$): δ 7.22 (d, J = 5.1 Hz, 1H), 6.93 (d, J = 5.1 Hz, 1H), 5.66 (s, 1H), 2.92 (q, J = 7.6 Hz, 2H), 1.45 (s, 9H), 1.26 (t, J = 7.6 Hz, 3H); **^{13}C NMR** (100 MHz, $CDCl_3$): δ 162.5, 146.8, 131.8, 130.2, 125.8, 51.9, 28.9, 22.8, 15.0.

HRMS (ESI) m/z calcd. for $C_{11}H_{18}NOS$ $[M+H]^+$ 212.1104, found 212.1103.

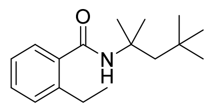

S-1l

2-ethyl-N-(2,4,4-trimethylpentan-2-yl)benzamide (S-1l)

White powder, 2.09 g (80% yield). **^{1}H NMR** (400 MHz, $CDCl_3$): δ 7.37-7.29 (m, 2H), 7.28-7.24 (m, 1H), 7.20 (td, J = 7.4, 1.4 Hz, 1H), 5.59 (s, 1H), 2.83 (q, J = 7.6 Hz, 2H), 1.88 (s, 2H), 1.54 (s, 6H), 1.27 (t, J = 7.6 Hz, 3H), 1.08 (s, 9H); **^{13}C NMR** (100 MHz, $CDCl_3$): δ 169.4, 142.1, 137.7, 129.5, 129.4, 126.3, 125.7, 55.8, 51.9, 31.7, 31.6, 29.2, 26.2, 15.9.

HRMS (ESI) m/z calcd. for $C_{17}H_{28}NO$ $[M+H]^+$ 262.2165, found 262.2164.

General synthesis of *N*-alkylbenzamides S-1m–S-1q

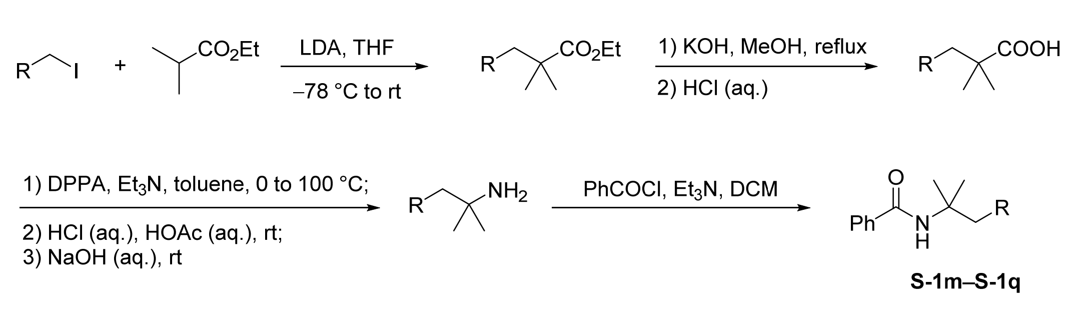

Supplementary Figure 238 General synthesis of *N*-alkylbenzamides **S-1m–S-1q**

According to the literature procedures.[5,7]

To a solution of freshly distilled diisopropylamine (1.05 equiv.) in anhydrous THF (0.33 M) was added *n*-butyllithium (2.4 M, 1.05 equiv.) dropwise at −78 °C under argon. Upon completion, the reaction mixture was allowed to warm to 0 °C and stirred for 0.5 h. Next, the reaction solution was cooled to −78 °C and ethyl isobutyrate (1.0 equiv.) was added dropwise. The stirring was continued for 1 h at −78 °C and then, alkyl iodide (1.0 equiv.) was added. The stirring was continued overnight at room temperature. Upon completion, the reaction mixture was poured into ice-water and extracted with diethyl ether. The combined organic phase was washed with brine and dried over anhydrous $MgSO_4$. Evaporation of organic solvent gave the crude product ester without further purification for the next step.

The crude ester was dissolved in methanol (1.0 M) and 30% aqueous NaOH (2.5 equiv.) was added. The reaction solution was then refluxed for 2 h. After being cooled, the mixture was diluted with water and extracted once with diethyl ether. Then, the aqueous solution was acidified with 10% HCl at 0 °C until pH = 1. The mixture was extracted with diethyl ether. The combined organic phase was washed with brine and dried over anhydrous Na_2SO_4. Evaporation of organic solvent gave the crude acid without further purification for the next step.

To a stirred solution of the crude acid (1.0 equiv.) and triethylamine (1.05 equiv.) in dry toluene (0.2 M) was added diphenylphosphoryl azide (DPPA) (1.05 equiv) at 0 °C for 1 h under argon. The reaction was stirred for 1 h at rt and then refluxed for 12 h (evolution of N_2) under argon. Upon completion, the solution was cooled and washed with water. The toluene phase was separated and dried over anhydrous Na_2SO_4. Next, toluene was removed by evaporation and a mixture of 15 % HCl (10 mL) and acetic acid (10 mL) was added. After about 10 mins of a rapid evolution of CO_2, the reaction mixture was stirred at rt overnight. Upon completion, the mixture was extracted once with diethyl ether and then, the aqueous solution was basified by a cooled 10% aqueous solution of NaOH until pH =13. The mixture was extracted with diethyl ether. The combined extracts were washed with water and brine and dried over anhydrous Na_2SO_4. Evaporation of organic solvent gave the crude amine without further purification for the next

step.

To a solution of the crude amine in DCM (0.3 M) were sequentially added benzoyl chloride (1.5 equiv.) and triethylamine (1.5 equiv.) at rt. The reaction mixture was stirred for 1 to 3 h under the same conditions. Upon completion, the mixture was quenched with 1 M aqueous HCl and transferred to a separatory funnel. The crude mixture was diluted with DCM and water. The organic layer was removed and the aqueous layer was extracted with DCM. The combined organic layers were washed with saturated aqueous $NaHCO_3$ and then brine. The organic layer was dried with $NaSO_4$, filtered, and concentrated by rotary evaporation. The residue thus obtained was purified by silica gel column chromatography (typically 20% EtOAc in hexanes) to afford the pure amide.

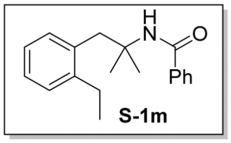

***N*-(1-(2-ethylphenyl)-2-methylpropan-2-yl)benzamide (S-1m)**

White powder, 1.46 g (26% yield). **^{1}H NMR** (400 MHz, $CDCl_3$): δ 7.71-7.64 (m, 2H), 7.49-7.44 (m, 1H), 7.42-7.37 (m, 2H), 7.25-7.11 (m, 3H), 7.07 (td, *J* = 7.2, 1.6 Hz, 1H), 5.84 (s, 1H), 3.23 (s, 2H), 2.75 (q, *J* = 7.5 Hz, 2H), 1.47 (s, 6H), 1.20 (t, *J* = 7.5 Hz, 3H); **^{13}C NMR** (100 MHz, $CDCl_3$): δ 167.3, 143.4, 136.0, 135.4, 131.5, 131.2, 128.7, 128.5, 126.8, 126.7, 125.4, 55.2, 40.4, 27.5, 26.0, 15.6.

HRMS (ESI) m/z calcd. for $C_{19}H_{24}NO$ $[M+H]^+$ 282.1852, found 282.1851.

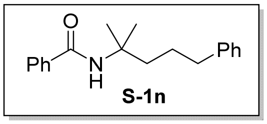

***N*-(2-methyl-5-phenylpentan-2-yl)benzamide (S-1n)**

White powder, 2.18 g (39% yield). **^{1}H NMR** (400 MHz, $CDCl_3$): δ 7.70-7.67 (m, 2H), 7.49-7.44 (m, 1H), 7.43-7.37 (m, 2H), 7.29-7.26 (m, 2H), 7.20-7.14 (m, 3H), 5.81 (s, 1H), 2.63 (t, *J* = 7.7 Hz, 2H), 1.93-1.86 (m, 2H), 1.69-1.60 (m, 2H), 1.42 (s, 6H); **^{13}C NMR** (100 MHz, $CDCl_3$) δ 166.9, 142.5, 135.9, 131.1, 128.5, 128.40, 128.36, 126.7, 125.8, 54.1, 39.9, 36.2, 27.1, 26.4.

HRMS (ESI) m/z calcd. for $C_{19}H_{24}NO$ $[M+H]^+$ 282.1852, found 282.1850.

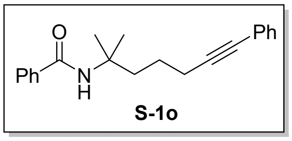

***N*-(2-methyl-7-phenylhept-6-yn-2-yl)benzamide (S-1o)**

Yellow oil, 1.35 g (22% yield). **^{1}H NMR** (400 MHz, $CDCl_3$): δ 7.76-7.69 (m, 2H), 7.49-7.35 (m, 5H), 7.29-7.24 (m, 3H), 5.96 (s, 1H), 2.54-2.34 (m, 2H), 2.12-1.92 (m, 2H), 1.78-1.59 (m, 2H), 1.47 (s, 6H); **^{13}C NMR** (100 MHz, $CDCl_3$): δ 167.0, 135.8, 131.6, 131.2, 128.5, 128.2, 127.6, 126.7, 123.9, 90.0, 81.0, 54.0, 39.6, 27.1, 23.9, 19.7.

HRMS (ESI) m/z calcd. for $C_{21}H_{24}NO$ $[M+H]^+$ 306.1852, found 306.1850.

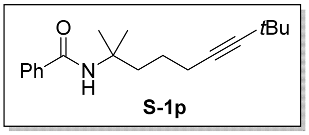

***N*-fluoro-*N*-(2,8,8-trimethylnon-6-yn-2-yl)benzamide (S-1p)**

Yellow oil, 2.00 g (35% yield). **^{1}H NMR** (400 MHz, $CDCl_3$): δ 7.75-7.69 (m, 2H), 7.52-7.37 (m, 3H), 5.89 (s, 1H), 2.17 (t, *J* = 7.1 Hz, 2H), 1.93-1.84 (m, 2H), 1.59-1.47 (m, 2H), 1.45 (s, 6H), 1.19 (s, 9H); **^{13}C**

NMR (100 MHz, $CDCl_3$): δ 166.8, 135.9, 131.1, 128.5, 126.7, 89.5, 78.2, 54.0, 39.7, 31.4, 27.3, 27.0, 24.3, 19.0.
HRMS (ESI) m/z calcd. for $C_{19}H_{28}NO$ $[M+H]^+$ 286.2165, found 286.2164.

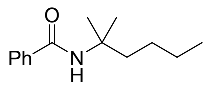

S-1q

***N*-(2-methylhexan-2-yl)benzamide (S-1q)**
White powder, 1.75 g (40% yield). **^{1}H NMR** (400 MHz, $CDCl_3$): δ 7.75-7.66 (m, 2H), 7.51-7.37 (m, 3H), 5.84 (s, 1H), 1.85-1.76 (m, 2H), 1.43 (s, 6H), 1.41-1.23 (m, 4H), 0.91 (t, J = 7.0 Hz, 3H); **^{13}C NMR** (100 MHz, $CDCl_3$): δ 166.8, 136.1, 131.1, 128.5, 126.7, 54.1, 40.2, 27.0, 26.4, 23.1, 14.1.
HRMS (ESI) m/z calcd. for $C_{14}H_{22}NO$ $[M+H]^+$ 220.1696, found 220.1695.

General synthesis of *N*-alkylbenzamides [D$_1$]-S-1aa and [D$_2$]-S-1aa

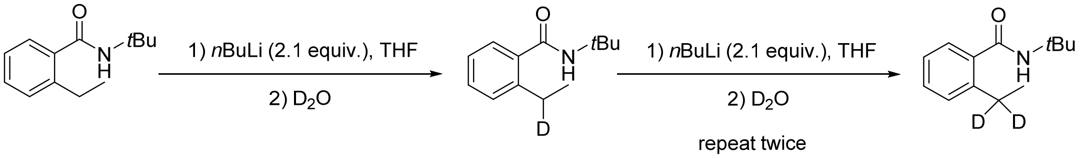

1aa **[D$_1$]-1aa** **[D$_2$]-1aa**

Supplementary Figure 239 General synthesis of **[D$_1$]-S-1aa and [D$_2$]-S-1aa**

To a flame-dried 50 mL round-bottom flask with a stir bar was added **S-1aa** (10 mmol). The contents were evacuated and backfilled three times with argon. THF (20 mL, 0.2 M) was added via syringe and the flask was cooled on an ice bath for 15 minutes. *n*-Butyllithium (2.8 mL, 2.1 equiv., 2.2 M in hexanes) was added dropwise. The blood-red solution was stirred at 0 °C for 45 minutes, and then quenched with D_2O (4 mL). The biphasic mixture was transferred to a separatory funnel and diluted with DCM and brine. The organic layer was removed, and the aqueous layer was extracted with DCM. The combined organic layers were dried with $MgSO_4$, filtered, and concentrated by rotary evaporation to afford [**D$_1$**]-**S-1aa**. ^{1}H NMR analysis showed >95% D-incorporation. The compound **[D$_2$]-S-1aa** was prepared following the same procedure described above using [**D$_1$**]-**S-1aa** (5 mmol) as substrate, and ^{1}H NMR analysis showed >95% D-incorporation after the procedure was repeated twice.

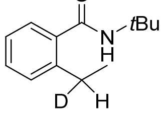

[D$_1$]-S-1aa

***N*-(*tert*-butyl)-2-(ethyl-1-*d*)benzamide ([D$_1$]-S-1aa)**
White powder, 2.06 g (100% yield). **^{1}H NMR** (400 MHz, $CDCl_3$): δ 7.34-7.27 (m, 2H), 7.25-7.21 (m, 1H), 7.17 (td, J = 7.4, 1.4 Hz, 1H), 5.56 (s, 1H), 2.82-2.72 (m, 1H), 1.46 (s, 9H), 1.24 (d, J = 7.6 Hz, 2H); **^{13}C NMR** (100 MHz, $CDCl_3$): δ 169.8, 141.8, 137.6, 129.5, 129.3, 126.5, 125.6, 51.7, 28.8, 25.9 (t, J = 19.5 Hz), 15.8.
HRMS (ESI) m/z calcd. for $C_{13}H_{19}DNO$ $[M+H]^+$ 207.1602, found 207.1602.

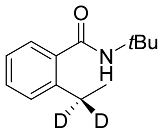

[D$_2$]-S-1aa

***N*-(*tert*-butyl)-2-(ethyl-1,1-*d*$_2$)benzamide ([D$_2$]-S-1aa)**

White powder, 1.01 g (97% yield). ^{1}**H NMR** (400 MHz, $CDCl_3$): δ 7.34-7.25 (m, 2H), 7.24-7.21 (m, 1H), 7.17 (td, *J* = 7.4, 1.2 Hz, 1H), 5.56 (s, 1H), 1.46 (s, 9H), 1.23 (s, 3H); ^{13}**C NMR** (100 MHz, $CDCl_3$): δ 169.8, 141.8, 137.6, 129.6, 129.3, 126.5, 125.7, 51.7, 28.84, 28.82, 25.6 (p, *J* = 18.8 Hz), 15.7.
HRMS (ESI) m/z calcd. for $C_{13}H_{18}D_2NO$ $[M+H]^+$ 208.1665, found 208.1664.

General procedure for the synthesis of *N*-fluoro-*N*-alkylcarboxamides.

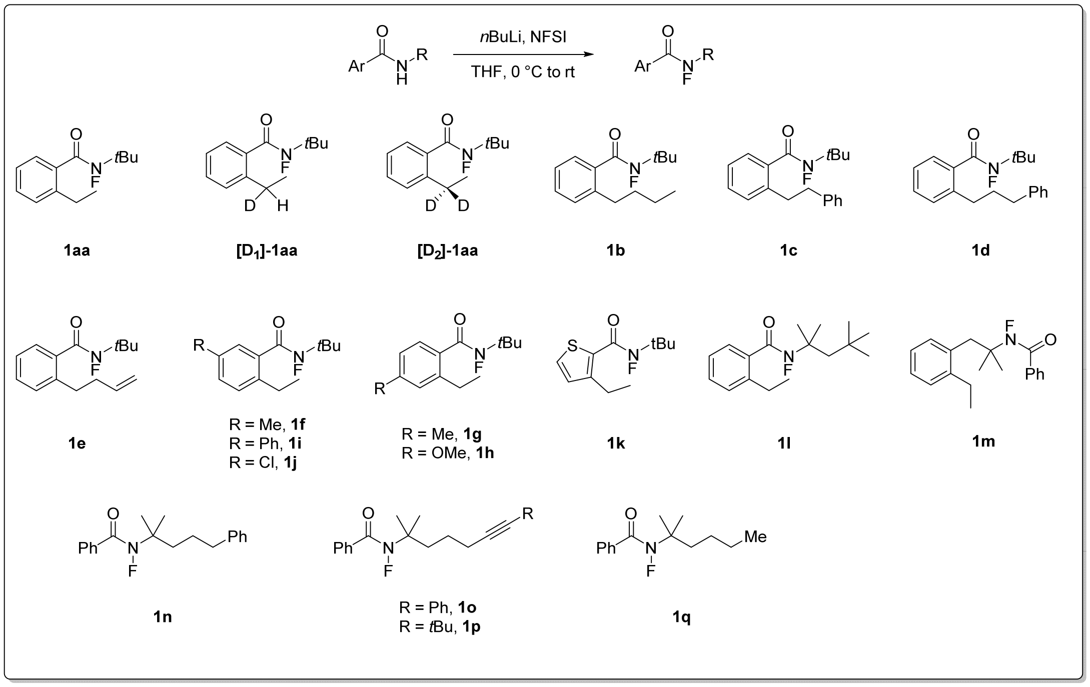

Supplementary Figure 240 General synthesis of *N*-fluoro-*N*-alkylcarboxamides.

All the *N*-fluoro-*N*-alkylcarboxamides were prepared by *N*-fluorination of their parent carboxamides according to conventional methods.[5,8] To a flame-dried round-bottom flask with a stir bar was added amide (1.0 equiv.). The contents were evacuated and backfilled three times with argon. Anhydrous THF (0.13 M) was added and the stirred solution was cooled on an ice bath for 15 min. *n*-Butyllithium (1.1 equiv., 2.4 M in hexanes) was added dropwise. The reaction was maintained at 0 °C for 1.5 h. NFSI (1.5 equiv., 0.6 M in THF) was added dropwise. The reaction was left overnight in the ice bath and allowed to warm to rt. After 10 to 14 h, the reaction was quenched with 1 M aqueous HCl and transferred to a separatory funnel. The crude mixture was diluted with DCM (0.1 M) and water (0.1 M). The organic layer was removed, and the aqueous layer was extracted with DCM. The combined organic layers were washed with saturated aqueous $NaHCO_3$ and then brine, dried with $MgSO_4$, filtered, and concentrated by rotary evaporation. The residue thus obtained was purified by silica gel column chromatography (15% EtOAc in hexanes) to afford pure fluoroamides.

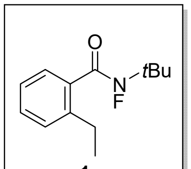

1aa

***N*-(*tert*-butyl)-2-ethyl-*N*-fluorobenzamide (1aa)**

1aa was synthesized according to the procedures.[5]
Yellow oil, 590 mg (27% yield). **^{1}H NMR** (400 MHz, $CDCl_3$): δ 7.38 (td, *J* = 7.4, 1.4 Hz, 1H), 7.34-7.28 (m, 2H), 7.24 (td, *J* = 7.4, 1.4 Hz, 1H), 2.77 (q, *J* = 7.6 Hz, 2H), 1.58 (d, *J* = 2.0 Hz, 9H), 1.27 (t, *J* = 7.6 Hz, 3H); **^{13}C NMR** (100 MHz, $CDCl_3$): δ 175.0 (d, *J* = 10.9 Hz), 141.6 (d, *J* = 2.2 Hz), 134.6, 130.0 (d, *J* = 1.1 Hz), 128.9, 127.1 (d, *J* = 4.4 Hz), 125.4, 64.3 (d, *J* = 10.5 Hz), 27.1 (d, *J* = 5.6 Hz), 26.1, 15.7; **^{19}F NMR** (376 MHz, $CDCl_3$): δ -63.36.

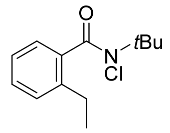

1ab

***N*-(*tert*-butyl)-*N*-chloro-2-ethylbenzamide (1ab)**
1ab was synthesized according to the procedures.[5]
Colorless oil, 370 mg (21% yield). **^{1}H NMR** (400 MHz, $CDCl_3$): δ 7.36-7.31 (m, 1H), 7.28-7.20 (m, 3H), 2.73 (q, *J* = 7.6 Hz, 2H), 1.64 (s, 9H), 1.28 (t, *J* = 7.6 Hz, 3H); **^{13}C NMR** (100 MHz, $CDCl_3$): δ 174.6, 140.5, 137.2, 129.3, 128.7, 126.3, 125.5, 64.5, 28.6, 26.0, 15.1.

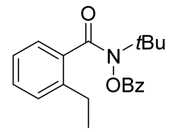

1ac

***N*-(benzoyloxy)-*N*-(*tert*-butyl)-2-ethylbenzamide (1ac)**
1ab was synthesized according to the procedures.[9]
Colorless oil, 490 mg (41% yield). **^{1}H NMR** (400 MHz, $CDCl_3$): δ 7.55-7.51 (m, 2H), 7.43-7.34 (m, 2H), 7.32-7.26 (m, 3H), 7.23-7.18 (m, 1H), 7.13 (td, *J* = 7.6, 1.2 Hz, 1H), 2.70-2.60 (m, 2H), 1.61 (s, 9H), 1.00 (t, *J* = 7.5 Hz, 3H); **^{13}C NMR** (100 MHz, $CDCl_3$): δ 171.7, 166.0, 146.8, 136.4, 132.9, 130.2, 130.0, 129.7, 127.9, 127.2, 125.9, 125.6, 62.8, 27.7, 26.7, 15.6.
HRMS (ESI) m/z calcd. for $C_{20}H_{24}NO_3$ $[M+H]^+$ 326.1751, found 326.1746.

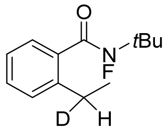

[D$_1$]-1aa

***N*-(*tert*-butyl)-2-(ethyl-1-*d*)-*N*-fluorobenzamide ([D$_1$]-1aa)**
Yellow oil, 390 mg (35% yield). **^{1}H NMR** (400 MHz, $CDCl_3$): δ 7.37-7.32 (m, 1H), 7.31-7.24 (m, 2H), 7.20 (td, *J* = 7.5, 1.4 Hz, 1H), 2.76-2.67 (m, 1H), 1.55 (d, *J* = 2.0 Hz, 9H), 1.25-1.21 (m, 3H); **^{13}C NMR** (100 MHz, $CDCl_3$): δ 175.0 (d, *J* = 10.8 Hz), 141.6 (d, *J* = 2.0 Hz), 134.7, 130.0 (d, *J* = 1.4 Hz), 128.9, 127.1 (d, *J* = 4.4 Hz), 125.4, 64.3 (d, *J* = 10.5 Hz), 27.2 (d, *J* = 5.5 Hz), 25.8 (t, *J* = 19.5 Hz), 15.6; **^{19}F NMR** (376 MHz, $CDCl_3$) δ -63.39.
HRMS (ESI) m/z calcd. for $C_{13}H_{18}DFNO$ $[M+H]^+$ 225.1508, found 225.1505.

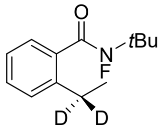

[D$_2$]-1aa

***N*-(*tert*-butyl)-2-(ethyl-1,1-*d*$_2$)-*N*-fluorobenzamide ([D$_2$]-1aa)**
Yellow oil, 310 mg (29% yield). **^{1}H NMR** (400 MHz, $CDCl_3$): δ 7.37-7.32 (m, 1H), 7.31-7.23 (m, 2H), 7.23-7.18 (m, 1H), 1.55 (d, *J* = 2.0 Hz, 9H), 1.22 (s, 3H); **^{13}C NMR** (100 MHz, $CDCl_3$): δ 175.0 (d, *J* = 10.9 Hz), 141.6, 134.6, 130.0 (d, *J* = 1.4 Hz), 128.9, 127.1 (d, *J* = 4.4 Hz), 125.4, 64.3 (d, *J* = 10.5 Hz), 27.2 (d, *J* = 5.7 Hz), 25.4 (p, *J* = 18.7 Hz),15.5; **^{19}F NMR** (376 MHz, $CDCl_3$) δ -63.4.
HRMS (ESI) m/z calcd. for $C_{13}H_{17}D_2FNO$ $[M+H]^+$ 226.1571, found 226.1568.

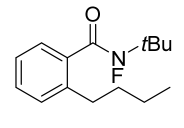

1b

_N_-(_tert_-butyl)-2-butyl-N-fluorobenzamide (1b)

Yellow oil, 440 mg (41% yield). **^{1}H NMR** (400 MHz, $CDCl_3$): δ 7.35-7.27 (m, 2H), 7.26-7.17 (m, 2H), 2.77-2.65 (m, 2H), 1.62-1.56 (m, 2H), 1.55 (d, *J* = 2.0 Hz, 9H), 1.42-1.32 (m, 2H), 0.92 (t, *J* = 7.3 Hz, 3H); **^{13}C NMR** (100 MHz, $CDCl_3$): δ 175.0 (d, *J* = 10.9 Hz), 140.4 (d, *J* = 2.1 Hz), 134.8, 129.8, 129.6, 127.2 (d, *J* = 4.3 Hz), 125.3, 64.2 (d, *J* = 10.5 Hz), 33.7, 32.8, 27.2 (d, *J* = 5.6 Hz), 22.7, 13.9; **^{19}F NMR** (376 MHz, $CDCl_3$): δ -63.35.

HRMS (ESI) m/z calcd. for $C_{15}H_{23}FNO$ $[M+H]^+$ 252.1758, found 252.1754.

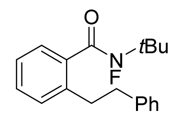

1c

_N_-(_tert_-butyl)-_N_-fluoro-2-phenethylbenzamide (1c)

Yellow oil, 420 mg (26% yield). **^{1}H NMR** (400 MHz, $CDCl_3$): δ 7.44-7.08 (m, 9H), 3.05-2.96 (m, 2H), 2.96-2.86 (m, 2H), 1.56 (d, *J* = 1.8 Hz, 9H); **^{13}C NMR** (100 MHz, $CDCl_3$): δ 174.9 (d, *J* = 10.8 Hz), 141.7, 139.3 (d, *J* = 2.0 Hz), 134.8, 130.0, 129.9, 128.45, 128.38, 127.5 (d, *J* = 4.5 Hz), 126.0, 125.7, 64.3 (d, *J* = 10.5 Hz), 38.0, 35.5, 27.2 (d, *J* = 5.6 Hz); **^{19}F NMR** (376 MHz, $CDCl_3$): δ -63.08.

HRMS (ESI) m/z calcd. for $C_{19}H_{23}FNO$ $[M+H]^+$ 300.1758, found 300.1755.

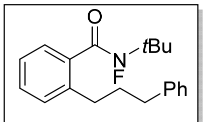

1d

_N_-(_tert_-butyl)-_N_-fluoro-2-(3-phenylpropyl)benzamide (1d)

Yellow oil, 430 mg (26% yield). **^{1}H NMR** (400 MHz, $CDCl_3$): δ 7.37-7.20 (m, 6H), 7.20-7.14 (m, 3H), 2.79-2.70 (m, 2H), 2.66 (t, *J* = 7.7 Hz, 2H), 2.01-1.90 (m, 2H), 1.52 (d, *J* = 1.8 Hz, 9H); **^{13}C NMR** (100 MHz, $CDCl_3$): δ 174.9 (d, *J* = 10.9 Hz), 142.1, 139.8 (d, *J* = 2.0 Hz), 134.9, 129.9, 129.6, 128.5, 128.3, 127.3 (d, *J* = 4.4 Hz), 125.8, 125.5, 64.3 (d, *J* = 10.4 Hz), 35.8, 33.0, 32.8, 27.1 (d, *J* = 5.6 Hz); **^{19}F NMR** (376 MHz, $CDCl_3$): δ -63.28.

HRMS (ESI) m/z calcd. for $C_{20}H_{25}FNO$ $[M+H]^+$ 314.1915, found 314.1911.

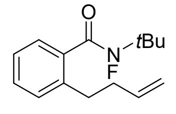

1e

2-(but-3-en-1-yl)-_N_-(_tert_-butyl)-_N_-fluorobenzamide (1e)

Yellow oil, 250 mg (29% yield). **^{1}H NMR** (400 MHz, $CDCl_3$): δ 7.38-7.29 (m, 2H), 7.27-7.18 (m, 2H), 5.90-5.78 (m, 1H), 5.08-5.93 (m, 2H), 2.85-2.75 (m, 2H), 2.43-2.29 (m, 2H), 1.55 (d, *J* = 1.9 Hz, 9H); **^{13}C NMR** (100 MHz, $CDCl_3$): δ 174.8 (d, *J* = 10.9 Hz), 139.3 (d, *J* = 2.1 Hz), 137.9, 134.9, 129.9 (d, *J* = 1.4 Hz), 129.7, 127.3 (d, *J* = 4.4 Hz), 125.6, 115.0, 64.3 (d, *J* = 10.5 Hz), 35.5, 32.5, 27.2 (d, *J* = 5.6 Hz); **^{19}F NMR** (376 MHz, $CDCl_3$) δ -63.26.

HRMS (ESI) m/z calcd. for $C_{15}H_{21}FNO$ $[M+H]^+$ 250.1602, found 250.1599.

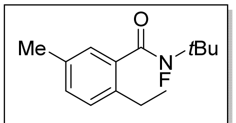

1f

_N_-(_tert_-butyl)-2-ethyl-_N_-fluoro-5-methylbenzamide (1f)

Yellow oil, 730 mg (42% yield). **^{1}H NMR** (400 MHz, $CDCl_3$): δ 7.18-7.07 (m, 3H), 2.69 (q, *J* = 7.5 Hz, 2H), 2.32 (s, 3H), 1.55 (d, *J* = 2.0 Hz, 9H), 1.21 (t, *J* = 7.6 Hz, 3H); **^{13}C NMR** (100 MHz, $CDCl_3$): δ 175.2 (d, *J* = 11.2 Hz), 138.5 (d, *J* = 2.2 Hz), 135.0, 134.6, 130.8, 128.8, 127.5 (d, *J* = 4.2 Hz),

64.3 (d, *J* = 10.5 Hz), 27.2 (d, *J* = 5.6 Hz), 25.7, 20.8, 15.7; ^{19}**F NMR** (376 MHz, $CDCl_3$): δ -63.49.
HRMS (ESI) m/z calcd. for $C_{14}H_{21}FNO$ $[M+H]^+$ 238.1602, found 238.1598.

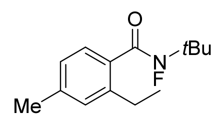

1g

***N*-(*tert*-butyl)-2-ethyl-*N*-fluoro-4-methylbenzamide (1g)**
610 mg (39% yield). ^{1}**H NMR** (400 MHz, $CDCl_3$): δ 7.22 (d, *J* = 7.8 Hz, 1H), 7.06 (s, 1H), 7.04-6.98 (m, 1H), 2.71 (q, *J* = 7.6 Hz, 2H), 2.34 (s, 3H), 1.54 (d, *J* = 2.0 Hz, 9H), 1.23 (t, *J* = 7.6 Hz, 3H); ^{13}**C NMR** (100 MHz, $CDCl_3$): δ 175.4 (d, *J* = 10.5 Hz), 141.9 (d, *J* = 1.9 Hz), 140.2, 131.7, 129.8, 127.5 (d, *J* = 4.7 Hz), 126.1, 64.2 (d, *J* = 10.5 Hz), 27.2 (d, *J* = 5.6 Hz), 26.1, 21.4, 15.8; ^{19}**F NMR** (376 MHz, $CDCl_3$): δ -62.95.
HRMS (ESI) m/z calcd. for $C_{14}H_{21}FNO$ $[M+H]^+$ 238.1602, found 238.1598.

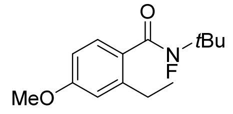

1h

***N*-(*tert*-butyl)-2-ethyl-*N*-fluoro-4-methoxybenzamide (1h)**
Yellow oil, 520 mg (43% yield). ^{1}**H NMR** (400 MHz, $CDCl_3$): δ 7.32 (d, *J* = 8.5 Hz, 1H), 6.81-6.66 (m, 2H), 3.82 (s, 3H), 2.75 (q, *J* = 7.6 Hz, 2H), 1.53 (d, *J* = 1.9 Hz, 9H), 1.24 (t, *J* = 7.7 Hz, 3H); ^{13}**C NMR** (100 MHz, $CDCl_3$): δ 175.3 (d, *J* = 10.5 Hz), 161.0, 144.7 (d, *J* = 1.9 Hz), 129.8 (d, *J* = 5.0 Hz), 126.9, 114.7, 110.4, 64.11 (d, *J* = 10.6 Hz), 55.2, 27.1 (d, *J* = 5.7 Hz), 26.4, 15.7; ^{19}**F NMR** (376 MHz, $CDCl_3$): δ -62.16.
HRMS (ESI) m/z calcd. for $C_{14}H_{21}FNO_2$ $[M+H]^+$ 254.1551, found 254.1547.

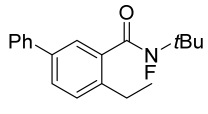

1i

***N*-(*tert*-butyl)-4-ethyl-*N*-fluoro-[1,1'-biphenyl]-3-carboxamide(1i)**
Yellow oil, 330 mg (46% yield). ^{1}**H NMR** (400 MHz, $CDCl_3$): δ 7.59-7.55 (m, 3H), 7.54-7.50 (m, 1H), 7.45-7.40 (m, 2H), 7.36-7.30 (m, 2H), 2.77 (q, *J* = 7.6 Hz, 2H), 1.57 (d, *J* = 1.9 Hz, 9H), 1.27 (t, *J* = 7.6 Hz, 3H); ^{13}**C NMR** (100 MHz, $CDCl_3$): δ 174.9 (d, *J* = 10.9 Hz), 140.6 (d, *J* = 2.1 Hz), 140.3, 138.5, 135.1, 129.4, 128.8, 128.7, 127.4, 127.1, 125.8 (d, *J* = 4.3 Hz), 64.4 (d, *J* = 10.5 Hz), 27.2 (d, *J* = 5.5 Hz), 25.8, 15.6; ^{19}**F NMR** (376 MHz, $CDCl_3$) δ -63.19.
HRMS (ESI) m/z calcd. for $C_{19}H_{23}FNO$ $[M+H]^+$ 300.1758, found 300.1755.

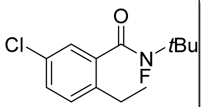

1j

***N*-(*tert*-butyl)-5-chloro-2-ethyl-*N*-fluorobenzamide (1j)**
Yellow oil, 650 mg (48% yield). ^{1}**H NMR** (400 MHz, $CDCl_3$): δ 7.27-7.23 (m, 2H), 7.18 (dd, *J* = 8.2, 2.0 Hz, 1H), 2.71 (q, *J* = 7.6 Hz, 2H), 1.54 (d, *J* = 2.0 Hz, 9H), 1.23 (t, *J* = 7.6 Hz, 3H); ^{13}**C NMR** (100 MHz, $CDCl_3$): δ 174.0 (d, *J* = 10.9 Hz), 143.8 (d, *J* = 2.2 Hz), 135.9 (d, *J* = 1.7 Hz), 133.0, 129.0, 128.7 (d, *J* = 4.6 Hz), 125.6, 64.4 (d, *J* = 10.5 Hz), 27.1 (d, *J* = 5.6 Hz), 26.0, 15.3; ^{19}**F NMR** (376 MHz, $CDCl_3$) δ -63.23.
HRMS (ESI) m/z calcd. for $C_{13}H_{18}{}^{34.9689}ClFNO$ $[M+H]^+$ 258.1055, found 258.1052; calcd. for $C_{13}H_{18}{}^{36.9659}ClFNO$ $[M+H]^+$ 260.1026, found 260.1023.

N-(*tert*-butyl)-3-ethyl-*N*-fluorothiophene-2-carboxamide (1k)

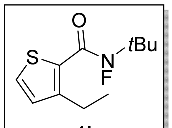

1k

Yellow oil, 670 mg (44% yield). **^{1}H NMR** (400 MHz, $CDCl_3$): δ 7.43 (d, *J* = 5.1 Hz, 1H), 6.97 (dd, *J* = 5.1, 1.7 Hz, 1H), 2.93 (q, *J* = 7.5 Hz, 2H), 1.51 (d, *J* = 2.0 Hz, 9H), 1.25 (t, *J* = 7.5 Hz, 3H); **^{13}C NMR** (100 MHz, $CDCl_3$): δ 169.2 (d, *J* = 7.0 Hz), 152.2, 129.9 (d, *J* = 6.3 Hz), 129.2, 127.5 (d, *J* = 2.6 Hz), 64.5 (d, *J* = 10.7 Hz), 26.9 (d, *J* = 6.2 Hz), 23.4, 14.8; **^{19}F NMR** (376 MHz, $CDCl_3$): δ -64.22.
HRMS (ESI) m/z calcd. for $C_{11}H_{17}FNOS$ $[M+H]^+$ 230.1009, found 230.1007.

2-ethyl-*N*-fluoro-*N*-(2,4,4-trimethylpentan-2-yl)benzamide (1l)

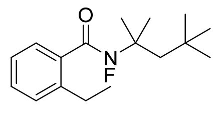

1l

Yellow oil, 690 mg (31% yield). **^{1}H NMR** (400 MHz, $CDCl_3$): δ 7.38-7.30 (m, 1H), 7.26 (dd, *J* = 7.2, 1.6 Hz, 2H), 7.20 (td, *J* = 7.4, 1.2 Hz, 1H), 2.73 (q, *J* = 7.6 Hz, 2H), 1.90 (d, *J* = 2.0 Hz, 2H), 1.62 (d, *J* = 2.2 Hz, 6H), 1.24 (t, *J* = 7.6 Hz, 3H), 1.08 (s, 9H); **^{13}C NMR** (100 MHz, $CDCl_3$): δ 173.8 (d, *J* = 11.4 Hz),141.5 (d, *J* = 2.2 Hz), 134.9, 129.9 (d, *J* = 1.5 Hz), 128.9, 126.8 (d, *J* = 4.5 Hz), 125.4, 68.1 (d, *J* = 9.6 Hz), 51.3 (d, *J* = 3.6 Hz), 31.6, 31.3, 27.6 (d, *J* = 6.0 Hz), 26.0, 15.7; **^{19}F NMR** (376 MHz, $CDCl_3$): δ -61.33.
HRMS (ESI) m/z calcd. for $C_{17}H_{27}FNO$ $[M+H]^+$ 280.2071, found 280.2069.

N-(1-(2-ethylphenyl)-2-methylpropan-2-yl)-*N*-fluorobenzamide (1m)

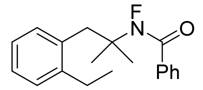

1m

Yellow oil, 430 mg (28% yield). **^{1}H NMR** (400 MHz, $CDCl_3$): δ 7.73-7.67 (m, 2H), 7.52-7.47 (m, 1H), 7.41 (dd, *J* = 8.2, 6.8 Hz, 2H), 7.25-7.18 (m, 3H), 7.15-7.09 (m, 1H), 3.28 (s, 2H), 2.76 (q, *J* = 7.5 Hz, 2H), 1.51 (d, *J* = 2.3 Hz, 6H), 1.20 (t, *J* = 7.5 Hz, 3H); **^{13}C NMR** (100 MHz, $CDCl_3$): δ 174.1 (d, *J* = 7.8 Hz), 143.5, 134.6, 134.3, 131.8, 131.6, 128.9 (d, *J* = 6.0 Hz), 128.7, 128.1, 127.0, 125.4, 68.1 (d, *J* = 9.4 Hz), 38.9 (d, *J* = 3.7 Hz), 26.0, 25.2 (d, *J* = 6.5 Hz), 16.0; **^{19}F NMR** (376 MHz, $CDCl_3$): δ -63.82.
HRMS (ESI) m/z calcd. for $C_{19}H_{23}FNO$ $[M+H]^+$ 300.1758, found 300.1755.

N-fluoro-*N*-(2-methyl-5-phenylpentan-2-yl)benzamide (1n)

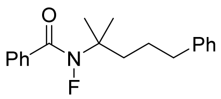

1n

Yellow oil, 910 mg (39% yield). **^{1}H NMR** (400 MHz, $CDCl_3$): δ 7.70-7.65 (m, 2H), 7.53 – 7.45 (m, 1H), 7.42 – 7.37 (m, 2H), 7.30-7.25 (m, 2H), 7.22-7.15 (m, 3H), 2.64 (t, *J* = 7.6 Hz, 2H), 1.94-1.87 (m, 2H), 1.82-1.73 (m, 2H), 1.49 (d, *J* = 1.9 Hz, 6H); **^{13}C NMR** (100 MHz, $CDCl_3$): δ 174.3 (d, *J* = 7.7 Hz), 142.3, 134.2 (d, *J* = 1.3 Hz), 131.7, 129.7, 128.8 (d, *J* = 6.1 Hz), 128.4 (d, *J* = 4.0 Hz), 128.0, 125.8, 66.7 (d, *J* = 9.7 Hz), 39.8 (d, *J* = 4.8 Hz), 36.2, 26.1, 25.1 (d, *J* = 6.2 Hz); **^{19}F NMR** (376 MHz, $CDCl_3$) δ -66.11.
HRMS (ESI) m/z calcd. for $C_{19}H_{23}FNO$ $[M+H]^+$ 300.1758, found 300.1755.

N-fluoro-*N*-(2-methyl-7-phenylhept-6-yn-2-yl) benzamide (1o)

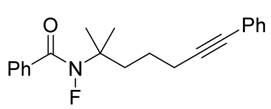

1o

Yellow oil, 310 mg (23% yield). **^{1}H NMR** (400 MHz, $CDCl_3$): δ 7.73-7.67 (m, 2H), 7.55-7.47 (m, 1H), 7.44-7.37 (m, 4H), 7.30-7.24 (m, 3H), 2.45 (t, *J* = 7.1 Hz, 2H), 2.07-1.99 (m, 2H), 1.84-1.72 (m, 2H), 1.55 (d, *J* = 1.9 Hz, 6H); **^{13}C NMR** (100 MHz, $CDCl_3$): δ 174.3 (d, *J* = 7.6 Hz), 134.1, 131.8, 131.6, 128.9 (d, *J* = 6.2 Hz), 128.2, 128.0, 127.6, 123.9, 89.8, 81.0, 66.6 (d, *J* = 9.9 Hz), 39.5 (d, *J* = 5.0 Hz), 25.1 (d, *J* = 6.1 Hz), 23.7, 19.7; **^{19}F NMR** (376 MHz, $CDCl_3$): δ -66.08.
HRMS (ESI) m/z calcd. for $C_{21}H_{23}FNO$ $[M+H]^+$ 324.1758, found 324.1756.

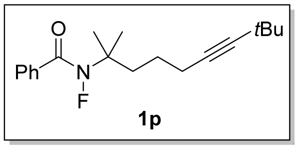

N-fluoro-N-(2,8,8-trimethylnon-6-yn-2-yl)benzamide (1p)

Yellow oil, 410 mg (19% yield). **^{1}H NMR** (400 MHz, $CDCl_3$): δ 7.73-7.65 (m, 2H), 7.53-7.46 (m, 1H), 7.45-7.37 (m, 2H), 2.17 (t, *J* = 7.1 Hz, 2H), 1.98-1.89 (m, 2H), 1.67-1.60 (m, 2H), 1.52 (d, *J* = 1.9 Hz, 6H), 1.20 (s, 9H); **^{13}C NMR** (100 MHz, $CDCl_3$): δ 174.3 (d, *J* = 7.7 Hz), 134.2, 131.7, 128.9 (d, *J* = 6.1 Hz), 128.0, 89.5, 78.0, 66.6 (d, *J* = 9.8 Hz), 39.3 (d, *J* = 5.0 Hz), 31.4, 27.3, 25.1 (d, *J* = 6.0 Hz), 24.1, 19.0; **^{19}F NMR** (376 MHz, $CDCl_3$): δ -66.15.
HRMS (ESI) m/z calcd. for $C_{19}H_{26}FNO$ $[M+H]^+$ 304.2071, found 304.2069.

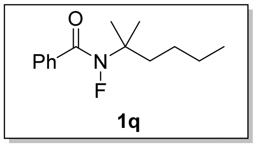

***N*-fluoro-*N*-(2-methylhexan-2-yl)benzamide (1q)**

Yellow oil, 870 mg (46% yield). **^{1}H NMR** (400 MHz, $CDCl_3$): δ 7.72-7.66 (m, 2H), 7.53-7.44 (m, 1H), 7.43-7.36 (m, 2H), 1.92-1.80 (m, 2H), 1.51 (d, *J* = 2.1 Hz, 6H), 1.47-1.27 (m, 4H), 0.92 (t, *J* = 7.2 Hz, 3H); **^{13}C NMR** (100 MHz, $CDCl_3$): δ 174.1 (d, *J* = 7.8 Hz), 134.3 (d, *J* = 1.4 Hz), 131.6, 128.8 (d, *J* = 6.2 Hz), 128.0, 66.8 (d, *J* = 9.6 Hz), 39.7 (d, *J* = 4.9 Hz), 26.3, 25.1 (d, *J* = 6.1 Hz), 23.1, 14.1; **^{19}F NMR** (376 MHz, $CDCl_3$): δ -66.25.
HRMS (ESI) m/z calcd. for $C_{14}H_{21}FNO$ $[M+H]^+$ 238.1602, found 238.1598.

General procedure for enantioselective Sonogashira-type oxidative cross-coupling of unactivated C(sp^3)–H bonds with terminal alkynes

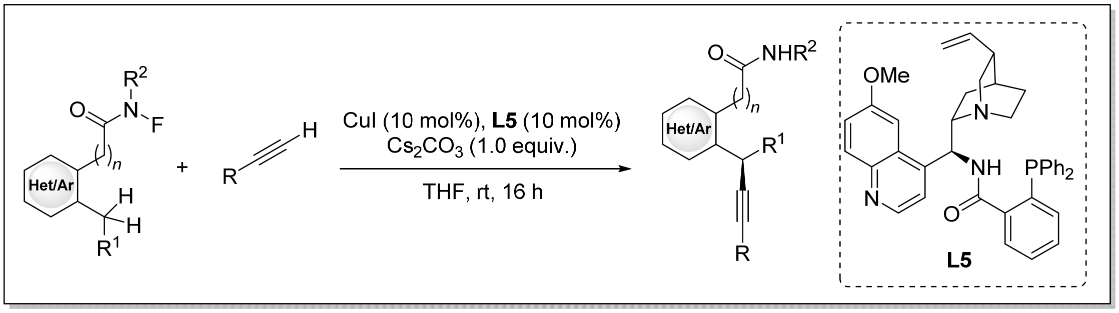

Supplementary Figure 241 General procedure for enantioselective reaction

General procedure A:

Under argon atmosphere, an oven-dried resealable Schlenk tube equipped with a magnetic stir bar was charged with CuI (3.8 mg, 0.020 mmol, 10 mol%), **L5** (12.2 mg, 0.020mmol, 10 mol%), Cs_2CO_3 (65.2 mg, 0.20 mmol, 1.0 equiv.), and anhydrous THF (2.4 mL). Then, *N*-fluorocarocarboxamide (0.20 mmol, 1.0 equiv.) and alkyne (0.40 mmol, 2.0 equiv.) were sequentially added into the mixture and the reaction mixture was stirred at rt for 16 h. Upon completion (monitored by TLC), the precipitate was filtered off and washed by DCM. The filtrate was evaporated and the residue was purified by column chromatography on silica gel to afford the desired product.

General procedure B:

Under argon atmosphere, an oven-dried resealable Schlenk tube equipped with a magnetic stir bar was charged with CuTc (5.7 mg, 0.030 mmol, 15 mol%), **L5** (12.2 mg, 0.020mmol, 10 mol%), Cs_2CO_3 (130.4 mg, 0.40 mmol, 2.0 equiv.), and anhydrous THF (2.4 mL). Then, *N*-fluorocarocarboxamide (0.20 mmol, 1.0 equiv.) and alkyne (0.40 mmol, 2.0 equiv.) were sequentially added into the mixture and the reaction mixture was stirred at rt for 24 h. Upon completion (monitored by TLC), the precipitate was filtered off and washed by DCM. The filtrate was evaporated and the residue was purified by column chromatography on silica gel to afford the desired product.

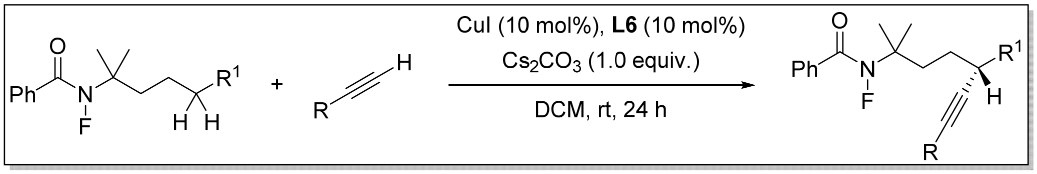

Supplementary Figure 242 General procedure for enantioselective reaction

General procedure C:

Under argon atmosphere, an oven-dried resealable Schlenk tube equipped with a magnetic stir bar was charged with CuI (3.8 mg, 0.020 mmol, 10 mol%), **L6** (16.7 mg, 0.020mmol, 10 mol%), Cs_2CO_3 (65.2 mg, 0.20 mmol, 1.0 equiv.), and anhydrous DCM (2.4 mL). Then, *N*-

fluorocarocarboxamide (0.20 mmol, 1.0 equiv.) and alkyne (0.40 mmol, 2.0 equiv.) were sequentially added into the mixture and the reaction mixture was stirred at rt for 24 h. Upon completion (monitored by TLC), the precipitate was filtered off and washed by DCM. The filtrate was evaporated and the residue was purified by column chromatography on silica gel to afford the desired product.

General procedure D:

Under argon atmosphere, an oven-dried resealable Schlenk tube equipped with a magnetic stir bar was charged with CuI (3.8 mg, 0.020 mmol, 10 mol%), **L7** (13.9 mg, 0.020mmol, 10 mol%), Cs_2CO_3 (65.2 mg, 0.20 mmol, 1.0 equiv.), and anhydrous chloroform (2.4 mL). Then, *N*-fluorocarocarboxamide (0.20 mmol, 1.0 equiv.) and alkyne (0.40 mmol, 2.0 equiv.) were sequentially added into the mixture and the reaction mixture was stirred at rt for 24 h. Upon completion (monitored by TLC), the precipitate was filtered off and washed by DCM. The filtrate was evaporated and the residue was purified by column chromatography on silica gel to afford the desired product.

Since the reaction is sensitive to water and air, Schlenk tubes and the reagents must be dried prior to use.

General procedure for preparation of the racemates:

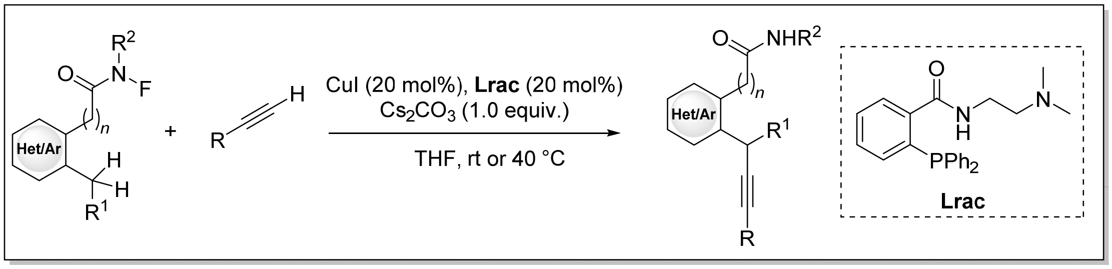

Supplementary Figure 243 General procedure for racemic reaction

The racemates of products were prepared following the same procedure described above using CuI (0.020 mmol, 20 mol%) and **Lrac** (0.020 mmol, 20 mol%) as catalyst and ligand, respectively, at rt or 40 °C in anhydrous THF (1.2 mL) for 16 to 24 h. Upon completion (monitored by TLC), the precipitate was filtered off and washed with DCM. The filtrate was concentrated, and the residue was purified by column chromatography on silica gel to afford the desired product.

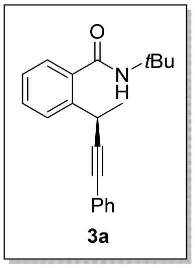

(*S*)-*N*-(*tert*-butyl)-2-(4-phenylbut-3-yn-2-yl)benzamide (3a)

According to general procedure A: Colorless oil, 50.8 mg (83%, 94% ee).

HPLC analysis: Chiralcel AD-H (*n*-hexane/*i*-PrOH = 90/10, flow rate 0.8 mL/min, λ = 254 nm), t_R (minor) = 5.96 min, t_R (major) = 8.93 min.

^{1}H NMR (400 MHz, $CDCl_3$): δ 7.72 (dd, *J* = 7.9, 1.2 Hz, 1H), 7.51-7.38 (m, 3H), 7.33 (dd, *J* = 7.6, 1.5 Hz, 1H), 7.30-7.21 (m, 4H), 5.76 (s, 1H), 4.48 (q, *J* = 7.0 Hz, 1H), 1.61 (d, *J* = 7.0 Hz, 3H), 1.47 (s, 9H); **^{13}C NMR** (100 MHz,

$CDCl_3$): δ 169.3, 141.1, 136.6, 131.6, 130.1, 128.2, 127.9, 127.8, 126.8, 126.7, 123.6, 93.1, 82.1, 52.0, 29.0, 28.8, 24.1.
HRMS (ESI) m/z calcd. for $C_{21}H_{24}NO$ $[M+H]^+$ 306.1852, found 306.1849.

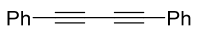

3a'

1,4-diphenylbuta-1,3-diyne (3a')
3a' matched the reported spectra.[10]
^{1}H NMR (400 MHz, $CDCl_3$): δ 7.57-7.49 (m, 2H), 7.39-7.29 (m, 3H); **^{13}C NMR** (100 MHz, $CDCl_3$): δ 132.5, 129.3, 128.5, 121.8, 81.6, 74.0.

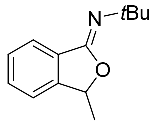

3a''

(*Z*)-*N*-(*tert*-butyl)-3-methylisobenzofuran-1(3*H*)-imine (3a")
Colorless oil, **^{1}H NMR** (400 MHz, $CDCl_3$): δ 7.64-7.40 (m, 3H), 7.35-7.28 (m, 1H), 5.68 (q, *J* = 6.6 Hz, 1H), 1.62 (d, *J* = 6.6 Hz, 3H), 1.47 (s, 9H); **^{13}C NMR** (100 MHz, $CDCl_3$): δ 147.3, 132.2, 129.0, 128.3, 125.1, 120.7, 81.8, 54.7, 29.5, 20.9.
HRMS (ESI) m/z calcd. for $C_{13}H_{18}NO$ $[M+H]^+$ 204.1383, found 204.1380.

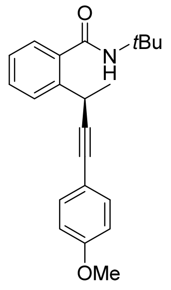

3b

(*S*)-*N*-(*tert*-butyl)-2-(4-(4-methoxyphenyl)but-3-yn-2-yl)benzamide (3b)
According to general procedure A: Colorless oil, 47.3 mg (71%, 93% ee).
HPLC analysis: Chiralcel AD-H (*n*-hexane/*i*-PrOH = 90/10, flow rate 0.8 mL/min, λ = 254 nm), t_R (minor) = 7.30 min, t_R (major) = 15.69 min.
^{1}H NMR (400 MHz, $CDCl_3$): δ 7.71 (dd, *J* = 8.0, 1.2 Hz, 1H), 7.41 (td, *J* = 7.6, 1.6 Hz, 1H), 7.36-7.30 (m, 3H), 7.27-7.21 (m, 1H), 6.83-6.77 (m, 2H), 5.78 (s, 1H), 4.45 (q, *J* = 7.0 Hz, 1H), 3.79 (s, 3H), 1.60 (d, *J* = 7.0 Hz, 3H), 1.47 (s, 9H); **^{13}C NMR** (100 MHz, $CDCl_3$): δ 169.3, 159.2, 141.2, 136.6, 133.0, 130.1, 127.9, 126.9, 126.7, 115.8, 113.8, 91.6, 81.8, 55.3, 52.0, 29.0, 28.8, 24.1.
HRMS (ESI) m/z calcd. for $C_{22}H_{26}NO_2$ $[M+H]^+$ 336.1958, found 336.1953.

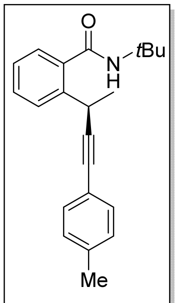

3c

(*S*)-*N*-(*tert*-butyl)-2-(4-(*p*-tolyl)but-3-yn-2-yl)benzamide (3c)
According to general procedure A: Colorless oil, 46.2 mg (73%, 94% ee).
HPLC analysis: Chiralcel AD-H (*n*-hexane/*i*-PrOH = 90/10, flow rate 0.8 mL/min, λ = 254 nm), t_R (minor) = 5.69 min, t_R (major) = 10.25 min.
^{1}H NMR (400 MHz, $CDCl_3$): δ 7.71 (dd, *J* = 8.0, 1.2 Hz, 1H), 7.40 (td, *J* = 7.6, 1.6 Hz, 1H), 7.35-7.27 (m, 3H), 7.24 (td, *J* = 8.0, 1.6 Hz, 1H), 7.12 -7.05 (m, 2H), 5.78 (s, 1H), 4.46 (d, *J* = 7.0 Hz, 1H), 2.32 (s, 3H), 1.60 (d, *J* = 7.0 Hz, 3H), 1.46 (s, 9H); **^{13}C NMR** (100 MHz, $CDCl_3$): δ 169.3, 141.2, 137.8, 136.6, 131.5, 130.1, 129.0, 127.9, 126.9, 126.7, 120.5, 92.4, 82.1, 52.0, 29.0, 28.8, 24.1, 21.4.
HRMS (ESI) m/z calcd. for $C_{22}H_{26}NO$ $[M+H]^+$ 320.2009, found 320.2006.

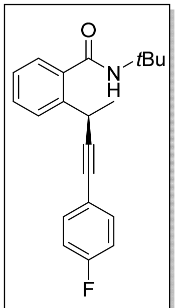

3d

(*S*)-*N*-(*tert*-butyl)-2-(4-(4-fluorophenyl)but-3-yn-2-yl)benzamide (3d)
According to general procedure A: Colorless oil, 50.1 mg (78%, 93% ee).

HPLC analysis: Chiralcel AD-H (*n*-hexane/*i*-PrOH = 90/10, flow rate 0.8 mL/min, λ = 254 nm), t_R (minor) = 6.45 min, t_R (major) = 12.19 min.
^{1}H NMR (400 MHz, $CDCl_3$): δ 7.70 (dd, *J* = 8.0, 1.2 Hz, 1H), 7.45-7.36 (m, 3H), 7.33 (dd, *J* = 7.6, 1.6 Hz, 1H), 7.28-7.22 (m, 1H), 7.00-6.94 (m, 2H), 5.75 (s, 1H), 4.48 (q, *J* = 7.0 Hz, 1H), 1.60 (d, *J* = 7.1 Hz, 3H), 1.47 (s, 9H); **^{13}C NMR** (100 MHz, $CDCl_3$): δ 169.2, 162.2 (d, *J* = 248.6 Hz), 141.1, 136.5, 133.4 (d, *J* = 8.2 Hz), 130.1, 127.9, 126.83, 126.75, 119.7 (d, *J* = 3.5 Hz), 115.4 (d, *J* = 22.0 Hz), 92.8, 80.9, 52.0, 28.9, 28.8, 24.1; **^{19}F NMR** (376 MHz, $CDCl_3$): δ -111.84.
HRMS (ESI) m/z calcd. for $C_{21}H_{23}FNO$ $[M+H]^+$ 324.1758, found 324.1755.

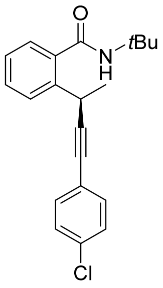
3e

(*S*)-*N*-(*tert*-butyl)-2-(4-(4-chlorophenyl)but-3-yn-2-yl)benzamide (3e)
According to general procedure A: Colorless oil, 56.4 mg (83%, 92% ee).
HPLC analysis: Chiralcel AD-H (*n*-hexane/*i*-PrOH = 90/10, flow rate 0.8 mL/min, λ = 254 nm), t_R (minor) = 6.46 min, t_R (major) = 14.36 min.
^{1}H NMR (400 MHz, $CDCl_3$): δ 7.69 (dd, *J* = 7.9, 1.2 Hz, 1H), 7.41 (td, *J* = 7.6, 1.5 Hz, 1H), 7.36 -7.30 (m, 3H), 7.28 -7.23 (m, 3H), 5.75 (s, 1H), 4.50 (q, *J* = 7.0 Hz, 1H), 1.60 (d, *J* = 7.0 Hz, 3H), 1.46 (s, 9H); ^{13}C NMR (100 MHz, $CDCl_3$): δ 169.2, 140.9, 136.5, 133.7, 132.8, 130.2, 128.5, 127.9, 126.84, 126.78, 122.1, 94.2, 80.9, 52.0, 29.0, 28.8, 24.1.
HRMS (ESI) m/z calcd. for $C_{21}H_{23}{}^{34.9689}ClNO$ $[M+H]^+$ 340.1463, found 340.1460; calcd. for $C_{21}H_{23}{}^{36.9659}ClNO$ $[M+H]^+$ 342.1433, found 342.1430.

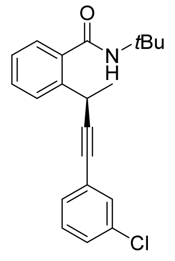
3f

(*S*)-*N*-(*tert*-butyl)-2-(4-(3-chlorophenyl)but-3-yn-2-yl)benzamide (3f)
According to general procedure A: Colorless oil, 51.6 mg (76%, 92% ee).
HPLC analysis: Chiralcel AD-H (*n*-hexane/*i*-PrOH = 98/2, flow rate 0.8 mL/min, λ = 254 nm), t_R (minor) = 10.69 min, t_R (major) = 15.99 min.
^{1}H NMR (400 MHz, $CDCl_3$): δ 7.69 (dd, *J* = 8.0, 1.2 Hz, 1H), 7.46-7.38 (m, 2H), 7.33 (dd, *J* = 7.6, 1.6 Hz, 1H), 7.31-7.18 (m, 4H), 5.74 (s, 1H), 4.51 (q, *J* = 7.0 Hz, 1H), 1.60 (d, *J* = 7.0 Hz, 3H), 1.47 (s, 9H); **^{13}C NMR** (100 MHz, $CDCl_3$): δ 169.2, 140.9, 136.5, 134.0, 131.5, 130.2, 129.7, 129.5, 128.1, 127.9, 126.82, 126.81, 125.4, 94.6, 80.7, 52.0, 28.9, 28.8, 24.0.
HRMS (ESI) m/z calcd. for $C_{21}H_{23}{}^{34.9689}ClNO$ $[M+H]^+$ 340.1463, found 340.1459; calcd. for $C_{21}H_{23}{}^{36.9659}ClNO$ $[M+H]^+$ 342.1433, found 342.1430.

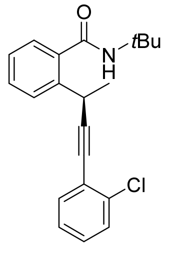
3g

(*S*)-*N*-(*tert*-butyl)-2-(4-(2-chlorophenyl)but-3-yn-2-yl)benzamide (3g)
According to general procedure A: Colorless oil, 66.5 mg (98%, 93% ee).
HPLC analysis: Chiralcel AD-H (*n*-hexane/*i*-PrOH = 95/5, flow rate 0.8 mL/min, λ = 254 nm), t_R (minor) = 7.07 min, t_R (major) = 8.73 min.
^{1}H NMR (400 MHz, $CDCl_3$): δ 7.80 (dd, *J* = 8.0, 1.2 Hz, 1H), 7.46-7.39 (m, 2H), 7.38-7.30 (m, 2H), 7.25-7.14 (m, 3H), 5.76 (s, 1H), 4.56 (q, *J* = 7.0 Hz,

1H), 1.64 (d, J = 7.0 Hz, 3H), 1.47 (s, 9H); **^{13}C NMR** (100 MHz, $CDCl_3$): δ 169.3, 140.9, 136.5, 136.0, 133.2, 130.1, 129.2, 128.8, 128.1, 126.8, 126.4, 123.5, 98.7, 79.1, 52.0, 29.3, 28.8, 24.2.
HRMS (ESI) m/z calcd. for $C_{21}H_{23}{}^{34.9689}ClNO$ $[M+H]^+$ 340.1463, found 340.1460; calcd. for $C_{21}H_{23}{}^{36.9659}ClNO$ $[M+H]^+$ 342.1433, found 342.1431.

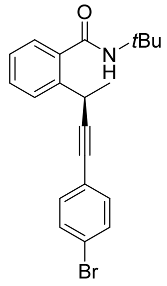
3h

(*S*)-2-(4-(4-bromophenyl)but-3-yn-2-yl)-*N*-(*tert*-butyl)benzamide (3h)
According to general procedure A: White powder, 64.7 mg (84%, 92% ee).
HPLC analysis: Chiralcel AD-H (*n*-hexane/*i*-PrOH = 90/10, flow rate 0.8 mL/min, λ = 254 nm), t_R (minor) = 6.60 min, t_R (major) = 15.87 min.
^{1}H NMR (400 MHz, $CDCl_3$): δ 7.69 (dd, J = 7.9, 1.2 Hz, 1H), 7.44 -7.39 (m, 3H), 7.33 (dd, J = 7.6, 1.5 Hz, 1H), 7.29 -7.23 (m, 3H), 5.74 (s, 1H), 4.49 (q, J = 7.0 Hz, 1H), 1.60 (d, J = 7.0 Hz, 3H), 1.46 (s, 9H); **^{13}C NMR** (100 MHz, $CDCl_3$): δ 169.2, 140.9, 136.5, 133.1, 131.5, 130.2, 127.9, 126.83, 126.79, 122.6, 121.9, 94.5, 81.0, 52.0, 29.0, 28.8, 24.0.
HRMS (ESI) m/z calcd. for $C_{21}H_{23}{}^{78.9183}BrNO$ $[M+H]^+$ 384.0958, found 384.0954; calcd. for $C_{21}H_{23}{}^{80.9163}BrNO$ $[M+H]^+$ 386.0937, found 386.0932.

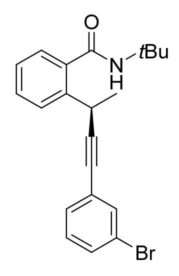
3i

(*S*)-2-(4-(3-bromophenyl)but-3-yn-2-yl)-*N*-(*tert*-butyl)benzamide (3i)
According to general procedure A: White powder, 63.3 mg (83%, 92% ee).
HPLC analysis: Chiralcel AD-H (*n*-hexane/*i*-PrOH = 90/10, flow rate 0.8 mL/min, λ = 254 nm), t_R (minor) = 5.81 min, t_R (major) = 6.80 min.
^{1}H NMR (400 MHz, $CDCl_3$): δ 7.69 (dd, J = 7.8, 1.2 Hz, 1H), 7.56 (t, J = 1.8 Hz, 1H), 7.45-7.38 (m, 2H), 7.35-7.30 (m, 2H), 7.28-7.23 (m, 1H), 7.15 (t, J = 7.9 Hz, 1H), 5.74 (s, 1H), 4.51 (q, J = 7.0 Hz, 1H), 1.60 (d, J = 7.1 Hz, 3H), 1.47 (s, 9H); **^{13}C NMR** (100 MHz, $CDCl_3$): δ 169.2, 140.9, 136.5, 134.4, 131.0, 130.19, 130.15, 129.7, 127.9, 126.8, 125.7, 122.0, 94.7, 80.6, 52.0, 28.9, 28.8, 24.0.
HRMS (ESI) m/z calcd. for $C_{21}H_{23}{}^{78.9183}BrNO$ $[M+H]^+$ 384.0958, found 384.0954; calcd. for $C_{21}H_{23}{}^{80.9163}BrNO$ $[M+H]^+$ 386.0937, found 386.0932.

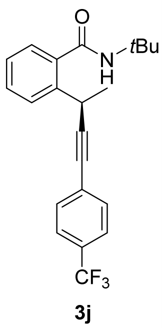
3j

(*S*)-*N*-(*tert*-butyl)-2-(4-(4-(trifluoromethyl)phenyl)but-3-yn-2-yl)benzamide (3j)
According to general procedure A: Colorless oil, 56.8 mg (76%, 92% ee).
HPLC analysis: Chiralcel AD-H (*n*-hexane/*i*-PrOH = 90/10, flow rate 0.8 mL/min, λ = 254 nm), t_R (minor) = 5.92 min, t_R (major) = 15.01 min.
^{1}H NMR (400 MHz, $CDCl_3$): δ 7.70 (d, J = 8.0 Hz, 1H), 7.57-7.48 (m, 4H), 7.43 (td, J = 7.6, 1.6 Hz, 1H), 7.34 (dd, J = 7.6, 1.6 Hz, 1H), 7.29-7.23 (m, 1H), 5.73 (s, 1H), 4.55 (q, J = 7.0 Hz, 1H), 1.62 (d, J = 7.1 Hz, 3H), 1.48 (s, 9H); **^{13}C NMR** (100 MHz, $CDCl_3$): δ 169.2, 140.8, 136.5, 131.8, 130.2, 129.0 (q, J = 32.5 Hz), 127.9, 127.5, 126.9, 126.8, 125.1 (q, J = 3.8 Hz), 124.0 (q, J = 271.0 Hz), 95.9, 80.8, 52.0, 28.9, 28.8, 24.0; **^{19}F NMR** (376 MHz, $CDCl_3$): δ -62.73.

HRMS (ESI) m/z calcd. for $C_{22}H_{23}F_3NO$ $[M+H]^+$ 374.1726, found 374.1722.

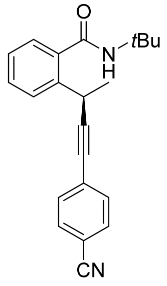

3k

(*S*)-*N*-(*tert*-butyl)-2-(4-(4-cyanophenyl)but-3-yn-2-yl)benzamide (3k)

According to general procedure A: Colorless oil, 45.9 mg (70%, 89% ee).

HPLC analysis: Chiralcel AD-H (*n*-hexane/*i*-PrOH = 90/10, flow rate 0.8 mL/min, λ = 254 nm), t_R (minor) = 13.07 min, t_R (major) = 23.37 min.

^{1}H NMR (400 MHz, $CDCl_3$): δ 7.68 (dd, *J* = 7.8, 1.3 Hz, 1H), 7.59-7.54 (m, 2H), 7.51-7.46 (m, 2H), 7.43 (td, *J* = 7.6, 1.6 Hz, 1H), 7.34 (dd, *J* = 7.6, 1.5 Hz, 1H), 7.30-7.23 (m, 1H), 5.76 (s, 1H), 4.59 (q, *J* = 7.0 Hz, 1H), 1.61 (d, *J* = 7.1 Hz, 3H), 1.47 (s, 9H); **^{13}C NMR** (100 MHz, $CDCl_3$): δ 169.1, 140.6, 136.4, 132.2, 131.9, 130.2, 128.7, 127.9, 126.92, 126.86, 118.6, 111.1, 98.2, 80.6, 52.0, 29.0, 28.8, 24.0.

HRMS (ESI) m/z calcd. for $C_{22}H_{23}N_2O$ $[M+H]^+$ 331.1805, found 331.1802.

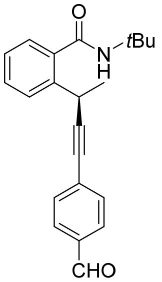

3l

(*S*)-*N*-(*tert*-butyl)-2-(4-(4-formylphenyl)but-3-yn-2-yl)benzamide (3l)

According to general procedure A: White powder, 40.8 mg (62%, 91% ee).

HPLC analysis: Chiralcel AD-H (*n*-hexane/*i*-PrOH = 90/10, flow rate 0.8 mL/min, λ = 254 nm), t_R (minor) = 11.70 min, t_R (major) = 21.85 min.

^{1}H NMR (400 MHz, $CDCl_3$): δ 10.0 (s, 1H), 7.80 (d, *J* = 8.0 Hz, 2H), 7.71 (dd, *J* = 8.0, 1.2 Hz, 1H), 7.55 (d, *J* = 8.0 Hz, 2H), 7.43 (td, *J* = 7.6, 1.6 Hz, 1H), 7.35 (dd, *J* = 7.6, 1.6 Hz, 1H), 7.27 (t, *J* = 6.4 Hz, 1H), 5.77 (s, 1H), 4.58 (q, *J* = 7.0 Hz, 1H), 1.62 (d, *J* = 7.0 Hz, 3H), 1.48 (s, 9H); **^{13}C NMR** (100 MHz, $CDCl_3$): δ 191.5, 169.2, 140.7, 136.4, 135.1, 132.2, 130.2, 130.1, 129.5, 127.9, 126.88, 126.85, 97.8, 81.3, 52.0, 29.1, 28.8, 24.0.

HRMS (ESI) m/z calcd. for $C_{22}H_{24}NO_2$ $[M+H]^+$ 334.1802, found 334.1798.

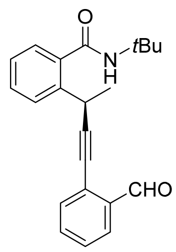

3m

(*S*)-*N*-(*tert*-butyl)-2-(4-(2-formylphenyl)but-3-yn-2-yl)benzamide (3m)

According to general procedure A: Colorless oil, 50.3 mg (76%, 87% ee).

HPLC analysis: Chiralcel AD-H (*n*-hexane/*i*-PrOH = 90/10, flow rate 0.8 mL/min, λ = 254 nm), t_R (minor) = 8.40 min, t_R (major) = 11.80 min.

^{1}H NMR (400 MHz, $CDCl_3$): δ 10.51 (s, 1H), 7.89 (d, *J* = 7.8 Hz, 1H), 7.70 (dd, *J* = 8.0, 1.2 Hz, 1H), 7.55-7.50 (m, 2H), 7.47-7.37 (m, 2H), 7.34 (dd, *J* = 7.6, 1.6 Hz, 1H), 7.27 (td, *J* = 7.6, 1.2 Hz, 1H), 5.77 (s, 1H), 4.64 (q, *J* = 7.0 Hz, 1H), 1.64 (d, *J* = 7.1 Hz, 3H), 1.48 (s, 9H); **^{13}C NMR** (100 MHz, $CDCl_3$): δ 192.0, 169.1, 140.7, 136.3, 136.1, 133.8, 133.4, 130.3, 128.2, 127.9, 127.4, 127.1, 126.93, 126.85, 100.7, 77.7, 52.0, 29.1, 28.8, 24.1.

HRMS (ESI) m/z calcd. for $C_{22}H_{24}NO_2$ $[M+H]^+$ 334.1802, found 334.1798.

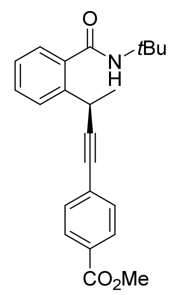

3n

methyl-(*S*)-4-(3-(2-(*tert*-butylcarbamoyl)phenyl)but-1-yn-1-yl)benzoate (3n)

According to general procedure A: Colorless oil, 54.7 mg (76%, 92% ee).

HPLC analysis: Chiralcel AD-H (*n*-hexane/*i*-PrOH = 90/10, flow rate 0.8 mL/min, λ = 254 nm), t_R (minor) = 8.27 min, t_R (major) = 19.26 min.
^{1}H NMR (400 MHz, $CDCl_3$): δ 7.99-7.91 (m, 2H), 7.70 (dd, *J* = 8.0, 1.2 Hz, 1H), 7.49-7.45 (m, 2H), 7.42 (td, *J* = 7.6, 1.6 Hz, 1H), 7.33 (dd, *J* = 7.6, 1.6 Hz, 1H), 7.25 (td, *J* = 7.4, 1.2 Hz, 1H), 5.80 (s, 1H), 4.55 (q, *J* = 7.0 Hz, 1H), 3.89 (s, 3H), 1.62 (d, *J* = 7.0 Hz, 3H), 1.47 (s, 9H); **^{13}C NMR** (100 MHz, $CDCl_3$): δ 169.2, 166.6, 140.8, 136.5, 131.5, 130.2, 129.4, 129.1, 128.5, 127.9, 126.85, 126.82, 96.6, 81.4, 52.2, 52.0, 29.0, 28.8, 24.0.
HRMS (ESI) m/z calcd. for $C_{23}H_{26}NO_3$ $[M+H]^+$ 364.1907, found 364.1904.

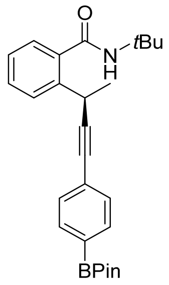

3o

(*S*)-*N*-(*tert*-butyl)-2-(4-(4-(4,4,5,5-tetramethyl-1,3,2-dioxaborolan-2-yl)phenyl)but-3-yn-2-yl)benzamide (3o)
According to general procedure A: Colorless oil, 61.9 mg (72%, 94% ee).
HPLC analysis: Chiralcel AD-H (*n*-hexane/*i*-PrOH = 90/10, flow rate 0.8 mL/min, λ = 254 nm), t_R (minor) = 5.35 min, t_R (major) = 14.91 min.
^{1}H NMR (400 MHz, $CDCl_3$): δ 7.74-7.67 (m, 3H), 7.45-7.38 (m, 3H), 7.33 (dd, *J* = 7.6, 1.6 Hz, 1H), 7.27-7.22 (m, 1H), 5.78 (s, 1H), 4.49 (q, *J* = 7.0 Hz, 1H), 1.61 (d, *J* = 7.0 Hz, 3H), 1.46 (s, 9H), 1.33 (s, 12H); **^{13}C NMR** (100 MHz, $CDCl_3$): δ 169.3, 140.9, 136.6, 134.5, 130.8, 130.1, 127.9, 126.9, 126.7, 126.4, 94.6, 83.9, 82.2, 52.0, 29.1, 28.8, 24.9, 24.0.
HRMS (ESI) m/z calcd. for $C_{27}H_{35}BNO_3$ $[M+H]^+$ 432.2705, found 432.2700.

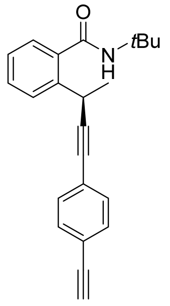

3p

(*S*)-*N*-(*tert*-butyl)-2-(4-(4-ethynylphenyl)but-3-yn-2-yl)benzamide (3p)
According to general procedure A: Colorless oil, 47.2 mg (72%, 92% ee).
HPLC analysis: Chiralcel AD-H (*n*-hexane/*i*-PrOH = 90/10, flow rate 0.8 mL/min, λ = 254 nm), t_R (minor) = 6.53 min, t_R (major) = 12.38 min.
^{1}H NMR (400 MHz, $CDCl_3$): δ 7.70 (dd, *J* = 7.8, 1.2 Hz, 1H), 7.45-7.38 (m, 3H), 7.38-7.31 (m, 3H), 7.29-7.22 (m, 1H), 5.75 (s, 1H), 4.51 (q, *J* = 7.0 Hz, 1H), 3.14 (s, 1H), 1.60 (d, *J* = 7.1 Hz, 3H), 1.46 (s, 9H); **^{13}C NMR** (100 MHz, $CDCl_3$): δ 169.2, 140.9, 136.5, 132.0, 131.5, 130.2, 127.9, 126.84, 126.79, 124.2, 121.4, 95.4, 83.3, 81.5, 78.7, 52.0, 29.0, 28.8, 24.0.
HRMS (ESI) m/z calcd. for $C_{23}H_{24}NO$ $[M+H]^+$ 330.1852, found 330.1848.

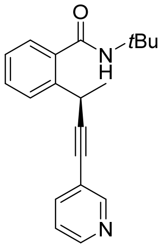

3q

(*S*)-*N*-(*tert*-butyl)-2-(4-(pyridin-3-yl)but-3-yn-2-yl)benzamide (3q)
According to general procedure B: Colorless oil, 34.7 mg (57%, 86% ee).
HPLC analysis: Chiralcel AD-H (*n*-hexane/*i*-PrOH = 80/20, flow rate 0.8 mL/min, λ = 254 nm), t_R (minor) = 5.91 min, t_R (major) = 15.28 min.
^{1}H NMR (400 MHz, $CDCl_3$): δ 8.68-8.60 m, 1H), 8.53-8.45 (m, 1H), 7.76-7.65 (m, 2H), 7.43 (td, *J* = 7.6, 1.6 Hz, 1H), 7.34 (dd, *J* = 7.6, 1.6 Hz, 1H), 7.30-7.15 (m, 2H), 5.77 (s, 1H), 4.55 (q, *J* = 7.0 Hz, 1H), 1.62 (d, *J* = 7.0 Hz, 3H), 1.48 (s, 9H); **^{13}C NMR** (100 MHz, $CDCl_3$): δ 169.2, 152.3, 148.2, 140.8, 138.5, 136.5,

130.2, 127.9, 126.9, 126.8, 123.0, 120.8, 96.8, 78.7, 52.0, 28.9, 28.8, 24.0.
HRMS (ESI) m/z calcd. for $C_{20}H_{23}N_2O$ $[M+H]^+$ 307.1805, found 307.1800.

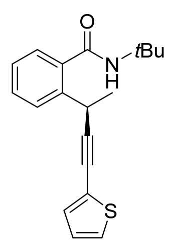
3r

(*S*)-*N*-(*tert*-butyl)-2-(4-(thiophen-2-yl)but-3-yn-2-yl)benzamide (3r)
According to general procedure A: Colorless oil, 38.8 mg (63%, 95% ee).
HPLC analysis: Chiralcel AD-H (*n*-hexane/*i*-PrOH = 90/10, flow rate 0.8 mL/min, λ = 254 nm), t_R (minor) = 6.24 min, t_R (major) = 8.40 min.
^{1}H NMR (400 MHz, $CDCl_3$): δ 7.67 (dd, *J* = 8.0, 1.2 Hz, 1H), 7.45-7.38 (m, 1H), 7.33 (dd, *J* = 7.6, 1.6 Hz, 1H), 7.26 (td, *J* = 8.0, 1.2 Hz, 1H), 7.18 (dd, *J* = 5.2, 1.2 Hz, 1H), 7.14 (dd, *J* = 3.6, 1.2 Hz, 1H), 6.93 (dd, *J* = 5.2, 3.6 Hz, 1H), 5.75 (s, 1H), 4.50 (q, *J* = 7.0 Hz, 1H), 1.61 (d, *J* = 7.0 Hz, 3H), 1.47 (s, 9H); **^{13}C NMR** (100 MHz, $CDCl_3$): δ 169.2, 140.7, 136.6, 131.3, 130.2, 127.9, 126.84, 126.80, 126.3, 123.7, 97.1, 75.2, 52.0, 29.2, 28.8, 23.8.
HRMS (ESI) m/z calcd. for $C_{19}H_{22}NOS$ $[M+H]^+$ 312.1417, found 312.1412.

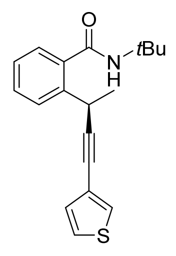
3s

(*S*)-*N*-(*tert*-butyl)-2-(4-(thiophen-2-yl)but-3-yn-2-yl)benzamide (3s)
According to general procedure A: Colorless oil, 51.6 mg (83%, 94% ee).
HPLC analysis: Chiralcel AD-H (*n*-hexane/*i*-PrOH = 90/10, flow rate 0.8 mL/min, λ = 254 nm), t_R (minor) = 7.09 min, t_R (major) = 14.33 min.
^{1}H NMR (400 MHz, $CDCl_3$): δ 7.70 (dd, *J* = 8.0, 1.2 Hz, 1H), 7.41 (td, *J* = 7.6, 1.6 Hz, 1H), 7.36 (dd, *J* = 3.0, 1.2 Hz, 1H), 7.32 (dd, *J* = 7.6, 1.6 Hz, 1H), 7.27-7.21 (m, 2H), 7.07 (dd, *J* = 5.0, 1.2 Hz, 1H), 5.77 (s, 1H), 4.46 (q, *J* = 7.0 Hz, 1H), 1.60 (d, *J* = 7.0 Hz, 3H), 1.46 (s, 9H); **^{13}C NMR** (100 MHz, $CDCl_3$): δ 169.3, 141.0, 136.6, 130.1, 130.0, 128.0, 127.9, 126.9, 126.7, 125.1, 122.6, 92.7, 77.1, 52.0, 29.0, 28.8, 24.0.
HRMS (ESI) m/z calcd. for $C_{19}H_{22}NOS$ $[M+H]^+$ 312.1417, found 312.1412.

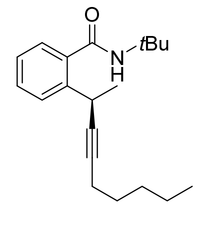
3t

(*S*)-*N*-(*tert*-butyl)-2-(non-3-yn-2-yl)benzamide (3t)
According to general procedure B: White powder, 34.7 mg (58%, 94% ee).
HPLC analysis: Chiralcel AD-H (*n*-hexane/*i*-PrOH = 98/2, flow rate 0.8 mL/min, λ = 214 nm), t_R (minor) = 7.25 min, t_R (major) = 10.10 min.
^{1}H NMR (400 MHz, $CDCl_3$): δ 7.64 (dd, *J* = 8.0, 1.2 Hz, 1H), 7.38 (td, *J* = 7.6, 1.6 Hz, 1H), 7.31 (dd, *J* = 7.6, 1.6 Hz, 1H), 7.22 (td, *J* = 7.6, 1.2 Hz, 1H), 5.77 (s, 1H), 4.22-4.14 (m, 1H), 2.18 (td, *J* = 7.2, 2.3 Hz, 2H), 1.53-1.44 (m, 14H), 1.39-1.28 (m, 4H), 0.89 (t, *J* = 7.1 Hz, 3H); **^{13}C NMR** (100 MHz, $CDCl_3$): δ 169.3, 141.7, 136.5, 129.9, 127.7, 126.8, 126.5, 83.5, 82.2, 51.9, 31.1, 28.8, 28.7, 28.5, 24.3, 22.2, 18.8, 14.0.
HRMS (ESI) m/z calcd. for $C_{20}H_{30}NO$ $[M+H]^+$ 300.2322, found 300.2317.

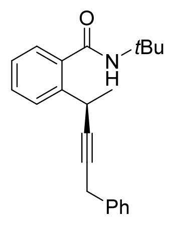
3u

(*S*)-*N*-(*tert*-butyl)-2-(5-phenylpent-3-yn-2-yl)benzamide (3u)
According to general procedure B: Colorless oil, 38.4 mg (60%, 93% ee).

HPLC analysis: Chiralcel AD-H (*n*-hexane/*i*-PrOH = 95/5, flow rate 0.8 mL/min, λ = 214 nm), t_R (minor) = 8.32 min, t_R (major) = 10.33 min.
^{1}H NMR (400 MHz, $CDCl_3$): δ 7.68 (dd, *J* = 8.0, 1.2 Hz, 1H), 7.39 (td, *J* = 7.6, 1.6 Hz, 1H), 7.35-7.28 (m, 5H), 7.25-7.19 (m, 2H), 5.72 (s, 1H), 4.34-4.25 (m, 1H), 3.63 (d, *J* = 2.4 Hz, 2H), 1.54 (d, *J* = 7.0 Hz, 3H), 1.45 (s, 9H); **^{13}C NMR** (100 MHz, $CDCl_3$): δ 169.3, 141.5, 137.3, 136.5, 130.0, 128.4, 127.9, 127.8, 126.8, 126.6, 126.5, 85.9, 79.4, 51.9, 28.8, 28.5, 25.2, 24.3.
HRMS (ESI) m/z calcd. for $C_{22}H_{26}NO$ $[M+H]^+$ 320.2009, found 320.2006.

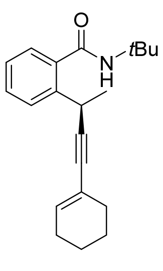

3v

(*S*)-*N*-(*tert*-butyl)-2-(4-(cyclohex-1-en-1-yl)but-3-yn-2-yl)benzamide (3v)
According to general procedure A: Colorless oil, 39.2 mg (64%, 90% ee).
HPLC analysis: Chiralcel AD-H (*n*-hexane/*i*-PrOH = 98/2, flow rate 0.8 mL/min, λ = 214 nm), t_R (minor) = 8.38 min, t_R (major) = 12.18 min.
^{1}H NMR (400 MHz, $CDCl_3$): δ 7.64 (dd, *J* = 8.0, 1.2 Hz, 1H), 7.38 (td, *J* = 7.6, 1.6 Hz, 1H), 7.31 (dd, *J* = 7.6, 1.6 Hz, 1H), 7.22 (td, *J* = 7.4, 1.2 Hz, 1H), 6.04 (dt, *J* = 4.0, 2.0 Hz, 1H), 5.75 (s, 1H), 4.33 (q, *J* = 7.0 Hz, 1H), 2.13-2.03 (m, 4H), 1.67-1.55 (m, 4H), 1.52 (d, *J* = 7.0 Hz, 3H), 1.46 (s, 9H); **^{13}C NMR** (100 MHz, $CDCl_3$): δ 169.3, 141.3, 136.5, 133.9, 130.0, 127.8, 126.8, 126.6, 120.8, 90.3, 83.9, 51.9, 29.5, 28.9, 28.8, 25.6, 24.2, 22.4, 21.6.
HRMS (ESI) m/z calcd. for $C_{21}H_{28}NO$ $[M+H]^+$ 310.2165, found 310.2161.

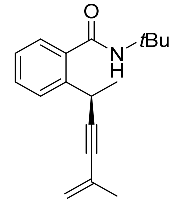

3w

(*S*)-*N*-(*tert*-butyl)-2-(5-methylhex-5-en-3-yn-2-yl)benzamide (3w)
According to general procedure B: Colorless oil, 44.0 mg (81%, 94% ee).
HPLC analysis: Chiralcel AD-H (*n*-hexane/*i*-PrOH = 98/2, flow rate 0.8 mL/min, λ = 214 nm), t_R (minor) = 7.51 min, t_R (major) = 10.03 min.
^{1}H NMR (400 MHz, $CDCl_3$): δ 7.63 (dd, *J* = 8.0, 1.2 Hz, 1H), 7.40 (td, *J* = 7.6, 1.6 Hz, 1H), 7.31 (dd, *J* = 7.6, 1.6 Hz, 1H), 7.23 (td, *J* = 7.4, 1.2 Hz, 1H), 5.73 (s, 1H), 5.26-5.13 (m, 2H), 4.35 (q, *J* = 7.0 Hz, 1H), 1.88 (t, *J* = 1.2 Hz, 3H), 1.53 (d, *J* = 7.0 Hz, 3H), 1.47 (s, 9H); **^{13}C NMR** (100 MHz, $CDCl_3$): δ 169.2, 141.1, 136.5, 130.1, 127.8, 127.1, 126.8, 126.6, 120.9, 92.2, 83.3, 51.9, 28.9, 28.8, 24.1, 23.8.
HRMS (ESI) m/z calcd. for $C_{18}H_{24}NO$ $[M+H]^+$ 270.1852, found 270.1848.

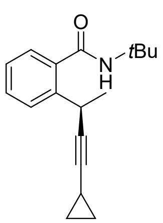

3x

(*S*)-*N*-(*tert*-butyl)-2-(4-cyclopropylbut-3-yn-2-yl)benzamide (3x)
According to general procedure B: Colorless oil, 45.9 mg (85%, 94% ee).
HPLC analysis: Chiralcel AD-H (*n*-hexane/*i*-PrOH = 95/5, flow rate 0.8 mL/min, λ = 214 nm), t_R (minor) = 6.98 min, t_R (major) = 10.08 min.
^{1}H NMR (400 MHz, $CDCl_3$): δ 7.60 (d, *J* = 7.8 Hz, 1H), 7.37 (td, *J* = 7.6, 1.6 Hz, 1H), 7.33-7.26 (m, 1H), 7.21 (td, *J* = 7.6, 1.2 Hz, 1H), 5.79 (s, 1H), 4.15 (qd, *J* = 7.0, 2.0 Hz, 1H), 1.47 (m, *J* = 6.0 Hz, 12H), 1.25-1.19 (m, 1H), 0.74-0.67 (m, 2H), 0.67-0.58 (m, 2H); **^{13}C NMR** (100 MHz, $CDCl_3$): δ 169.7, 141.8, 136.9, 130.3, 128.0, 127.2, 126.9, 85.4, 79.2, 52.3, 29.2, 28.8, 24.6, 8.5, 8.4, 0.0.

HRMS (ESI) m/z calcd. for $C_{18}H_{24}NO$ $[M+H]^+$ 270.1852, found 270.1848.

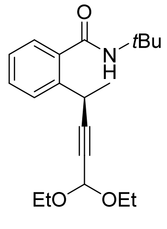
3y

(*S*)-*N*-(*tert*-butyl)-2-(5,5-diethoxypent-3-yn-2-yl)benzamide (3y)

According to general procedure A: Colorless oil, 62.5 mg (94%, 94% ee).

HPLC analysis: Chiralcel IC (*n*-hexane/*i*-PrOH = 95/5, flow rate 0.8 mL/min, λ = 214 nm), t_R (minor) = 14.65 min, t_R (major) = 19.40 min.

^{1}H NMR (400 MHz, $CDCl_3$): δ 7.64 (dd, *J* = 7.8, 1.2 Hz, 1H), 7.39 (td, *J* = 7.6, 1.6 Hz, 1H), 7.30 (dd, *J* = 7.6, 1.6 Hz, 1H), 7.23 (td, *J* = 7.4, 1.2 Hz, 1H), 5.68 (s, 1H), 5.30 (d, *J* = 1.6 Hz, 1H), 4.34 (qd, *J* = 7.0, 1.6 Hz, 1H), 3.80-3.68 (m, 2H), 3.63-3.52 (m, 2H), 1.53 (d, *J* = 7.0 Hz, 3H), 1.46 (s, 9H), 1.23 (td, *J* = 7.1, 2.0 Hz, 6H); **^{13}C NMR** (100 MHz, $CDCl_3$): δ 169.1, 140.6, 136.4, 130.1, 128.0, 126.72, 126.67, 91.5, 89.1, 77.2, 60.72, 60.70, 52.0, 28.8, 28.4, 24.0, 15.11, 15.10.

HRMS (ESI) m/z calcd. for $C_{20}H_{29}NO_3Na$ $[M+Na]^+$ 354.2040, found 354.2036.

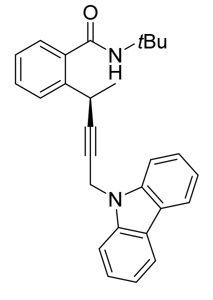
3z

(*S*)-2-(5-(9*H*-carbazol-9-yl)pent-3-yn-2-yl)-*N*-(*tert*-butyl)benzamide (3z)

According to general procedure A: Yellow powder, 65.7 mg (81%, 89% ee).

HPLC analysis: Chiralcel AD-H (*n*-hexane/*i*-PrOH = 90/10, flow rate 0.8 mL/min, λ = 214 nm), t_R (major) = 10.66 min, t_R (minor) = 13.63 min.

^{1}H NMR (400 MHz, $CDCl_3$): δ 8.08 (dt, *J* = 7.8, 1.0 Hz, 2H), 7.52 -7.39 (m, 5H), 7.32-7.22 (m, 4H), 7.17 (td, *J* = 7.4, 1.2 Hz, 1H), 5.54 (s, 1H), 5.05 (d, *J* = 2.1 Hz, 2H), 4.22 (dt, *J* = 7.0, 2.1 Hz, 1H), 1.43 (d, *J* = 7.0 Hz, 3H), 1.34 (s, 9H); **^{13}C NMR** (100 MHz, $CDCl_3$): δ 169.2, 140.8, 140.0, 136.4, 130.0, 127.9, 126.7, 125.8, 123.2, 120.4, 119.3, 109.0, 87.8, 75.8, 51.9, 32.9, 28.7, 28.3, 24.1.

HRMS (ESI) m/z calcd. for $C_{28}H_{29}N_2O$ $[M+H]^+$ 409.2274, found 409.2269.

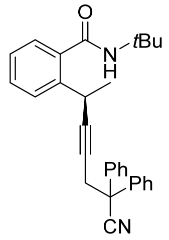
3za

(*S*)-*N*-(*tert*-butyl)-2-(6-cyano-6,6-diphenylhex-3-yn-2-yl)benzamide (3za)

According to general procedure A: Yellow oil, 42.4 mg (49%, 88% ee).

HPLC analysis: Chiralcel AD-H (*n*-hexane/*i*-PrOH = 99/1, flow rate 0.8 mL/min, λ = 214 nm), t_R (minor) = 48.66 min, t_R (major) = 52.79 min.

^{1}H NMR (400 MHz, $CDCl_3$): δ 7.41-7.30 (m, 11H), 7.29-7.23 (m, 2H), 7.20-7.15 (m, 1H), 5.62 (s, 1H), 4.20-4.11 (m, 1H), 3.35-3.12 (m, 2H), 1.41 (s, 9H), 1.38 (d, *J* = 6.9 Hz, 3H); **^{13}C NMR** (100 MHz, $CDCl_3$): δ 169.3, 140.8, 139.19, 139.16, 136.3, 130.0, 128.8, 128.20, 128.16, 127.9, 127.2, 126.6, 126.5, 121.9, 88.8, 76.4, 51.9, 51.7, 31.4, 28.8, 28.3, 23.9.

HRMS (ESI) m/z calcd. for $C_{30}H_{31}N_2O$ $[M+H]^+$ 435.2431, found 435.2427.

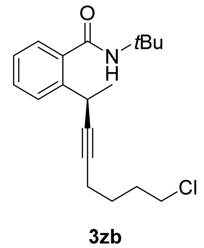
3zb

(*S*)-*N*-(*tert*-butyl)-2-(8-chlorooct-3-yn-2-yl)benzamide (3zb)

According to general procedure B: Colorless oil, 52.6 mg (82%, 93% ee).

HPLC analysis: Chiralcel AD-H (*n*-hexane/*i*-PrOH = 95/5, flow rate 0.8 mL/min, λ = 214 nm), t_R (minor) = 8.70 min, t_R (major) = 11.37 min.
^{1}H NMR (400 MHz, $CDCl_3$): δ 7.63 (dd, *J* = 8.0, 1.2 Hz, 1H), 7.39 (td, *J* = 7.6, 1.5 Hz, 1H), 7.30 (dd, *J* = 7.6, 1.6 Hz, 1H), 7.22 (td, *J* = 7.4, 1.2 Hz, 1H), 5.75 (s, 1H), 4.29-4.16 (m, 1H), 3.55 (t, *J* = 6.6 Hz, 2H), 2.24 (td, *J* = 6.9, 2.4 Hz, 2H), 1.94-1.81 (m, 2H), 1.66 (dt, *J* = 14.6, 7.2 Hz, 2H), 1.48 (d, *J* = 7.0 Hz, 3H), 1.46 (s, 9H); **^{13}C NMR** (100 MHz, $CDCl_3$): δ 169.3, 141.6, 136.5, 130.0, 127.7, 126.8, 126.5, 84.3, 81.1, 51.9, 44.6, 31.6, 28.8, 28.4, 26.1, 24.4, 18.2.
HRMS (ESI) m/z calcd. for $C_{19}H_{27}{}^{34.9689}ClNO$ $[M+H]^+$ 320.1776, found 320.1772; calcd. for $C_{19}H_{27}{}^{36.9659}ClNO$ $[M+H]^+$ 322.1746, found 322.1742.

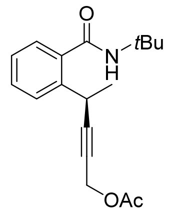

3zc

(*S*)-4-(2-(*tert*-butylcarbamoyl)phenyl)pent-2-yn-1-yl acetate (3zc)
According to general procedure A: Colorless oil, 41.6 mg (69%, 93% ee).
HPLC analysis: Chiralcel AD-H (*n*-hexane/*i*-PrOH = 90/10, flow rate 0.8 mL/min, λ = 214 nm), t_R (minor) = 10.38 min, t_R (major) = 17.83 min.
^{1}H NMR (400 MHz, $CDCl_3$): δ 7.61 (dd, *J* = 8.0, 1.2 Hz, 1H), 7.40 (td, *J* = 7.6, 1.6 Hz, 1H), 7.31 (dd, *J* = 7.6, 1.6 Hz, 1H), 7.24 (td, *J* = 7.6, 1.2 Hz, 1H), 5.72 (s, 1H), 4.70 (d, *J* = 2.1 Hz, 2H), 4.32 (dt, *J* = 7.1, 2.1 Hz, 1H), 2.08 (s, 3H), 1.52 (d, *J* = 7.1 Hz, 3H), 1.46 (s, 9H); **^{13}C NMR** (100 MHz, $CDCl_3$): δ 170.3, 169.1, 140.6, 136.5, 130.1, 127.8, 126.8, 90.5, 75.5, 52.8, 52.0, 28.8, 28.4, 23.8, 20.8.
HRMS (ESI) m/z calcd. for $C_{18}H_{24}NO_3$ $[M+H]^+$ 302.1751, found 302.1746.

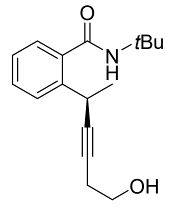

3zd

(*S*)-*N*-(*tert*-butyl)-2-(6-hydroxyhex-3-yn-2-yl)benzamide (3zd)
According to general procedure B: Colorless oil, 50.7 mg (92%, 93% ee).
HPLC analysis: Chiralcel AD-H (*n*-hexane/*i*-PrOH = 80/20, flow rate 0.8 mL/min, λ = 214 nm), t_R (minor) = 5.55 min, t_R (major) = 7.97 min.
^{1}H NMR (400 MHz, $CDCl_3$): δ 7.61 (dd, *J* = 7.8, 1.2 Hz, 1H), 7.39 (td, *J* = 7.6, 1.6 Hz, 1H), 7.30 (dd, *J* = 7.6, 1.6 Hz, 1H), 7.23 (td, *J* = 7.6, 1.2 Hz, 1H), 5.75 (s, 1H), 4.28-4.17 (m, 1H), 3.67 (q, *J* = 6.0 Hz, 2H), 2.45 (td, *J* = 6.4, 2.4 Hz, 2H), 2.17 (s, 1H), 1.50 (d, *J* = 7.0 Hz, 3H), 1.46 (s, 9H); **^{13}C NMR** (100 MHz, $CDCl_3$): δ 169.3, 141.3, 136.5, 130.1, 127.6, 126.8, 126.6, 85.8, 78.3, 61.2, 52.0, 28.8, 28.4, 24.1, 23.3.
HRMS (ESI) m/z calcd. for $C_{17}H_{24}NO_2$ $[M+H]^+$ 274.1802, found 274.1797.

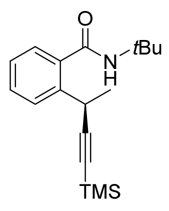

3ze

(*R*)-*N*-(*tert*-butyl)-2-(4-(trimethylsilyl)but-3-yn-2-yl)benzamide (3ze)
According to general procedure B:White powder, 46.9 mg (78%, 97% ee).
HPLC analysis: Chiralcel AS3 (*n*-hexane/*i*-PrOH = 99/1, flow rate 0.6 mL/min, λ = 214 nm), t_R (minor) = 7.52 min, t_R (major) = 7.98 min.
^{1}H NMR (400 MHz, $CDCl_3$): δ 7.65 (dd, *J* = 7.8, 1.2 Hz, 1H), 7.40 (td, *J* = 7.6, 1.6 Hz, 1H), 7.31 (dd, *J* = 7.6, 1.6 Hz, 1H), 7.23 (td, *J* = 7.6, 1.2 Hz, 1H), 5.74 (s, 1H), 4.25 (q, *J* = 7.0 Hz, 1H), 1.51 (d, *J* = 7.0 Hz, 3H), 1.46 (s, 9H), 0.16 (s,

9H); **^{13}C NMR** (100 MHz, $CDCl_3$): δ 169.2, 140.8, 136.5, 130.1, 127.8, 126.8, 126.7, 110.1, 85.9, 52.0, 29.4, 28.8, 24.3, 0.2.
HRMS (ESI) m/z calcd. for $C_{18}H_{28}NOSi$ $[M+H]^+$ 302.1935, found 302.1930.

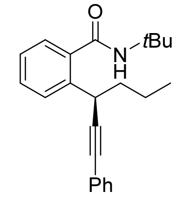

3zf

(*S*)-*N*-(*tert*-butyl)-2-(1-phenylhex-1-yn-3-yl)benzamide (3zf)
According to general procedure A: Colorless oil, 52.1 mg (78%, 94% ee).
HPLC analysis: Chiralcel AD-H (*n*-hexane/*i*-PrOH = 90/10, flow rate 0.8 mL/min, λ = 254 nm), t_R (minor) = 5.51 min, t_R (major) = 7.57 min.
^{1}H NMR (400 MHz, $CDCl_3$): δ 7.69 (dd, *J* = 7.8, 1.2 Hz, 1H), 7.44-7.36 (m, 3H), 7.32 (dd, *J* = 7.6, 1.6 Hz, 1H), 7.29-7.21 (m, 4H), 5.74 (s, 1H), 4.39 (dd, *J* = 8.8, 5.7 Hz, 1H), 1.89-1.78 (m, 2H), 1.69-1.52 (m, 2H), 1.46 (s, 9H), 0.96 (t, *J* = 7.3 Hz, 3H); **^{13}C NMR** (100 MHz, $CDCl_3$): δ 169.3, 140.1, 136.9, 131.6, 129.9, 128.4, 128.2, 127.8, 126.8, 126.6, 123.8, 92.1, 83.0, 51.9, 40.2, 34.4, 28.8, 21.0, 13.8.
HRMS (ESI) m/z calcd. for $C_{23}H_{28}NO$ $[M+H]^+$ 334.2165, found 334.2161.

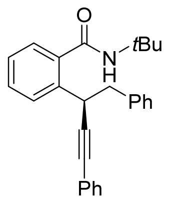

3zg

(*S*)-*N*-(*ter*t-butyl)-2-(1,4-diphenylbut-3-yn-2-yl)benzamide (3zg)
According to general procedure A: Colorless oil, 43.7 mg (58%, 94% ee).
HPLC analysis: Chiralcel AD-H (*n*-hexane/*i*-PrOH = 90/10, flow rate 0.8 mL/min, λ = 254 nm), t_R (minor) = 6.52 min, t_R (major) = 9.66 min.
^{1}H NMR (400 MHz, $CDCl_3$): δ 7.70 (dd, *J* = 8.0, 1.2 Hz, 1H), 7.43-7.37 (m, 1H), 7.36-7.30 (m, 3H), 7.29-7.18 (m, 9H), 5.56 (s, 1H), 4.62 (dd, *J* = 9.2, 5.3 Hz, 1H), 3.29 (dd, *J* = 12.9, 5.3 Hz, 1H), 3.01 (dd, *J* = 12.9, 9.2 Hz, 1H), 1.45 (s, 9H); **^{13}C NMR** (100 MHz, $CDCl_3$): δ 169.3, 139.5, 139.3, 136.9, 131.6, 129.9, 129.7, 128.9, 128.2, 128.1, 127.8, 126.9, 126.7, 126.4, 123.7, 91.4, 84.0, 51.9, 44.6, 37.3, 28.8.
HRMS (ESI) m/z calcd. for $C_{27}H_{28}NO$ $[M+H]^+$ 382.2165, found 382.2160.

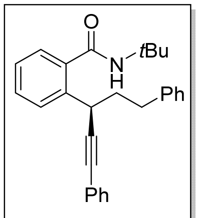

3zh

(*R*)-*N*-(*tert*-butyl)-2-(1,5-diphenylpent-1-yn-3-yl)benzamide (3zh)
According to general procedure A: Colorless oil, 66.8 mg (85%, 92% ee).
HPLC analysis: Chiralcel AD-H (*n*-hexane/*i*-PrOH = 96/4, flow rate 0.8 mL/min, λ = 254 nm), t_R (minor) = 17.78 min, t_R (major) = 20.22 min.
^{1}H NMR (400 MHz, $CDCl_3$): δ 7.71 (dd, *J* = 8.0, 1.2 Hz, 1H), 7.48-7.38 (m, 3H), 7.35-7.20 (m, 9H), 7.18-7.12 (m, 1H), 5.71 (s, 1H), 4.41 (dd, *J* = 9.4, 5.2 Hz, 1H), 2.99-2.89 (m, 1H), 2.87-2.77 (m, 1H), 2.29-2.06 (m, 2H), 1.41 (s, 9H); **^{13}C NMR** (100 MHz, $CDCl_3$): δ 169.1, 141.7, 139.5, 136.9, 131.7, 130.0, 128.6, 128.39, 128.38, 128.3, 127.9, 127.0, 126.8, 125.9, 123.6, 91.6, 83.7, 51.9, 39.5, 34.4, 34.1, 28.8.
HRMS (ESI) m/z calcd. for $C_{28}H_{30}NO$ $[M+H]^+$ 396.2322, found 396.2317.

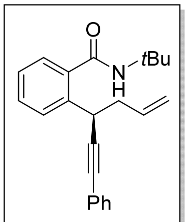

3zi

(*S*)-*N*-(*tert*-butyl)-2-(1-phenylhex-5-en-1-yn-3-yl)benzamide (3zi)
According to general procedure A: Colorless oil, 51.7 mg (78%, 96% ee).

HPLC analysis: Chiralcel AD-H (*n*-hexane/*i*-PrOH = 90/10, flow rate 0.8 mL/min, λ = 254 nm), t_R (minor) = 5.60 min, t_R (major) = 9.17 min.
^{1}H NMR (400 MHz, $CDCl_3$): δ 7.69 (dd, *J* = 7.9, 1.2 Hz, 1H), 7.44-7.37 (m, 3H), 7.33 (dd, *J* = 7.6, 1.5 Hz, 1H), 7.30-7.22 (m, 4H), 6.04-5.85 (m, 1H), 5.74 (s, 1H), 5.19-5.00 (m, 2H), 4.48 (dd, *J* = 8.6, 5.4 Hz, 1H), 2.75-2.47 (m, 2H), 1.46 (s, 9H); **^{13}C NMR** (100 MHz, $CDCl_3$): δ 169.2, 139.2, 136.8, 135.5, 131.7, 129.9, 128.6, 128.2, 127.9, 126.83, 126.82, 123.6, 117.2, 91.4, 83.5, 52.0, 42.1, 34.7, 28.8.
HRMS (ESI) m/z calcd. for $C_{23}H_{26}NO$ $[M+H]^+$ 332.2009, found 332.2003.

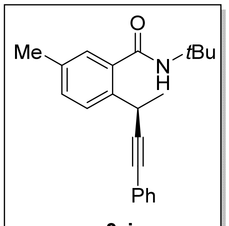

3zj

(*S*)-*N*-(*tert*-butyl)-5-methyl-2-(4-phenylbut-3-yn-2-yl)benzamide (3zj)
According to general procedure A: Colorless oil, 53.9 mg (85%, 93% ee).
HPLC analysis: Chiralcel AD-H (*n*-hexane/*i*-PrOH = 90/10, flow rate 0.8 mL/min, λ = 254 nm), t_R (minor) = 6.26 min, t_R (major) = 14.59 min.
^{1}H NMR (400 MHz, $CDCl_3$): δ 7.58 (d, *J* = 7.9 Hz, 1H), 7.42-7.36 (m, 2H), 7.29-7.20 (m, 4H), 7.14 (s, 1H), 5.77 (s, 1H), 4.42 (q, *J* = 7.0 Hz, 1H), 2.33 (s, 3H), 1.59 (d, *J* = 7.0 Hz, 3H), 1.46 (s, 9H); **^{13}C NMR** (100 MHz, $CDCl_3$): δ 169.4, 138.0, 136.52, 136.45, 131.6, 130.8, 128.2, 127.8, 127.4, 123.7, 93.4, 82.0, 51.9, 28.8, 28.7, 24.1, 20.9.
HRMS (ESI) m/z calcd. for $C_{22}H_{26}NO$ $[M+H]^+$ 320.2009, found 320.2005.

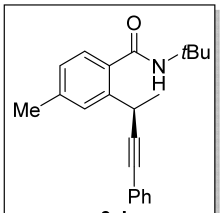

3zk

(*S*)-*N*-(*tert*-butyl)-4-methyl-2-(4-phenylbut-3-yn-2-yl)benzamide (3zk)
According to general procedure A: Colorless oil, 38.2 mg (60%, 90% ee).
HPLC analysis: Chiralcel AD-H (*n*-hexane/*i*-PrOH = 90/10, flow rate 0.8 mL/min, λ = 254 nm), t_R (minor) = 5.82 min, t_R (major) = 11.10 min.
^{1}H NMR (400 MHz, $CDCl_3$): δ 7.50 (d, *J* = 1.1 Hz, 1H), 7.44-7.39 (m, 2H), 7.30-7.22 (m, 4H), 7.07-7.02 (m, 1H), 5.76 (s, 1H), 4.49 (q, *J* = 7.0 Hz, 1H), 2.38 (s, 3H), 1.60 (d, *J* = 7.0 Hz, 3H), 1.46 (s, 9H); **^{13}C NMR** (100 MHz, $CDCl_3$): δ 169.4, 141.1, 140.2, 133.8, 131.6, 128.5, 128.2, 127.8, 127.4, 126.9, 123.7, 93.4, 82.0, 51.9, 28.84, 28.82, 24.1, 21.5.
HRMS (ESI) m/z calcd. for $C_{22}H_{26}NO$ $[M+H]^+$ 320.2009, found 320.2006.

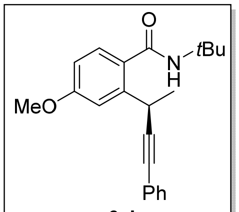

3zl

(*S*)-*N*-(*tert*-butyl)-4-methoxy-2-(4-phenylbut-3-yn-2-yl)benzamide (3zl)
According to general procedure A: White powder, 36.9 mg (55%, 90% ee).
HPLC analysis: Chiralcel AD-H (*n*-hexane/*i*-PrOH = 90/10, flow rate 0.8 mL/min, λ = 254 nm), t_R (minor) = 8.04 min, t_R (major) = 14.57 min.
^{1}H NMR (400 MHz, $CDCl_3$): δ 7.44-7.37 (m, 2H), 7.30-7.22 (m, 5H), 6.75 (dd, *J* = 8.4, 2.6 Hz, 1H), 5.75 (s, 1H), 4.57 (q, *J* = 7.0 Hz, 1H), 3.83 (s, 3H), 1.60 (d, *J* = 7.0 Hz, 3H), 1.46 (s, 9H); **^{13}C NMR** (100 MHz, $CDCl_3$): δ 169.1, 160.8, 143.5, 131.6, 129.1, 128.6, 128.2, 127.8, 123.6, 113.6, 111.6, 93.1, 82.2, 55.3, 51.8, 29.1, 28.8, 24.1.
HRMS (ESI) m/z calcd. for $C_{22}H_{26}NO_2$ $[M+H]^+$ 336.1958, found 336.1954.

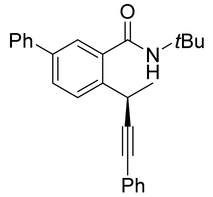

3zm

(*S*)-*N*-(*tert*-butyl)-4-(4-phenylbut-3-yn-2-yl)-[1,1'-biphenyl]-3-carboxamide (3zm)

According to general procedure A: White powder, 62.1 mg (81%, 92% ee).
HPLC analysis: Chiralcel AD-H (*n*-hexane/*i*-PrOH = 95/5, flow rate 0.8 mL/min, λ = 254 nm), t_R (minor) = 13.86 min, t_R (major) = 38.41 min.
^{1}H NMR (400 MHz, $CDCl_3$): δ 7.78 (d, *J* = 8.0 Hz, 1H), 7.62 (dd, *J* = 8.1, 2.1 Hz, 1H), 7.59-7.53 (m, 3H), 7.47-7.40 (m, 4H), 7.38-7.32 (m, 1H), 7.30-7.25 (m, 3H), 5.82 (s, 1H), 4.49 (q, *J* = 7.0 Hz, 1H), 1.65 (d, *J* = 7.0 Hz, 3H), 1.48 (s, 9H); **^{13}C NMR** (100 MHz, $CDCl_3$): δ 169.2, 140.2, 140.0, 139.9, 137.1, 131.6, 128.9, 128.7, 128.4, 128.3, 127.9, 127.6, 127.1, 125.6, 123.6, 93.1, 82.2, 52.1, 28.8, 24.0.
HRMS (ESI) m/z calcd. for $C_{27}H_{28}NO$ $[M+H]^+$ 382.2165, found 382.2161.

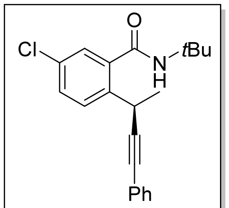

3zn

(*S*)-*N*-(*tert*-butyl)-5-chloro-2-(4-phenylbut-3-yn-2-yl)benzamide (3zn)

According to general procedure A: Colorless oil, 56.4 mg (83%, 93% ee).
HPLC analysis: Chiralcel AD-H (*n*-hexane/*i*-PrOH = 90/10, flow rate 0.8 mL/min, λ = 254 nm), t_R (minor) = 5.79 min, t_R (major) = 10.13 min.
^{1}H NMR (400 MHz, $CDCl_3$): δ 7.69 (d, *J* = 2.0 Hz, 1H), 7.45-7.38 (m, 2H), 7.32-7.25 (m, 4H), 7.21 (dd, *J* = 8.0, 2.0 Hz, 1H), 5.78 (s, 1H), 4.48 (q, *J* = 7.0 Hz, 1H), 1.59 (d, *J* = 7.0 Hz, 3H), 1.45 (s, 9H); **^{13}C NMR** (101 MHz, $CDCl_3$): δ 168.3, 143.2, 136.0, 134.9, 131.6, 128.32, 128.27, 128.1, 128.0, 126.9, 123.3, 92.2, 82.7, 52.1, 28.9, 28.8, 23.9.
HRMS (ESI) m/z calcd. for $C_{21}H_{23}{}^{34.9689}ClNO$ $[M+H]^+$ 340.1463, found 340.1459; calcd. for $C_{21}H_{23}{}^{36.9659}ClNO$ $[M+H]^+$ 342.1433, found 342.1429.

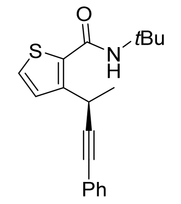

3zo

(*S*)-*N*-(*tert*-butyl)-3-(4-phenylbut-3-yn-2-yl)thiophene-2-carboxamide (3zo)

According to general procedure A: Colorless oil, 42.7 mg (69%, 88% ee).
HPLC analysis: Chiralcel AD-H (*n*-hexane/*i*-PrOH = 98/2, flow rate 0.8 mL/min, λ = 254 nm), t_R (minor) = 7.37 min, t_R (major) = 7.95 min.
^{1}H NMR (400 MHz, $CDCl_3$): δ 7.39 (dd, *J* = 6.7, 3.0 Hz, 2H), 7.33-7.18 (m, 5H), 6.02 (s, 1H), 4.74 (q, *J* = 7.1 Hz, 1H), 1.61 (d, *J* = 7.1 Hz, 3H), 1.45 (s, 9H); **^{13}C NMR** (100 MHz, $CDCl_3$): δ 162.1, 145.5, 132.2, 131.6, 129.0, 128.2, 127.9, 126.1, 123.4, 92.4, 82.1, 52.1, 28.9, 26.4, 23.0.
HRMS (ESI) m/z calcd. for $C_{19}H_{22}NOS$ $[M+H]^+$ 312.1417, found 312.1413.

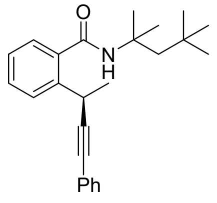

3zp

(*S*)-2-(4-phenylbut-3-yn-2-yl)-*N*-(2,4,4-trimethylpentan-2-yl)benzamide (3zp)

According to general procedure A: Colorless oil, 60.9 mg (84%, 93% ee).
HPLC analysis: Chiralcel AD-H (*n*-hexane/*i*-PrOH = 98/2, flow rate 0.8 mL/min, λ = 254 nm), t_R (minor) = 12.87 min, t_R (major) = 20.06 min.

^{1}H NMR (400 MHz, $CDCl_3$): δ 7.73 (dd, *J* = 8.0, 1.2 Hz, 1H), 7.44-7.38 (m, 3H), 7.32 (dd, *J* = 7.6, 1.6 Hz, 1H), 7.28 -7.22 (m, 4H), 5.74 (s, 1H), 4.53 (q, *J* = 7.0 Hz, 1H), 1.92 (d, *J* = 15.0 Hz, 1H), 1.80 (d, *J* = 15.0 Hz, 1H), 1.61 (d, *J* = 7.0 Hz, 3H), 1.54 (s, 3H), 1.51 (s, 3H), 1.05 (s, 9H); **^{13}C NMR** (100 MHz, $CDCl_3$): δ 168.9, 141.5, 136.6, 131.6, 130.1, 128.2, 128.1, 127.8, 126.7, 126.5, 123.7, 93.3, 81.9, 56.0, 51.9, 31.7, 31.6, 29.2, 29.1, 28.9, 24.3.

HRMS (ESI) m/z calcd. for $C_{25}H_{32}NO$ $[M+H]^+$ 362.2478, found 362.2474.

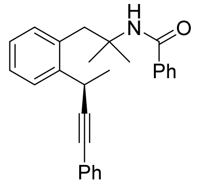

3zq

(*S*)-*N*-(2-methyl-1-(2-(4-phenylbut-3-yn-2-yl)phenyl)propan-2-yl)benzamide (3zq)

According to general procedure A: Colorless oil, 52.8 mg (69%, 89% ee).

HPLC analysis: Chiralcel AD-H (*n*-hexane/*i*-PrOH = 96/4, flow rate 0.8 mL/min, λ = 254 nm), t_R (major) = 16.54 min, t_R (minor) = 19.30 min.

^{1}H NMR (400 MHz, $CDCl_3$): δ 7.73-7.66 (m, 3H), 7.46-7.41 (m, 1H), 7.39-7.33 (m, 4H), 7.28-7.23 (m, 4H), 7.15-7.09 (m, 2H), 5.84 (s, 1H), 4.38 (q, *J* = 7.0 Hz, 1H), 3.32-3.24 (m, 2H), 1.56 (s, 3H), 1.51 (d, *J* = 7.0 Hz, 3H), 1.47 (s, 3H); **^{13}C NMR** (100 MHz, $CDCl_3$): δ 167.5, 143.2, 135.9, 134.1, 131.8, 131.6, 131.2, 128.5, 128.1, 127.70, 127.67, 127.3, 126.8, 126.3, 123.8, 93.5, 81.5, 55.1, 40.1, 28.8, 27.9, 27.4, 24.6.

HRMS (ESI) m/z calcd. for $C_{27}H_{28}NO$ $[M+H]^+$ 382.2165, found 382.2161.

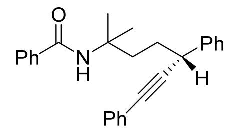

3zr

(*R*)-*N*-(2-methyl-5,7-diphenylhept-6-yn-2-yl)benzamide (3zr)

According to general procedure C: Colorless oil, 57.6 mg (72%, 91% ee).

HPLC analysis: Chiralcel AD-H (*n*-hexane/*i*-PrOH = 90/10, flow rate 0.8 mL/min, λ = 254 nm), t_R (minor) = 9.59 min, t_R (major) = 13.82 min.

^{1}H NMR (400 MHz, $CDCl_3$): δ 7.71-7.64 (m, 2H), 7.47-7.40 (m, 5H), 7.40-7.24 (m, 8H), 5.86 (s, 1H), 3.85 (t, *J* = 7.1 Hz, 1H), 2.07-1.99 (m, 2H), 1.91-1.84 (m, 2H), 1.44 (s, 3H), 1.43 (s, 3H); **^{13}C NMR** (100 MHz, $CDCl_3$): δ 166.9, 141.9, 135.8, 131.7, 131.1, 128.6, 128.5, 128.3, 127.9, 127.5, 126.9, 126.7, 123.6, 91.3, 83.7, 54.0, 38.5, 38.3, 33.6, 27.1, 27.0.

HRMS (ESI) m/z calcd. for $C_{27}H_{28}NO$ $[M+H]^+$ 382.2165, found 382.2161.

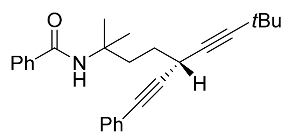

3zs

(*S*)-*N*-(2,8,8-trimethyl-5-(phenylethynyl)non-6-yn-2-yl)benzamide (3zs)

According to general procedure C: Colorless oil, 52.9 mg (69%, 78% ee).

HPLC analysis: Chiralcel AD3 (*n*-hexane/*i*-PrOH = 98/2, flow rate 0.8 mL/min, λ = 254 nm), t_R (minor) = 13.31 min, t_R (major) = 13.63 min.

^{1}H NMR (400 MHz, $CDCl_3$): δ 7.77-7.66 (m, 2H), 7.47-7.35 (m, 6H), 7.30-7.26 (m, 2H), 5.94 (s, 1H), 3.57 (t, *J* = 6.8 Hz, 1H), 2.13-2.02 (m, 2H), 1.86-1.77 (m, 2H), 1.48 (d, *J* = 1.7 Hz, 6H), 1.22 (s, 9H); **^{13}C NMR** (100 MHz, $CDCl_3$): δ 166.9, 135.8, 131.8, 131.1, 128.5, 128.2, 128.0, 126.8, 123.3, 90.2, 88.7, 80.9, 76.5, 53.9, 37.9, 31.4, 31.1, 27.4, 27.0, 24.1.

HRMS (ESI) m/z calcd. for $C_{27}H_{32}NO$ $[M+H]^+$ 386.2478, found 386.2476.

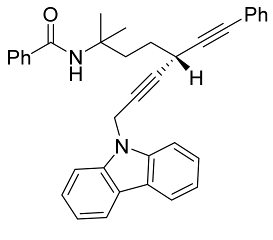

3zt

(*R*)-*N*-(8-(9*H*-carbazol-9-yl)-2-methyl-5-(phenylethynyl)oct-6-yn-2-yl)benzamide (3zt)

According to general procedure C: Yellow oil, 54.7 mg (56%, 87% ee).

HPLC analysis: Chiralcel AD-H (*n*-hexane/*i*-PrOH = 90/10, flow rate 0.8 mL/min, λ = 230 nm), t_R (major) = 26.30 min, t_R (minor) = 32.30 min.

^{1}H NMR (400 MHz, $CDCl_3$): δ 8.07 (d, *J* = 7.7 Hz, 2H), 7.68-7.62 (m, 2H), 7.54-7.49 (m, 2H), 7.48-7.42 (m, 3H), 7.41-7.36 (m, 4H), 7.31-7.25 (m, 3H), 7.23 (t, *J* = 7.4 Hz, 2H), 5.71 (s, 1H), 5.08 (d, *J* = 2.1 Hz, 2H), 3.54 (td, *J* = 6.6, 5.7, 3.3 Hz, 1H), 2.02-1.89 (m, 2H), 1.78-1.68 (m, 2H), 1.32 (d, *J* = 2.5 Hz, 6H); **^{13}C NMR** (100 MHz, $CDCl_3$): δ 166.9, 140.0, 135.8, 131.8, 131.2, 128.5, 128.2, 128.1, 126.7, 125.8, 123.2, 122.8, 120.4, 119.4, 109.0, 87.0, 82.8, 81.1, 75.8, 53.7, 37.4, 32.8, 30.8, 26.9, 24.1.

HRMS (ESI) m/z calcd. for $C_{36}H_{33}N_2O$ $[M+H]^+$ 509.2587, found 509.2586.

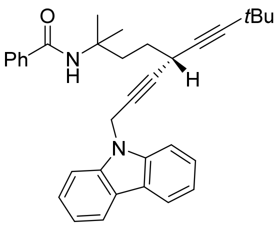

3zu

(*R*)-*N*-(5-(3-(9*H*-carbazol-9-yl)prop-1-yn-1-yl)-2,8,8-trimethylnon-6-yn-2-yl)benzamide (3zu)

According to general procedure D: Yellow oil, 58.4 mg (72%, 75% ee).

HPLC analysis: Chiralcel AD-H (*n*-hexane/*i*-PrOH = 90/10, flow rate 0.8 mL/min, λ = 230 nm), t_R (minor) = 11.20 min, t_R (major) = 17.91 min.

^{1}H NMR (400 MHz, $CDCl_3$): δ 8.06 (dt, *J* = 7.7, 0.9 Hz, 2H), 7.70-7.62 (m, 2H), 7.54-7.36 (m, 7H), 7.22 (dd *J* = 7.9, 7.0 Hz, 2H), 5.69 (s, 1H), 5.03 (d, *J* = 2.1 Hz, 2H), 3.27 (td, *J* = 5.7, 4.7, 3.4 Hz, 1H), 1.85-1.76 (m, 2H), 1.63-1.52 (m, 2H), 1.30 (s, 6H), 1.17 (s, 9H); **^{13}C NMR** (100 MHz, $CDCl_3$): δ 166.8, 140.0, 135.9, 131.1, 128.5, 126.7, 125.8, 123.2, 120.3, 119.4, 109.0, 90.1, 83.4, 76.2, 75.0, 53.7, 37.6, 32.9, 31.1, 27.3, 26.79, 26.77, 23.4.

HRMS (ESI) m/z calcd. for $C_{34}H_{37}N_2O$ $[M+H]^+$ 489.2900, found 489.2898.

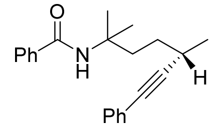

3zv

(*S*)-*N*-(2,5-dimethyl-7-phenylhept-6-yn-2-yl)benzamide (3zv)

According to general procedure C: Colorless oil, 32.1 mg (50%, 0% ee).

HPLC analysis: Chiralcel IA (*n*-hexane/*i*-PrOH = 98/2, flow rate 0.8 mL/min, λ = 254 nm), t_R (minor) = 28.03 min, t_R (major) = 30.70 min.

^{1}H NMR (400 MHz, $CDCl_3$): δ 7.75-7.64 (m, 2H), 7.48-7.36 (m, 5H), 7.31-7.24 (m, 3H), 5.91 (s, 1H), 2.66 (q, *J* = 6.9 Hz, 1H), 2.15-1.90 (m, 2H), 1.60-1.52 (m, 2H), 1.47 (d, *J* = 6.5 Hz, 6H), 1.27 (d, *J* = 6.9 Hz, 3H); **^{13}C NMR** (100 MHz, $CDCl_3$): δ 166.9, 135.9, 131.6, 131.1, 128.5, 128.2, 127.6, 126.7, 123.9, 94.4, 81.1, 54.0, 38.3, 31.7, 27.1, 27.0, 26.8, 21.1.

HRMS (ESI) m/z calcd. for $C_{22}H_{26}NO$ $[M+H]^+$ 320.2009, found 320.2006.

Removing the amide group to afford indirect enantioselective oxidative C(sp^3)–C(sp) coupling

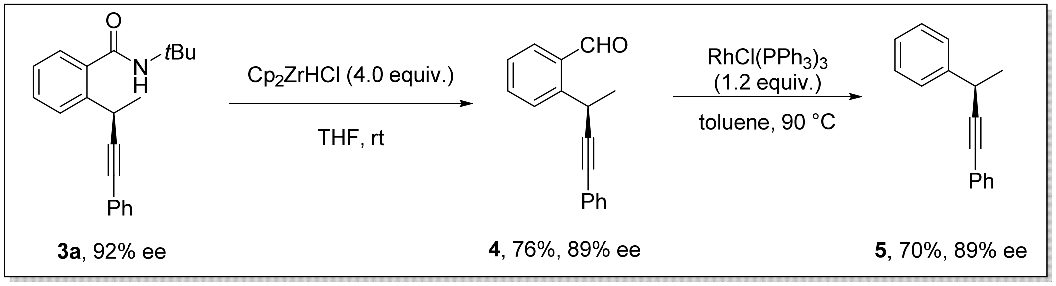

Supplementary Figure 244 Removing the amide group

In a glovebox, the Schwartz reagent (103.2 mg, 0.4 mmol) and a stir bar were added to a Schlenk tube, which was then sealed with a rubber septum and removed from the box. Next, amide **3a** (30.5 mg, 0.10 mmol) in anhydrous THF (2.0 mL) was added into the tube via syringe and the heterogeneous mixture was stirred vigorously at rt for 4 h. The resulting reaction mixture was then transferred to a separatory funnel and diluted with 20 mL water. The resulting mixture was extracted with DCM and the combined organic layers were dried over anhydrous $MgSO_4$. After removal of the solvent under reduced pressure, the crude product was purified by column chromatography on silica gel to give the corresponding aldehyde **4** (17.8 mg, 76%, 89% ee) as a colorless oil.

Under argon atmosphere, an oven-dried Schlenk tube equipped with a magnetic stir bar was charged with aldehyde **4** (11.7 mg, 0.050 mmol), $RhCl(PPh_3)_3$ (55.5 mg, 0.060 mmol), and anhydrous toluene (1.0 mL). Then, the mixture was stirred at 90 °C for 12 h. The reaction mixture was filtered through celite and washed with EtOAc. After removal of solvent, the residue was purified by silica gel column chromatography to give alkyne **5** (7.1 mg, 70%, 89% ee) as a colorless oil.

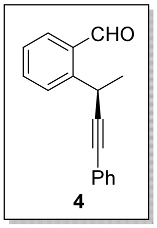

(*S*)-2-(4-phenylbut-3-yn-2-yl)benzaldehyde (4)

HPLC analysis: Chiralcel AD-H (*n*-hexane/*i*-PrOH = 90/10, flow rate 0.8 mL/min, λ = 254 nm), t_R (minor) = 7.25 min, t_R (major) = 8.22 min.

^{1}H NMR (400 MHz, $CDCl_3$): δ 10.30 (s, 1H), 7.89 (dd, *J* = 7.8, 1.2 Hz, 1H), 7.82 (dd, *J* = 7.6, 1.5 Hz, 1H), 7.62 (td, *J* = 7.6, 1.5 Hz, 1H), 7.50-7.40 (m, 3H), 7.34-7.26 (m, 3H), 5.09 (q, *J* = 7.0 Hz, 1H), 1.59 (d, *J* = 7.0 Hz, 3H); **^{13}C NMR** (100 MHz, $CDCl_3$): δ 192.7, 145.6, 134.2, 133.6, 132.4, 131.6, 128.8, 128.3, 127.9, 127.3, 123.5, 92.3, 82.7, 28.2, 24.6.

HRMS (ESI) m/z calcd. for $C_{17}H_{15}O$ $[M+H]^+$ 235.1117, found 235.1114.

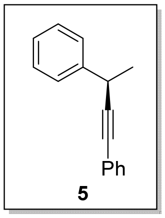

(-)-(*R*)-but-1-yne-1,3-diyldibenzene (5)

HPLC analysis: Chiralcel OD-H (*n*-hexane/*i*-PrOH = 99.5/0.5, flow rate 1.0 mL/min, λ = 254 nm), t_R (minor) = 13.60 min, t_R (major) = 19.33 min.

$[\alpha]_D^{27} = -12.8$ (*c* 0.71, $CHCl_3$).
^{1}H NMR (400 MHz, $CDCl_3$): δ 7.49-7.41 (m, 4H), 7.39-7.27 (m, 5H), 7.27-7.21 (m, 1H), 3.98 (q, *J* = 7.1 Hz, 1H), 1.58 (d, *J* = 7.2 Hz, 3H); **^{13}C NMR** (100 MHz, $CDCl_3$) δ 143.4, 131.7, 128.6, 128.2, 127.8, 127.0, 126.7, 123.7, 92.6, 82.5, 32.5, 24.6.
HRMS (ESI) *m/z* calcd. for $C_{16}H_{15}$ $[M+H]^+$ 207.1168, found 207.1161.

Versatile transformations for construction of chiral C(*sp*^{3})–C(*sp*^{2}) and C(*sp*^{3})–C(*sp*^{3}) bonds

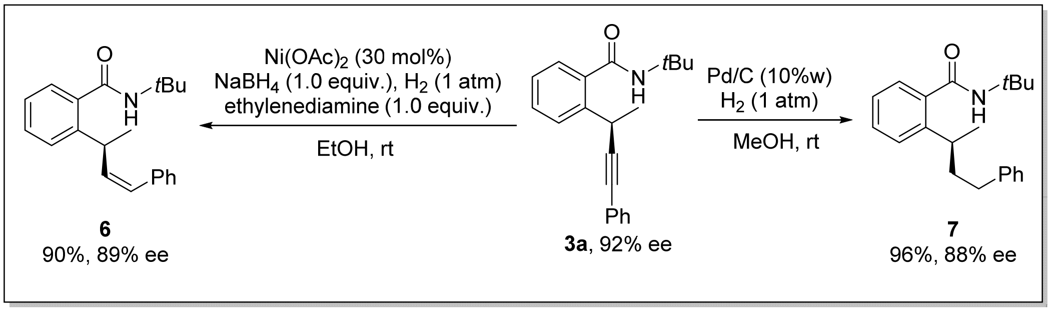

Supplementary Figure 245 Construction of chiral C(*sp*^{3})–C(*sp*^{2}) and C(*sp*^{3})–C(*sp*^{3}) bonds

To a solution of $Ni(OAc)_2 \cdot H_2O$ (3.7 mg, 0.015 mmol) in EtOH (0.5 mL) under H_2 atmosphere (1 atm) was added a solution of $NaBH_4$ (1.9 mg, 0.05 mmol) in EtOH (0.5 mL) at rt. After stirring for 1 h, a solution of amide **3a** (15.3 mg, 0.05 mmol) and ethylenediamine (3.0 mg, 0.05 mmol) in EtOH (0.5 mL) was added and the reaction was stirred overnight. Solvent was evaporated and the residue was purified by column chromatography on silica gel to give product **6** (13.8 mg, 90%, 89% ee) as a colorless oil.

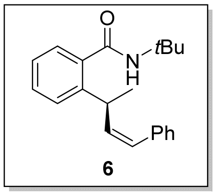

(*S,Z*)-*N*-(*tert*-butyl)-2-(4-phenylbut-3-en-2-yl)benzamide (6)
HPLC analysis: Chiralcel AD3 (*n*-hexane/*i*-PrOH = 98/2, flow rate 0.8 mL/min, λ = 254 nm), t_R (minor) = 16.19 min, t_R (major) = 18.13 min.
^{1}H NMR (400 MHz, $CDCl_3$): δ 7.44 (dd, *J* = 8.0, 1.2 Hz, 1H), 7.35 (td, *J* = 7.6, 1.6 Hz, 1H), 7.31-7.25 (m, 3H), 7.24-7.15 (m, 4H), 6.42 (d, *J* = 11.6 Hz, 1H), 5.95 (dd, *J* = 11.6, 9.8 Hz, 1H), 5.40 (s, 1H), 4.49-4.36 (m, 1H), 1.45 (d, *J* = 6.8 Hz, 3H), 1.27 (s, 9H); **^{13}C NMR** (100 MHz, $CDCl_3$): δ 169.6, 144.3, 137.5, 137.2, 136.7, 129.8, 128.7, 128.4, 128.3, 127.10, 127.06, 126.8, 125.9, 51.6, 35.0, 28.6, 24.4.
HRMS (ESI) m/z calcd. for $C_{21}H_{26}NO$ $[M+H]^+$ 308.2009, found 308.2006.

A 10 mL Schlenk tube was charged with amide **3a** (15.3 mg, 0.050 mmol) and palladium on carbon (10%w, 2.0 mg). The tube was evacuated and refilled with hydrogen through a hydrogen balloon. After addition of 0.5 mL of methanol, the mixture was stirred at rt for 24 h under hydrogen. The reaction mixture was filtered through celite and washed with EtOAc. After removal of solvent, the residue was purified by silica gel chromatography to give 14.8 mg (96%, 88%ee) of amide **7** as a colorless oil.

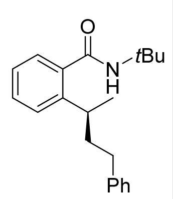

7

(*S*)-*N*-(*tert*-butyl)-2-(4-phenylbutan-2-yl)benzamide (7)

HPLC analysis: Chiralcel AD-H (*n*-hexane/*i*-PrOH = 90/10, flow rate 0.8 mL/min, λ = 214 nm), t_R (minor) = 7.50 min, t_R (major) = 13.52 min.

^{1}H NMR (400 MHz, $CDCl_3$): δ 7.40-7.31 (m, 2H), 7.29-7.21 (m, 3H), 7.20-7.09 (m, 4H), 5.50 (s, 1H), 3.28-3.17 (m, 1H), 2.66-2.53 (m, 1H), 2.51-2.40 (m, 1H), 2.04-1.83 (m, 2H), 1.43 (s, 9H), 1.30 (d, *J* = 6.9 Hz, 3H); **^{13}C NMR** (100 MHz, $CDCl_3$): δ 170.0, 144.8, 142.5, 138.1, 129.7, 128.29, 128.27, 126.4, 126.3, 125.68, 125.66, 51.8, 39.8, 35.2, 34.2, 28.9, 22.8.

HRMS (ESI) m/z calcd. for $C_{21}H_{28}NO$ $[M+H]^+$ 310.2165, found 310.2160.

Synthesis of chiral terminal alkyne building blocks

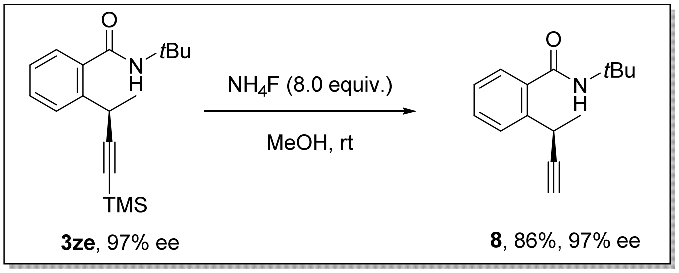

Supplementary Figure 246 Synthesis of chiral terminal alkyne building blocks

To a solution of **3ze** (30.1 mg, 0.10 mmol) in MeOH (2.0 mL) was added NH_4F (29.6 mg, 0.8 mmol), and the reaction was stirred at rt for 24 h. After removal of solvent, the residue was purified by silica gel chromatography to give amide **8** (19.7 mg, 86%, 97% ee) as a colorless solid.

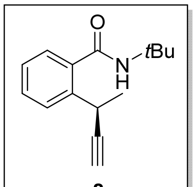

8

(*S*)-2-(but-3-yn-2-yl)-*N*-(*tert*-butyl)benzamide (8)

HPLC analysis: Chiralcel OD-3 (*n*-hexane/*i*-PrOH = 98/2, flow rate 0.8 mL/min, λ = 214 nm), t_R (minor) = 8.86 min, t_R (major) = 10.06 min.

^{1}H NMR (400 MHz, $CDCl_3$): δ 7.65 (d, *J* = 7.8 Hz, 1H), 7.40 (t, *J* = 7.6 Hz, 1H), 7.31 (d, *J* = 7.4 Hz, 1H), 7.26-7.21 (m, 1H), 5.72 (s, 1H), 4.29 (dd, *J* = 7.1, 2.7 Hz, 1H), 2.22 (d, *J* = 2.6 Hz, 1H), 1.54 (d, *J* = 7.0 Hz, 3H), 1.47 (s, 9H); **^{13}C NMR** (100 MHz, $CDCl_3$): δ 169.2, 140.5, 136.5, 130.1, 127.7, 126.8, 87.7, 69.8, 52.0, 28.8, 28.1, 23.8.

HRMS (ESI) m/z calcd. for $C_{15}H_{20}NO$ $[M+H]^+$ 230.1539, found 230.1536.

Radical inhibition experiments

Supplementary Table 2 Radical inhibition experiments

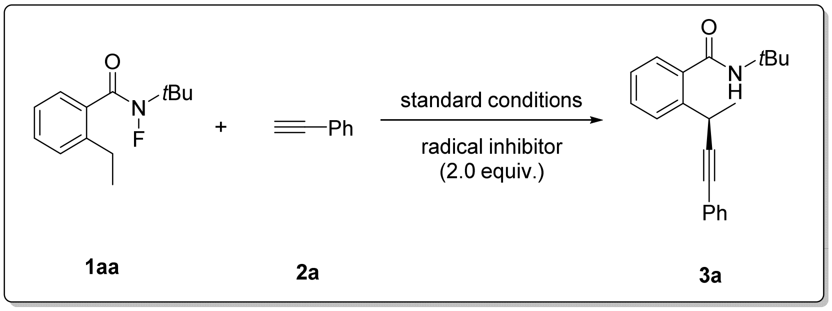

entry	additive	yield
1	no	88%
2	BHT	trace
3	TEMPO	trace

Under argon atmosphere, an oven-dried resealable Schlenk tube equipped with a magnetic stir bar was charged with CuI (1.9 mg, 0.010 mmol, 10 mol%), **L5** (6.1 mg, 0.010mmol, 10 mol%), Cs_2CO_3 (32.3 mg, 0.10 mmol, 1.0 equiv.), additive (0.20 mmol), and anhydrous THF (1.2 mL). Then, *N*-fluorocarocarboxamide **1aa** (0.10 mmol, 1.0 equiv.) and alkyne (0.20 mmol, 2.0 equiv.) were sequentially added into the mixture and the reaction mixture was stirred at rt for 16 h. Then, the precipitate was filtered off and washed by DCM. The filtrate was evaporated and the yield of product **3a** was determined by ^{1}H NMR analysis using CH_2Br_2 as an internal standard.

Synthesis of the clock substrate (±)-9:

(±)-9 was synthesized according to the literature procedures.[5]

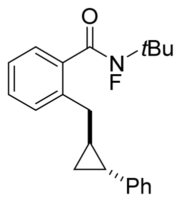

(±)-**9**

***N*-(tert-butyl)-*N*-fluoro-2-((*trans*-2-phenylcyclopropyl)methyl)benzamide ((±)-9)**

Yellow oil, 400 mg (27% yield). **^{1}H NMR** (400 MHz, $CDCl_3$): δ 7.42-7.30 (m, 3H), 7.26-7.19 (m, 3H), 7.14-7.09 (m, 1H), 7.07-7.01 (m, 2H), 2.94-2.76 (m, 2H), 1.81 (dt, *J* = 8.6, 4.9 Hz, 1H), 1.54 (d, *J* = 1.9 Hz, 9H), 1.45-1.34 (m, 1H), 1.01-0.91 (m, 2H); **^{13}C NMR** (100 MHz, $CDCl_3$): δ 174.9 (d, *J* = 10.7 Hz), 143.2, 138.8 (d, *J* = 2.0 Hz), 134.8, 130.1, 129.2, 128.3, 127.2 (d, *J* = 4.5 Hz), 125.8, 125.7, 125.4, 64.4 (d, *J* = 10.5 Hz), 36.8, 27.2 (d, *J* = 5.7 Hz), 23.6, 23.5, 16.2; **^{19}F NMR** (376 MHz, $CDCl_3$): δ -63.28.

HRMS (ESI) m/z calcd. for $C_{21}H_{25}FNO$ $[M+H]^+$ 325.1915, found 325.1912.

Radical clock experiment

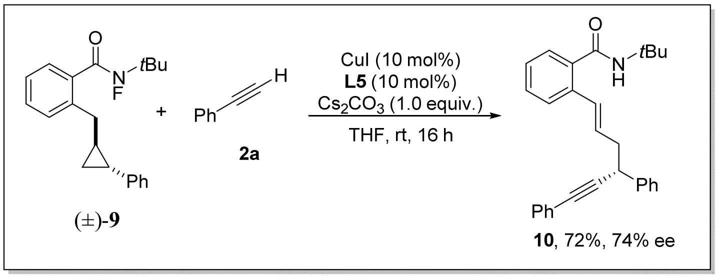

Supplementary Figure 247 Radical clock experiment

Under argon atmosphere, an oven-dried resealable Schlenk tube equipped with a magnetic stir bar was charged with CuI (3.8 mg, 0.020 mmol, 10 mol%), **L5** (12.2 mg, 0.020mmol, 10 mol%), Cs_2CO_3 (65.2 mg, 0.20 mmol, 1.0 equiv.), and anhydrous THF (2.4 mL). Then, *N*-fluorocarocarboxamide (±)-**9** (65 mg, 0.20 mmol, 1.0 equiv.) and phenylacetylene **2a** (40.8 mg, 0.40 mmol, 2.0 equiv.) were sequentially added into the mixture and the reaction mixture was stirred at rt for 16 h. Upon completion (monitored by TLC), the precipitate was filtered off and washed by DCM. The filtrate was evaporated and the residue was purified by column chromatography on silica gel to afford the desired product **10** (58.9 mg, 72%, 74% ee).

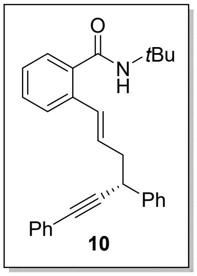

(*R*,*E*)-*N*-(*tert*-butyl)-2-(4,6-diphenylhex-1-en-5-yn-1-yl)benzamide (10)
HPLC analysis: Chiralcel OZ3 (*n*-hexane/*i*-PrOH = 80/20, flow rate 0.8 mL/min, λ = 254 nm), t_R (minor) = 7.53 min, t_R (major) = 11.43 min.
Colorless oil, **^{1}H NMR** (400 MHz, $CDCl_3$): δ 7.51-7.41 (m, 5H), 7.40-7.31 (m, 4H), 7.31-7.18 (m, 5H), 6.80-6.74 (m, 1H), 6.28 (dt, *J* = 15.7, 7.1 Hz, 1H), 5.55 (s, 1H), 4.00 (t, *J* = 7.1 Hz, 1H), 2.77 (td, *J* = 7.2, 1.5 Hz, 2H), 1.41 (s, 9H); **^{13}C NMR** (100 MHz, $CDCl_3$): δ 169.0, 141.3, 136.4, 135.1, 131.7, 130.1, 129.73, 129.70, 128.6, 128.3, 127.9, 127.6, 127.3, 127.2, 127.0, 126.5, 123.6, 90.8, 84.1, 51.9, 42.2, 38.9, 28.9.
HRMS (ESI) m/z calcd. for $C_{29}H_{30}NO$ $[M+H]^+$ 408.2322, found 408.2314.

Intramolecular kinetic isotope effect (KIE) study

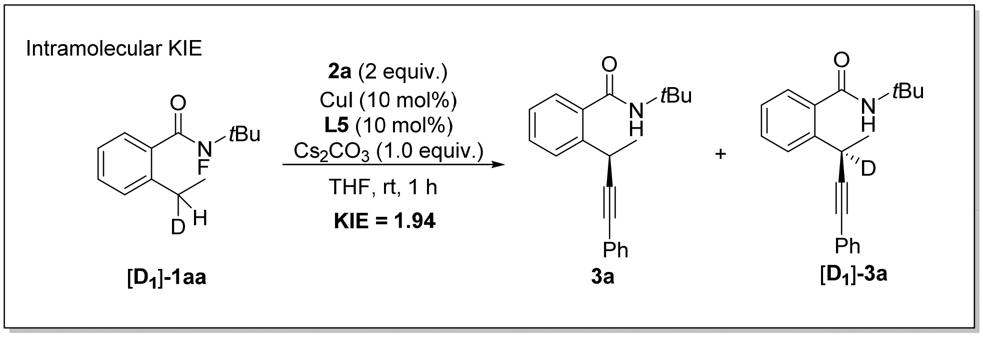

Supplementary Figure 248 Intramolecular kinetic isotope effect (KIE) study

According to the General Procedure A, the intramolecular KIE experiment was performed using

[D$_1$]-1aa (0.1 mmol, run in triplicate) for 1 h. The crude reaction mixture was analyzed by ^{1}H NMR, and the intramolecular KIE was calculated by the observed ratio of **[D$_1$]-3a/3a**, with a range from 1.78 to 2.14.

Intermolecular KIE study

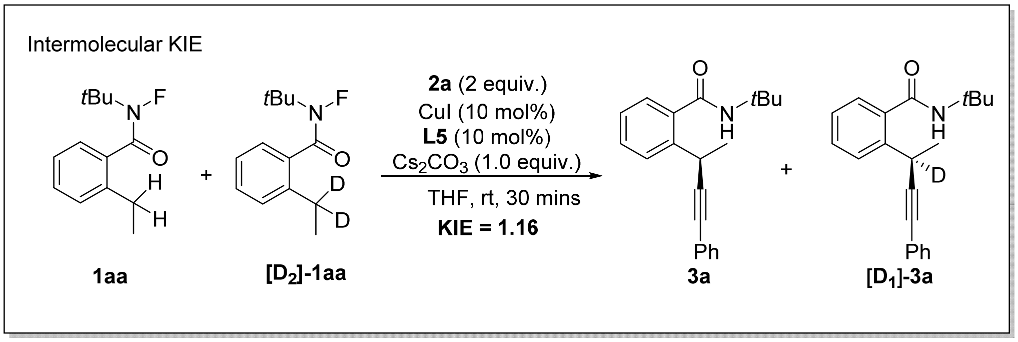

Supplementary Figure 249 Intermolecular KIE study

According to the General Procedure A, the intermolecular KIE experiment was performed using a 1:1 mixture of **1aa** and [**D$_2$**]**-1aa** (0.05 mmol each, run in triplicate) for 30 mins. The crude reaction mixture was analyzed by ^{1}H NMR, and the intramolecular KIE was calculated by the observed ratio of **3a/[D$_1$]-3a**, with a range from 1.11 to 1.27.

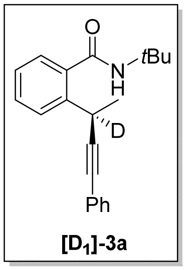

(*S*)-*N*-(*tert*-butyl)-2-(4-phenylbut-3-yn-2-yl-2-d)benzamide ([D$_1$]-3a)
According to general procedure A: 55.0 mg (85% yield).
Colorless oil, **^{1}H NMR** (400 MHz, $CDCl_3$): δ 7.72 (dd, *J* = 8.0, 1.2 Hz, 1H), 7.46-7.36 (m, 3H), 7.33 (dd, *J* = 7.6, 1.6 Hz, 1H), 7.30-7.23 (m, 4H), 5.76 (s, 1H), 1.60 (s, 3H), 1.47 (s, 9H); **^{13}C NMR** (100 MHz, $CDCl_3$): δ 169.3, 141.1, 136.6, 131.6, 130.1, 128.2, 127.9, 127.8, 126.8, 126.7, 123.7, 93.2, 82.1, 52.0, 28.8, 28.4(t, *J* = 19.5 Hz), 24.0.
HRMS (ESI) m/z calcd. for $C_{21}H_{23}DNO$ $[M+H]^+$ 307.1915, found 307.1911.

Control experiment with copper phenylacetylide

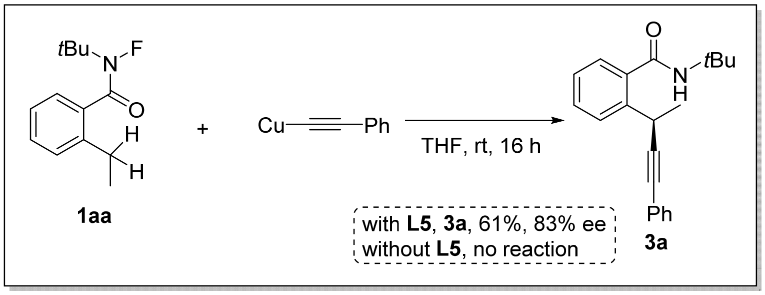

Supplementary Figure 250 Control experiment with copper phenylacetylide

Copper phenylacetylide was synthesized according to literature procedures.[11]
Under argon atmosphere, an oven-dried resealable Schlenk tube equipped with a magnetic stir bar was charged with copper phenylacetylide (16.5 mg, 0.10 mmol, 1.0 equiv.), *N*-fluorocarocarboxamide **1aa** (22.3 mg, 0.10 mmol, 1.0 equiv.), **L5** (61.1 mg, 0.10 mmol, 1.0 equiv.), and anhydrous THF (1.2 mL). The resulting reaction mixture was stirred at rt for 16 h. Then, the reaction mixture was filtered and washed by DCM. The filtrate was concentrated and the residue was purified by column chromatography on silica gel (petroleum ether) to afford **3a** (18.3 mg, 61% yield, 83% ee).
The procedure for the reaction without **L5** was the same with that described above except that **L5** was not added. No desired product **3a** was observed.

Supplementary References:

1 Guisán-Ceinos, M., Martín-Heras, V. & Tortosa, M. Regio- and stereospecific copper-catalyzed substitution reaction of propargylic ammonium salts with aryl Grignard reagents. *J. Am. Chem. Soc.* **139**, 8448–8451 (2017).

2 García Ruano, J. L., Marzo, L., Marcos, V., Alvarado, C. & Alemán, J. Highly stereoselective synthesis of tertiary propargylic centers and their isomerization to enantiomerically enriched allenes. *Chem. Eur. J.* **18**, 9775–9779 (2012).

3 Caeiro, J., Sestelo, J. P. & Sarandeses, L. A. Enantioselective nickel-catalyzed cross-coupling reactions of trialkynylindium reagents with racemic secondary benzyl bromides. *Chem. Eur. J.* **14**, 741–746 (2008).

4 Osako, T., Nagaosa, M., Hamasaka, G. & Uozumi, Y. Asymmetric copper-catalyzed C(sp)−H bond insertion of carbenoids derived from N-tosylhydrazones. *synlett* **29**, 2551–2556 (2018).

5 Groendyke, B. J., AbuSalim, D. I. & Cook, S. P. Iron-catalyzed, fluoroamide-directed C–H fluorination. *J. Am. Chem. Soc.* **138**, 12771−12774 (2016).

6 Miles, K. C., Le, C. C. & Stambuli, J. P. Direct carbocyclizations of benzoic acids: catalyst-controlled synthesis of cyclic ketones and the development of tandem aHH (acyl Heck−Heck) reactions. *Chem. Eur. J.* **20**, 11336−11339 (2014).

7 Ling, P.-X. et al. Palladium-catalyzed arylation of unactivated γ-methylene $C(sp^3)$−H and *δ*-C−H bonds with an oxazoline-carboxylate auxiliary. *Chem. Eur. J.* **21**, 17503−17507 (2015).

8 Wang, D. et al. Asymmetric copper-catalyzed intermolecular aminoarylation of styrenes: efficient access to optical 2,2-diarylethylamines. *J. Am. Chem. Soc.* **139**, 6811−6814 (2017).

9 Chen, H. Guo, L. & Yu, S. Primary, secondary, and tertiary *γ*-$C(sp^3)$−H vinylation of amides via organic photoredox-catalyzed hydrogen atom transfer. *Org. Lett.* **20**, 6255−6259 (2018).

10 Kamata, K., Yamaguchi, S., Kotani, M., Yamaguchi, K. & Mizuno, N. Efficient oxidative alkyne homocoupling catalyzed by a monomeric dicopper-substituted silicotungstate. *Angew. Chem., Int. Ed.* **47**, 2407–2410 (2008).

11 Sagadevan, A., Ragupathi, A. & Hwang, K. C. Photoinduced copper-catalyzed regioselective synthesis of indoles: three-component coupling of arylamines, terminal alkynes, and quinones. *Angew. Chem., Int. Ed.* **54**, 13896−13901 (2015).
